# Posicionamento da Sociedade Brasileira de Cardiologia para Gravidez e Planejamento Familiar na Mulher Portadora de Cardiopatia – 2020

**DOI:** 10.36660/abc.20200406

**Published:** 2020-05-22

**Authors:** Walkiria Samuel Avila, Elizabeth Regina Giunco Alexandre, Marildes Luiza de Castro, Alexandre Jorge Gomes de Lucena, Celi Marques-Santos, Claudia Maria Vilas Freire, Eduardo Giusti Rossi, Felipe Favorette Campanharo, Ivan Romero Rivera, Maria Elizabeth Navegantes Caetano Costa, Maria Alayde Mendonça Rivera, Regina Coeli Marques de Carvalho, Alexandre Abzaid, Antonio Fernandes Moron, Auristela Isabel de Oliveira Ramos, Carlos Japhet da Mata Albuquerque, Claudine Maia Alves Feio, Daniel Born, Fábio Bruno da Silva, Fernando Souza Nani, Flavio Tarasoutchi, José de Ribamar Costa, José Xavier de Melo, Leila Katz, Maria Cristina Costa Almeida, Max Grinberg, Melania Maria Ramos de Amorim, Nilson Roberto de Melo, Orlando Otávio de Medeiros, Pablo Maria Alberto Pomerantzeff, Sérgio Luiz Navarro Braga, Sonia Conde Cristino, Tania Leme da Rocha Martinez, Tatiana de Carvalho Andreuci Torres Leal

**Affiliations:** 1 Instituto do Coração Hospital das Clínicas da Faculdade de Medicina Universidade de São Paulo São PauloSP Brasil Instituto do Coração (Incor) do Hospital das Clínicas da Faculdade de Medicina da Universidade de São Paulo (HC-FMUSP),São Paulo, SP – Brasil; 2 Hospital do Coração São PauloSP Brasil Hospital do Coração (HCor),São Paulo, SP – Brasil; 3 Hospital das Clínicas da Faculdade de Medicina Universidade Federal de Minas gerais Belo HorizonteMG Brasil Hospital das Clínicas da Faculdade de Medicina da Universidade Federal de Minas gerais (UFMG),Belo Horizonte, MG – Brasil; 4 Hospital Agamenom Magalhães RecifePE Brasil Hospital Agamenom Magalhães,Recife, PE – Brasil; 5 Universidade Tiradentes AracajuSE Brasil Universidade Tiradentes,Aracaju, SE – Brasil; 6 Hospital São Lucas Rede D'Or Aracaju AracajuSE Brasil Hospital São Lucas, Rede D'Or Aracaju,Aracaju, SE – Brasil; 7 Universidade Federal de Minas Gerais Belo HorizonteMG Brasil Universidade Federal de Minas Gerais (UFMG),Belo Horizonte, MG – Brasil; 8 Universidade Federal de São Paulo Escola Paulista de Medicina São PauloSP Brasil Universidade Federal de São Paulo (UNIFESP), Escola Paulista de Medicina (EPM),São Paulo, SP – Brasil; 9 Hospital Israelita Albert Einstein São PauloSP Brasil Hospital Israelita Albert Einstein,São Paulo, SP – Brasil; 10 Universidade Federal de Alagoas MaceióAL Brasil Universidade Federal de Alagoas,Maceió, AL – Brasil; 11 Cardio Diagnóstico BelémPA Brasil Cardio Diagnóstico,Belém, PA – Brasil; 12 Centro Universitário Metropolitano da Amazônia BelémPA Brasil Centro Universitário Metropolitano da Amazônia (UNIFAMAZ),Belém, PA – Brasil; 13 Centro Universitário do Estado Pará BelémPA Brasil Centro Universitário do Estado Pará (CESUPA),Belém, PA – Brasil; 14 Secretaria de Saúde do Estado do Ceará FortalezaCE Brasil Secretaria de Saúde do Estado do Ceará,Fortaleza, CE – Brasil; 15 Instituto Dante Pazzanese de Cardiologia São PauloSP Brasil Instituto Dante Pazzanese de Cardiologia,São Paulo, SP – Brasil; 16 Hospital Metropolitano Oeste Pelópitas Silveira RecifePE Brasil Hospital Metropolitano Oeste Pelópitas Silveira,Recife, PE – Brasil; 17 Instituto de Medicina Integral Professor Fernando Figueira RecifePE Brasil Instituto de Medicina Integral Professor Fernando Figueira (IMIP),Recife, PE – Brasil; 18 Hospital Barão de Lucena RecifePE Brasil Hospital Barão de Lucena,Recife, PE – Brasil; 19 Hospital EMCOR RecifePE Brasil Hospital EMCOR,Recife, PE – Brasil; 20 Diagnósticos do Coração LTDA RecifePE Brasil Diagnósticos do Coração LTDA,Recife, PE – Brasil; 21 Universidade Federal do Pará BelémPA Brasil Universidade Federal do Pará,Belém, PA – Brasil; 22 UDI Hospital, Rede D'Or São LuísMA Brasil UDI Hospital, Rede D'Or,São Luís, MA – Brasil; 23 Centro Universitário de Belo Horizonte Belo HorizonteMG Brasil Centro Universitário de Belo Horizonte,Belo Horizonte, MG – Brasil; 24 Departamento de Obstetrícia e Ginecologia Faculdade de Medicina Universidade de São Paulo São PauloSP Brasil Departamento de Obstetrícia e Ginecologia da Faculdade de Medicina da Universidade de São Paulo (USP),São Paulo, SP – Brasil; 25 Ministério da Saíd BrasíliaDF Brasil Ministério da Saúde,Brasília, DF – Brasil; 26 Universidade de São Paulo São PauloSP Brasil Universidade de São Paulo (USP),São Paulo, SP – Brasil

**Realização:** Departamento de Cardiologia da Mulher da Sociedade Brasileira de Cardiologia

**Conselho de Normatizações e Diretrizes (2020-2021):** Brivaldo Markman Filho, Antonio Carlos Sobral Sousa, Aurora Felice Castro Issa, Bruno Ramos Nascimento, Harry Correa Filho, Marcelo Luiz Campos Vieira

**Coordenador de Normatizações e Diretrizes (2020-2021):** Brivaldo Markman Filho

**Coordenadoras do Posicionamento:** Walkiria Samuel Avila, Elizabeth Regina Giunco Alexandre, Marildes Luiza de Castro

**Comitê de Redação:** Alexandre Jorge Gomes de Lucena, Celi Marques-Santos, Claudia Maria Vilas Freire, Eduardo Giusti Rossi, Elizabeth Regina Giunco Alexandre, Felipe Favorette Campanharo, Ivan Romero Rivera, Maria Elizabeth Navegantes Caetano Costa, Maria Alayde Mendonça Rivera, Marildes Luiza de Castro, Regina Coeli Marques de Carvalho, Walkiria Samuel Avila

**Table:** 

Posicionamento da Sociedade Brasileira de Cardiologia para Gravidez e Planejamento Familiar na Mulher Portadora de Cardiopatia – 2020 Declaração de potencial conflito de interesses dos autores/colaboradores do Posicionamento:							

Nomes Integrantes do posicionamento	Participou de estudos clínicos e/ou experimentais subvencionados pela indústria farmacêutica ou de equipamentos relacionados ao posicionamento em questão	Foi palestrante em eventos ou atividades patrocinadas pela indústria relacionados ao posicionamento em questão	Foi (é) membro do conselho consultivo ou diretivo da indústria farmacêutica ou de equipamentos	Participou de comitês normativos de estudos científicos patrocinados pela indústria	Recebeu auxílio pessoal ou institucional da indústria	Elaborou textos científicos em periódicos patrocinados pela indústria	Tem ações da indústria
Alexandre Abzaid	Não	Não	Não	Boston Scientific, Medtronic, Edwards, Elixir	Não	Não	Não
Alexandre Jorge Gomes de Lucena	Não	Não	Não	Não	Não	Não	Não
Antonio Fernandes Moron	Não	Não	Não	Não	Não	Não	Não
Auristela Isabel de Oliveira Ramos	Não	Não	Não	Não	Não	Não	Não
Carlos Japhet da Matta Albuquerque	Não	Não	Não	Não	Não	Não	Não
Celi Marques-Santos	Não	Não	Não	Não	Não	Não	Não
Cláudia Maria Vilas Freire	Não	Não	Não	Não	Não	Não	Não
Claudine Maia Alves Feio	Não	Não	Não	Não	Não	Não	Não
Daniel Born	Não	Não	Não	Não	Não	Não	Não
Eduardo Giusti Rossi	Não	Não	Não	Não	Não	Não	Não
Elizabeth Regina Giunco Alexandre	Não	Não	Não	Não	Novo Nordisk, Servier, AstraZeneca	Não	Não
Fábio Bruno da Silva	Não	Não	Não	Não	Não	Não	Não
Felipe Favorette Campanharo	Não	Não	Não	Não	Não	Não	Não
Fernando Souza Nani	Não	Boehringer	Não	Não	Não	Não	Não
Flavio Tarasoutchi	Não	Não	Não	Não	Não	Não	Não
Ivan Romero Rivera	Não	Não	Não	Não	Não	Não	Não
José de Ribamar Costa Junior	Não	Não	Não	Não	Não	Não	Não
José Xavier de Melo Filho	Não	Não	Não	Não	Servier, Novartis	Não	Não
Leila Katz	Não	Não	Não	Não	Não	Não	Não
Maria Alayde Mendonça Rivera	Não	Não	Não	Não	Não	Não	Não
Maria Cristina Costa Almeida	Não	Não	Não	Não	Não	Não	Não
Maria Elizabeth Navegantes Caetano Costa	Não	Não	Não	Não	Biolab, Pfizer, Boehringer	Não	Speak, Biolab, Bouehringer
Marildes Luiza de Castro	Não	Não	Não	Não	Não	Não	Não
Max Grinberg	Não	Não	Não	Não	Não	Não	Não
Melania Maria Ramos de Amorim	Não	Não	Não	Não	Não	Não	Não
Nilson Roberto de Melo	Não	Não	Não	Não	Não	Não	Não
Orlando Otávio de Medeiros	Não	Não	Não	Não	Não	Não	Não
Pablo Maria Alberto Pomerantzeff	Não	Não	Não	Não	Não	Não	Não
Regina Coeli Marques de Carvalho	Não	Não	Não	Não	Não	Não	Não
Sérgio Luiz Navarro Braga	Não	Não	Não	Não	Não	Não	Não
Sonia Conde Cristino	Não	Não	Não	Não	Não	Não	Não
Tania Leme da Rocha Martinez	Não	Não	Não	Não	Não	Não	Não
Tatiana de Carvalho Andreuci Torres Leal	Não	Não	Não	Não	Não	Não	Não
Walkiria Samuel Avila	Não	Não	Não	Não	Não	Não	Não

Sumário

1. Introdução 853

2. Aspectos Gerais 854

2.1. Modificações Fisiológicas da Gravidez, Parto e Puerpério 854

**2.1.1. Modificações Hemodinâmicas 854**

**2.1.2. Modificações da Coagulação Sanguínea 856**

**2.1.3. Modificações Respiratórias 856**

**2.1.4. Mudanças na Parede Vascular 857**

**2.1.5. Pontos-chaves 858**

2.2. Avaliação Materna e Fetal 858

**2.2.1. Avaliação Clínica Materna 858**

***2.2.1.1. Anamnese e Exame Físico* 858**

***2.2.1.2. Pontos-chaves* 858**

**2.2.2. Avaliação Obstétrica e Fetal 858**

***2.2.2.1. Pontos-chaves* 860**

2.3. Exames Subsidiários Isentos de Radiação 860

**2.3.1. Eletrocardiograma 860**

**2.3.2. Ecocardiograma 861**

**2.3.3. Monitoramento Ambulatorial da Pressão Arterial 861**

**2.3.4. Sistema Holter-24 Horas 861**

**2.3.5. Teste Ergométrico 862**

**2.3.6. Pontos-chaves 862**

2.4. Exames de Imagem com Radiação 862

**2.4.1. Administração de Agentes de Contrastes 863**

**2.4.2. Ressonância Magnética Nuclear 864**

**2.4.3. Pontos-chaves 864**

2.5. Fármacos de Ação Cardiovascular na Gravidez e no Aleitamento 864

**2.5.1. Anti-hipertensivos 865**

**2.5.2. Antiarrítmicos 866**

**2.5.3. Fármacos na Insuficiência Cardíaca 867**

**2.5.4. Antiplaquetários 867**

**2.5.5. Trombolíticos 868**

**2.5.6. Anticoagulantes 868**

**2.5.7. Hipolipemiantes 868**

**2.5.8. Pontos-chaves 869**

2.6. Princípios de Conduta Durante a Gravidez 870

**2.6.1. Estilo de Vida 870**

**2.6.2. Atividade Física 870**

**2.6.3. Dieta 870**

**2.6.4. Atividade Profissional 871**

**2.6.5. Pontos-chaves 871**

2.7. Conduta no Parto e Puerpério 871

**2.7.1. Conduta no Parto 871**

**2.7.2. Conduta no Puerpério 872**

**2.7.3. Pontos-chaves 873**

2.8. Anestesia na Gestante Cardiopata 873

**2.8.1. Jejum 874**

**2.8.2. Anticoagulação e Bloqueio do Neuroeixo 874**

**2.8.3. Heparina Não Fracionada (Subcutânea) 874**

**2.8.4. Heparina de Baixo Peso Molecular (Subcutânea) 874**

**2.8.5. Monitoramento Hemodinâmico 875**

**2.8.6. Uterotônicos Intraparto 875**

**2.8.7. Pós-parto 875**

**2.8.8. Pontos-chaves 875**

3. Avaliação e Conduta das Doenças Cardíacas Durante a Gravidez 875

3.1. Doença Valvar 875

**3.1.1. Considerações Gerais sobre a Terapêutica 876**

**3.1.2. Pontos-chaves: Gravidez em Valvopatias – Valva Nativa 876**

**3.1.3. Prótese Valvar 877**

**3.1.4. Risco Materno 878**

**3.1.5. Riscos para o Concepto 879**

**3.1.6. Pontos-chaves: Gravidez e Prótese Valvar 880**

3.2. Cardiopatias Congênitas 881

**3.2.1. Conduta na Gestação 882**

**3.2.2. Pontos-chaves 883**

3.3. Cardiomiopatias 883

**3.3.1. Cardiomiopatia Dilatada 883**

**3.3.2. Cardiomiopatia Hipertrófica 884**

**3.3.3. Displasia Arritmogênica do Ventrículo Direito 884**

**3.3.4. Cardiomiopatia Não Compactada 884**

**3.3.5. Cardiomiopatia Restritiva 884**

**3.3.6. Pontos-chaves 885**

**3.3.7. Cardiomiopatia Periparto 885**

***3.3.7.1. Pontos-chaves* 887**

3.4. Cardiopatia Isquêmica 887

**3.4.1. Pontos-chaves 888**

3.5. Dislipidemia na Gestação 888

**3.5.1. Alterações Lipídicas 888**

**3.5.2. Pontos-chaves 889**

3.6. Outras Doenças 889

**3.6.1. Arterite de Takayasu 889**

***3.6.1.1. Prevalência* 889**

***3.6.1.2. Prognóstico* 889**

***3.6.1.3. Tratamento* 889**

***3.6.1.4. Pontos-chaves* 890**

**3.6.2. Doença de Kawasaki 890**

***3.6.2.1. Avaliação Pré-concepção* 890**

***3.6.2.2. Pontos-chaves* 890**

**3.6.3. Hipertensão Pulmonar 890**

***3.6.2.1. Avaliação Pré-concepção* 890**

***3.6.2.2. Pontos-chaves* 890**

**3.6.3. Hipertensão Pulmonar 890**

***3.6.3.1. Pontos chaves* 891**

**3.6.4. Doenças da Aorta 891**

***3.6.4.1. Dissecção e Ruptura Aórtica* 892**

***3.6.4.2. Pontos-chaves* 893**

**3.6.5. Doença de Chagas 894**

***3.6.5.1. Prevalência* 894**

***3.6.5.2. Diagnóstico e Conduta da Infecção por T. Cruzi durante a gravidez* 894**

***3.6.5.3. Cardiopatia Chagásica Crônica* 894**

***3.6.5.4. Transmissão Vertical de Trypanosoma Cruzi* 894**

***3.6.5.5. Reativação da Doença de Chagas* 894**

***3.6.5.6. Aleitamento* 894**

***3.6.5.7. Pontos-chaves* 895**

4. Síndrome Hipertensiva da Gestação 895

4.1. Introdução 895

4.2. Recomendações para Aferição da Pressão Arterial 896

4.3. Classificação 896

**4.3.1. Hipertensão Crônica, Preexistente (Essencial ou Secundária) 896**

**4.3.2. Pré-eclâmpsia/Eclâmpsia 896**

***4.3.2.1 Síndrome HELLP (Hemólise, Elevação das Enzimas Hepáticas, Plaquetopenia)* 897**

**4.3.3. Hipertensão Crônica (Preexistente) com Pré-eclâmpsia Sobreposta 897**

**4.3.4. Hipertensão Gestacional 897**

***4.3.4.1. Pontos-chaves* 897**

4.4. Tratamento da Síndrome Hipertensiva Gestacional 897

**4.4.1. Tratamento Não Farmacológico 897**

**4.4.2. Quando Tratar – Alvo da Pressão Arterial 898**

**4.4.3. Drogas Anti-hipertensivos Orais – Hipertensão Crônica/Hipertensão Gestacional 898**

**4.4.4. Anti-hipertensivos na Emergência Hipertensiva (Hipertensão Grave/Pré-eclâmpsia) 899**

4.5. Conduta na Emergência Hipertensiva em Pré-eclâmpsia (PA ≥ 160/110 mmHg) 900

4.6. A Profilaxia da Crise Convulsiva na Pré-eclâmpsia – Eclâmpsia e Terapêutica com o de Sulfato de Magnésio 900

**4.6.1. Pontos-chaves 901**

4.7. Prognóstico e Prevenção da Pré-eclâmpsia 901

**4.7.1. Pontos-chaves 902**

4.8. Hipertensão Arterial No Puerpério 902

**4.8.1. Recomendações 902**

**4.8.2. Pontos-chaves 903**

4.9. Hipertensão na Gestação e Risco Cardiovascular Futuro 903

**4.9.1. Pontos-chaves 903**

5. Tratamento e Prevenção das Complicações Cardíacas 903

5.1. Arritmias Cardíacas 903

**5.1.1. Epidemiologia 903**

**5.1.2. Apresentação Clínica 903**

**5.1.3. Risco Materno-fetal 904**

**5.1.4. Tratamento 904**

**5.1.5. Pontos-chaves 905**

5.2. Tromboembolismo 905

**5.2.1. Epidemiologia 905**

**5.2.2. Fatores de Risco 905**

**5.2.3. Trombofilias 906**

**5.2.4. Diagnóstico 907**

***5.2.4.1. Trombose Venosa Profunda* 907**

***5.2.4.2. Dímero D* 908**

***5.2.4.3. Ultrassonografia Venosa* 908**

***5.2.4.4. Ressonância Magnética de Veias Ilíacas* 908**

***5.2.4.5. Tromboembolismo Pulmonar* 908**

***5.2.4.6. Diagnóstico Diferencial* 909**

**5.2.5. Tratamento 909**

***5.2.5.1. Consideração geral* 909**

***5.2.5.2. Uso da Heparina* 909**

***5.2.5.2.1. Doses Recomendadas* 909**

***5.2.5.2.2. Trabalho de Parto e Parto* 910**

***5.2.5.2.3. Puerpério* 910**

***5.2.5.2.4. Tempo de Anticoagulação* 910**

***5.2.5.3. Filtros de Veia Cava Inferior* 910**

***5.2.5.4. Trombólise* 911**

**5.2.6. Profilaxia 911**

**5.2.7. Pontos-chaves 911**

5.3. Tratamento e Prevenção 911

**5.3.1. Insuficiencia Cardíaca 911**

**5.3.2 Pontos-chaves 914**

5.4. Tratamento e Prevenção 914

**5.4.1. Endocardite Infecciosa 914**

**5.4.2. Doença Reumática 915**

**5.4.3. Pontos-chaves 915**

5.5. Cirurgia Cardiovascular na Gravidez 915

**5.5.1. Pontos-chaves 916**

5.6. Intervenção Cardíaca Percutânea 916

**5.6.1. Princípios Gerais 916**

**5.6.2. Intervenções Percutâneas Valvares 917**

***5.6.2.1. Valvoplastia por Cateter-Balão na Estenose Mitral* 917**

***5.6.2.2. Estenose Aórtica* 917**

***5.6.2.3. Estenose Congênita da Valva Pulmonar* 917**

***5.6.2.4. Implante Percutâneo de Próteses Valvares* 917**

***5.6.2.5. Procedimento de “Valve in Valve” na Disfunção de Prótese Biológica* 917**

***5.6.2.6. Angioplastia Coronariana* 917**

**5.6.3. Pontos-chaves 918**

5.7. Emergências Cardiológicas 918

**5.7.1. Insuficiência Cardíaca Aguda 918**

**5.7.2. Arritmia 919**

**5.7.3. Infarto Agudo do Miocárdio 920**

**5.7.4. Síndrome Aórtica Aguda 921**

**5.7.5. Trombose de Prótese Valvar 921**

**5.7.6. Parada Cardiorrespiratória 921**

**5.7.7. Pontos-chaves 922**

6. Planejamento Familiar 922

6.1. Aconselhamento à Gravidez e Estratificação de Risco Materno 922

**6.1.1. Pontos-chaves 924**

6.2. Contracepção Na Paciente com Doença Cardiovascular 924

**6.2.1. Diferentes Métodos Anticoncepcionais 924**

**6.2.2. Critérios de Elegibilidade Médica 925**

**6.2.3. Contracepção em Diferentes Condições 926**

***6.2.3.1. Hipertensão* 926**

***6.2.3.2. Diabetes Melito* 926**

***6.2.3.3. Doença Valvar* 927**

***6.2.3.4. Evento Cardiovascular Prévio* 927**

***6.2.3.5. Obesidade* 928**

***6.2.3.6. Cardiopatia Congênita* 928**

***6.2.3.7. Hipertensão Pulmonar* 928**

**6.2.4. Contracepção na Adolescência 928**

***6.2.4.1. Pontos-chaves* 929**

6.3. Aspectos Bioéticos 929

Referências 930

## 1. Introdução

O Departamento de Cardiologia da Mulher (DCM) apresenta este documento, elaborado de acordo com as normas estabelecidas pela Sociedade Brasileira de Cardiologia (SBC), com a finalidade de discutir sobre as patologias cardiovasculares mais prevalentes que acometem a mulher durante o ciclo gravídico-puerperal e para as quais não existem evidências substanciais ou ensaios clínicos randomizados.

Com o apoio da SBC, o então Departamento de Cardiopatia e Gravidez publicou, em 1999, o 1° Consenso sobre Cardiopatia e Gravidez e Planejamento Familiar, pioneiro no mundo, que atraiu a atenção para a evolução da gestação em cardiopatas, quando a máxima vigente era “mulheres com doenças cardíacas não devem engravidar porque a mortalidade materna é proibitiva”. Passados 10 anos, a experiência daquele departamento, hoje DCM, exigiu que fossem reconsideradas as restrições da gravidez em cardiopatas. Então, foi publicada, em 2009, a Diretriz para Gravidez da Mulher Portadora de Cardiopatia, divulgando as estratégias terapêuticas disponíveis na época, específicas e adequadas a cada situação clínica.

Após duas décadas da primeira publicação, o DCM revalida seu compromisso publicando o 1º Posicionamento para Gravidez e Planejamento Familiar na Mulher Cardiopata, resultado da experiência e do empenho de especialistas que trabalham na elaboração de protocolos que contribuem para decisões terapêuticas durante o período gestacional, bem como para aconselhamento no planejamento familiar da mulher cardiopata.

A taxa de mortalidade materna de um país é um dos mais sensíveis indicadores das condições de vida de uma população e reflete, particularmente, a qualidade da assistência de saúde prestada à mulher no pré-natal. Embora ainda aquém das metas estimadas para este milênio, nas três últimas décadas o Brasil registrou importante redução no coeficiente de mortalidade materna em decorrência de complicações durante o ciclo gravídico-puerperal.

Incidindo em 4% das gestações, a cardiopatia, por si só, continua sendo, no mundo, a principal causa não obstétrica de morte materna. Entretanto, o avanço da cardiologia no aperfeiçoamento dos métodos de diagnóstico e das alternativas terapêuticas tem favorecido uma mudança significativa no prognóstico das doenças cardiovasculares e nas características das cardiopatias que ocorrem na idade reprodutiva. Isso tem possibilitado maior expectativa e qualidade de vida das mulheres cardiopatas, proporcionando alento ao desejo de maternidade e segurança de uma gravidez com menor risco.

A medicina individualiza cada vez mais a abordagem das diversas patologias, sobretudo quanto ao gênero, uma vez que o organismo feminino difere, em muito, do masculino, particularmente durante o ciclo gravídico-puerperal.

A atualização deste documento vem ao encontro da responsabilidade universal quanto à melhora do prognóstico materno-fetal. Assim, é inegável que a experiência acumulada pelo DCM contribui para estabelecer protocolos norteadores de condutas terapêuticas durante a gravidez, aconselhar futuras gestações, melhorar a expectativa de vida com qualidade e reduzir a mortalidade materna por cardiopatia.

Em consonância com a literatura mundial, neste documento são discutidos os novos conceitos de cardiopatia *versus* gravidez, tais como: a estratificação dos riscos maternos fundamentada nas recomendações da Organização Mundial da Saúde (OMS); os aspectos da hipertensão arterial; o reforço da multidisciplinaridade, incluindo a participação do *Heart Team*; as propostas terapêuticas das principais complicações; as mudanças na classificação dos riscos materno-fetais no que diz respeito a fármacos utilizados na gravidez e lactação; e a contracepção.

O objetivo desta publicação é uniformizar condutas e disponibilizar mais uma ferramenta que seja útil no cotidiano da prática clínica. O DCM deseja que as recomendações e sugestões aqui contidas tenham repercussão em nível nacional, contribuindo para o melhor tratamento e consequente benefício na redução do risco cardiovascular da mulher portadora de cardiopatia durante o período reprodutivo.

## 2. Aspectos Gerais

### 2.1. Modificações Fisiológicas da Gravidez, Parto e Puerpério

A integração entre embrião e útero materno provoca no organismo um intrínseco estímulo hormonal que induz a transformações na fisiologia do sistema cardiovascular, as quais são fundamentais para o adequado desenvolvimento da gravidez.^1^ Essas mudanças, porém, determinam uma sobrecarga hemodinâmica que pode revelar doenças cardíacas previamente não reconhecidas ou agravar o estado funcional de cardiopatias subjacentes. Por isso, a compreensão das modificações hemodinâmicas, da coagulação sanguínea e respiratórias que ocorrem durante o ciclo gravídico-puerperal é fundamental para a interpretação do quadro clínico materno, predição dos riscos da gestaçao e avaliação da saúde fetal.

#### 2.1.1. Modificações Hemodinâmicas (Tabela 1)

**Tabela 1:** – Alterações hemodinâmicas da gestação

Parâmetro	Alteração
Débito cardíaco	Aumento de 30 a 50% (2 l/min)
Frequência cardíaca	Aumento de 15 a 20% (15 bpm)
Volume sanguíneo	Aumento de 20 a 30% (1,8 l)
Pressão arterial média	Redução de menos de 5%
Resistência vascular sistêmica	Redução de 20 a 30% (320 dinas-s/cm^5^)
Resistência vascular pulmonar	Redução de 30% (40 dinas-s/cm^5^)
Pressão venosa central	Inalterada
Pressão venosa de membros inferiores	Aumentada em 15%

**Figura 1:**
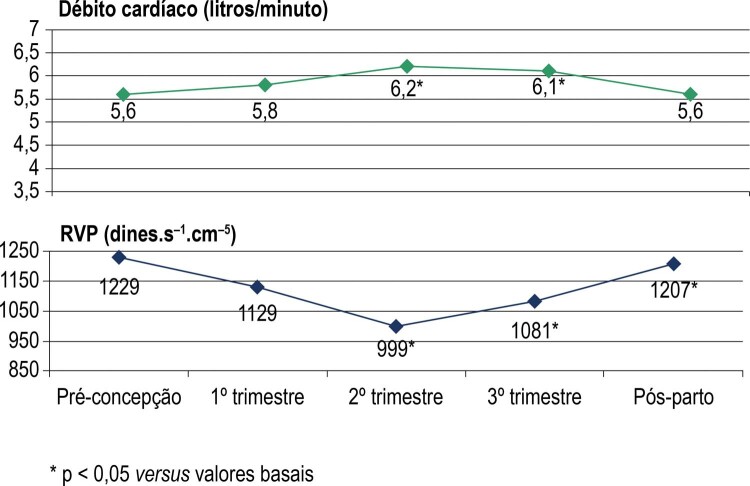
**–** Variação do débito cardíaco e da resistência vascular periférica (RVP) durante a gravidez e após o parto. Adaptada e traduzida de Sanghavi e Rutheford, 2014.^3^

O débito cardíaco, calculado pelo produto do volume sistólico e da frequência cardíaca, aumenta progressivamente, em média, 40% acima dos valores pré-gestacionais a partir do 1º trimestre, com alcance do maior incremento no início do 3º trimestre da gestação, tendendo a reduzir no termo^2^(Figura 1). A magnitude do aumento do débito cardíaco varia individualmente, sendo 15% maior na gravidez múltipla. O volume plasmático é o maior responsável pelo aumento do débito cardíaco na primeira metade da gestação. A partir de então, a frequência cardíaca, que habitualmente não ultrapassa 100 batimentos por minuto (bpm), desempenha papel importante nesse incremento até o termo da gestação.

A desproporção entre o aumento no volume plasmático e a produção de glóbulos vermelhos resulta na anemia dilucional ou fisiológica da gravidez, mais aparente no final do 2º trimestre da gestação, quando o volume plasmático alcança seu pico em relação ao volume de hemácias. Assumindo que a função renal seja normal, o volume sanguíneo e os constituintes retornam aos valores prévios à gestação, em razão da diurese, após oito semanas do parto, enquanto a hemoglobina se eleva a partir do terceiro dia do puerpério.^2,3^

No termo da gestação o volume sanguíneo é estimado em 100 ml/kg, quase duas vezes maior quando comparado aos 65 a 70 ml/kg em mulheres não grávidas. A massa de eritrócitos aumenta a partir da 8ª à 10ª semana de gestação, induzida pela elevação da eritropoetina no plasma.

Dentre os mecanismos hormonais utilizados para explicar a hipervolemia da gravidez, destacam-se: o estrógeno, que aumenta os níveis de renina, causando retenção de sódio e água corpórea total; a prolactina; o lactogeno placentário; as prostaglandinas; e o hormônio do crescimento.

Após a segunda metade da gestação, podem-se observar variações do débito cardíaco de repouso secundárias a mudanças na posição adotada pela gestante. A mudança de decúbito dorsal para lateral esquerdo, por exemplo, produz aumento de cerca de 22% do débito cardíaco, redução de aproximadamente 6% da frequência cardíaca e aumento de 27% do volume sistólico. A compressão da veia cava inferior pelo útero aumentado, na posição supina provoca a chamada síndrome da hipotensão supina, que pode se manifestar com tontura e/ou síncope.^4^

Durante a gravidez ocorre uma redução da pressão coloidosmótica plasmática em cerca de 12 a 18%, consequente à queda da concentração da albumina circulante, observada em níveis mais baixos durante a 24ª semana de gestação. Esse declínio piora o edema de membros inferiores e predispõe à congestão pulmonar gestantes e parturientes que recebem infusão intravenosa excessiva de cristaloides.^5^

A redução da resistência vascular periférica no início da gestação não é limitada ao plexo uterino e tem maior magnitude do que a concomitante elevação do débito cardíaco. Já na segunda metade da gestação, a resistência alcança os menores valores, momento em que o débito cardíaco chega ao seu máximo (Figura 1).^6^ A dilatação arteriolar da gravidez tem sido atribuída aos componentes estrogênicos, à prolactina e ao aumento dos níveis de prostaglandina circulante (PGE2 e PGI2), substância responsável pela redução da resposta vascular à angiotensina exógena.

Um decréscimo na síntese de prostaglandinas ou um aumento no seu metabolismo podem resultar em incremento da responsividade vascular à angiotensina II, uma característica observada em grávidas que desenvolvem hipertensão. A progesterona e seus metabólitos também parecem participar da modulação da resposta vascular à angiotensina II durante a gravidez. Recentemente, tem-se demonstrado que alterações no tônus vascular durante a gravidez podem ser atribuídas, em parte, a mudanças na síntese de substâncias vasoativas derivadas do endotélio, destacando-se a endotelina, que, teoricamente, é capaz de mediar a síntese das prostaglandinas, e a redução do óxido nítrico, que tem sido relacionado com a vasodilatação da gestação.^7^

É oportuno ressaltar que, durante a gestação, o sistema arterial sofre um remodelamento para acomodar o volume sanguíneo aumentado. O estrógeno proporciona uma deposição de colágeno no interior da camada média das grandes e médias artérias; a elastase circulante favorece a ruptura da lâmina elástica e o enfraquecimento da média da parede dos vasos; e a relaxina, hormônio fator de crescimento *insulina-like* (detectado no plasma), causa redução da síntese de colágeno. Todos esses fatores explicam a predisposição à dissecção arterial durante a gravidez.

**Figura 2:**
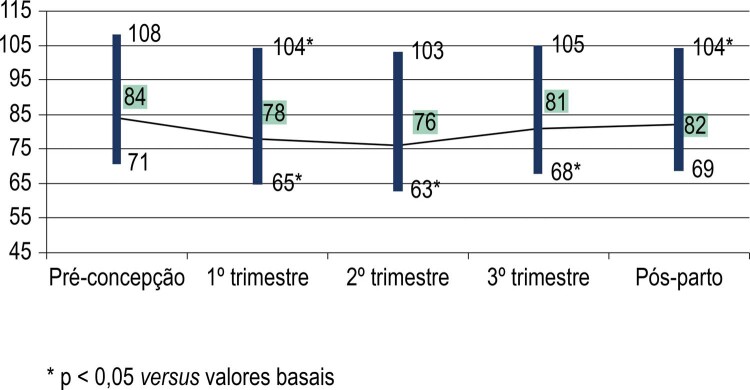
**–** Variação da pressão arterial sistêmica no ciclo gravídico-puerperal. Adaptada e traduzida de Sanghavi e Rutheford, 2014.^3^

A pressão arterial sistólica (PAS) diminui desde o início até a metade da gestação,^8^ particularmente à custa da pressão diastólica, para, posteriormente, elevar-se e alcançar os valores pré-gestacionais quando se aproxima o termo (Figura 2). A PAS eleva-se durante as contrações uterinas, principalmente no segundo estágio do trabalho de parto.

Ocasionalmente pode ocorrer um quadro de hipotensão ortostática secundário à redução do retorno venoso quando a gestante está na posição supina, com consequente queda do débito cardíaco. Considerando-se que o débito pulmonar é igual ao aórtico no adulto normal, as modificações da resistência vascular pulmonar são paralelas às da resistência vascular sistêmica.^9^Nesse sentido, estudos recentes desafiam esse “dogma” e mostram uma tendência a elevação da pressão arterial em mulheres com índice de massa corporal (IMC) > 25 kg/m^2^ e obesas antes da gestação.^2^

O trabalho de parto normal está associado a alterações hemodinâmicas significativas, devido a ansiedade, esforço, dor, contrações uterinas, postura materna (lateral esquerda *versus* supina), involução uterina e sangramento. Durante o trabalho de parto, o sangue dos sinusoides uterinos é lançado para a circulação sistêmica a cada contração, aumentando a pré-carga em cerca de 500 ml de sangue, o que determina aumento do débito cardíaco e da pressão arterial. Assim, no segundo estágio do parto, o débito cardíaco está em torno de 50% mais elevado em relação ao pré-parto, e na expulsão fetal, 60 a 80% acima dos níveis pré-gestacionais. A brusca mudança do débito cardíaco é transitória, permanece elevada no puerpério imediato e não é acompanhada por variações da pressão arterial. No parto normal, perde-se em torno de 400 ml de sangue, podendo ser maior na cesariana, em torno de 800 ml. Após o período expulsivo, ocorre aumento súbito do retorno venoso, que se deve à autotransfusão do plexo uterino, à descompressão do fluxo da veia cava inferior e à redução da capacidade do sistema venoso. Além disso, a resistência vascular periférica está aumentada pela contração sustentada do útero, ocluindo os vasos que abrem na superfície materna da placenta. A autotransfusão contínua que ocorre durante 24 a 72 horas após o parto representa alto risco de congestão pulmonar na mulher cardiopata.^10^

Os efeitos cardiovasculares durante o parto também são influenciados pela eventual ocorrência de infecção, hemorragia e administração de anestésicos ou analgésicos.^11^

De modo geral, os padrões de alteração do volume sanguíneo materno durante o trabalho de parto, o período expulsivo e o puerpério obedecem às seguintes fases:

1. Hemoconcentração durante o trabalho de parto, variável com o grau de atividade uterina e de desidratação materna;
2. Redução do volume sanguíneo durante e imediatamente após o parto proporcionalmente à quantidade de sangue perdida;
3. Elevação imediata e transitória do volume sanguíneo após a dequitação placentária atribuída ao influxo de líquido para o espaço intravascular, devido ao esvaziamento uterino;
4. Discreta elevação do volume sanguíneo entre o segundo e terceiro dias do pós-parto, secundária ao aumento transitório da secreção de aldosterona;
5. Redução do volume plasmático após uma semana do parto, de modo que o volume sistólico materno pode apresentar uma discreta queda nesse período, normalizando-se em curto prazo.

#### 2.1.2. Modificações da Coagulação Sanguínea

**Figura 3:**
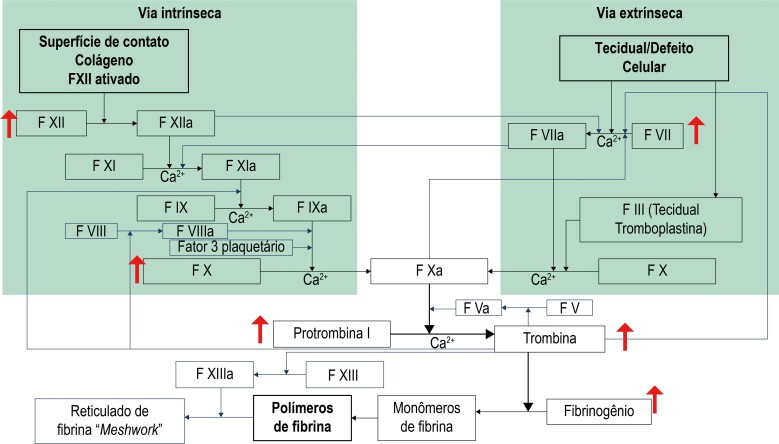
**–** Ativação dos fatores de coagulação durante a gravidez. F: fator. Adaptada e traduzida de Bremme et al., 2003.^12^

Durante a gravidez, ocorre uma ativação da síntese dos fatores de coagulação II, VII, VIII, IX e X, bem como do fibrinogênio, além da redução dos anticoagulantes endógenos (sobretudo da antitrombina e da proteína S), todos determinantes do estado de hipercoagulabilidade, peculiar a uma gravidez saudável.^12^ Essas modificações ocorrem progressivamente após o primeiro trimestre da gestação, com encurtamento dos tempos de protrombina, de tromboplastina parcial e de trombina, favorecendo a fragilização da função anticoagulante.^13^ Somando esses mecanismos à compressão mecânica do plexo venoso para os membros inferiores pelo útero gravídico, justifica-se a predisposição característica da gravidez ao tromboembolismo (Figura 3).

#### 2.1.3. Modificações Respiratórias (Figura 4)

**Figura 4:**
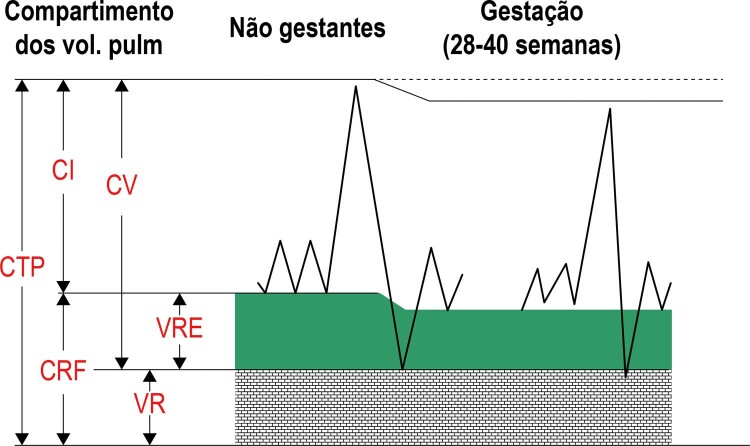
**–** Modificações respiratórias da gravidez. CI: capacidade inspiratória; CRF: capacidade residual funcional; CTP: capacidade total pulmonar; CV: capacidade vital; VR: volume residual; VRE: volume reserva expiratório. Adaptada e traduzida de Hegewald e Crapo, 2011.^*16*^

O consumo de oxigênio aumenta em torno de 50%, especialmente nos dois últimos trimestres da gestação, e não é proporcional ao ganho de peso materno. O ganho ponderal durante a gestação inclui não somente a atividade metabólica do feto, mas também o peso do líquido amniótico e o aumento de fluido nos tecidos maternos, ambos considerados metabolicamente inertes. No trabalho de parto, o consumo de oxigênio eleva-se de 250 para 750 ml/min em cada contração.^14^

O trato respiratório normal é modificado durante a gravidez, o que induz à alcalose respiratória, com maior pressão arterial de oxigênio (PaO_2_) e menor de gás carbônico (PaCO_2_) ,quando comparado ao estado não gravídico. A menor PaCO_2_ favorece um gradiente de difusão que facilita a capacidade de o feto eliminar produtos do seu metabolismo aeróbico.^3^

O aumento da ventilação-minuto é acompanhado do incremento do volume corrente sem modificação da frequência respiratória. A hiperventilação materna é considerada um mecanismo protetor para o feto contra os efeitos prejudiciais de uma excessiva concentração de CO_2_ tecidual, ao mesmo tempo em que ocorre elevação da PaO_2_ para 100 mmHg.

Modificações da caixa torácica ocorrem com o aumento uterino e a elevação do diafragma. A circunferência torácica aumenta em torno de 5 a 7 cm, o ângulo subesternal alarga-se, e o diâmetro vertical diminui. Essas modificações são acompanhadas por alterações na distribuição do ar nos diversos compartimentos pulmonares.

O exame histológico da mucosa do trato respiratório superior durante a gravidez revela: hiperemia, hiperatividade glandular, aumento da atividade fagocítica e incremento do conteúdo de mucopolissacarídeo. A congestão nasal e a epistaxe, frequentes na gestação, possivelmente são causadas por essas alterações.^15^ A função das vias aéreas e respiratórias é preservada durante a gravidez, como refletido por um volume expiratório forçado inalterado no primeiro segundo (FEV1) e uma relação inalterada entre FEV1 e capacidade vital forçada (CVF).^15^

A redução de 25% da capacidade residual funcional (CRF) dos pulmões associa-se ao aumento similar da capacidade inspiratória (CI). Consequentemente, a capacidade vital (CV) não apresenta modificação durante a gravidez.

A diminuição da CRF para 300 ml durante a gravidez não é acompanhada do aumento da resistência das vias respiratórias, que, ao contrário, sofre uma redução importante, talvez por relaxamento da tonicidade da musculatura lisa secundário à ação hormonal. Essa redução serve para diminuir o trabalho da respiração.

Com a hiperventilação, ocorre um aumento na PaO_2_ e um desvio da curva de dissociação da hemoglobina para a direita. A gasometria normal em uma mulher grávida deve ter um pH entre 7,40 e 7,47, uma PaCO_2_ entre 30 e 32 e um aumento discreto da PaO_2_. A alcalose respiratória é compensada parcialmente pelo aumento da excreção renal de bicarbonato, o que mantém os níveis séricos de HCO_3_ entre 18 e 21 meq/L (déficit de base 3 a 4 meq/L). A diminuição na CRF pulmonar e o aumento no consumo de oxigênio reduzem a reserva de oxigênio materno, o que, em uma insuficiência respiratória, representa um estado de alerta para a adoção de medidas precoces de suporte respiratório ou ventilatório, a fim de não haver comprometimento fetal ou materno.^16^

O mecanismo da dispneia durante a gravidez normal não é totalmente claro. A hiperventilação induzida pela progesterona provavelmente é, pelo menos, parcialmente responsável, talvez devido à elevação da ventilação acima do nível necessário para atender ao aumento da demanda metabólica.

#### 2.1.4. Mudanças na Parede Vascular

Mudanças hormonais da gravidez podem alterar a estrutura da parede vascular, resultando em fragilização da parede das artérias. O estrógeno influencia na deposição anormal de colágeno no interior da camada média das grandes e médias artérias. A elastase circulante pode provocar a ruptura da lâmina elástica e o enfraquecimento da média da parede dos vasos. Além disso, a relaxina, hormônio fator de crescimento *insulina-like* (detectado no plasma), causa redução da síntese de colágeno e predispõe à dissecção arterial.^17^

#### 2.1.5. Pontos-chaves

O conhecimento das modificações fisiológicas do ciclo gravídico-puerperal é fundamental na conduta durante a gestação e na estratificação de risco de mulheres portadoras de cardiopatias.

## 2.2. Avaliação Materna e Fetal

### 2.2.1. Avaliação Clínica Materna

***2.2.1.1. Anamnese e Exame Físico***

A investigação clínica inicial da gestante cardiopata exige o questionamento sobre a história familiar no que diz respeito a doenças cardíacas genéticas transmissíveis. Destaca-se o histórico familiar de morte súbita prematura, cardiomiopatias, cardiopatias congênitas (CC), síndrome de Marfan, síndrome do QT longo, taquicardia ventricular (TV) catecolaminérgica e síndrome de Brugada.

As modificações fisiológicas da gravidez influenciam na avaliação do estado cardiovascular, e a interpretação entre ser ou não saudável exige um conhecimento especializado (Tabela 2).

**Tabela 2:** – Avaliação clínica da gestante normal

Sintomas	Sinais
Diminiuição da capacidade física ao exercício	Hiperventilação
Dispneia	Edema de membros
Cansaço	Distenção das veias do pescoço
Palpitação	Estertores de bases pulmonares
Tonturas	Ictus cordis desviado para a esquerda
Ortopneia	Impulso do ventrículo direito palpável
Inchaço nas pernas	Impulso do tronco de pulmonar

*Adaptada de Davies et al., 2007.^19^*

As queixas de falta de ar (hiperventilação), fadiga fácil, diminuição da capacidade funcional ao exercício e estertores basais que desaparecem com tosse ou respiração profunda são sintomas que surgem com o crescimento uterino e seu efeito mecânico na compressão diafragmática, especialmente no final da gestação. Além disso, o edema periférico e as veias varicosas são frequentes nos estágios mais tardios da gestação. O pulso arterial sistêmico é caracterizado por um rápido aumento e um rápido colapso (“pequeno golpe de aríete”) a partir do primeiro trimestre.

Na palpação do tórax, nota-se que o “ictus” cardíaco é desviado para a esquerda, anterior, e girado em direção a uma posição transversal à medida que o útero aumenta. Como resultado, o impulso apical é deslocado para o quarto espaço intercostal e lateralmente para a linha hemiclavicular. O impulso ventricular esquerdo é relativamente hiperdinâmico, mas não sustentado; o ventrículo direito pode ser palpável porque, como o ventrículo esquerdo, ele suporta um volume maior de sangue, que é ejetado contra uma resistência relativamente baixa. À medida que a gravidez avança, o aumento das mamas e do abdômen dificulta, e às vezes impossibilita, a palpação adequada do coração.^18^

As alterações auscultatórias que acompanham a gestação normal começam no final do primeiro trimestre e geralmente desaparecem dentro de uma semana após o parto. Frequência cardíaca basal mais alta, sons cardíacos mais altos no precórdio, desdobramento da primeira e da segunda bulha no terceiro trimestre e sopros de ejeção sistólica (até grau 2/6) sobre as áreas pulmonar e tricúspide são regularmente detectados na ausculta cardíaca. A terceira bulha pode estar presente na maioria das mulheres grávidas; o quarto som do coração é raramente ouvido e, em geral, é patológico. O “hum” venoso é quase universal em mulheres saudáveis durante a gestação normal, mais audível sobre a borda esternal direita superior, atribuível ao aumento do retorno venoso. O sopro mamário (sistólico ou contínuo) é audível sobre o torax anterior no final da gestação e é peculiar à gravidez, decorrente do aumento do fluxo sanguíneo mamário. É especialmente comum após o parto em mulheres lactantes.^20^

Sopros diastólicos são incomuns em gestações normais. Quando ocorrem, podem refletir aumento do fluxo através da valva tricúspide ou mitral, ou dilatação fisiológica da artéria pulmonar. No entanto, esses sopros podem representar uma condição patológica, necessitando de investigação com exames subsidiários.^20^

O estado hiperdinâmico da gravidez pode manifestar-se com episódios de taquicardia, e a frequência basal de repouso pode oscilar em torno de 90 bpm. Bradicardias são raras; quando ocorrem, torna-se necessária uma investigação mais detalhada. O ritmo sinusal deve ser prevalente entre as grávidas, mas é muito comum a presença de extrassístoles supraventriculares ou ventriculares.

Na aferição da pressão arterial em grávidas, é aceito como diagnóstico da pressão diastólica o quarto ruído de Korotkoff, a partir do qual os sons começam a se modificar, o que às vezes não é facilmente reprodutível. Por isso, é fundamental aferir a pressão arterial em decúbito lateral esquerdo usando um método padronizado. A hipotensão arterial é um achado comum no primeiro trimestre, mantida até 22 a 24 semanas, com retorno da pressão arterial ao nível pré-gravidez quando próximo ao termo da gestação.

***2.2.1.2. Pontos-chaves***

- Anamnese detalhada considerando os sintomas atuais e pregressos;
- História familiar;
- Exame fisico detalhado para diferenciar entre o normal e a doença cardíaca.

### 2.2.2. Avaliação Obstétrica e Fetal

**Figura 5:**
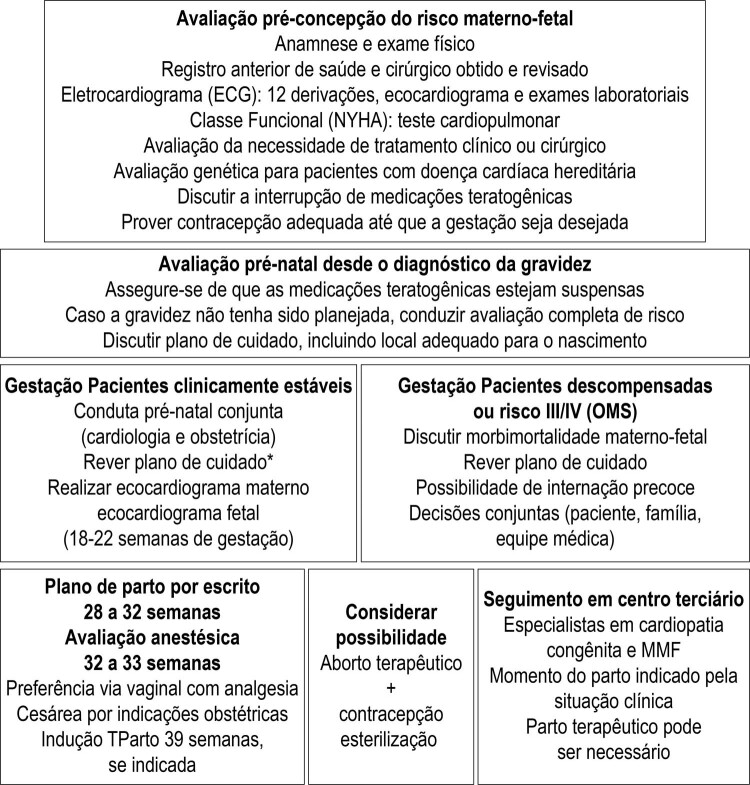
**–** Avaliação e conduta das mulheres com doenças cardiovasculares. ECG: eletrocardiograma; OMS: Organização Mundial da Saúde; NYHA: New York Heart Association; MMF: Medicina Materno-fetal.

As complicações obstétricas e perinatais são significativamente maiores em mulheres portadoras de cardiopatias, fato que resulta na primeira causa de morte materna durante o ciclo gravídico-puerperal. A carência de protocolos no atendimento da gestante cardiopata e a frágil interação multidisciplinar contribuem para o mau desfecho da gravidez. Nesse cenário, é necessária a elaboração de protocolos de atendimento alinhados na prevenção e no tratamento das complicações durante a gravidez, o parto e o puerpério da gestante cardiopata. O Serviço de Cardiopatia e Gravidez do Departamento de Obstetrícia da Universidaddse Federal de São Paulo propõe neste documento um protocolo apresentado na Figura 5.

O plano de cuidado inclui: preparação e prontidão para o parto, em hospital de referência; obediência à rotina para pacientes anticoaguladas e em trabalho de parto prematuro; prevenção da hemorragia pós-parto (HPP); e a profilaxia de endocardite infecciosa.

Os principais fatores maternos que comprometem o crescimento e desenvolvimento fetais são: baixo débito cardíaco (insuficiência cardíaca [IC] e lesões cardíacas obstrutivas), hipoxemia (hipertensão pulmonar [HP], cardiopatias cianogênicas), uso de medicamentos (anticoagulantes, betabloqueadores, diuréticos, antiarrítmicos), hereditariedade (transmissão genética), infecções maternas (por *Trypanosoma cruzi* [*T. cruzi*]) e complicações obstétricas (Tabela 3).

**Tabela 3:** – Preditores de eventos neonatais em gestantes portadoras de cardiopatias

Classe funcional III/IV (NYHA)
Cianose
Lesões cardíacas obstrutivas
Tabagismo
Hipoxemia – saturação de oxigênio < 90%
Necessidade de anticoagulação permanente
Dopplerfluxometria uteroplacentária anormal
Infecções maternas (por Trypanosoma cruzi, vírus da imunodeficiência humana e toxoplasmose)
Pais portadores de cardiopatias congênitas
Uso de fármacos durante a gravidez (IECA, BRA, betabloqueadores)
Complicações obstétricas – hipertensão arterial, diabetes gestacional

*BRA: bloqueadores dos receptores da angiotensina; IECA: inibidores da enzima de conversão da angiotensina; NYHA: New York Heart Association.*

As consequências para o concepto incluem a maior frequência de prematuridade, de recém-nascidos com crescimento intrauterino restrito (RCIU), de abortamentos, de anomalias cardíacas e não cardíacas e de morte. Complicações clínicas maternas que se associam ao baixo débito cardíaco causam maior frequência de baixo peso, índice de Apgar inferior a 7 e peso médio menor em 300 g, comparadas àquelas cujas gestações evoluíram sem complicações.^21^

A hipoxemia materna em portadoras de cardiopatias cianogênicas aumenta o risco fetal, mesmo existindo mecanismo de compensação para facilitar o recebimento de oxigênio pelo feto. A maioria dos recém-nascidos de mães hipoxêmicas é pequena para a idade gestacional e prematura. Observa-se também uma elevada frequência de abortos, proporcional aos valores elevados de hematócrito e hemoglobina materna.

O uso de anticoagulantes durante a gravidez causa perdas fetais expressivas. Estima-se que a incidência de abortos espontâneos no primeiro trimestre seja de 28,6 *versus* 9,2% para gestantes em uso de varfarina *versus* heparina, respectivamente.^22^ A varfarina sódica, quando usada no primeiro trimestre, causa em 5 a 10% a síndrome da varfarina fetal, que ocorre entre a sexta e a nona semana de gestação^23^ (Tabela 4). A incidência é variável porque, muitas vezes, a síndrome sob a visão clínica pode não ser identificada; porém, na avaliação de geneticistas, sua frequência é muito maior. O risco da síndrome varfarínica, quando comparado com o da população em geral, é de *OR* 3,86, (1,86 - 8,00 – IC 95%). Por isso, essas crianças devem ter uma avaliação genética detalhada na primeira infância e ser acompanhadas no desenvolvimento escolar.

**Tabela 4:** – Síndrome varfarínica fetal23

Acometimento ósseo/cartilagem (condrodisplasia punctata)
Hipoplasia de extremidades (nanismo e distrofia óssea) Defeito óptico: cegueira, atrofia óptica, microftalmia
Sistema nervoso central: retardo mental, surdez
Restrição de crescimento intrauterine
Escoliose
Cardiopatia congênita
Morte

Recém-nascidos de mães que utilizaram amiodarona, sotalol, inibidores da enzima de conversão da angiotensina (IECA), bloqueadores dos receptores da angiotensina (BRA) e outros medicamentos durante a gestação devem ter uma avaliação específica das anormalidades relacionadas a esses fármacos durante o período neonatal. Em pacientes que já foram operadas e sofreram transfusão sanguínea anterior, é essencial a pesquisa de infecções por hepatite B e pelo vírus da imunodeficiência humana (HIV), já que a utilização de medicamentos para essas condições pode diminuir a transmissão vertical.

A avaliação fetal deve ter periodicidade decidida pelo obstetra de acordo com a gravidade dos casos e os parâmetros a serem avaliados. Pacientes graves que incluem classe funcional (CF) III/IV (New York Heart Association – NYHA), doença valvar obstrutiva grave, cardiopatias cianogênicas, CC complexas e HP podem necessitar de reavaliação fetal, por ultrassom, até mesmo semanal. O doppler fetal das artérias uterinas durante o segundo trimestre tem a finalidade de prever pré-eclâmpsia. Ele inclui a avaliação das artérias umbilical, cerebral média e uterina, e da relação cérebro-placentária e ducto venoso (Tabela 5).

**Tabela 5:** – Propedêutica fetal

Ultrassonografia de primeiro trimestre (estabelecer a idade gestacional)
Ultassonografia de segundo trimestre (análise da morfologia fetal)
Doppler de artérias uterinas para predição de pré-eclâmpsia
Doppler fetal a partir de 26 semanas (quinzenal ou semanal nos casos graves)
Ecocardiografia fetal (gestante com cardiopatia congênita): 20 semanas
Ultrassonografia de terceiro trimestre (perfil de crescimento fetal e perfil biofísico fetal) quinzenal a partir de 26 semanas nos casos graves

Na avaliação fetal por ultrassonografia (US), é importante estimar a idade gestacional, a vitalidade, a morfologia, o volume de líquido amniótico e o perfil de crescimento fetal. Se for detectada alguma anormalidade, a avaliação deve ser complementada por exames mais específicos, como doppler fetal, perfil biofísico fetal e ecocardiograma (ECO) fetal.^24^Este último deve ser realizado de rotina e sempre após as 20 semanas, por indicação materna ou fetal. As indicações maternas são: diabetes melito, cardiopatia congênita de um dos pais, infecção materna relacionada à teratogenicidade (rubéola, citomegalovírus, HIV), doença de Chagas e toxoplasmose (relacionadas a miocardiopatias ou miocardites fetais), idade materna > 35 anos, fenilcetonúria, doenças do tecido conjuntivo (mais associadas a bloqueio atrioventricular fetal) e exposição a agentes teratogênicos. A avaliação complementar por indicações fetais são o achado de outras anormalidades em estudo morfológico, doenças cromossômicas e arritmias fetais.

***2.2.2.1. Pontos-chaves***

- A morbimortalidade perinatal é maior em recém-nascidos de mães cardiopatas, quando comparada à população em geral;
- Múltiplos fatores maternos associam-se a maior incidência de perdas fetais, malformações, RCIU e prematuridade;
- Obstetras e neonatologistas devem atentar-se para as intercorrências neonatais relacionadas com a cardiopatia materna.

## 2.3. Exames Subsidiários Isentos de Radiação

### 2.3.1. Eletrocardiograma

O eletrocardiograma (ECG) é o primeiro método utilizado para confirmação diagnóstica na prática clínica cardiológica. Os critérios para a sua realização em gestantes seguem aqueles definidos para a população geral; contudo, ele não deve fazer parte da rotina pré-natal para triagem de doença cardíaca. O ECG deve servir para avaliação e acompanhamento de gestantes com uma cardiopatia prévia e para investigação de arritmias.^25^

As alterações fisiológicas da gestação devem ser consideradas na interpretação do registro eletrocardiográfico, destacando-se: discreto desvio do eixo elétrico para a esquerda; inversão da onda T nas derivações DIII, V1, V2 e, às vezes, V3; onda “q” proeminente nas paredes inferior e anterolateral; e aumento da duração da onda P e do intervalo QT.^26^Medidas de duração da onda P e do intervalo QT nos três trimestres da gravidez mostraram prolongamento da duração da onda P no segundo trimestre e do intervalo QT máximo no termo da gestação.^27^

A depressão do segmento ST pode ser observada em 25 a 47% das gestantes durante o parto cesáreo ou 30 minutos depois, independentemente do tipo de anestesia utilizado. Nenhuma alteração sugestiva de isquemia foi observada durante o parto vaginal em gestantes normais.^26,27^

### 2.3.2. Ecocardiograma

É o exame de escolha para investigação diagnóstica da maioria das cardiopatias, pela facilidade de uso, ausência de risco materno-fetal e pelo baixo custo, quando comparado a outros métodos. As indicações seguem as da população geral,^28^ seja para diagnóstico inicial, quando há suspeita de cardiopatia, para estratificação de risco pela medida da fração de ejeção ou do *Strain* global longitudinal, ou ainda, para determinação da conduta terapêutica clínica ou de intervenção percutânea ou cirúrgica, nas estenoses importantes das valvas mitral e aórtica.

A volemia aumentada da gestação pode determinar dilatação discreta das câmaras cardíacas (até 20% das câmaras direitas e 10 a 12% das esquerdas), refluxo discreto das valvas mitral e tricúspide, aparecimento de mínimos gradientes fisiológicos através das valvas e aumento de gradientes prévios, à gravidez, como nas lesões obstrutivas do coração.^29^

O ECO transesofágico é relativamente seguro e tem suas indicações convencionais,^29^ destacando-se que o risco de vômito e aspiração é aumentado principalmente após 20 semanas de gestação. Essa realidade exige a presença do anestesista, que auxilia na seleção da sedação mais adequada e no controle da ventilação materna e do monitoramento fetal durante o procedimento.

Nos períodos finais da gestação, pode ser notada a presença de pequeno derrame pericárdico consequente à retenção hídrica e salina excessiva, que desaparece no puerpério. Esses derrames não tem significado patológico e geralmente são assintomáticos; contudo, merecem uma reavaliação seis semanas após o parto.

O ECO fetal pode ser utilizado para a detecção de cardiopatia congênita e pode ser realizado a partir da 12ª semana de gestação, por via transvaginal, e da 18ª semana por via abdominal. Embora a principal indicação da ecocardiografia fetal seja a presença de alteração no exame de ultrassom de rotina, algumas indicações maternas são importantes para a realização do estudo, dentre as quais diabetes melito pré-gestacional ou identificada no primeiro trimestre de gestação, fenilcetonúria, lúpus eritematoso sistêmico (LES) e síndrome de Sjögren com anticorpos lúpicos positivos.^30^ Nesses casos, a presença de bloqueio atrioventricular total (BAVT) fetal em uma gestação anterior ou de LES neonatal aumenta a possibilidade de comprometimento fetal na próxima gestação, ou, às vezes, a alteração do ritmo cardíaco fetal determina a indicação de melhor avaliação materna para a pesquisa de auto-anticorpos.

Outras indicações são os casos de reprodução assistida, doença cardíaca congênita materna ou infecções, tais como a rubéola no primeiro trimestre da gestação ou outras viroses, quando existe a suspeita de miocardite ou pericardite fetal associada. Indicações menos precisas se referem ao uso materno de medicamentos no primeiro trimestre da gestação, como anticonvulsivantes, lítio, IECA, ácido retinóico, vitamina A, paroxetina e anti-inflamatórios não hormonais, seja pelos riscos de malformação fetal ou constricção ductal.^31^

### 2.3.3. Monitoramento Ambulatorial da Pressão Arterial

Monitoramento ambulatorial da pressão arterial (MAPA) é considerado um exame seguro, cuja principal indicação é a identificação da hipertensão arterial precoce, que ocorre nas primeiras 20 semanas da gestação. Estima-se que aproximadamente um terço das gestantes apresenta hipertensão do avental branco (HAB), e quase a metade delas pode desenvolver a hipertensão arterial verdadeira, com necessidade de tratamento.^32^

O monitoramento da pressão arterial em diferentes trimestres mostra resultados conflitantes em relação ao comportamento pressórico, tendo pouca utilidade para identificar gestantes que desenvolvem hipertensão tardia ou mesmo para predizer eventos adversos em pacientes hipertensas. Os valores de referência são os mesmos utilizados para a população geral, e não há estudos que sugiram o uso de rotina da MAPA para o diagnóstico ou o monitoramento da pressão arterial em substituição da medida convencional com tensiômetro.^33^

### 2.3.4. Sistema Holter-24 Horas

O sistema Holter é utilizado principalmente para a detecção ou estratificação de arritmias durante a gestação. As principais indicações são: investigação de palpitação, síncope ou pré-síncope sem causa aparente, ou, menos frequentemente, investigação de eventos neurológicos nos quais a fibrilação atrial (FA) possa estar envolvida na etiologia.^34^

O Holter é o modelo de exame para identificar e caracterizar as arritmias em simples ou complexas, sintomáticas ou assintomáticas, que são informações fundamentais para a conduta durante a gravidez. Destaca-se a sua indicação na investigação de FA paroxística, outras taquiarritmias, bradiarritmias sinusais sintomáticas e bloqueio atrioventricular de diferentes graus. O Holter tem também grande valor na avaliação de portadoras de marca-passo ou cardioversor desfibrilador implantável (CDI), na ocorrência de sintomas como palpitação, síncope ou pré-síncope, ou quando existe suspeita de falha de comando do dispositivo.

### 2.3.5. Teste Ergométrico

A principal indicação do teste ergométrico durante a gestação é a investigação de doença isquêmica coronariana. A realização de teste submáximo, alcançando 80% da frequência cardíaca máxima prevista, parece ser um método seguro durante a gestação, mas a carência de estudos não possibilita validar a sua indicação para definir doença isquêmica. Por isso, não há recomendação de realização do teste ergométrico durante a gestação para investigação de doença isquêmica coronária. Da mesma maneira, a utilização do estresse com dobutamina deve ser contraindicada durante a gravidez.

Em contrapartida, antes da concepção, a resposta cronotrópica anormal identificada no teste ergométrico em pacientes cardiopatas parece ser uma informação preditiva de eventos adversos na futura gravidez. Na mesma linha de investigação, o teste ergoespirométrico é válido para a avaliação da reserva miocárdica, particularmente em portadora de CC.^35^

### 2.3.6. Pontos-chaves

- O ECG e o ECO devem ser indicados na suspeita de doença cardíaca;
- O ECO fetal é indicado nas CC ou quando há suposição de comprometimento fetal decorrente de doença materna;
- O Sistema Holter de 24 horas auxilia na identificação e estratificação das arritmias cardíacas;
- A principal indicação da MAPA é a identificação da hipertensão arterial “precoce”, que ocorre nas primeiras 20 semanas da gestação;
- O teste ergométrico não é indicado na investigação de doença isquêmica coronária durante a gestação;
- O teste ergoespirométrico auxilia na estratificação de risco em portadoras de CC no planejamento da gestação.

## 2.4. Exames de Imagem com Radiação

A utilização de exames por imagens radiológicas em adultos cardiopatas corresponde a 12% da totalidade dos exames a que esses pacientes são expostos^36^ e a 40% da dose total de radiação que receberão durante toda a vida.^37^ Assim, causa preocupação a realização de exames que emitem radiação durante a gravidez e lactação.

As medidas de radiação ionizante podem ser em sieverts (Sv), que expressa a dose equivalente de radiação no tecido, ou em gray (Gy), indicando a dose total da radiação. O Sv é a medida de maior significado biológico.^38^

Existem dois efeitos biológicos da radiação: o determinístico, que leva à morte celular quando se excede a dose máxima recomendada de radiação, tornando-se evidente após alguns dias, semanas ou meses do procedimento (catarata, leucopenia, anemia, esterilidade e outros); e o estocástico, que causa transformação celular com alteração aleatória no DNA (ácido desoxirribonucleico) de uma única célula, que continua a se reproduzir. Quando o dano ocorre em célula germinativa, efeitos genéticos ou hereditários podem ocorrer. Não apresenta limiar de dose, podendo o dano ser causado por uma dose mínima de radiação. Além disso, os efeitos são difíceis de serem medidos experimentalmente, devido ao longo período de latência. Dentre os principais exemplos estão o câncer (leucemia de 5 a 7 anos, tumores sólidos de 10 a 15 anos ou mais) e os efeitos genéticos. Verifica-se que o risco estocástico é máximo em crianças e maior em mulheres quando comparadas aos homens, sendo reduzido em 50% entre os idosos octagenários.^39^

Durante a gravidez, os efeitos biológicos da radiação no embrião dependem da dose e da idade gestacional, podendo ser divididos em quatro categorias: óbito intrauterino, malformações, distúrbios do crescimento e desenvolvimento, e efeitos mutagênicos e carcinogênicos.^40,41^

Admite-se que o risco não carcinogênico, que inclui aborto e malformação, é insignificante nas doses inferiores a 50 mGy, comparado a outros riscos da gravidez. Em contrapartida, estima-se^42^ que doses superiores a 100 mGy apresentam potenciais efeitos sobre o feto/embrião de acordo com a idade gestacional, tal como: morte fetal quando a exposição ocorre entre a primeira e a segunda semana de gestação; graves anormalidades no sistema nervoso central (hidrocefalia, microcefalia, retardo mental) entre a 3ª e a 15ª semana; retardo mental, microcefalia e restrição de crescimento fetal entre a 16ª e a 30ª semana; após a 32ª semana de gestação, os efeitos teratogênicos são ausentes, mas permanece o risco aumentado de desenvolvimento de neoplasia maligna durante a infância e na idade adulta. A indicação de interrupção da gestação poderia ser considerada em doses de radiação entre 100 e 500 mGy, com base em circunstâncias individuais, como doença maligna materna que requer imagens seriadas durante a gestação, procedimentos intervencionistas ou radioterapia.^43^

Nesse sentido, é importante lembrar que a incidência natural de anomalias congênitas na população em geral varia entre 0,5 e 5%, e que a exposição à dose de 10 mGy de radiação associa-se à probabilidade de malformações, microcefalia e retardo mental da ordem de 0,5, 0,4 e 0,1%, respectivamente.^41^Ainda nessa linha de investigação, estudos demonstram que a exposição uterina mesmo a baixas doses de radiação (20 mGy) aumenta o risco de câncer na infância e de ocorrência de leucemia, por um fator de 1,5 a 2,0, quando comparado à incidência naturaldessas doenças.^43^ Os principais métodos radiológicos e as doses de radiação absorvidas pelo feto, pelo paciente e pelas mamas (durante a lactação) estão expostos na Tabela 6.

**Tabela 6:** – Doses de radiação associadas a exames radiológicos

Modalidade	Fetal dose (mGy)	Materna dose (mSv)	Mamária dose (mGy)
**Tomografia**			
Angiografia pulmonar	0,01 a 0,66	2,7 a 40	8 a 70
Abdômen e pelve	13 a 25	3 a 45	–
Angiografia das aortas torácica e abdominal, com ou sem agente de contraste	6,7 a 56	4 a 68	16 a 130
Angiografia da artéria coronária	0,1 a 3	7 a 39	10 a 90
Tomografia computadorizada simples de abdômen e pelve	10 a 11	3 a 10	–
**Medicina nuclear**			
Cintilografia de perfusão com baixa dose	0,1 a 0,5	0,6 a 1,0	0,1 a 0,3
Cintilografia V/Q	0,1 a 0,8	1,2 a 2,8	0,2 a 0,7
Viabilidade miocárdica ^18^F-FDG PET	6,8 a 8,1	7	–
Perfusão miocárdica com ^99m^Tc-sestamibi	17	11,4 a 14,8	–
Perfusão miocárdica com ^99m^Tc-tetrofosmin	8,45	9,3 a 11,6	–
**Radiografia**			
Mamografia, duas posições	0,001 a 0,01	0,1 a 0,7	3
Radiografia de tórax, duas posições	0,0005 a 0,01	0,06 a 0,29	< 0,4
Radiografia abdominal	0,1 a 0,3	0,01 a 1,1	–

*FDG: fluorodesoxiglicose; PET: tomografia com emissão de pósitrons; V/Q: ventilação/perfusão. Nota: Doses estimadas que variam de acordo com protocolos, radiomarcador e dose, método de cálculo da dose e fatores paciente-dependente (p. ex., peso corporal e percentual do tecido da glândula mamária).*

Deve-se lembrar que não existe exame radiológico único que exponha o feto a doses superiores a 250 mGy, o que pode ocorrer em uma combinação de exames, ou na vigência de um tratamento essencial à mãe.

Fluoroscopia, radiografias, cateterismo cardíaco e radiologia intervencionista, quando não envolvem diretamente o útero ou a exposição direta do abdômen, resultam em doses de radiação pouco significativas ao feto. Nesse sentido, devem-se considerar estratégias^44^ que possam reduzir a radiação, às vezes em torno de 30 a 65%. Dentre elas, destacam-se: uso de protetores de chumbo sobre o abdômen, colimação do feixe de raios X para a área de interesse, utilização de equipamentos permanentemente calibrados e aferidos, preferência por radiografias digitais e redução do tempo de fluoroscopia e do número de imagens adquiridas. Além disso, as ampliações devem ser realizadas com o emprego de menor número de imagens e de exposições.

Nos exames de cintilografia nuclear, a exposição do feto à radiação ionizante provém da radioatividade acumulada no organismo materno e do transporte e difusão do radiofármaco através da placenta.^45^ A cintilografia ventilação/perfusão (V/Q) é a imagem cintilográfica mais frequente com dose materna reduzida, quando comparada à angiotomografia pulmonar (ATCP). Entretanto, esta fornece doses mais baixas quando o feto ainda é pequeno e mais longe do campo de visão ou tórax.

A cintilografia V/Q e a ATCP têm eficácia no diagnóstico da embolia pulmonar durante a gravidez, embora a ATCP demonstre vantagem de identificar outras doenças pulmonares. Considera-se que, na suspeita clínica de embolia pulmonar, a radiografia de tórax simples e a US com doppler de membros inferiores bilateral sejam os exames iniciais para orientar a indicação da cintilografia V/Q, que deve ser preferível à ATCP quando ambas estiverem disponíveis.^45^ Estresse farmacológico com uso de vasodilatadores, tanto com a adenosina como com o dipiridamol, não é recomendado durante a gestação, pelos riscos decorrentes da hipotensão ortostática.

### 2.4.1. Administração de Agentes de Contrastes

Agentes de contraste iodados não apresentam efeitos teratogênicos e podem ser usados na forma oral ou intravenosa, quando a informação do exame é importante para conduta imediata; de outra maneira, seu uso deve ser adiado para após o parto.^46^ Isso porque a maturação da tireoide fetal se dá a partir de 12 semanas e está em mínimo funcionamento na 20ª semana de gestação. Assim, a preocupação é o agente de contraste iodado induzir ao desenvolvimento de hipotireoidismo, embora, nas últimas três décadas, não haja registro de ocorrências nessa situação. Em casos de reação alérgica ao contraste, a difenilefedrina e os corticosteroides podem ser utilizados com segurança. Nas situações de prevenção, a prednisona e a dexametasona devem ser consideradas, porque a maioria desses agentes é metabolizada na placenta antes de alcançar o feto. Contudo, há relatos de casos de supressão adrenal do feto com o uso de corticosteroides, e a metilprednisona foi associada a lábio leporino quando usada antes de 10 semanas de gestação.^43^

Mais recentemente, têm sido utilizados tomógrafos com múltiplas fileiras de detectores (*multislice*), proporcionando vantagens indiscutíveis, principalmente relacionadas a rapidez e definição em estudos abdominais e angiográficos (angio-TC). Todavia, esses benefícios têm sido acompanhados de aumento significativo das doses de radiação absorvidas em órgãos abdominais, em torno de 90 a 180%, quando comparados aos equipamentos helicoidais com uma única linha de detectores. Ao mesmo tempo que a tecnologia *multislice* se consolida como ferramenta extremamente útil em estudos tóracoabdominais, faz-se necessário um investimento na otimização e no ajuste de protocolos que visem ao controle e à limitação da dose de radiação emitida, principalmente durante a gestação.

### 2.4.2. Ressonância Magnética Nuclear

A ressonância magnética cardíaca (RMC) é aconselhável quando outros métodos de diagnóstico não invasivos são insuficientes para definir o diagnóstico, sendo preferível aos exames de imagem que emitem radiação ionizante. A exposição durante o primeiro trimestre da gestação não foi associada a efeitos nocivos ao feto ou à criança na primeira infância.

As evidências considerando o uso de contraste gadolínio durante a gravidez são controversas. O gadolínio (Gd+3) é um íon metálico paramagnético que, no organismo, tem comportamento farmacológico semelhante ao meio de contraste iodado, ou seja, atua como um agente extracelular, difundindo-se rapidamente do compartimento intravascular para o espaço intersticial. Não foi documentada a ocorrência de quaisquer efeitos na mutação ou teratogênicos após a administração inadvertida de meios de contraste baseados em gadolínio durante a gravidez. Contudo, dependendo da dose, seu uso parece estar associado a maiores riscos de manifestações cutâneas dos tipos reumático, inflamatório e infiltrativo, além de perdas fetais.^47^

O íon de gadolínio na sua forma livre é neurotóxico; no entanto, sua ligação a um agente quelante forma um complexo estável, protegendo o organismo contra os efeitos adversos. Os quelatos de gadolínio atravessam a barreira placentária e podem acumular-se na cavidade amniótica; contudo, alguns estudos demonstraram que apenas 0,01% da dose está presente na circulação fetal 4 horas após a administração do contraste, e que apenas vestígios são detectados após 24 horas.

Durante a lactação, ambos os agentes de contraste iodados e o gadolínio têm baixa solubilidade lipídica, e sua concentração no leite materno é inferior a 1 e 0,04%, respectivamente.^46^ Por isso, a Academia Americana de Pediatria e a OMS recomendam não suspender a lactação.

A obtenção do consentimento informado e o esclarecimento às pacientes sobre os riscos inerentes dos exames necessários à conduta médica são medidas essenciais que devem fazer parte da decisão interdisciplinar na indicação do exame com radiação durante a gestação, o que inclui o obstetra e a equipe de radiologia.

### 2.4.3. Pontos-chaves

- A indicação de exame radiológico deve considerar o real benefício para a determinação da conduta terapêutica durante a gestação e na impossibilidade de substituição por método alternativo sem radiação (US, ECO e ressonância magnética);
- O médico radiologista é o profissional mais preparado para avaliar a melhor opção diagnóstica em determinada situação clínica, garantindo segurança à gestante e ao feto;
- Os exames radiológicos devem ser feitos em instituições que possam garantir a adoção de medidas efetivas de proteção radiológica e possuam equipamentos modernos e regularmente calibrados e aferidos;
- A RMC é um exame complementar para definição do diagnóstico de cardiopatia. É seguro durante a gestação, contudo o uso de gadolínio deve ser evitado;
- A necessidade de exame com radiação demanda uma discussão interdisciplinar que envolve radiologista, cardiologista e obstetra, além do consentimento informado da paciente.

## 2.5. Fármacos de Ação Cardiovascular na Gravidez e no Aleitamento

A necessidade de terapêutica farmacológica é muito frequente durante a gravidez e a lactação.^48^Estima-se que 34% das gestantes cardiopatas utilizam medicamentos cardiovasculares de acordo com a seguinte distribuição: betabloqueadores (22%), antiplaquetários (8%), diuréticos (7%), IECA (2,8%) e estatinas (0,5%).^49^ Nessa casuística, a prevalência de eventos adversos ao feto, particularmente o RCIU, foi duas vezes maior quando comparado às gestantes que não utilizaram medicação.^50^

Presume-se que 10 a 15% das mulheres cardiopatas apresentam complicações cardíacas que exigem tratamento farmacológico durante a gestação como na doença hipertensiva gestacional, nas arritmias cardíacas, na IC e no tromboembolismo.^51,52^Entretanto, a prescrição de medicamentos durante a gravidez exige conhecimentos básicos sobre a farmacocinética e a classificação dos fármacos quanto à segurança materno-fetal durante a gestação e na lactação.

A farmacocinética dos medicamentos é influenciada pelas modificações fisiológicas da gravidez, acarretando, muitas vezes, uma redução da concentração plasmática dos fármacos de tal maneira que eventuais ajustes nas doses devem ser cogitados para se obter a eficácia terapêutica.^53^ A Tabela 7 resume os aspectos^54^ que merecem as seguintes considerações:

**Tabela 7:** – Farmacocinética durante a gravidez

Diminuição da absorção	Retardo da motilidade intestinal,
**Aumento do volume de distribuição**	Redução do pico de concentração dos fármacos hidrofílicos e lipofílicos, e variações na meia-vida
**Aumento do metabolismo hepático**	Redução da concentração plasmática de fármacos que têm passagem pelo fígado
**Aumento do fluxo renal**	Redução da concentração plasmática de fármacos com excreção renal. Função tubular absorção/excreção é variável

*Adaptada de Feghali et al., 2015.^54^*

- A absorção dos fármacos administrados por via oral é reduzida devido ao retardo da motilidade intestinal.^55^ Além disso, o uso de antiácidos e de ferro como suplemento parece induzir a quelação de medicamentos em meio de pH gástrico aumentado, provocando redução na biodisponibilidade do fármaco;^56^
- O volume de distribuição dos fármacos é aumentado durante a gestação decorrente da expansão do volume plasmático contribuindo para redução do pico de concentração do fármaco;^57^
- O metabolismo hepático está acelerado durante a gestação porque a perfusão hepática é maior. Isso significa que a fração do fármaco removido da circulação pelo fígado está aumentada de tal modo que medicamentos como propanolol, nitroglicerina e verapamil são extraídos mais rápido da circulação sistêmica.^54^ Fármacos como a varfarina, que não depende do fluxo, mas da atividade hepática e da fração livre no plasma, não sofrem influências na sua concentração durante a gravidez. Por outro lado, níveis plasmáticos de nifedipina e do metoprolol estão reduzidos na gravidez por conta da maior atividade de catalização enzimática;^58^
- Aumento de 85% do fluxo sanguíneo renal quando comparado aos níveis pré-gestacionais.^59,60^ Contudo, a função tubular é variável, com redução na excreção de ácido úrico e na absorção de glicose, e aumento na excreção de proteína.^61^

Quanto à segurança, a maioria dos estudos de fármacos na gestação é realizada em animais e tem pouca aplicabilidade porque os efeitos são, de modo geral, espécie-específicos. Os estudos em humanos quase sempre são retrospectivos e incluem casuísticas pequenas. As gestantes, salvo raras circunstâncias, são excluídas dos grandes ensaios clínicos. Assim, a literatura médica sobre fármacos na gestação tem, na maioria, questionável evidência científica.

Em 1979, a *Food and Drug Administration* (FDA)^62^introduziu a classificação dos medicamentos de acordo com as categorias de A a X, muito utilizadas na prática diária.^55^Essa classificação rotulava os fármacos de acordo com estudos em animais e mulheres em categorias que variavam desde fármacos que não apresentavam riscos ao feto (categoria A) até aqueles teratogênicos (categoria X).

Em 2015, a classificação (A, B, C, D e X) foi substituída pela denominada *Pregnancy and Lactation Labelling Rule* (PLLR),^63^ que atualmente tem sido mais aceita. Ela fornece um resumo descritivo e informações detalhadas sobre estudos em animais e ensaios clínicos, como exposto na Tabela 8.

**Tabela 8:** – Pregnancy and Lactation Labelling Rule – Food and Drug Administration

Informações obrigatórias
**Referentes à gravidez:** risco de uso da medicação, compatibilidade com a lactação, potencial reprodutivo em homens e mulheres, informações sobre testes de gravidez e uso de anticoncepcionais
**Resumo de risco:** absorção do medicamento por via sistêmica durante a gravidez, dados de estudos em humanos, animais e desfechos fetais adversos rotulados que incluem perdas fetais e malformação
**Contraindicação na gravidez:** anomalia estrutural, embriopatia ou mortalidade fetal e neonatal, prejuízo funcional (toxicidade múltipla de órgãos), alteração de crescimento, retardo ou prematuridade
**Considerações clínicas:** orientações essenciais para prescrição considerando ajustes de dose durante a gravidez e no pós-parto, doenças maternas associadas e/ou risco de embriopatia fetal, reações adversas maternas e fetais e efeitos da medicação no trabalho de parto e no parto
**Dados adicionais:** informações de estudos em animais e humanos que suportam as declarações de risco apresentadas anteriormente
**Registro da exposição durante a gravidez:** informação para profissionais da saúde, com telefone toll free para se obter informação sobre o registro
• Data
• Humano
• Animal

### 2.5.1. Anti-hipertensivos (Tabela 9)

**Tabela 9:** – Efeitos do uso de anti-hipertensivos na gravidez e na lactação

Fármaco	Uso na gravidez	Efeitos materno- fetais	Lactação
IECA e BRA	Não	Disginesia e insuficiência renal	Compatível (captopril, enalapril, losartana)
		Malformação congênita cardiovascular e neurológica	
Amlodipina	Sim	Não teratogênico	Provavelmente compatível
		Dados limitados em humanos	
Atenolol	Não	RCIU	Compatível, mas com cautela (opções mais seguras)
		Bradicardia e hipoglicemia fetal	
Succinato de metoprolol	Sim	Baixo peso ao nascer e RCIU	Compatível, mas com cautela (efeitos do betabloqueador Não recém-nascido)
		Bradicardia e hipoglicemia fetal	
Nifedipina	Sim	Provável baixo risco em qualquer fase da gestação	Compatível
Metildopa	Sim	Provável baixo risco em qualquer fase da gestação	Compatível
Clonidina	Sim	Provável baixo risco em qualquer fase da gestação	Compatível
Verapamil	Sim	Provável baixo risco em qualquer fase da gestação	Compatível
Nitroprussiato de sódio	Sim – risco da exposição fetal aos cianetos	Não foi descrita mal formação congênita Acúmulo de cianeto	Não compatível
Furosemida	Sim	Redução no líquido amniótico	Compatível*
Hidroclorotiazida	Sim	Sem evidência de teratogênese. Risco de hipovolemia	Compatível*
Hidralazina	Sim	Trombocitopenia neonatal e síndrome lúpus “*like*”	Compatível
Espironolactona	Não (ação antiandrogênica)	Sem evidência de teratogênese. Ação antiandrogênica (feminilização de feto masculino)	Não recomendado
Amilorida	Sim	Sem evidência de teratogênese de Risco de hipovolemia	Compatível*

*BRA: bloqueadores dos receptores da angiotensina I; IECA: inibidores da enzima de conversão da angiotensina; RCIU: crescimento intrauterino restrito.*

**Nifedipina:** ação hipotensora e tocolítica; não teratogênica. Pode requerer encurtamento do intervalo das tomadas ou maior dose devido ao metabolismo hepático acelerado, mediado pela CYP3A4. Hipotensão mais acentuada com uso de sulfato de magnésio concomitante.^64-66^

**Alfametildopa (agonista receptor** α**2-adrenérgico):** não é teratogênica, considerada como segura e eficaz no tratamento da doença hipertensiva gestacional com resultados favoráveis nos desfechos primários e secundários tais como controle da pressão arterial, crescimento fetal e prematuridade. Os efeitos maternos adversos tais como, hipotensão postural, síndrome lúpus “*like*”, depressão, congestão nasal, sonolência e toxicidade hepática, foram registrados em 1% dos casos tratados.^67,68^

**Hidralazina:** vasodilatador arteriolar direto, de uso oral ou intravenoso nas emergências hipertensivas. Os efeitos adversos são sintomas maternos de lúpus “*like*” e trombocitopenia fetal.^69^

**Clonidina:** agonista α2 tem efeito hemodinâmico divergente, na redução da resistência vascular versus redução no débito cardíaco, e consequente impacto no crescimento fetal. A suspensão abrupta pode causar hipertensão “rebote”. Não é teratogênica. É disponível por via transdérmica.^70^

**Os diuréticos** são indicados na hipervolemia e na IC; no entanto, a redução do volume plasmático, débito cardíaco e fluxo placentário é a principal restrição ao uso de diuréticos durante a gravidez. Seu uso durante a gravidez não tem sido relacionado a efeitos prejudiciais ao concepto. Furosemida é o mais utilizado, enquanto a hidroclorotiazida tem sido relacionada a menor peso ao nascer, icterícia e trombocitopenia neonatal.^71^

**Betabloqueador:** o atenolol não é recomendado porque seu uso está associado à RCIU e a recém-nascidos de baixo peso.^71^

**Amlodipina:** pode ser considerado tratamento de segunda linha sem referência a teratogênico quando usado no primeiro trimestre da gravidez.^71^

**IECA, BRA, inibidores diretos da renina e antagonistas da aldosterona**: contraindicados na gravidez e não devem ser prescritos em mulheres que desejam engravidar. Esses medicamentos causam disgenesia renal, oligoidrâmnio, insuficiência renal, RCIU, anúria neonatal e morte fetal, particularmente no segundo e terceiro trimestres da gravidez.^72^No entanto, os IECA podem ser usados na lactação. Antagonistas da aldosterona têm efeitos antiandrogênicos no feto masculino e são contraindicados na lactação.^52,73^

### 2.5.2. Antiarrítmicos (Tabela 10)

**Tabela 10:** – Efeitos do uso de antiarrítmicos na gravidez e na lactação

Fármaco	Uso na gravidez	Efeitos materno-fetais	Lactação
Lidocaína	Sim	Não teratogênico, altas doses; são descritas depressão respiratória e acidose fetal	Compatível
Propafenona	Sim	Sem dados no primeiro trimestre, sem complicações nos demais	Provavelmente compatível
Propranolol	Sim	Baixo peso ao nascer e RCIU Bradicardia e hipoglicemia fetal	Compatível, mas com cautela (efeitos do betabloqueador no recém-nascido)
Sotalol	Não	Baixo peso, RCIU, *torsade* de *pointes* quando associado a hipomagnesemia	Não
Amiodarona	Não	Hipo e hipertireoidismo fetal, baixo peso ao nascer, QT longo	Não

*RCIU: crescimento intrauterino restrito.*

**Adenosina:** nucleosídio com meia-vida de segundos. É seguro, mas os efeitos adversos incluem bradiarritmias, dispneia, dor precordial e rubor.^74,75^

**Betabloqueadores:** são os fármacos mais utilizados durante a gestação. Não são teratogênicos. Estudos controlados mostram maior frequência de bradicardia e hipoglicemia neonatal, além de maior risco de prematuridade e de recém-nascidos pequenos para a idade gestacional.^76-78^

**Atenolol:** hidrofílico com eliminação renal, é contraindicado pelo elevado risco de RCIU.^79,80^**Propranolol** é seguro; porém, dependendo da dose, pode ocorrer RCIU, hipoglicemia, policitemia e hiperbilirrubinemia.^81^**Metoprolol** é bem tolerado, com *clearance* alto na segunda metade da gestação. Considera-se que o succinato seja mais seguro do que o tartarato porque as doses são menores e podem ser fracionadas.^82,83^**Sotalol** está associado à *torsades de pointes* devido ao prolongamento do intervalo QT. O Sotalol apresenta-se em concentração maior no leite materno, devendo ser suspenso na lactação. Em casos de manutenção da lactação, o controle eletrocardiográfico deve ser feito na mãe e no neonato. De acordo com as Diretrizes ESC 2018,^52^ o sotalol foi contraindicado na gestação e lactação por conta do risco de morte súbita materno-fetal. A proposta é a substituição por propafenona ou flecainide. Contudo, a restrição ao uso de sotalol durante a gravidez e a lactação ainda é controversa, uma vez que os resultados no controle de arritmias complexas têm sido satisfatórios na prática dos especialistas. Embora não existam estudos adequados, o sotalol é parece ser mais seguro quando comparado à amiodarona.^84-85^

**Amiodarona:** lipofílica, acumula-se no músculo esquelético e no tecido adiposo, com meia-vida de semanas a meses. Os efeitos adversos são disfunção tireoidiana (causando o hipotireoidismo neonatal em 17 a 25% dos casos) e comprometimento do desenvolvimento neurológico. Deve ser contraindicada na gestação.^86-87^

**Lidocaína:** mais estudada como agente anestésico do que como antiarrítmico. Sessenta por cento dela é ligada a proteína plasmática, entrando rapidamente na circulação materna e na placenta. Pode levar à depressão do sistema nervoso central do feto quando usada em altas doses.^73,88^

**Propafenona:** é recomendada para a prevenção de taquicardia supraventricular em pacientes com síndrome de Wolff Parkinson White, na taquicardia atrial e na FA refratária a agentes com bloqueio nodal.^52^

**Procainamida:** associada à síndrome lúpus “like” materna.^89^

### 2.5.3. Fármacos na Insuficiência Cardíaca (Tabela 11)

**Tabela 11:** – Efeitos do tratamento da insuficiência cardíaca na gravidez e na lactação

Fármaco	Uso na gravidez	Efeitos materno-fetais	Lactação
Monitrato de isosorbida	Sim	Cefaleia, hipotensão, não teratogênico	Compatível
Hidralazina	Sim	Trombocitopenia neonatal e síndrome lúpus “like”	Compatível
Carvedilol	Sim	Bradicardia	Compatível
Succinato de metoprolol	Sim	Baixo peso ao nascer e RCIU	Compatível, mas com cautela (efeitos do betabloqueador no recém-nascido)
		Bradicardia e hipoglicemia fetal	
Bisoprolol	Não	Baixo peso ao nascer e RCIU Bradicardia e hipoglicemia fetal	Compatível (efeitos do betabloqueador no recém-nascido)
	Risco/benefício		
Digoxina	Sim	Não teratogênico	Compatível
Dobutamina	Sim	Não teratogênico em animais	Provavelmente compatível
Milrinone	Não	Risco em animais	Provavelmente compatível
	Risco/benefício	Sem evidência em humanos	
Sacubitril/ valsartana	Não	Idem BRA; sacubitril dados inadequados	Não
Ivabradina	Não	Defeitos cardíacos em animais	Não
		RCIU	
		Bradicardia no recém-nascido	

*BRA: bloqueadores dos receptores da angiotensina; RCIU: crescimento intrauterino restrito.*

**Carvedilol:** há carência de estudos. É o betabloqueador cardiosseletivo de primeira escolha. Não é teratogênico e não tem sido associado a RCIU.^90^

**Bisoprolol:** não está associado a maior risco de abortamento espontâneo ou mal formação fetal quando usado no primeiro trimestre da gestação. Contudo, RCIU não pode ser descartada no seu uso prolongado, durante toda a gestação.^52,78^

**Hidralazina:** fármaco que substitui os IECA e BRA.^52^

**Nitratos:** não são usados de rotina e não são teratogênicos. Baixa tolerância materna devido a hipotensão e cefaleia.^52^

**Sacubitril/valsartana**: é contraindicado durante a gravidez e, apesar de não haver estudos sobre excreção no leite humano, também não há recomendação de uso durante a amamentação.

**Ivabradina:** estudos em animais mostram sua associação com mal-formação, bradicardia e alteração no crescimento fetal.

### 2.5.4. Antiplaquetários (Tabela 12)

**Tabela 12:** – Efeitos do uso de antiplaquetários na gravidez e na lactação

Fármaco	Pode ser usado na gravidez	Riscos materno-fetais	Lactação
Aspirina	Sim	Hemorragia	Compatível
Clopidogrel	Sim (benefício maior que o risco)	Hemorragia	Provavelmente compatível
Prasugrel	Não Risco/benefício	Sem evidência em humanos	Provavelmente compatível
Ticagrelor	Não Risco/benefício	Sem evidência em humanos	Provavelmente compatível
Ticlopidina	Não	Trombocitopenia, neutropenia	Não
Tirofiban	Sim (benefício maior que o risco)	Hemorragia	Compatível
Abciximab	Sim (benefício maior que o risco)	Hemorragia	Compatível
Epifibatide	Sim (benefício maior que o risco)	Hemorragia	Compatível

**Aspirina:** em baixas doses, é segura em qualquer fase da gestação.^52,91,92^Não é teratogênico, apresenta risco de sangramento materno-fetal. Deve ser suspenso três dias antes do parto.^52,93,94^

**Clopidogrel**: não há estudos que assegurem o seu uso durante a gravidez. Parece não ser teratogênico. A recomendação do uso do clopidogrel deve ser em casos muito específicos, porque a sua suspensão, obrigatório antes do parto, pode prejudicar o tratamento da doença para a qual está sendo indicado.^93,94^

### 2.5.5. Trombolíticos (Tabela 13)

**Tabela 13:** – Efeitos do uso de trombolíticos na gravidez e na lactação

Fármaco	Pode ser usado na gravidez	Riscos materno-fetais	Lactação
Streptoquinase	Sim (benefício maior que o risco)	Risco de hemorragia	Compatível
Tenecteplase	Sim (benefício maior que o risco)	Risco de hemorragia	Compatível
Alteplase	Sim (benefício maior que o risco)	Risco de hemorragia	Compatível
Uroquinase	Sim	Inibidores de proteinase da placenta inativam uroquinase	Compatível

Não ultrapassam a barreira placentária, mas apresentam risco de hemorragia materna.^52^

### 2.5.6. Anticoagulantes (Tabela 14)

**Tabela 14:** – Efeitos do uso dos anticoagulantes na gravidez e na lactação

Fármaco	Pode ser usado na gravidez	Observações e efeitos materno-fetais	Lactação
Varfarina	Não	Síndrome varfarínica no primeiro trimestre, outras anomalias congênitas e neurológicas nos demais	Compatível
	Risco/benefício		
Heparina	Sim	Trombocitopenia	Compatível
Enoxaparina	Sim		Compatível
Fondaparinux	Sim	Várias revisões sugerem segurança durante a gravidez	Compatível
Apixabana	Não	Baixo risco em animais, evidências não apoiam a segurança em humanos	Não
Dabigatrana	Não	Moderado risco em animais, evidências não apoiam a segurança em humanos	Não
Rivaroxibana	Não	Moderado risco em animais, evidências não apoiam a segurança em humanos	Não

A **varfarina sódica**, quando usada no primeiro trimestre, causa a síndrome da varfarina fetal em 5 a 10% dos casos.^95^ A incidência é variável porque, muitas vezes, a síndrome sob a visão clínica pode ser imperceptível, embora, na avaliação de geneticistas, sua frequência seja muito maior. O risco de aborto espontâneo (menos de 20 semanas de gestação) é quase 30% e o de natimorto (mais de 20 semanas de gestação) é de 10%, ambos causados pela intoxicação varfarínica.^96^ A hemorragia materna do parto em pacientes em uso de varfarina é séria; contudo, mais grave é a hemorragia intracraniana neonatal e suas sequelas.^96,97^ Para pacientes sob uso de anticoagulante oral que entram em trabalho de parto prematuro, a indicação é parto cesárea.

A hipótese de que doses inferiores a 5 mg de varfarina podem causar menor risco de embriopatia^52^ não tem apoio em estudos apropriados para servir de base na orientação da anticoagulação no primeiro trimestre da gestação. Entende-se que a propriedade teratogênica de um fármaco independe da sua dose. De fato, recentes relatos vêm demonstrando a ocorrência da embriopatia mesmo em doses inferiores a 5 mg de varfarina.^98,99^Conclui-se que é de “boa prática” que a dose de varfarina seja adequada em busca da meta terapêutica, controlada pelo índice internacional normalizado (INR) e individualizada para cada situação clínica.

A **heparina** não ultrapassa a barreira placentária. A Tabela 15 apresenta as vantagens da heparina de baixo peso molecular (HBPM) sobre a heparina não fracionada (HNF). Ambas se associam ao risco de 10 a 15% de abortos espontâneos devido à hemorragia placentária.^100^ O uso permanente da HNF durante a gravidez apresenta riscos maternos, como: hemorragia (2%); osteoporose (30%); fraturas espontâneas (2%) e trombocitopenia (5 a 15%).^101^ Entretanto, parece que esses efeitos adversos são menores com o uso da HBPM. O controle da anticoagulação da HNF deve ser diário, de acordo com tempo de tromboplastina parcial ativado (TTPA), com meta de 1,5 a 2 vezes maior que o valor basal. Já o controle da HBPM deve ser semanal, de acordo com os valores terapêuticos entre 0,6 e 1,2 IU/ml do fator antiXa, nas pacientes com próteses valvares mecânicas.

**Tabela 15:** – Comparação entre a heparina não fracionada e a de baixo peso molecular

Particularidades	HNF	HBPM
Peso molecular	12.000 a 14.000	4.000 a 6.000
Ação anticoagulante	Trombina e Xa	Xa
Biodisponibilidade	30%	100%
Meia-vida após aplicação	45 a 60 min	12 h
Absorção após injeção SC	Variável	100%
Trombocitopenia	27%	0%
Monitoramento	TTPA	Fator antiXa
Custo	Baixo	Alto
Periodicidade de controle	Maior	Menor
Controle	1,5 a 2 vezes o valor basal	7 a 12 u/ml

*HBPM: heparina de baixo peso molecular; HNF: heparina não fracionada; SC: subcutânea; TTPA: tempo de tromboplastina parcial ativado. Adaptada de Ginsberg et al., 2003.^100^*

A anticoagulação ainda é um desafio terapêutico. Exige conhecimento do risco de trombose para cada situação clínica e dos efeitos colaterais dos anticoagulantes nos diversos momentos do ciclo gravídico-puerperal. O fato é que, quando existe indicação de anticoagulação, a gravidez não deve influenciar no rigor e nas metas convencionais. A fondaparinux tem se mostrado uma alternativa segura quando as heparinas não são toleradas.^52,102^

**Novos anticoagulantes orais (NOACS):** A carência de dados sobre o uso dos NOACS, não permite o seu uso durante a gestação.

### 2.5.7. Hipolipemiantes (Tabela 16)

**Tabela 16:** – Efeitos do uso de hipolipemiantes na gravidez e na lactação

Fámaco	Uso na gravidez	Efeitos materno-fetais	Lactação
Estatinas	Não	Sem evidências de teratogênese	Não
Fibratos	Não	Teratogênese em animais, sem evidência em humanos	Não
Ezetimibe	Não	Baixo risco em animais, evidências atuais não apoiam o uso na gestação	Não
Alirocumab	Não	Baixo risco em animais, evidências atuais não apoiam o uso na gestação	Não
Colestiramina	Sim	Possível redução na absorção de vitaminas	Provavelmente compatível

A primeira escolha é a colestiramina, considerada a mais segura.^103^ As estatinas não parecem ser teratogênicas. Não está esclarecida sua correlação a anomalias congênitas; contudo, devido à carência de estudos, o seu uso deve ser desaconselhado durante a gestação e deve ser suspenso na pré-concepção.^104-106^ Gemfibrozil, fenofibrate e ezetimibe são considerados com potencial teratogênico.^73^

O tratamento da hipertensão arterial pulmonar (HAP) será discutido no tópico 3.6.3. Os fármacos liberados para o uso durante a gravidez incluem:^73,107^

- Prostaglandinas: epoprostenol, treprostinil, Iloprost;
- Inibidores da fosfodiesterase 5: sildenafil e tadalafil;
- Bloqueadores dos canais de cálcio (BCC): diltiazen e nifedipina;
- Óxido nítrico: via inalatória.

Os fármacos contraindicados durante a gravidez são:^73,107^

- Antagonistas do receptor da endotelina: bosentan, ambrisentam e macitentan;
- Estimuladores da guanilatociclase: riociguat.

### 2.5.8. Pontos-chaves

As recomendações básicas para a prescrição de medicamentos durante a gravidez, quando possível, são:

- Considerar a farmacocinética durante a gravidez antes da prescrição;
- Prescrever quando houver indicação de tratamento e o seu benefício superar o potencial risco;
- Orientar a prescrição pela classificação PLLR;
- Evitar medicação no primeiro trimestre da gestação;
- Usar a menor dose, desde que efetiva, pelo menor tempo e, fracionar a dose diária;
- Usar medicamentos que já sejam amplamente aceitos e seguros na gestação;
- Considerar que fármacos com peso molecular inferior a 1.500 Da atravessam a placenta e alcançam a circulação fetal;
- Orientar a pré-concepção para mulheres que fazem uso permanente de medicamentos;
- Considerar que a prioridade do tratamento é materna, mas os riscos obstétricos e fetais devem ser considerados.

## 2.6. Princípios de Conduta Durante a Gravidez

### 2.6.1. Estilo de Vida

A gravidez é a época ideal para modificar o estilo de vida. Isso porque, enquanto gestante, a mulher está motivada a melhorar hábitos não saudáveis pela preocupação com a saúde do concepto, como cessar o tabagismo e o etilismo, consumir dieta mais equilibrada ou controlar o peso.

O tabagismo está relacionado a complicações como placenta prévia, descolamento prematuro da placenta, baixo peso fetal e prematuridade, até mesmo em fumantes passivas.^108^ O consumo de bebida alcoólica deve ser evitado porque pode provocar atraso no crescimento fetal e anomalias da face e do sistema nervoso central.^109^

A gravidez em cardiopatas deve ser acompanhada por equipe multidisciplinar, com consultas mensais ao cardiologista durante a primeira metade da gestação, quinzenais após a 21ª semana e semanais até o parto, devendo respeitar a constante interação com o obstetra, o que assegura a melhor conduta nas diversas fases da gestação.

Entretanto, tais rotinas devem ser alteradas conforme a gravidade do caso. Nesse sentido, recomenda-se que gestantes em uso permanente de anticoagulantes tenham avaliação semanal para o controle clínico e laboratorial. Portadoras de cardiopatias com risco IV-OMS devem ser hospitalizadas no terceiro trimestre da gestação para estabilização clínica e planejamento do parto. Além disso, cuidados com alimentação, atividade física, qualidade do sono, redução do estresse e carga horária do trabalho, dependendo da atividade e da cardiopatia, devem ser recomendados. Ademais, a revisão e o ajuste das medicações de uso continuado, assim como a suspensão ou substituição daquelas que são prejudiciais ao feto, devem ser condutas antecipadas à concepção, ou seja, na fase do planejamento da gestação.

### 2.6.2. Atividade Física

Na gravidez não complicada, os benefícios da atividade física são indiscutíveis e incluem: melhora da resistência física e da função cardiorrespiratória; redução de estresse, ansiedade e risco de comorbidades relacionadas ao sedentarismo; e ganho ponderal.^110,111^ Contudo, o Colégio Americano de Obstetras e Ginecologistas contraindica o exercício na gravidez em portadoras de cardiopatias com repercussão hemodinâmica, pacientes classificadas como riscos III e IV-OMS e em casos de pré-eclâmpsia, hipertensão induzida pela gravidez, anemia grave e doença pulmonar restritiva.^112^

### 2.6.3. Dieta

A dieta equilibrada fornece nutrientes essenciais ao desenvolvimento do concepto e previne complicações relacionadas ao descontrole do peso na gestante. A obesidade está associada a aborto, recém-nascidos com baixo peso, macrossomia, diabetes gestacional, tromboembolismo e hipertensão gestacional, enquanto a desnutrição está ligada a prematuridade, baixo peso ao nascer e morte perinatal.^113^ A despeito da dieta adequada, as metas nutricionais exigem também suplementação oral, como apresentado na Tabela 17. A ingesta de alimentos ricos em ácido fólico e a suplementação nas doses de 1 a 5 mg/dia antes da concepção e durante o primeiro trimestre evitam o defeito do tubo neural em 72% dos casos.^114^ A suplementação de cálcio (≥ 1 g/dia) está associada à redução significativa no risco de pré-eclâmpsia (particularmente nas mulheres com ingesta baixa de cálcio), bem como à diminuição da prematuridade e da ocorrência do desfecho composto “morte materna ou morbidade grave”.^115^ A OMS recomenda 1,5 a 2 g de cálcio por dia para mulheres grávidas com baixa ingestão de cálcio na dieta.

**Tabela 17:** – Recomendações dietéticas durante a gestação

Ingestão calórica diária – 340 a 450 kcal adicionais	2.200 a 2.900 kcal/dia

Suplementos adicionados à dieta	
Acido fólico	1 a 5 mg/dia, pré-concepção
Ferro	27 mg > 20 semanas
Cálcio	250 a 1.000 mg/dia
Ácido fólico	0,4 mg; 0,6 mg no segundo e terceiro trimestres
Iodo	150 mcg/dia
Vitamina D	200 a 600 UI
Vitaminas A, E, C, B e Zinco	Quantidades variáveis, segundo e terceiro trimestres

O consumo de peixe é a principal fonte de exposição materna não ocupacional ao metilmercúrio, que é encontrado em todos os tecidos do peixe e é absorvido em mais de 95%. Contudo, apesar do risco de intoxicação pelo mercúrio, estudos de coorte mostraram que o maior consumo materno de peixe no pré-natal estava associado a melhor desenvolvimento neurológico em recém-nascidos^116^ e que a ingestão moderada (até três refeições por semana) antes das 22 semanas de gestação estava ligada à redução da prematuridade de repetição. Entretanto, recente revisão sistemática e metanálise de ensaios randomizados não mostraram significância estatística no efeito dos ácidos graxos poli-insaturados de cadeia longa (n-3 PUFA) em reduzir prematuridade ou qualquer outro efeito nos conceptos, como desenvolvimento neurológico, cognitivo ou acuidade visual.^117,118^

Quanto à ingesta de cafeína, em função da carência de estudos adequados, recomenda-se limitar seu consumo a menos de 200 mg/dia.^119^Deve-se ressaltar que o café, principal fonte de cafeína em muitos países, contém 50 a 70% mais cafeína que o chá e outros produtos. Admite-se uma relação teórica entre cafeína e arritmogênese, especialmente em pacientes cardiopatas.

A ingesta salina sem restrição significativa de sal é geralmente recomendada, em especial próximo ao parto. Entretanto, gestantes com risco de IC devem ser orientadas quanto à ingestão de 3 a 4 g de cloreto de sódio ao dia, sem adição de sal aos alimentos após cozimento, evitando itens salgados. Dieta com 2 g de sódio ao dia deve ser restrita a casos mais graves (CF III/IV-NYHA), assim como a orientação quanto à restrição hídrica nesses casos.

### 2.6.4. Atividade Profissional

Atualmente, a maioria das mulheres grávidas trabalha até um mês antes do parto, e apenas pequena porcentagem suspende suas atividades mais cedo. O risco de desenvolver complicações não tem relação com a atividade laboral ou o estresse psicossocial no trabalho.^120^ Entretanto, as exigências e condições laborais devem ser avaliadas individualmente em mulheres com cardiopatia limitante ou situações obstétricas como pré-eclâmpsia e RCIU. Mudanças ou adaptações nas atividades desenvolvidas, redução no estresse do trabalho ou aumento de períodos de repouso e relaxamento muitas vezes são medidas benéficas, principalmente se houver piora ou aparecimento de sintomas limitantes.

Gestantes portadoras de cardiopatias com repercussão hemodinâmica ou sintomáticas devem manter o repouso domiciliar a partir do início do terceiro trimestre da gravidez, ou seja, da 28ª semana de gestação. Nessa fase, a reserva cardíaca limitada pela cardiopatia é insuficiente para a adaptação ao máximo das alterações hemodinâmicas, favorecendo a ocorrência de IC, arritmias, RCIU e prematuridade.

Segundo as leis de proteção à gravidez, toda mulher tem direito a, no mínimo, seis dispensas para realizar exames e consultas, comprovadas por atestado médico, pelo tempo que o atestado indicar necessário para a realização dos procedimentos.^121^

### 2.6.5. Pontos-chaves

- O atendimento pré-natal à cardiopata é multidisciplinar, com consultas regulares ao cardiologista. A periodicidade deve estar de acordo com a gravidade da doença, sua evolução e/ou possíveis complicações;
- A orientação do estilo de vida deve ser individualizada de acordo com o risco cardíaco classificado pela OMS;
- A gestante deve ser conscientizada quanto a dieta, controle do peso, restrição da atividade física e controle do estresse laboral. Medidas como cessar o tabagismo e a ingestão de bebida alcoólica e consumir moderadamente cafeína são essenciais durante o pré-natal;
- O acompanhamento nutricional inclui o controle da ingesta calórica e a alimentação balanceada e rica em nutrientes, evitando consumo de produtos industrializados ou malcozidos e mal lavados;
- A suplementação de vitaminas e minerais essenciais deve estar incluída na rotina do pré-natal da gestante portadora de cardiopatia.

## 2.7. Conduta no Parto e Puerpério

A visão interdisciplinar do parto e puerpério em pacientes cardiopatas deve considerar a evolução clínica e obstétrica durante a gestação e a situação funcional no período que antecede o parto. A programação do parto em mulheres cardiopatas exige a prévia estabilização hemodinâmica, o rastreamento de possíveis intercorrências como infecção, anemia, hipertensão arterial e arritmias, e o ajuste da terapêutica cardiovascular.

Do ponto de vista obstétrico, são obrigatórios: a discussão com a cardiologista e anestesista sobre o momento e a via de parto, o monitoramento materno-fetal durante o trabalho de parto e a especial atenção para o balanço hídrico. Nesse sentido, gestantes portadoras de cardiopatias classes III/IV (OMS) necessitam de assistência em hospital terciário com retaguarda de unidade de terapia intensiva (UTI) no puerpério.

A equipe de atendimento à parturiente cardiopata deve estar preparada para prevenção e tratamento das principais complicações no intraparto e puerpério. Dentre elas, destacam-se IC, edema agudo dos pulmões, arritmias, tromboembolismo e dissecção de aorta como as complicações cardíacas mais frequentes, enquanto pré-eclâmpsia, hemorragia e infecções se incluem nas obstétricas.

### 2.7.1. Conduta no Parto

É de consenso geral que a via de parto é de indicação obstétrica. No entanto, em pacientes consideradas risco I/II-OMS^122^em condições clínicas e hemodinâmicas favoráveis, recomenda-se parto espontâneo no termo da gestação. O consenso sobre o tipo de parto é fundamentado na opinião de especialistas, que julgam o parto vaginal como mais vantajoso porque está associado a menor perda sanguínea, mais rápida recuperação e menor risco trombótico e infeccioso, sendo, portanto, preferido para mulheres cardiopatas com quadro clínico estável e não complicado.

No que diz respeito ao parto cesárea, sua incidência em pacientes com doença cardíaca varia, no mundo, entre 21% e 55%.^123^ Dados brasileiros disponíveis apontam um índice de cesariana na população geral ao redor de 52%, sendo que, na Rede Brasileira de Vigilância a Morbidade Materna Grave, a taxa chegou a 76% entre as cardiopatas.^124^ Não há justificativa clínica e nem explicação plausível para taxa tão elevada.

A proporção de parto cesárea reflete o nível de acesso a essa intervenção e seu uso; porém, a tarefa de definir qual a taxa “desejável” em determinada população, um número que mantenha as indicações médicas e, ao mesmo tempo, evite as cesáreas “desnecessárias”, é um grande desafio

O Registro Europeu sobre Cardiopatia e Gravidez mostrou que a frequência de parto vaginal programado foi de 69%; dentre os partos cesárea, 44% tiveram indicação cardiológica.^52^Nas conclusões, o registro mostrou que, em termos de resultados maternos, a cesárea programada não mostrou nenhuma vantagem sobre o parto vaginal programado e foi associada a pior evolução fetal.^125^

As indicações maternas ao parto cesárea abrangem situações clínicas muito específicas, tais como: trabalho de parto em pacientes sob anticoagulação oral, doenças com diâmetros de aorta aumentados (risco III/IV-OMS), coarctação de aorta grave, arterite de Takayasu, dissecção de aorta, HAP, IC aguda, congestão pulmonar em cardiopatia preexistente, cardiomiopatia, periparto (CMPP) com IC grave ou outras situações clínicas em que a condição materna seja crítica.^52^

Apesar de controverso, há recomendações do parto assistido, seja por vácuo-extração ou fórceps, em situações de real benefício materno-fetal pela abreviação da fase ativa do segundo estágio do trabalho de parto e do esforço do período expulsivo prolongado. Recomenda-se a utilização da posição reclinada e lateral esquerda para evitar a compressão da aorta e veia cava inferior pelo útero gravídico, favorecendo melhor retorno venoso materno, além de facilitar o esforço no período expulsivo.

O monitoramento básico durante o parto deve incluir a medida não invasiva de pressão arterial, a oximetria de pulso e a eletrocardiografia contínua, além de controle fetal (ausculta dos batimentos fetais pelo sonar doppler a cada 15 min no primeiro estágio, a cada 5 min no segundo estágio ou cardiotocografia contínua). A necessidade de monitoramento adicional deve ser vista caso a caso. Deve-se restringir a infusão excessiva de líquidos para evitar excessiva hidratação e congestão pulmonar.

Os benefícios da analgesia são indiscutíveis na prevenção da hipertensão arterial e da taquicardia, reduzindo o estresse cardíaco. Um modo seguro e eficaz de reduzir a ansiedade neste momento é humanizar a assistência ao parto, ou seja, requisitar a presença de um acompanhante de livre escolha, ter liberdade para deambulação e adotar posição mais confortável durante o trabalho de parto.

O parto em pacientes anticoaguladas deve ser programado a partir de 37 semanas de gestação. Aquelas com alto risco trombótico exigem o uso de HNF cerca de 36 horas antes do parto, sendo essa infusão interrompida 4 a 6 horas antes do nascimento e reintroiduzida 6 horas depois, com controle do TTPA. Em baixo risco trombótico, usa-se HBPM até o dia anterior ao parto, e a dose noturna deve ser omitida se a indução do parto ou a cesariana for realizada na manhã seguinte. Bloqueio regional é possível se transcorridas 24 horas da última dose.

A indução do trabalho de parto deve ser considerada com 40 semanas de gestação em todas as pacientes cardiopatas, porque o benefício dessa prática supera os eventuais riscos.^126^ O modo de indução depende principalmente da avaliação do colo uterino e da vitalidade fetal. Recomenda-se tanto o misoprostol (PGE1)^127^ como a dinoprostona (PGE2) para preparo do colo uterino. Os métodos como o de Krause (balão), a amniotomia e a infusão de ocitocina também são considerados seguros.^128^

Em contrapartida, a inibição do trabalho de parto prematuro deve ser considerada com muita cautela e até mesmo contraindicada em mulheres cardiopatas. O grau de prematuridade deve ser ponderado pelos riscos da tocólise e da terapêutica com os corticoides, porque ambas podem levar a complicações como IC grave e arritmias cardíacas.

Quando indicada, a tocólise deve ser mantida durante 48 horas, que corresponde ao tempo suficiente para a ação do corticoide, no intuito de reduzir a ocorrência de síndrome do desconforto respiratório, hemorragia peri e intraventricular e enterocolite necrosante do recém-nascido. Os fármacos utilizados na inibição, como a nifedipina, podem induzir hipotensão, além de apresentar sinergismo quando empregados juntamente ao sulfato de magnésio. A terbutalina tem intensos efeitos β-miméticos e pode levar a IC materna. Nessa situação, a atosibana, antagonista competitivo do receptor de ocitocina humana, tem sido o agente tocolítico mais seguro quando usado em infusão intravenosa de cerca de 400 ml de solução (soro fisiologico de 0,9%, lactato de Ringer ou solução de glicose a 5%) em 48 h (cerca de 200 ml/24 h).

### 2.7.2. Conduta no Puerpério

Os cuidados devem ser intensificados no período de puerpério, e as medidas preventivas das principais complicações (IC, HPP e tromboembolismo) devem fazer parte de protocolos nas maternidades de alto risco.

A volemia materna sofre importantes variações no pós-parto imediato, seja pelo aumento do retorno venoso após dequitação placentária, seja pela perda sanguínea estimada em até 500 ml e 1000 ml para parto vaginal e cesárea, respectivamente (definição da OMS e da Organização Pan-americana da Saúde). O impacto dessas oscilações na hemodinâmica materna explica a ocorrência de complicações graves, como IC, edema agudo dos pulmões e choque cardiogênico. O descuido quanto às oscilações hemodinâmicas no puerpério é, em parte, responsável pela mortalidade materna; por isso, é mandatório que pacientes com cardiopatias graves, mesmo estáveis, permaneçam no período de 24 a 48 h na UTI para monitoramento hemodinâmico efetivo.

De igual importância é a HPP, que ocorre em cerca de 10% dos partos vaginais, chegando a ser considerada grave em cerca de 3% deles. Em pacientes cardiopatas, a incidência de HPP chega a 21% e tem relação com parto cesárea, parto assistido com fórceps, anestesia geral e uso de heparina antes do parto.^129^ Na verdade, o aumento da morbidade materna por transfusão, infecção e tromboembolismo é a causa principal de morte em cardiopatas.

Por isso, toda maternidade deve ter protocolo específico para conduta na prevenção e no tratamento da HPP, que inclui o uso dos uterotônicos, recomendados durante a terceira fase para ambas as vias de parto, na prevenção da HPP.

A ocitocina é o fármaco recomendado pelo benefício na prevenção de hemorragia e deve ser aplicada por via intramuscular, na dose de 10 UI para parto vaginal ou cesárea. A via intravenosa é também uma opção, especialmente no parto cesárea, em doses ≤ 5 UI e em infusão lenta (> 30 segundos) a cada 3 minutos até três infusões. A profilaxia intravenosa deve ser associada à dose de manutenção em infusão contínua.

O misoprostol (600 a 1.000 µg) pode ser utilizado com segurança tanto para profilaxia como para tratamento da HPP, mas a ocitocina administrada em bólus deve ser evitada pelo risco de hipotensão. A ergometrina e a metilergometrina devem ser evitadas pela sua associação com a vasoconstrição coronária e a hipertensão arterial sistêmica (HAS).

Admite-se que o puerpério representa um período de alto risco para trombose; por isso, medidas como a deambulação precoce, mais factível no parto vaginal, e a anticoagulação com heparina devem ser recomendadas nas primeiras 48 horas após o parto. Contudo, a prevenção do tromboembolismo deve ser individualizada e será discutida adiante.

Quando houver indicação de esterilização definitiva, a salpingotripsia bilateral por incisão infraumbilical pode ser realizada nas primeiras 48 a 72 horas após o parto vaginal. De modo geral, a discussão da contracepção deve ser realizada antes da alta da maternidade.

### 2.7.3. Pontos-chaves e Recomendações

- Assistência multidisciplinar ao parto e ao puerpério deve considerar: estratificação de risco da cardiopatia e elaboração de protocolos na prevenção e no tratamento de IC, HPP, infecção e tromboembolismo;
- A assistência ao parto e puerpério deve ser realizada em maternidade de alto risco;
- Parto via vaginal, espontâneo e no termo da gestação é o recomendado para pacientes cardiopatas;
- Indicações maternas do parto cesárea: IC grave, doenças da aorta com dilatação importante, obstruções graves do coração esquerdo, formas graves de HP e disfunção ventricular;
- Parto cesárea é indicado em pacientes que iniciaram trabalho de parto espontâneo utilizando anticoagulantes orais (antagonistas da vitamina K) ou que suspenderam num período inferior a 15 dias;
- Parto em pacientes sob uso de anticoagulantes orais deve ser programado a partir de 37 semanas de gestação, para ajustes da anticoagulação sob o uso da heparina;
- Indicação de preparo de colo e indução do parto: misoprostol (PGE1) e dinoprostona (PGE2);
- Indicação de inibição do trabalho de parto, a princípio, é contraindicada. Em casos excepcionais, a atosibana é o agente tocolítico indicado;
- É exigência que as maternidades devem ter protocolo específico para prevenção e tratamento da HPP;
- Portadoras de cardiopatias graves devem permanecer na UTI no período de 24 a 48 horas após o parto;
- Contracepção deve ser discutida antes da alta da maternidade;
- Conscientização que o puerpério é tão importante e arriscado como a gravidez;
- Amamentação deve ser sempre incentivada.

## 2.8. Anestesia na Gestante Cardiopata

A anestesia obstétrica tem um papel fundamental na redução da morbi-mortalidade materno-fetal,^130^ principalmente no atendimento à gestante cardiopata. A complexidade das cardiopatias requer o envolvimento do anestesiologista nas discussões multidisciplinar no planejamento antenatal, intraparto e puerpério.

A frequência das avaliações deve ser conjugada ao risco da cardiopatia e à situação clínica das pacientes. De modo geral, a avaliação deve ser trimestral para aquelas em classe II de risco (OMS) e em intervalos de duas a quatro semanas para as de classes III e IV (OMS).^131^O planejamento formal de toda a equipe deve ser discutido entre 32 e 34 semanas de gestação,^132^de modo que a paciente seja admitida para o parto com uma orientação consolidada, o que auxilia no fluxo de atendimento e reduz o estresse das equipes nas urgências e a chance de desfechos negativos.

A equipe de anestesia terá a oportunidade de conhecer a evolução da gestação e as eventuais complicações, adequar a medicação na seleção da anestesia e analgesia e possibilitar a interação da paciente com maior clareza no entendimento das condutas tomadas no intraparto. É importante não negligenciar os riscos no manejo da via respiratória e a aspiração do conteúdo gástrico, os ajustes das eventuais medicações e a administração de uterotônicos no intraparto.

A programação atual para gestantes cardiopatas é o parto via vaginal com analgesia no neuroeixo. Esse tipo de anestesia é mais eficaz para o controle da dor na analgesia de parto quando comparada a outras técnicas, como o uso de opioides sistêmicos ou óxido nitroso inalatório.^133,134^ Quando efetiva, a analgesia espinhal diminui as catecolaminas endógenas circulantes, considerando-se que a simpatectomia parcial induzida pelo efeito do anestésico local no neuroeixo leva à queda da resistência vascular sistêmica e a alterações na frequência cardíaca relacionadas ao bloqueio simpático e aos reflexos cardíacos.

No parto cesárea, a anestesia no neuroeixo tem recebido destaque no manejo das gestantes cardiopatas, devido à crescente expertise dos anestesiologistas em utilizar técnicas de bloqueio espinhal que viabiliza melhor aferição da forma gradual da anestesia, diminuindo o impacto hemodinâmico.^135^ A literatura mundial demonstra taxas superiores a 60% em partos cesáreas eletivos realizados com bloqueio no neuroeixo, em menor número nos partos emergenciais, nos quais a anestesia geral é a escolhida em função da complexidade de fatores a serem avaliados para a tomada de decisão.^136,137^

Dentre as técnicas de neuroeixo mais utilizadas nas gestantes cardiopatas, podem-se destacar duas:

- Anestesia peridural sequencial;
- Anestesia combinada (raquiperidural) sequencial, com baixas doses no componente espinhal.

Essas técnicas são chamadas de “sequenciais”, pois permitem que a instalação e progressão cefálica do bloqueio simpático seja realizada gradualmente, prevenindo a instalação súbita e sua repercussão cardiovascular. Pequenas doses de anestésico local associado a opioides são utilizadas inicialmente, e complementações adicionais são realizadas através de cateter peridural, até se chegar ao nível sensitivo de T6.^138^

Nos casos que apresentam contraindicação à utilização do bloqueio do neuroeixo para cesariana, a anestesia geral deve ser realizada. O principal objetivo no manejo e planejamento dessa anestesia é minimizar os efeitos hemodinâmicos deletérios dos anestésicos sistêmicos e a resposta hipertensiva à laringoscopia, que é reflexo do estímulo simpático súbito e exacerbado. Nesse cenário, a avaliação pré-anestesia contribui para a identificação de anomalias do espaço dural e grave escoliose, situações habituais nas síndromes de Marfan, Loeys-Dietz ou Ehlers-Danlos.

Fármacos de ação rápida e com meia-vida curta são utilizados em doses de maneira conjugada à hemodinâmica materna, para atenuar as respostas simpáticas à laringoscopia, evitar grandes variações pressóricas e impedir o aumento da frequência cardíaca. Os opioides como alfentanil ou remifentanil podem ser úteis neste manejo, bem como a utilização de outras classes de fármacos. Betabloqueador de ação curta e anestésicos locais em doses adequadas, como o esmolol e a lidocaína, respectivamente, podem apresentar-se como boas opções. Além disso, o uso de indutores como cetamina e etomidato, em algumas circunstâncias, pode ser uma opção melhor em detrimento ao propofol, que apresenta maior potencial cardiodepressor quando utilizado em bólus ou em doses não ajustadas.

Na manutenção da anestesia geral, deve-se atentar para o potencial inotropismo negativo dos anestésicos inalatórios e a diminuição da resistência vascular sistêmica, fato que também ocorre com os anestésicos venosos. Pode haver também hipotonia uterina dose-dependente, com maior potencial de sangramento, relacionada ao uso de anestésicos inalatórios.^139,140^

Nos casos em que a anestesia geral foi empregada, um bom plano para analgesia pós-operatória deve ser realizado com o objetivo de reduzir as catecolaminas circulantes. Nesses casos, existem algumas opções, incluindo opioides espinhais ou analgesia peridural, realização de bloqueios de parede abdominal (bloqueio do transverso abdominal e bloqueio do quadrado lombar) ou utilização de analgesia sistêmica.

### 2.8.1. Jejum

Para o parto cesárea eletivo, recomenda-se jejum de sólidos de 6 a 8 horas, a depender do conteúdo de gordura ingerida e de eventuais alterações anatômicas ou fisiológicas que determinem maior retardo no esvaziamento gástrico. A ingesta de líquidos claros e sem resíduos pode acontecer até 2 horas antes da cirurgia. É recomendada a profilaxia farmacológica contra a aspiração do conteúdo gástrico, com antiácidos não particulados, antagonistas do receptor H_2_ e antagonistas dopaminérgicos. Durante o trabalho de parto, gestantes de baixo risco podem ingerir moderada quantidade de líquidos sem resíduos,^141^como água, chá, gelatina e isotônicos.

Na eventual instabilidade do quadro hemodinâmico materno, a equipe deve manter o jejum da mãe até que haja segurança para a reintrodução de líquidos. A analgesia de parto e a proximidade da segunda fase do trabalho de parto são pontos que exemplificam bem essa conduta, haja vista a possibilidade de instabilidade hemodinâmica e sangramento, respectivamente. Nas pacientes mais graves ou diante da maior probabilidade de parto cesárea, a paciente deve permanecer em jejum.

### 2.8.2. Anticoagulação e Bloqueio do Neuroeixo

Estima-se que o hematoma espinhal ocorra em 1:200.000 a 1:250.000 partos.^142,143^ Embora raro, é um evento grave; logo, estratégias devem ser tomadas para sua prevenção. As recomendações atuais consideram que a dose dos anticoagulantes e o período prévio de supensão deles são os parâmetros para assegurar uma anestesia segura no neuroeixo. Isso vale para pacientes recebendo anticoagulação única, com peso acima de 40 kg, função renal normal e sem outras condições que contraindiquem o bloqueio de neuroeixo.^144^ Em resumo, as recomendações estão explicadas a seguir.

### 2.8.3. Heparina Não Fracionada (Subcutânea)144

- Dose baixa (5.000 UI, 2 a 3 vezes/dia): aguardar 4 a 6 h, com TTPA normal ou fator antiXa indetectável;
- Dose intermediária (7.500 a 10.000 UI, 2 vezes/dia; 20.000 ou menos UI/dia): aguardar 12 h ou mais, com TTPA normal ou fator antiXa indetectável;
- Dose alta (Mais de 10.000 UI por dose; mais de 20.000 UI/dia): aguardar 24 h ou mais, com TTPA normal ou antiXa indetectável.

### 2.8.4. Heparina de Baixo Peso Molecular (Subcutânea)144

- Dose profilática (enoxaparina 40 mg ou deltaparina 5.000 UI 1 vez/dia): aguardar 12 h ou mais;
- Dose terapêutica (enoxaparina 1 mg/kg, 2 vezes/dia, ou deltaparina 120 UI/kg, 2 vezes/dia ou 200 UI/kg, dose única): aguardar 24 h.

Quaisquer regimes de anticoagulação que sejam diferentes dos mencionados devem ser avaliados e individualizados pela equipe, levando-se em consideração não apenas o risco de hematoma espinhal, mas também os riscos tromboembólicos, o tempo de jejum, as condições materno-fetais e a avaliação dos preditores de intubação difícil para anestesia geral.

Os tempos para reiniciar a anticoagulação devem obrigatoriamente ter a participação do anestesiologista, nos casos em que houve abordagem do neuroeixo. A reintrodução da anticoagulação deve ser individualizada porque existem condições técnicas na realização da anestesia raquidiana ou peridural que interferem nas condutas de reintrodução dos anticoagulantes.

### 2.8.5. Monitoramento Hemodinâmico

O uso de monitoramento invasivo nas pacientes de alto risco deve ser considerado para o reconhecimento antecipado do agravamento hemodinamico e o imediato tratamento. O monitoramento da pressão arterial invasiva (PAi) antes da aplicação do bloqueio do neuroeixo e da indução da anestesia geral nas pacientes com risco III e IV-OMS é importante para um desfecho melhor.^140^

A carência de validação para a utilização de métodos não invasivos no monitoramento do débito cardíaco permite em casos selecionados indicar a utilização de monitores invasivos, como cateter venoso central (CVC) e cateter de artéria pulmonar (CAP). Contudo, as informações obtidas podem ser imprecisas pela complexidade das cardiopatias, além do risco de arritmias induzidas e outras complicações. Por isso, há uma baixa adesão ao uso desses monitores. A ecocardiografia transtorácica intermitente e a ecocardiografia transesofágica, nos casos de cesariana com anestesia geral, têm ganhado espaço como opção de monitoramento de gestantes graves e podem ajudar na avaliação da função e do enchimento ventricular.^140^

### 2.8.6. Uterotônicos Intraparto

O uterotônico mais utilizado no periparto é a ocitocina, que tem um efeito brusco na resistência vascular sistêmica quando administrada em altas doses ou infusão rápida. Esses regimes devem ser evitados em qualquer gestante, principalmente nas portadoras de cardiopatia. A infusão de 2 UI do fármaco em 10 min parece ser efetiva e sem efeitos cardiovasculares significantes nas gestantes com cardiopatia.^135^ Em geral, é possível manter uma infusão de 2 a 5 UI em um intervalo de 15 a 30 min com baixos efeitos cardiovasculares. Os derivados do ergot induzem contração da musculatura lisa com vasoconstrição e consequente hipertensão. O misoprostol pode causar hipertermia e tremor e resulta em aumento do consumo de oxigênio, às vezes prejudicial, particularmente nas cardiopatas graves.

É controversa a regra mais utilizada pelos anestesistas para a administração da ocitocina em pacientes sem comorbidades. Trata-se da “regra dos 3”, que significa 3 UI a cada três min até 3 vezes enquanto a infusão de ocitocina 2 UI em 10 min parece ser lenta demais.^139,142^

### 2.8.7. Pós-parto

O acompanhamento das gestantes com risco alto e intermediário deve acontecer em UTI por 24 a 48 horas. Esse período de observação é importante, tendo em vista que a maior parte das mortes ocorre no puerpério. Monitoramento inadequado e manejo volêmico inapropriado podem levar à disfunção cardiovascular.^140^

### 2.8.8. Pontos-chaves

- O planejamento da anestesia para o parto de mulheres cardiopatas deve ser discutido com a equipe multidisciplinar entre 32 e 34 semanas de gestação;
- A programação para gestantes cardiopatas é o parto via vaginal com analgesia do neuroeixo;
- Nos casos de parto cesárea, a anestesia no neuroeixo tem recebido destaque em utilizar técnicas do bloqueio espinhal;
- A anestesia geral é indicada em casos de cardiopatias graves;
- A anestesia deve ser individualizada em pacientes sob anticoagulação;
- O monitoramento materno é indispensável durante o parto e o puerpério imediato.

## 3. Avaliação e Conduta das Doenças Cardíacas Durante a Gravidez

### 3.1. Doença Valvar

No Brasil, a doença reumática é a causa mais frequente de cardiopatia durante a gravidez, com incidência estimada em 50% em relação a outras cardiopatias.^145^ Admitindo-se que o surto reumático é um episódio da primeira infância e/ou da adolescência, o início da fase clínica coincide com a idade fértil da mulher.

A adaptação cardiocirculatória das doenças valvares ao aumento do débito cardíaco influencia diretamente o fluxo através das valvas cardíacas, com piora funcional nas lesões estenóticas. Por outro lado, a queda da resistência vascular periférica reduz o volume de regurgitação nas valvas insuficientes. Por essas razões, a evolução das lesões estenóticas geralmente é pior e se correlaciona ao grau anatômico da lesão valvar, enquanto as regurgitantes, à condição da função ventricular.^146^

Essas considerações iniciais auxiliam a estratificação de risco das valvopatias, tanto para um aconselhamento adequado no planejamento familiar como no atendimento durante a gestação. Nesse sentido, a classificação elaborada pela OMS foi adaptada às portadoras de valvopatias para a gestação.

A OMS julga que pacientes classificadas como I e II apresentam risco aceitável ou baixo e não impõem sérias restrições à gestação; já o risco III desaconselha a gravidez, e o risco IV a contraindica.^147^ Nesse posicionamento, as pacientes valvopatas são qualificadas da maneira a seguir: risco I, aceitável; riscos II e III, intermediário; e risco IV, alto risco à gravidez (Tabela 18).

**Tabela 18:** – Classificação dos riscos das valvopatias para a gravidez

Risco alto	Risco intermediário	Risco aceitável
Estenose mitral grave	PB com disfunção moderada	Valvopatia discreta
Estenose aórtica grave	Estenose pulmonar grave	PB sem disfunção
PB estenótica/calcificada		
PM com disfunção		
Valvopatia + HP importante (PAP ≥ 50 mmHg)	PM	Valvopatia + FEVE normal
	PM mitral > risco PM aorta	
Insuficiência aórtica + doenças de aorta	Insuficiência aórtica + doenças da aorta	Valvopatia sem fatores desfavoráveis
Síndrome de Marfan (DAorta > 45 mm)	Síndrome de Marfan (DAorta entre 40 e 45 mm)	
Valva aórtica bicúspide (DAorta > 50 mm)	Valva aórtica bicúspide (DAorta 45 a 50 mm)	
Valvopatia + FEVE < 35%	Necessidade de uso de anticoagulantes	

*DAorta: diâmetro da aorta ascendente; FEVE: fração de ejeção do ventrículo esquerdo; HP: hipertensão pulmonar; PAP: pressão arterial pulmonar; PB: prótese valvar biológica; PM: prótese valvar mecânica. Considera-se estenose mitral ou aórtica grave: área valvar mitral ≤ 1,0 cm^2^ e área valvar aórtica < 1,0 cm^2^, respectivamente.*

Vale acrescentar que a situação de alto risco em valvopatias não preenche os critérios para a indicação de interrupção da gestação (aborto terapêutico), uma vez que essas pacientes podem ser tratadas pela intervenção tanto cirúrgica como percutânea após a fase de embriogênese.

No planejamento familiar, a avaliação da valvopatia deve estabelecer os diagnósticos etiológico, anatômico e funcional e investigar a presença de fatores desfavoráveis que fazem parte da história natural das valvopatias e da correção cirúrgica prévia,^148^ que modificam o prognóstico materno e independem da lesão cardíaca estrutural *per se*, merecendo destaque especial:

- FA;
- HP;
- Disfunção ventricular;
- Doenças da aorta associadas;
- Antecedentes de IC, tromboembolismo ou endocardite infecciosa (EI).

A avaliação cardiológica prévia à gestação inclui a história, o exame físico e exames subsidiários que auxiliam na classificação do risco à gravidez, como:

- ECG: avalia o ritmo e a sobrecarga de câmaras cardíacas;
- ECO: informa tipo e gravidade da valvopatia, grau de dilatação ventricular, presença de disfunção ventricular, HP e defeitos associados;
- RMC: útil quando a valvopatia está associada às doenças da aorta;
- Teste ergométrico: válido na estimativa da capacidade funcional e da pressão arterial na estenose aórtica grave em pacientes assintomáticas e quando há dissociação entre os sintomas e o grau anatômico da estenose mitral. É somente indicado no planejamento de gravidez;
- Biomarcadores: aplicação controversa em valvopatias.

As recomendações pautadas em Diretrizes Nacionais^149,150^ e Internacionais^151^ para a conduta no planejamento familiar e durante a gravidez em valvopatias adquiridas, congênitas e próteses valvares estão apresentadas nas Tabelas 19 a 22.

**Tabela 19:** – Recomendações para conduta em valvas naturais adquiridas e congênitas149,150

Valvopatia	Aconselhamento na pré-concepção	Gestação		

		Risco materno	Risco fetal	Intervenção
**Estenose mitral** reumática grave	CF ≥ II **ou** assintomática + HP > 50 mmHg **ou** FA início recente **considerar** VCB ou CEC	Risco aumentado **se** • IC • FA Morte < 3%	Prematuridade 20 a 30% RCIU 5 a 20% Natimorto Aumenta em CF III/IV materna	Betabloqueador diurético Anticoagulação se FA **se** CF III/IV refratária **considerar** VCB ou CEC
**Estenose aórtica grave** reumática congênita (bicúspide) degenerativa	Sintomática **ou** assintomática + ECGE alterado **ou** FE < 50% **ou** AVAo < 0,7 cm^2^ gradiente médio > 60 mmHg **ou** Valva bicúspide + DAorta > 45 mm **considerar** VCP ou CEC	Risco aumentado IC - 10% Arritmia 3 a 25% Síncope Morte súbita	Complicações - 25% Prematuridade RCIU Baixo peso nascer Natimorto	Repouso diuréticos com critério **Se** FA betabloqueador ou BCC Anticoagulação IC grave ou síncope **considerar** VCB ou CEC
**Insuficiência mitral** Importante reumática prolapso valvar degenerativo	CF ≥ II **ou** Assintomática complicada + FE ≤ 60% +PSAP ≥ 50 mmHg + DSVE ≥ 40 mm **considerar** CEC (plastia ou prótese)	IC FA Risco aumenta com FE < 35%	Baixo risco	diurético hidralazina digoxina ***Se*** IC refratária ***considerar*** CEC ou “mitra clip”
**Insuficiência aórtica** importante reumática congênita (bicúspide) denegerativa	Sintomática CF ≥ II **ou** Fatores desfavoráveis FE < 50% DDVE > 70 mm (75 **se** reumático) DSVE > 50 mm (55 **se** reumático) **considerar CEC Se** Valva bicúspide isolada Daorta > 45 mm **considerar** intervenção na aorta proximal	Baixo risco Assintomática FE normal CF > II ou FE < 35% IC e/ou FA	Baixo risco	diurético hidralazina digoxina **Se** IC refratária **considerar Se** Valva bicúspide DAorta > 45 mm **considerar** Intervenção na aorta proximal

*AVAo: área valvar aórtica; AVM: área valvar mitral; BBC: bloqueador do canal de cálcio; CEC: circulação extracorpórea; CF: classe funcional; DAorta: diâmetro de aorta; DDVE: diâmetro diastólico do ventrículo esquerdo; DSVE: diâmetro sístólico do ventrículo esquerdo; EAo: estenose aórtica; ECGE: teste ergométrico; ECO: ecocardiograma; EM: estenose mitral; FA: fibrilação atrial; FE: fração ejeção ecocardiográfica; HP: hipertensão pulmonar; IC: insuficiência cardíaca; NYHA: New York Heart Association; PSAP: pressão sistólica da artéria pulmonar; CIUR: crescimento intrauterino restrito; VCB: valvoplastia por cateter balão. Considera-se estenose mitral ou aórtica grave: AVM ≤ 1,0 cm^2^ e AVAo < 1,0 cm^2^, respectivamente.*

**Tabela 20:** – Recomendações para a conduta nas valvopatias congênitas ou adquiridas por endocardite infeciosa149,150

Valvopatia	Aconselhamento na pré-concepção intervenção	Gestação		

		Risco materno	Risco fetal	Intervenção
**IT estrutural** *Anomalia de Ebstein*	IT grave sintomática Dilatação/disfunção do VD importante **considerar** cirurgia conservadora (plastia) **ou** Implante de PB	Lesões moderada/grave IC direita Arritmias supraventriculares	Risco baixo	Diurético Digoxina **Se** IC direita grave **considerar** cirurgia conservadora (plastia) Implante de PB
Estenose pulmonar grave	Dispneia de esforço/fadiga Hipoxemia Angina atípica IC direita (IT secundária) VCB ou CEC	Síncope IC direita Arritmia atrial Hipoxemia	Risco baixo	**Se** Hipóxia /IC grave **Considerar** VCB

*CEC: circulação extracorpórea; EI: endocardite infecciosa; IC: insuficiência cardíaca; IT: insuficiência tricúspide; PB prótese valvar biológica; VCB: valvoplastia por cateter balão.*

**Tabela 21:** – Prótese valvar com função normal e riscos para a gestação

Prótese biológica com FE normal		Prótese mecânica com FE normal	

Risco materno	Risco fetal	Risco materno	Resultados fetais
Não requer anticoagulação Risco baixo	Risco baixo	**Requer anticoagulação** Risco intermediário	Alto risco Embriopatia varfarínica Aborto Prematuro Natimorto Hemorragia perinatal
		Anticoagulação favorece a Hemorragia Embolia sistêmica **Se** Trombose prótese **considerar** Tratamento de emergência Trombólise ou CEC	

*CEC: circulação extracorpórea; FE: fração de ejeção.*

**Tabela 22:** – Conduta em próteses com disfunção durante a gestação149,150

Prótese biológica		Prótese mecânica	

Risco materno	Risco fetal	Risco materno	Risco fetal
Disfunção com predomínio de insuficiência, CF I/II e FE normal Medidas farmacológicas	Risco baixo	Disfunção com insuficiência “para valvar” leve a moderada sem hemólise significativa ou IC grave **considerar** Medidas farmacológicas para IC e anemia **Se** Insuficiência severa ou hemólise significativa **considerar** Intervenção Se IC e/ou hemólise sintomáticas **considerar** Fechamento percutâneo do escape (leak) para valvar por meio de dispositivo (Plug) **ou** CEC (alto risco de recidiva)	Alto risco fetal se CEC
Disfunção com predomínio de estenose valvar com calcificação (mitral, aórtica ou tricúspide) Riscos de IC grave, Choque, morte súbita **Sempre considerar Emergência** Implante percutâneo **ou** transapical de nova PB “valve in valve”* **ou** CEC	Alto risco fetal Perda fetal Prematuridade Natimorto	Estenose PM aórtica ou mitral por crescimento endotelial intravalvar – “Pannus”: necessidade de intervenção é rara Se indicado: **considerar** CEC Estenose PM (geralmente aórtica) “mismatch” Necessidade de intervenção é rara Se indicado, **considerar** CEC	Alto risco fetal se CEC

*CEC: circulação extracorpórea; CF: classe funcional; FE: fração de ejeção; IC: insuficiência cardíaca; PM: prótese mecânica.*

#### 3.1.1. Considerações Gerais sobre a Terapêutica

A restrição moderada de sal e de atividade física, o controle do ganho ponderal (não acima de 10 kg) e a suplementação de ferro após 20 semanas de gestação são as recomendações iniciais, cuidando para afastar fatores como anemia, infecção, hipertireoidismo e arritmias cardíacas. A prevenção do surto reumático deve ser mantida com penicilina benzatina na dose de 1.200.000 UI a cada 21 dias ou estearato de eritromicina, 500 mg, a cada 12 horas, quando alergia à penicilina. A sulfadiazina é contraindicada. A prevenção da EI para o parto é realizada com ampicilina 2 g por via intravenosa associada a gentamicina 1,5 mg/kg por via intramuscular (dose máxima de 120 mg) 1 hora antes do parto. A segurança e eficácia do tratamento farmacológico requer ajustes periódicos da posologia.

Antes da concepção, os fármacos de reconhecidos efeitos teratogênicos devem ser substituídos. Em portadoras de estenose mitral, destaca-se o uso de propranolol ou metoprolol em doses que não ultrapassem 80 e 75 mg, respectivamente, na prevenção e no controle da congestão pulmonar, atentando-se sempre para os efeitos colaterais perinatais, como hipoglicemia, hiperbilirrubinemia e policitemia, os quais não têm sido verificados nessas doses recomendadas.

A FA aguda deve ser prontamente revertida por cardioversão elétrica em portadoras de valvopatia mitral, pois esse procedimento é considerado inócuo ao concepto e tem a vantagem de evitar a utilização de fármacos em doses às vezes tóxicas. Por outro lado, batimentos ectópicos atrial ou ventricular e taquicardia atrial assintomática não exigem uso de antiarrítmicos. Para o controle da frequência em pacientes com FA permanente, devem ser considerados os betabloqueadores ou bloqueadores de canais de cálcio não di-hidropiridínicos, além de anticogulação.

A necessidade de intervenção nas valvopatias durante a gestação se deve aos casos refratários ao tratamento clínico. Os procedimentos percutâneos devem ser preferidos à cirurgia com circulação extracorpórea (CEC). A valvoplastia por cateter balão (VCB) na estenose aórtica tem sido indicada nas valvopatias de etiologia congênita ou na tentativa de resgate da vida materna e nos casos extremos de gravidade. A mitral requer ausência de trombo em átrio esquerdo, insuficiência mitral no máximo discreta e escore ecocardiográfico de Wilkins ≤ 8.^149^

#### 3.1.2. Pontos-chaves: Gravidez em Valvopatias – Valva Nativa

- Lesão valvar estenótica complica mais que a de regurgitação;
- Classe funcional I/II (NYHA) em lesões estenóticas não asseguram a boa evolução materna;
- Fatores complicadores aumentam significativamente o risco das valvopatias;
- Deve-se considerar intervenção percutânea antes da gestação em portadoras de estenose mitral e aórtica graves, mesmo em pacientes assintomáticas;
- A gravidez não modifica os critérios de indicação da valvoplastia por cateter-balão;
- Tratamento farmacológico das complicações durante a gestação deve ser considerado como primeira opção terapêutica;
- Manutenção da profilaxia da doença reumática durante toda a gestação;
- A consulta no pós-parto, além do exame clínico materno e avaliação da saúde do bebê, inclui ajuste da medicação, estímulo à lactação e orientação à contracepção.

#### 3.1.3. Prótese Valvar

A prevalência da doença reumática no Brasil e o crescente número de pacientes com cardiopatia congênita que requerem substituição valvar determinaram um aumento de portadoras de próteses valvares no período fértil. Um fator favorável, nessa faixa etária, é o desempenho ventricular esquerdo, que geralmente é conservado.

Do ponto de vista hemodinâmico, próteses valvares, tanto as mecânicas como as biológicas, melhoram a capacidade funcional e proporcionam semelhante evolução clínica durante a gravidez. As próteses biológicas têm atributos favoráveis à evolução da gravidez por não requererem anticoagulação e por apresentarem, em relação a outras cardiopatias, morbimortalidade materno-fetal considerada risco II-OMS. Contudo, elas têm durabilidade limitada, com possibilidade de reoperação em curto prazo, inclusive durante a gravidez.

A disfunção da prótese valvar biológica (PB) devido à calcificação tem má evolução, acarreta a congestão pulmonar e o baixo débito cardíaco, ambos refratários ao tratamento clínico, além de causar alto risco de morte súbita (risco IV-OMS). A ocorrência de calcificação de PB durante a gravidez torna obrigatória a indicação cirúrgica de substituição valvar, independentemente da idade gestacional.^152^

Em contrapartida, a gestação em portadora de prótese mecânica (PM) é considerada risco III - OMS. O risco de trombose devido à hipercoagulabilidade materna e a dificuldade da anticoagulação permanente associam-se à incidência variável de acidentes embólicos, abortamento espontâneo, embriopatia varfarínica e fenômenos hemorrágicos maternos e neonatais.^96^

Estima-se que a chance de gravidez livre de eventos materno-fetais seja de 58,3% para a PB e 46,9% para a PM,^96,153^ sendo que as taxas de mortalidade materna não são diferentes. Embora controverso, não há prótese valvar segura para a gravidez; contudo, a PB pode ser considerada a substituta mais adequada para a mulher em idade fértil, exceto em adolescentes em que a calcificação prematura da biológica favorece a escolha da PM.

Alguns fatores relacionados ao prognóstico da gravidez são:

- Estado funcional da prótese;
- Ritmo cardíaco (FA);
- Disfunção ventricular;
- CF (NYHA);
- Antecedentes de EI, IC ou tromboembolismo.

#### 3.1.4. Risco Materno

Pacientes portadoras de PM apresentam um risco estimado de 5% de trombose valvar durante a gestação, e a mortalidade materna varia entre 9 e 20%, associada a complicações tromboembólicas.^96^A incidência de trombose da PM varia conforme o regime de anticoagulação, sendo significativamente maior na fase de transição com a heparina.^154-157^ A incidência de tromboembolismo com a HBPM se deve às flutuações do fator antiXa que ocorrem nas 24 horas,^158^ mesmo com o valor terapêutico (0,6 a 1,2 UI/ml) no pico de ação após 4 h da aplicação,^159^ resultando em um nível subótimo de anticoagulação. Quanto a HNF em uso prolongado associa-se a trombocitopenia e osteoporose,^154,155^tem eficácia inferior à da HBPM, e seu uso por via subcutânea foi abolido na prática da anticoagulação. Em razão da alta incidência de tromboembolismo com as heparinas (HNF e HBPM), existe uma tendência a se priorizar o uso da varfarina durante toda a gravidez, por acreditar-se na sua maior segurança no desfecho materno-fetal.^154-157^

#### 3.1.5. Riscos para o Concepto

Para todos os esquemas de anticoagulação, os riscos obstétricos de hemorragia, descolamento prematuro de placenta que causa prematuridade e morte fetal são muito elevados.^155-157^ A varfarina sódica atravessa a barreira placentária e, quando usada no primeiro trimestre, é teratogênica, acarretando 0,6 a 10% de malformação caracterizada pela síndrome varfarínica fetal,^160^ mesmo em doses inferiores a 5mg.^161-163^

O esquema da anticoagulação para pacientes portadoras de PM que desejam engravidar ou se apresentam em curso da gestação na primeira consulta é ainda controverso. Fatores que devem ser levados em consideração na decisão do melhor tratamento anticoagulante incluem: preferências da paciente, *expertise* do médico assistente e disponibilidade de controle adequado da coagulação.

As recomendações para a prevenção de tromboembolismo em próteses mecânicas pretendem atender aos requisitos ideais de um posicionamento com base na documentação da literatura e na vivência dos autores, de modo que seja efetiva para a realidade dos diversos serviços. Entende-se que a dinâmica da anticoagulação permanente deva ser dividida em cinco momentos: na pré-concepção, em cada trimestre e no puerpério, conforme explicado a seguir.

**Momento 1: Pré-concepção: Conscientização da paciente/casal. Orientação quanto ao diagnóstico precoce da gravidez*.*** A paciente que planeja a gravidez deve ser esclarecida sobre a obrigatoriedade de manter a anticoagulação e a disponibilidade dos esquemas e os seus riscos em todas às fases da gestação, do parto e do puerpério. Para isso, o diálogo franco com o casal é fundamental. A orientação também inclui a informação sobre a importância do diagnóstico precoce da gravidez em reduzir a ocorrência da embriopatia. Nesta consulta, é fornecido um pedido de exame para a dosagem de gonadotrofina coriônica beta, que deve ser realizado à primeira dúvida de atraso menstrual.

**Figura 6:**
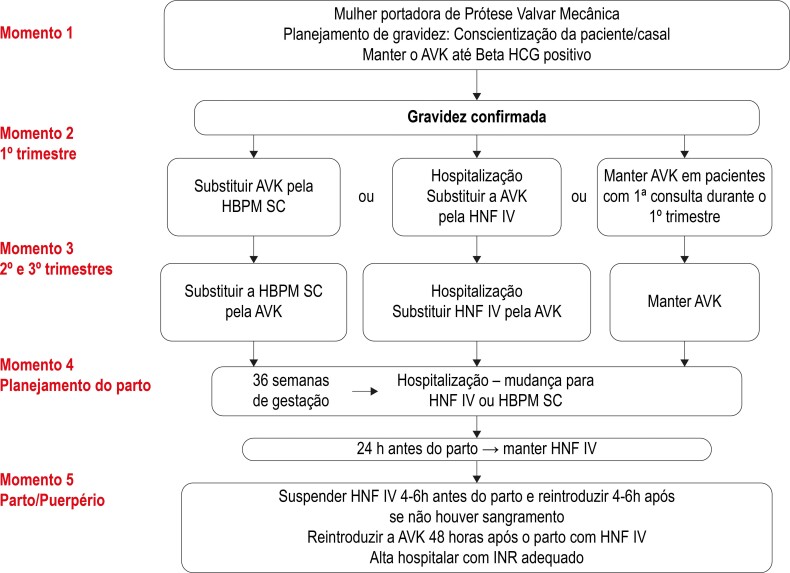
– *Recomendações para a anticoagulação em prótese valvar mecânica durante a gestação. AVK: antagonistas da vitamina K; HBPM SC: heparina de baixo peso molecular por via subcutânea; HCG: hormônio gonadotrofina coriônica; HNF IV: heparina não fracionada por via intravenosa; TTPA: tempo de tromboplastina parcial ativado. HBPM SC 12/12h = 1 mg/kg/dose; HNF IV = 18 UI/kg/h. Controle de dose e metas: HBPM SC = fator antiXa entre 0,6 e 1,2 U/ml semanal; HNF IV TTPA: 2 vezes o valor normal/diário; AVK = INR 2,5 a 3,5 quinzenal.*

**Momento 2: Primeiro trimestre. Substituição do anticoagulante (evitar a teratogênese).** A substituição da varfarina pela heparina possibilita uma conciliação entre o benefício da prevenção de trombose materna e o malefício da embriopatia. Neste momento existem opções que estão apresentadas na Figura 6. A primeira escolha é o uso de HBPM, que exige o controle semanal do fator antiXa. Na indisponibilidade dessa opção, é indicada a HNF intravenosa no período entre a sexta e a nona semana de gestação. Em pacientes que chegam na primeira visita médica depois da sexta semana de idade gestacional, a varfarina não deve ser suspensa. O casal deve ser informado da possibilidade de embriopatia e de os riscos da substituição pela heparina não serem mais justificados.

**Momento 3: Segundo e terceiro trimestres. Retorno ao anticoagulante oral, controle da anticoagulação e prevenção de hemorragia.** O retorno para a varfarina justifica-se pela suposição de que abreviar o uso da heparina reduz os efeitos adversos à mãe e provoca menor risco de embriopatia. A proposta é manter as doses de varfarina de acordo com as metas de antes da gestação, com controle do INR semanal ou quinzenal. A reintrodução da varfarina deve obedecer à dinâmica da transição, ou seja, simultaneamente à HBPM (via subcutânea) ou à HNF (via intravenosa) até o INR alcançar o “valor-alvo” (Figura 6).

**Momento 4: Planejamento do parto. Considerar a hospitalização, redirecionar para a anticoagulação parenteral.** A hospitalização deve ser programada com 36 semanas de gestação para o uso de HBPM por via subcutânea ou HNF por via intravenosa em doses terapêuticas (Tabela 23). A via de parto é discutida com o obstetra. Em parto prematuro sob anticoagulação, a via de parto é a cesárea e pode ser considerada o uso de complexo protrombínico.

**Tabela 23:** – Recomendação quanto a dose e controle da anticoagulação em prótese mecânica durante a gravidez

Idade gestacional	Anticoagulante	Controle
Entre a 6ª e 9ª semanas	HBPM 1,0 mg/kg SC 12/12h HNF IV 18 UI/kg/hora em bomba de infusão (< 30.000 UI IV)	AntiXa: 0,6 a 1,2 U/ml TTPA 1,5 a 2,0 vezes/VN
12ª até a 36ª semana	Varfarina de acordo com INR	Aórtica INR entre 2,5 e 3,0 Mitral INR 3,0 e 3,5
Após a 36ª semana até o parto	HBPM 1,0 mg/kg SC 12/12h HNF IV 18 UI/Kg/hora em bomba de infusão (< 30.000 UI IV)	AntiXa: 0,6 a 1,2 U/ml TTPA 1,5 a 2,0 vezes/VN
Puerpério	HBPM 1,0 mg/kg SC 12/12h HNF IV 18 UI/Kg/hora em bomba de infusão (< 30.000 UI IV) Varfarina alcançar INR-alvo para alta hospitalar	AntiXa: 0,6 a 1,2 U/ml TTPA 1,5 a 2,0 vezes/VN INR entre 2,0 e 2,5

*HBPM: heparina de baixo peso molecular; HNF: heparina não fracionada; INR: índice internacional normalizado; SC: subcutâneo; UI: unidades internacionais; VN: valor normal; IV: via intravenosa; TTPA: tempo de tromboplastina parcial ativado.*

**Momento 5: Puerpério. Reintrodução da anticoagulação oral e alta hospitalar*.*** Decorridas 6 h do parto e em ausência de complicação materna, a HNF via intravenosa ou a HBPM via subcutânea em doses terapêuticas devem ser reintroduzidas. A varfarina deve ser prescrita 48 h após o parto, obedecendo à dinâmica de transição em conjunto com a heparina até o valor de 2,0 do INR, quando é dada a alta hospitalar.

#### 3.1.6. Pontos-chaves: Gravidez e Prótese Valvar

- A PB não requer anticoagulação, exceto em pacientes com FA ou acidente tromboembólico prévio;
- A gravidez não influencia na degeneração estrutural da PB;
- PB calcificada e estenótica tem indicação cirúrgica independente da idade gestacional;
- A PM requer anticoagulação com ajuste permanente na busca das metas convencionais;
- A trombose de PM demanda intervenção imediata com trombolítico ou cirurgia de emergência com CEC, independentemente da idade gestacional;
- A escolha da PB como substituta preferível para mulher em idade fértil considera a não necessidade do uso de anticoagulação e a perspectiva futura da troca valvar percutânea (“valve in valve”);
- O procedimento percutâneo “valve in valve” requer centro especializado com “heart team” e recursos de tomografias valvar e arterial, ECO tridimensional esofágico e equipe hemodinâmica intervencionista e de cirurgia de prontidão;
- Portadoras de PM devem ser encaminhadas a serviços terciários e de referência em doença valvar para o acompanhamento da gravidez;
- A anticoagulação permanente em pacientes com próteses mecânicas ou doença valvar mitral com FA deve obedecer os algoritmo que inclui cinco momentos do ciclo gravídico-puerperal;
- A despeito do controle adequado e efetivo da anticoagulação em todos os momentos, ainda permanecem incertezas quanto ao sucesso da gravidez em mulheres portadoras de PM;
- A escolha da prótese valvar com perspectivas de futura gravidez deve ser discutida com a equipe multidisciplinar em conjunto com a paciente.

## 3.2. Cardiopatias Congênitas

O avanço do tratamento clínico-cirúrgico em cardiologia tem demonstrado que um número progressivamente maior de mulheres portadoras de CC consegue alcançar a idade reprodutiva^164^ e o desejo de engravidar, com grande oportunidade de sucesso materno-fetal.^165^

No Brasil, observa-se uma tendência ao crescimento do percentual de gestantes portadoras de CC, à semelhança do que já acontece em países europeus. A mortalidade materna na lesão cardíaca congênita é considerada a segunda causa indireta de óbito materno, alcançando até 20% das mortes por doença cardíaca.^166^

O planejamento da gravidez em portadoras de CC deve considerar: (1) o tempo do diagnóstico da cardiopatia; (2) cirurgia paliativa ou corretiva pregressa; (3) classe funcional (NYHA); (4) exames laboratoriais tais como o hematócrito, hemoglobina, saturação de oxigênio e peptídeo natriurético, provas de função hepática e tireoidiana.

Os diagnósticos estrutural e funcional são definidos pelo ECG, ECO transtorácico, ressonância magnética e teste cardiopulmonar.

A classificação da OMS tem sido muito bem aceita como um parâmetro para avaliação do risco materno-fetal de acordo com a lesão cardíaca estrutural. Acrescentem-se a essa classificação as condições clínicas previstas na história natural das CC (que modificam o prognóstico da gravidez e independem da lesão cardíaca estrutural (Tabela 24).

**Tabela 24:** – Fatores associados ao prognóstico materno em portadoras de CC

Hipertensão arterial pulmonar
Cianose
Lesões obstrutivas graves
Disfunção ventricular
Necessidade de anticoagulação permanente
Pacientes sintomáticas com indicação de intervenção da cardiopatia

**Síndrome de Eisenmenger:** considerada de elevado risco para morte materna, estimado em 50% durante a gravidez e puerpério.^167^ A doença vascular arterial restringe a adaptação às variações do débito cardíaco e à queda da resistência vascular periférica durante a gravidez, o parto e o puerpério. Disso decorrem as principais complicações que causam a morte, tais como IC, crises de hipóxia e arritmias. O risco do puerpério é tão alto quanto o da gestação, devido à hemorragia do pós-parto e ao tromboembolismo inerente a este período.^168^ Pacientes com síndrome de Eisenmenger parecem ter predisposição para trombocitopenia, deficiência de fatores de coagulação vitamina K-dependentes e sangramento. O risco para o feto de abortos espontâneos, prematuridade e mortalidade perinatal é proporcional ao grau de cianose.

**Cianose:** estima-se que 30% das pacientes com CC cianogênicas, submetidas ou não a cirurgias, apresentam complicações durante a gestação, destacando-se: IC, trombose sistêmico-pulmonar, arritmias e hipoxemia. O grau de saturação de oxigênio arterial é fator de prognóstico para a sobrevida materno-fetal, e a insaturação tem correlação significativa a morte materna, aborto espontâneo e morte perinatal.^169^ A indicação de flebotomia na eritrocitose materna, tem sido realizada somente quando o hematócrito está acima de 65%, em pacientes com sintomas de cefaléia, fadiga, distúrbio visual ou cognitivo e mialgia. A evolução do concepto, que inclui perdas fetais, prematuridade e morte perinatal, está relacionada ao grau de saturação arterial de oxigênio (SaO_2_). Estima-se que saturação de oxigênio < 85% associa-se a apenas 12% de recém-nascidos vivos.^170^

**IC:** a dispneia é um parâmetro clínico utilizado no auxílio de condutas, entretanto, tem limitações quando aplicado à gravidez. Em CC deve-se considerar o baixo fluxo pulmonar e as lesões que envolvem o coração esquerdo.^52^

**Arritmias cardíacas:** frequentes em adultos com CC, as arritmias são resultantes de sequelas de defeitos cardíacos como dilatação de câmaras cardíacas, hipertrofia miocárdica, fibrose ou cicatrizes cirúrgicas, trauma do tecido de condução e presença de enxertos endocárdicos.

**Intervenção prévia à gravidez:** a correção cirúrgica ou percutânea das CC está associada a melhor prognóstico materno-fetal em comparação com pacientes cujas cardiopatias não foram operadas. Nesse aspecto, a eventual necessidade de intervenção deve ser levada em conta antes da concepção.

### 3.2.1. Conduta na Gestação

Em continuidade com a avaliação da pré-concepção, a consulta inicial do pré-natal deve incluir (1) história; (2) tipo de cirurgia corretiva ou paliativa, (3) evolução pós-operatória imediata e tardia; (4) situação clínica funcional atual e (5) exames periódicos laboratoriais (hematócrito, hemoglobina, saturação de oxigênio, peptídeo natriurético).^171^

A assistência durante a gestação, parto e puerpério de pacientes com CC deve contar com equipe de especialistas, hospital terciário e consultas periódicas e simultâneas com a equipe obstétrica. Vale lembrar que a hereditariedade das CC impõe a rotina de ECO fetal a partir do segundo trimestre da gestação.^171^

Gestantes que se incluem no risco III/IV-OMS devem ser orientadas quanto à rotina de hospitalização a partir da 28ª a 32ª semana de gestação, para estabilização do quadro clínico, monitoramento contínuo fetal, ajuste de medicação e planejamento do parto. As decisões quanto ao término da gestação, o tipo de parto e a anestesia devem ser conjuntas, de acordo com a situação clínica da mãe, a vitalidade e a maturidade do feto.

**Cardiopatias congênitas associada à HP:** A interrupção no 1º trimestre da gestação deve ser recomendada para mulheres com HP e síndrome de Eisenmenger (Risco IV-OMS). Entretanto, perante a decisão da paciente em manter a gravidez, a equipe multidisciplinar deve obedecer ao protocolo que orienta a hospitalização após a 28ª semana de gestação, uso de enoxaeparina (HBPM) em dose profilática (1 mg/kg/dia) e oxigenoterapia (suplementação de oxigênio para saturação abaixo de 92%), medidas essenciais para controlar hipotensão, hipoxemia e acidose metabólica.^172^

Vasodilatadores específicos, como os inibidores de fosfodiesterase (sildenafil), podem levar à hipotensão arterial e devem ser indicados individualmente de acordo com o quadro clínico e a tolerância materna. O sildenafil ou outros inibidores da fosfodiesterase têm sido utilizados, bem como a eventual adição das prostaglandinas na persistência dos sintomas.^173,174^ Os antagonitas dos receptores da endotelina devem ser suspensos durante a gravidez.^175,176^

A HBPM em dose plena ou profilática deve substituir a varfarina no primeiro trimestre da gravidez e após 36 semanas, conduta recomendada em pacientes que já faziam uso desse fármaco antes da concepção (Figura 6). A prescrição de antiplaquetários (como aspirina) ou a HBPM deve ser realizada com muita cautela, porque pacientes com HP apresentam alto risco de hemoptise e trombocitopenia.

**CC com lesão estrutural obstrutiva:** pacientes com obstrução grave a via de saída de ventrículo esquerdo devem ser aconselhadas à correção cirúrgica ou percutânea antes da gestação. Contudo, se a paciente estiver grávida, a presença da tríade de sintomas (IC, angina de peito e síncope) é indicativa de intervenção durante a gestação^171^ Nesses casos, a valvoplastia percutânea com balão está indicada, sendo mais segura no segundo trimestre da gravidez, quando a fase de embriogênese foi ultrapassada, a tireoide fetal ainda é inativa e o útero tem volume pequeno, o que proporciona maior distância entre a radiação ionizante do procedimento e o concepto.

**Cardiopatia cianogênica sem HP:** as medidas gerais incluem restrição da atividade física, suplementação de oxigênio e prevenção da estase venosa pelo admitido risco de embolia paradoxal. O uso da HBPM em doses profiláticas é recomendado porque o tromboembolismo é uma das principais complicações. A suplementação de ferro pode ser utilizada em função da policitemia, à semelhança da síndrome de Eisenmenger.^175,176^

**Cardiopatia com comunicação sem HP:** a comunicação interatrial (CIA) é bem tolerada durante a gravidez, considerada risco I-OMS.^177^ Arritmias, geralmente supraventriculares, são comuns nas pacientes não operadas ou com correção cirúrgica na idade adulta e podem ser controladas com digital (digoxina), betabloqueador (propranolol ou metoprolol) ou antagonista dos canais de cálcio (verapamil), em doses baixas e fracionadas. Deve-se considerar que paciente com CIA não corrigida apresenta risco de tromboembolismo, o que pode sugerir o uso de HBPM. Embora não seja habitual, pacientes sintomáticas que apresentam fluxo esquerdo-direito e instabilidade hemodinâmica podem ser beneficiadas com o fechamento percutâneo dessa comunicação antes da gestação.

A comunicação interventricular (CIV) pequena ou operada tolera bem a gravidez considerada risco I-OMS, especialmente quando a função ventricular é normal.

A evolução dos defeitos de septo atrioventricular ou canal atrioventricular (DSAV) não corrigidos depende da magnitude da regurgitação valvar e do tamanho da comunicação entre as câmaras, o que é considerado risco II (OMS). As complicações mais frequentes são: arritmias, congestão pulmonar e IC em pacientes com disfunção ventricular. O tratamento inclui: digital (digoxina), diurético (furosemida), vasodilatador (hidralazina) ou betabloqueador (carvedilol).

**Coarctação da aorta:** a gravidez é bem tolerada em pacientes com coarctação aórtica corrigida, considerada risco II-OMS. Contudo, quando não há correção prévia à concepção, há complicações associadas que determinam alto risco para a gravidez, como: HAS com risco adicional de pré-eclâmpsia, aneurisma de aorta, dissecção aórtica e rotura de aneurisma cerebral, passando para risco IV-OMS.^178,179^ O controle da pressão arterial é fundamental, utilizando-se a terapêutica convencional.

**Tetralogia de Fallot:** é a cardiopatia cianogênica mais frequente do adulto, e as pacientes operadas toleram bem a gravidez. Nesse grupo, os fatores de risco são a disfunção ventricular direita e a insuficiência pulmonar, que, na maioria, tem adaptação adequada à gravidez.^179^ Nesse aspecto, a conduta atual de substituição da valva pulmonar na população de pacientes com dilatação importante do ventrículo direito tem contribuído para um contingente cada vez maior de gestantes portadoras de próteses biológicas em valva pulmonar. As arritmias cardíacas que eventualmente fazem parte da história natural do pós-operatório tardio não comprometem o resultado obstétrico e fetal.^180^ A experiência com tetralogia de Fallot não operada é muito limitada e deve seguir as recomendações das cardiopatias cianogênicas.

**Anomalia de Ebstein:** o prognóstico dessas gestantes está relacionado à presença ou não de cianose e IC. A instabilidade hemodinâmica está associada a insuficiência tricúspide ou disfunção do ventrículo direito. A gravidez deve ser planejada após a correção cirúrgica em pacientes sintomáticas, com IC ou cianose. É comum a síndrome de pré-excitação associada a anomalia, e arritmias podem ser um fator de complicação durante a gestação, mesmo em pacientes operadas.^181^

**Transposição das grandes artérias (TGA):** na dextro TGA, a evolução tardia após o “switch” atrial (técnicas de Senning ou Mustard) ou arterial (cirurgia de Jatene) tem sido boa, incluindo a tolerância à gravidez.^182^ A presença de disfunção ventricular direita ou insuficiência tricúspide importante é um fator de mau prognóstico e de restrição à gravidez.^175^A conduta no tratamento das complicações deve seguir as recomendações convencionais. Na levo TGA, também conhecida como discordância ventrículo-arterial e atrioventricular ou inversão ventricular, a evolução da gravidez depende da classe funcional, da função do ventrículo direito sistêmico, da presença de arritmias e das lesões associadas.^183^ No adulto, a presença de disfunção ventricular é um fator de restrição ao prognóstico materno-fetal.^184^Para estas mulheres, a gravidez deve ser desaconselhada.

**Circulação de Fontan:** gestações sucedidas têm sido reportadas em pacientes submetidas à cirurgia de Fontan, embora seja considerada risco III- OMS. As complicações presumíveis são consequentes ao baixo débito cardíaco, arritmias, tromboembolismo e à doença hepática.^185^ Desaconselha-se a gravidez para pacientes com StO_2_ menor que 85%, insuficiência atrioventricular grave, depressão da função ventricular ou perda proteica entérica, condições que inclui a circulação de Fontan no risco IV-OMS. A conduta é o tratamento e a prevenção da IC, das arritmias e do tromboembolismo. A evolução obstétrica e fetal de pacientes com Fontan é incerta e complicada devido à alta incidência de aborto espontâneo, prematuridade, recém-nascidos pequenos para a idade gestacional e óbito neonatal. Acresce o alto risco de HPP, peculiar a essa situação clínica.^185,186^

**Hereditariedade:** filhos de mães portadoras de cardiopatia congênitas têm maior risco de apresentar lesões cardíacas congênitas, as quais variam de acordo com o tipo de defeito materno e não são, necessariamente, iguais à lesão estrutural da mãe. A recorrência da CC é detectada pelo ECO fetal, sendo em torno de 2,7 a 10% dos casos.^187^ Verifica-se ainda que há síndromes genéticas associadas a defeitos específicos, tais como CIA na síndrome de Holt-Oram, anomalia conotruncal na síndrome de DiGeorge, dentre outras, que são transmissíveis. Esses dados reforçam a recomendação do ECO fetal na rotina do pré-natal nesse grupo de portadoras de CC hereditárias.

### 3.2.2. Pontos-chaves

- O planejamento da gravidez requer a determinação do diagnóstico estrutural e funcional da cardiopatia com base em exames laboratoriais e de imagem;
- O aconselhamento pré-concepção deve ser fundamentado na classificação de risco da OMS;
- A presença de HP, cianose, arritmias, disfunção ventricular, antecedentes tromboembólicos ou de IC acrescenta risco às categorias da OMS;
- Intervenção cirúrgica ou percutânea, quando indicada, deve ser realizada antes da concepção;
- Gestantes de risco III/IV-OMS devem ser encaminhadas para atendimento especializado em centros terciários com apoio do Heart Team;
- A hereditariedade das CC exige a realização do ECO fetal e o aconselhamento genético na pré-concepção.

## 3.3. Cardiomiopatias

Cardiomiopatia é uma doença do músculo cardíaco com comprometimento estrutural e funcional do coração, na ausência de doença arterial coronariana, hipertensão arterial, doença valvar ou cardiopatia congênita, que justifique a anormalidade miocárdica observada. De acordo com o fenótipo, as cardiomiopatias são classificadas em: hipertrófica, dilatada, restritiva, arritmogênica do ventrículo direito e não compactada.^188^Essa classificação é fundamental para a avaliação de riscos, a determinação de conduta e a estimativa de prognóstico da gestação, independentemente da provável etiologia. A CMPP será discutida adiante.

Um estudo retrospectivo sobre cardiomiopatias durante a gestação mostrou um percentual de 35% de complicações com 11% de mortalidade materna, dados que se relacionaram com a maneira e o grau de comprometimento miocárdico.^189,190^ A classificação de risco III/IV-OMS inclui as cardiomiopatias com fração de ejeção do ventrículo esquerdo reduzida (FEVE) abaixo de 30%, com manifestação de IC, HP e arritmias complexas.^52^

A IC é a principal complicação, principalmente após o segundo trimestre de gestação e durante o trabalho de parto. No puerpério imediato, momento tão delicado quanto a gestação, devem-se pressupor as seguintes recomendações: cautela com o uso de ocitócicos, moderação na infusão de fluidos durante o intraparto, atenção a HPP, controle da dor, prevenção de infecção e retaguarda de UTI nas primeiras 24 a 48 h após o parto.

### 3.3.1. Cardiomiopatia Dilatada

Cerca de 50% dos casos de cardiomiopatia dilatada (CMD) são idiopáticos, 20 a 35% são hereditários e, em quase 40%, têm sido identificadas mutações genéticas.^191^ Dentre as causas adquiridas, destacam-se: miocardites de etiologia viral (*Coxsackie virus*, parvovírus, ecovírus, adenovírus), H1N1, Epstein-Barr e outras relacionadas ao uso de fármacos.

No planejamento familiar, na intenção de engravidar, devem ser recomendados: (1) ajustes na terapêutica materna no controle da IC, considerando-se que fármacos essenciais que são contraindicados na gravidez (IECA, BRA, inibidores da Neprisilina, espirolactona, ivabradina) devem ser substituídos; (2) conscientização da paciente sobre o possível impacto imediato e a longo prazo da gravidez na doença cardíaca; (3) aconselhamento genético, em função de a doença estar associada à herança autossômica dominante, seguida da autossômica recessiva e às ligadas ao cromossomo X.^191^

### 3.3.2. Cardiomiopatia Hipertrófica

A prevalência global de cardiomiopatia hipertrófica (CMH) está em torno de 0,02 a 0,2% da população^192^ e foi estimada em 0,015% em um estudo de coorte com gestantes portadoras de cardiopatia.^145^ A gravidez constitui um risco potencial para mulheres com CMH; entretanto, o seu prognóstico ainda é incerto.

A grande variação da taxa de complicações cardiovasculares durante a gravidez, estimada entre 5 e 40%, tem sido atribuída à heterogeneidade do fenótipo dessa doença cardíaca.^193-195^Embora muitas vezes assintomáticas, as queixas mais frequentes das gestantes portadoras de CMH são dor torácica, dispneia, síncope e palpitação. Os fatores de pior prognóstico para a gravidez são história de IC, arritmia ventricular e morte súbita na família. As complicações durante a gravidez são decorrentes de obstrução da via de saída do ventrículo esquerdo, disfunção diastólica e isquemia miocárdica.

Dentre as arritmias mais frequentes, destacam-se extrassístoles atriais, taquiarritmias supraventriculares sustentadas e FA, que favorecem a instabilidade hemodinâmica materna. Do ponto de vista obstétrico, as complicações mais frequentes são aborto espontâneo em cerca de 20% dos casos e baixo peso ao nascimento em 10% das gestações.^193-195^

Outra importante questão é o risco de transmissão da doença para o feto, porque a CMH é um traço autossômico dominante mendeliano causado também por mutações que codificam os componentes do sarcômero cardíaco.^196^ A complexidade dessa doença ainda não permitiu a determinação da sua verdadeira incidência em recém-nascidos aparentemente saudáveis e que não apresentam anormalidades ao ECO convencional. Na maioria dos casos, o ECO no período neonatal não identifica a CMH porque a hipertrofia miocárdica ocorre com o desenvolvimento e se torna aparente após a adolescência. Contudo, destaca-se que a forma obstrutiva da doença e uma história familiar de morte cardíaca súbita (MCS) são os fatores de risco para a manifestação de hipertrofia precoce na criança.^131,197^

O estudo genético de crianças e adolescentes assintomáticos com antecedentes familiares de CMH pode identificar portadores “sãos” da mutação. No entanto, existem importantes obstáculos à aplicação clínica dessa investigação, tais como pluralidade genética, baixa frequência da mutação responsável na população doente, dificuldades das técnicas na identificação da mutação patogênica e alto custo.

Em pacientes sintomáticas, a conduta farmacológica inicial é o uso de betabloqueadores (propranolol ou succinato de metoprolol), associados ou não aos antagonistas de cálcio, como o verapamil.^52^A associação desses fármacos exige cuidados quanto a tolerância materna, pressão arterial e vitalidade fetal. O uso de prostaglandinas para a indução do parto não é aconselhado devido aos seus efeitos vasodilatadores. O parto vaginal é considerado seguro, enquanto a cesárea é reservada em situações especiais. A anestesia peridural ou raquidiana deve ser contraindicada nas formas obstrutivas graves.

No planejamento de gravidez em pacientes com arritmias de difícil controle farmacológico, deve-se considerar a discussão com o eletrofisiologista sobre a possibilidade de intervenção percutânea, como a ablação por radiofrequência nos casos de taquicardias complexas e/ou sintomáticas, ou mesmo o CDI em pacientes incluídas nas recomendações convencionais de classe IA de indicação.

### 3.3.3. Displasia Arritmogênica do Ventrículo Direito

A displasia arritmogênica do ventrículo direito é uma cardiomiopatia hereditária, autossômica dominante, com penetrância reduzida e expressão variável. Por esses motivos, é obrigatório o aconselhamento genético.

A gravidez é bem tolerada na maioria das mulheres com a doença, mas pacientes com doença biventricular preexistente têm maior risco de desenvolver IC à medida que a gravidez progride.^198^ O controle e a prevenção dos sintomas são feitos com os betabloqueadores (propranolol, succinato de metoprolol). Caso seja necessário, os antiarrítmicos devem ser mantidos, respeitando as retrições da toxicidade fetal. O implante do CDI, se indicado, deve ser preferencialmente realizado antes da gestação.^199^

### 3.3.4. Cardiomiopatia Não Compactada

O miocárdio não compactado caracteriza-se por uma morfologia acentuadamente trabeculada do miocárdio. É uma doença familiar em até 60% dos casos, com herança autossômica dominante. Sua prevalência é desconhecida, e as evidências são limitadas na literatura sobre a conduta durante a gravidez.^200^ O quadro clínico é muito variável, desde pacientes assintomáticas até mulheres com IC refratária e arritmias graves. Não há tratamento específico para a cardiomiopatia não compactada, e a conduta terapêutica deve ser apoiada na experiência das outras cardiomiopatias. No entanto, o risco de tromboembolismo é considerado maior pela própria morfologia do miocárdio, o que justifica a anticoagulação permanente durante a gestação em pacientes sintomáticas ou com disfunção ventricular.

### 3.3.5. Cardiomiopatia Restritiva

A cardiomiopatia restritiva idiopática caracteriza-se por apresentar ventrículos não hipertróficos, não dilatados e com disfunção diastólica, resultando na dilatação dos átrios. Pode ser idiopática ou estar associada a outras doenças, como amiloidose, endomiocardiofibrose, sarcoidose e hemocromatose. A escassez de experiência na literatura, a terapêutica limitada e controversa, e a evolução clínica muitas vezes grave são fatores que desaconselham a gravidez.

### 3.3.6. Pontos-chaves e Recomendações

- Portadoras de cardiomiopatia devem participar de planejamento familiar, incluindo o aconselhamento genético;
- A estratificação de risco para “nova” gravidez deve considerar o estado funcional e estrutural da cardiomiopatia;
- Filhos de portadoras de CMH, mesmo que aparentemente saudáveis, devem ter um seguimento diferenciado até a adolescência;
- A otimização terapêutica deve obedecer às diretrizes convencionais, considerando as clássicas contraindicações dos fármacos para a gestação;
- A anticoagulação permanente deve ser instituída nas gestantes portadoras de cardiomiopatia não compactada ou dilatadas com trombo intracavitário ou evento embólico prévio;
- Estudos genéticos são promissores para as mudanças no prognóstico das cardiomiopatias.

### 3.3.7. Cardiomiopatia Periparto

A CMPP é definida como uma forma idiopática de cardiomiopatia que se manifesta com IC secundária à disfunção sistólica do ventrículo esquerdo, com FEVE (< 45%), que ocorre no final da gravidez ou em meses que transcorrem após o parto ou abortamento, quando nenhuma outra causa de IC tenha sido encontrada.^201^

A fisiopatologia da CMPP, ainda não totalmente esclarecida, apoia-se em hipóteses que sugerem mecanismos hormonais, inflamatórios, autoimunes, infecciosos, genéticos e ambientais.^201^ Novos conceitos sobre a etiopatogenia têm sido apresentados, envolvendo o estresse oxidativo, o desequilíbrio angiogênico e a prolactina na gênese da CMPP.^202,203^

Os estudos têm demonstrado que o aumento do estresse oxidativo da gravidez^204^ em combinação com níveis elevados de fatores antiangiogênicos, menor expressão de reguladores da angiogênese e aumento de espécies reativas de oxigênio propiciam a clivagem da prolactina, pela catepsina D, formando vasoinibinas como a 16kDa prolactina. Ela tem propriedades antiangiogênicas, pró-apoptóticas e pró-inflamatórias que induzem no endotélio a expressão de micropartículas (miR-146a), as quais, no miocárdio, alteram o metabolismo dos cardiomiócitos, ocasionando a morte celular e a disfunção deles.^205,206^ O miR-146a é um marcador altamente específico no diagnóstico da CMPP, considerado o primeiro biomarcador específico da CMPP.^207^ Nesse sentido, a bromocriptina, agonista do receptor de dopamina-D2, ou a cabergolina, potente e prolongada atividade redutora de prolactina, vem apresentando resultados promissores na terapêutica e na recuperação da função ventricular.^207-211^

Os principais fatores de risco para a CMPP são as síndromes hipertensivas da gestação^212^ (hipertensão gestacional, pré-eclâmpsia, eclâmpsia ou síndrome HELLP), hipertensão arterial crônica, gestações múltiplas, obesidade, tabagismo, pré-diabetes e diabetes melito, idade avançada ou adolescência e uso prolongado de beta-agonistas.^213^

As taxas de mortalidade podem ser inferiores a 5% ou alcançar até 50% dos casos. As causas de morte materna são IC, arritmia ventricular e tromboembolismo, que ocorrem principalmente nos primeiros 6 meses da doença até 1 ano pós-parto (morte materna tardia), o que pode causar a subnotificação da doença.^214,215^

O quadro clínico compreende dispneia importante, edema agudo dos pulmões ou choque cardiogênico. Não é rara a ocorrência de parada cardíaca, arritmia grave ou eventos tromboembólicos (acidente vascular encefálico, isquemia mesentérica ou infarto agudo do miocárdio [IAM]) e choque cardiogênico como primeira manifestação da doença.^216^

O diagnóstico de CMPP deve ser sempre considerado quando ocorrer descompensação cardíaca nas semanas próximas ao termo da gestação ou meses subsequentes após o parto em mulheres previamente saudáveis.^201^ O diagnóstico da CMPP é de exclusão e deve ser diferenciado da miocardite, infarto agudo do miocárdio, tromboembolismo pulmonar (TEP), pré-eclâmpsia grave, embolia amniótica, cardiomiopatias preexistentes, síndrome de Takotsubo, doença valvar ou congênita preexistente e infecções sistêmicas. A não valorização de sintomas não muito específicos, como cansaço aos esforços , dor no peito ou fadiga, que costumam ocorrer no final da gravidez e no pós-parto, contribuem para o retardo no diagnóstico da CMPP e consequentemente pior prognóstico e menor chance de recuperação da função sistólica do miocárdio.^201,215,217,222^

Os exames complementares incluem:^201^

- ECG: na maioria dos casos apresenta alterações inespecíficas na repolarização ventricular, na taquicardia sinusal ou nas arritmias ventriculares. É preciso lembrar que o ECG normal não exclui o diagnóstico da doença;
- Radiografia de tórax: as alterações mais frequentes são: cardiomegalia, sinais de edema alveolar, redistribuição do fluxo sanguíneo para os ápices pulmonares e imagem de “asa de borboleta”;
- Biomarcadores: peptídeos natriuréticos (peptídeo natriurético tipo B [BNP] e/ou N-terminal-pro-BNP) são marcadores válidos na investigação da IC, porque, quando elevados, ajudam a estabelecer o diagnóstico e, quando normais, excluem o diagnóstico de IC na gravidez;
- Os valores de referência para o diagnóstico de IC são: N-terminal-pro-BNP > 300 pg/ml e BNP > 100 pg/ml;
- Concentrações de troponina sérica têm valor preditivo de persistência de disfunção ventricular após seis meses da doença instalada;
- Ecodoplercardiograma transtorácico é o exame “padrão ouro” para o diagnóstico de CMPP. As imagens demonstram predominância de hipocinesia do ventrículo esquerdo, com FEVE inferior a 45%, regurgitação das valvas atrioventriculares e derrame pericárdico. A FEVE abaixo de 30% e o diâmetro diastólico final do ventriculo esquerdo > 60 mm são correlacionados a pior prognóstico materno;
- RMC: fornece informações sobre o grau de acometimento miocárdico e deve ser considerada para a estimativa de prognóstico e tratamento na evolução tardia da doença;
- Cinecoronariografia/biópsia do miocárdio: não apresentam indicação para o diagnóstico da CMPP.

O momento do diagnóstico o PPCM é crucial para a sobrevivência da paciente. Os objetivos imediatos no tratamento agudo são estabilizar o estado hemodinâmico, proporcionar alívio sintomático e garantir o bem-estar materno e fetal. Os médicos devem conhecer o diagnóstico diferencial de dispneia em sala de emergência relacionado à gravidez e atuar no diagnóstico precoce. Os cuidados devem ser prestados por uma equipe multidisciplinar, incluindo cardiologistas, intensivistas, obstetras, neonatologistas, anestesistas e cirurgiões. Para diagnóstico rápido e tomada de decisão em todas as gestantes com insuficiência cardíaca aguda, é fundamental um algoritmo de gerenciamento pré-especificado e a composição da equipe multidisciplinar.^221,222^

O tratamento farmacológico da CMPP^218,219^ acompanha as diretrizes convencionais de IC com fração de ejeção ecocardiográfica (FE) reduzida. Os betabloqueadores, de preferência β1-seletivos (carvedilol, bisoprolol e metoprolol), são indicados em doses inicialmente baixas, associados ao diuréticos de alça; a digoxina pode ser considerada no controle da frequência cardíaca. O uso de anticoagulante, seja a heparina em dose plena ou a varfarina com meta de INR = 2, é indicado, uma vez que o tromboembolismo é causa de morte. Deve-se reforçar que IECA, BRA, sacubitril/valsartan, ivabradina, espirolactona e varfarina são contraindicados na gestação, mas devem ser considerados na lactação. Recomenda-se anticoagulação com heparina para evitar complicações cardio-embólicas em pacientes com FEVE ≤ 35% com HBPM ou anticoagulação oral pelo menos em dose profilática.

O uso de bromocriptina (alcaloide do ergot) e cabergolina (agonista do receptor da dopamina D2) mostrou resultados satisfatórios na resposta imediata e recuperação tardia da disfunção ventricular da CMPP.^208-211^ A eventual contra-indicação ao uso desses medicamentos também deve ser ponderada. Se a bromocriptina não estiver disponível, a cabergolina pode ser usada como um medicamento alternativo. Como foram relatados eventos tromboembólicos durante o uso de bromocriptina (embora principalmente em doses mais altas), o tratamento com bromocriptina deve sempre ser acompanhado de anticoagulação, pelo menos nas doses profiláticas de heparina; doses completas de heparina (fracionada/não fracionada) são obrigatórias na presença de trombo intracardíaco ou embolia sistêmica, bem como em FA paroxística ou persistente.^222^O esquema proposto mostra que as doses seguras que apresentam boa tolerância e eficácia são de 2,5 mg, 2 vezes ao dia, por 2 semanas, seguidas de 2,5 mg, 1 vez ao dia, durante 6 semanas para a bromocriptina; e de 1 mg em dose única para a cabergolina, pelo seu efeito prolongado de 14 a 21 dias.^218-219^ A abreviatura **BOARD** (Bromocriptina, Otimização da terapêutica da IC, Anticoagulação, Vaso Relaxadores e Diuréticos) foi proposta para o tratamento na PPCM após o parto.^218^

O tratamento com bromocriptina deve sempre ser acompanhado de anticoagulação com heparina (HBPM ou HNF), pelo menos em dose profiláticas; em pacientes com trombo intracardíaco detectado por imagem ou evidência de embolia sistêmica, bem como em pacientes com fibrilação atrial paroxística ou persistente.^222^

Em relação ao tratamento não farmacológico de PPCM, CDI, ressincronização cardíaca, dispositivos de assistência ventricular e transplante cardíaco são considerados.^220-222^ O CDI foi considerado na prevenção primária de morte súbita, seguindo diretrizes para pacientes com FE ventricular abaixo de 35%. O desfibrilador cardioversor tipo colete (wearable) é uma alternativa durante os primeiros meses após o diagnóstico com PPCM, considerando que a maioria desses pacientes recupera a função ventricular 6 meses após a fase aguda da doença.

A ressincronização cardíaca pode ser proposta após 6 meses do início da doença, de acordo com critérios convencionais de indicação, ou seja, IC avançada, CF III-IV (NYHA), com tratamento otimizado, ritmo sinusal, FE inferior a 35%, QRS > 150 mseg ou QRS > 120 mseg com dissincronismo Não ECO ou na ressonância magnética

Os dispositivos de assistência circulatória mecânica do ventrículo esquerdo podem ser uma opção nas pacientes criticamente graves, como “ponte para transplante” ou como “ponte para recuperação”. O transplante cardíaco é indicado em cerca de 10% dos casos de CMPP nas pacientes que não apresentam recuperação após 12 meses com suporte circulatório mecânico (SCM).

No seguimento clínico a longo prazo, deve-se seguir as recomendações adiante:^221^

1. Se não houve melhora da função cardíaca: manter betabloqueador, IECA ou BRA; espirolactona se FE < 40%, ivabradina se FC > 75/min, com dose máxima de betabloqueador (alcançar FC < 60/min); diuréticos de houver edema/congestão pulmonar;
2. Se houve recuperação completa e sustentada da função ventricular, informação apoiada no acompanhamento ecocardiográfico semestral: manter tratamento farmacológico (betabloqueador, IECA, espirolactona) por pelo menos 6 meses e diuréticos somente se houver sintomas de congestão ou edema de membros inferiores; no período entre 6 e 12 meses subsequentes, suspender a espirolactona e a ivabradina (se estiver em uso), mas continuar betabloqueador e IECA/BRA por pelo menos mais 6 meses depois da retirada da espirolactona; após 12 meses, reduzir gradativamente até suspender IECA/BRA e manter betabloqueador por mais 6 meses; após 18 meses, é controversa a suspensão do betabloqueador porque alguns admitem que ele deve ser mantido por no mínimo 5 anos;
3. O não aconselhamento a uma gravidez subsequente em pacientes que tiveram a recuperação completa da função sistólica de ventrículo esquerdo (VE) após CMPP é controverso, já que não existem evidências concludentes que possam apoiar tal orientação na prática médica.^218^

As recomendações para a conduta da IC agudam^208,222^ podem ser resumidas nos seguintes itens:

1. Monitoramento da saturação de O_2_ transcutânea;
2. Oxigenoterapia: SatO_2_ < 90% (oximetria de pulso); pressão arterial de O_2_ (PaO_2_) < 60 mmHg (gasometria arterial);
3. Intubação endotraqueal: deve ser realizada na insuficiência respiratória aguda com hipoxemia (PaO_2_ < 60 mmHg), hipercapnia (pressão arterial de gás carbônico [PaCO_2_] > 50 mmHg) e acidose (pH < 7,35);
4. Diurético se sinais de congestão (furosemida (20 a 40 mg) em bólus intermitentes ou em infusão contínua;
5. Vasodilatador se PAS > 110 mmHg. Nitroglicerina intravenosa (IV), dose inicial de 10 a 20 μg/min até o máximo de 200 μg/min;
6. Agentes inotrópicos (inibidores da dobutamina, dopamina, levosimendan, fosfodiesterase III (PDE III) nas pacientes hipotensas (PAS < 90 mmHg) e/ou sinais de baixo débito cardíaco; 6. Evidências experimentais e experiência clínica sugerem que catecolaminas como dobutamina são menos favoráveis em pacientes com PPCM devido a comprometimento metabólico. O levosimendan pode ser considerado como agente inotrópico de preferência, em infusão contínua de 0,1 µg/kg/h por 24 h sem uma dose inicial de carga (bolus) para pacientes com PPCM grave. Caso o levosimendan não esteja disponível, a dobutamina é a outra opção, enquanto a adrenalina deve ser evitada;
7. Vasopressores no choque cardiogênico;
8. Anticoagulação com HBPM em dose plena desde que não haja contraindicação;
9. Suporte mecânico circulatório como “ponte para decisão” de transplante cardíaco.

***3.3.7.1. Pontos-chaves***

- A etiopatogenia da CMPP ainda não está plenamente esclarecida;
- O diagnóstico e tratamento imediatos ao início dos sintomas são fundamentais para a recuperação ventricular;
- O emprego dos inibidores da prolactina (bromocriptina ou cabergolina) junto ao tratamento otimizado para IC é o diferencial na recuperação da função ventricular;
- Aproximadamente 50% dos pacientes com CMPP recuperam a função miocárdica em um prazo médio de 6 meses com terapêutica da IC;
- Ainda que haja recuperação da função ventricular, o seguimento deve ser periódico pelo menos por 5 ou quiçá 10 anos após o diagnóstico;
- A falta de evidências sobre a real recorrência da CMPP em gestações subsequentes não autoriza desaconselhar a concepção em pacientes que de fato recuperaram a função ventricular;
- Pacientes com CMPP transplantadas têm um prognóstico pós-operatório imediato e tardio semelhante ao das portadoras de outras formas de CMD.

## 3.4. Cardiopatia Isquêmica

A doença cardíaca isquêmica (DCI) é incomum durante a gravidez; a maioria das publicações considera a síndrome coronariana aguda, e não a doença isquêmica estável.^223^ Dados da OMS mostram que a taxa de infarto agudo é de 3,34 eventos por 100.000 gestações, sendo mais frequente durante o terceiro trimestre.^224^ A incidência de infarto sem supradesnivelamento do segmento ST é maior na gestação.^52^

Os fatores de risco para a DCI na gestação são: idade materna (acima dos 40 anos; para cada ano de vida há aumento de 20% de risco para infarto), história familiar de doença coronária prematura, tabagismo, hipertensão arterial, dislipidemia e diabetes melito.^52^

Fatores de risco adicionais incluem pré-eclâmpsia, trombofilia, infecção pós-parto, uso de cocaína, multiparidade, doenças autoimunes, estenose valvar aórtica/trombose de prótese valvar aórtica, estenose mitral e HP.^52^

A etiologia da DCI na gestação difere da população geral. Em uma revisão^225^contemporânea, foram identificados os mecanismos relacionados ao infarto e as seguintes incidências: dissecção espontânea da artéria coronária (43%), aterosclerose (27%), trombose coronária (17%), artérias normais à angiografia (9%), vasoespasmo (2%) e Takotsubo (2%).

**Dissecção espontânea da artéria coronária:** é a causa mais comum de IAM durante a gestação e o puerpério, com prevalência ao redor de 1,81 evento por 100.000 gestações, de ocorrência mais frequente no terceiro trimestre. O desfecho da dissecção associada à gravidez parece ter pior prognóstico quando comparado àquela não relacionada à gravidez.^226^

As condições demográficas e as comorbidades associadas incluem raça negra, hipertensão crônica, hipertensão gestacional, pré-eclâmpsia, anormalidades lipídicas, depressão crônica, enxaqueca, idade materna avançada, primeiro parto e tratamento para infertilidade.^226^

A etiologia da dissecção de coronária ainda não está esclarecida, mas parece estar relacionada ao desarranjo e enfraquecimento da parede arterial, consequente à influência dos hormônios da gestação. As complicações maternas mais comuns descritas são: choque cardiogênico em 24%, fibrilação ventricular (FV) em 16% e suporte mecânico em 28%, que resultam em 4% de morte hospitalar.^226^

**Aterosclerose:** a DCI de causa aterosclerótica está ligada à presença de fatores de risco clássicos e os atualmente denominados emergentes: doença hipertensiva da gestação, diabetes gestacional, histórico de parto prematuro, doenças autoimunes (Lupus eritematoso, artrite reumatoide, esclerodermia), tratamento com radioterapia no tórax/quimioterapia e depressão/ansiedade generalizada.^227^

**Trombose:** trombose coronariana na ausência de aterosclerose é mais provável devido à hipercoagulabilidade da gravidez e pode resultar de embolização paradoxal.

**Artérias normais:** mecanismos do IAM com coronárias normais permanecem incertos e incluem espasmo coronário transitório (aumento da reatividade vascular e/ou uso de derivados da ergotamina) ou dissecção coronária não detectada, refletindo as limitações do diagnóstico.^52^

**Vasoespasmo:** pode ser espontâneo ou induzido por fármacos, síndromes hipertensivas da gravidez, aumento da reatividade vascular à angiotensina II e à norepinefrina, disfunção endotelial ou liberação de renina pelo útero gravídico. O vasoespasmo pode ser induzido por fármacos da rotina obstétrica, como os beta-agonistas (terbutalina, salbutamol) na inibição do trabalho de parto prematuro, os derivados do ergot para indução do trabalho de parto ou prevenção da HPP e a bromocriptina, indicada para inibição da lactação.^227^

**Outras causas**: aneurisma de artéria coronária relacionado à doença de Kawasaki.^52^

O diagnóstico do IAM não é influenciado pelo estado gravídico e inclui sintomas (dispneia e dor torácica), exames laboratoriais (troponina aumentada), ECG (alterações específicas e clássicas do IAM) e ECO (alterações de contratilidade segmentar de parede). O diagnóstico diferencial de IAM durante a gravidez deve ser feito com embolia pulmonar, embolia amniótica, dissecção de aorta, CMPP e miocardite. Os exames adicionais para o diagnóstico, a estratificação do risco e o tratamento do IAM incluem cintilografia, ressonância magnética e a**ngiografia das coronárias.**

Em nosso entendimento, pacientes com síndrome coronariana aguda devem ter diagnóstico e tratamento definidos antes do parto. Por isso, em casos de dor torácica ou suspeita de doença isquêmica aguda, somos favoráveis à indicação de angiografia coronária, que, além de concluir o diagnóstico, favorece a chance de tratamento da artéria “culpada” pelo quadro isquêmico agudo. O risco da angiografia é relativamente pequeno diante do seu benefício para o planejamento do parto e a anestesia dessas pacientes.

O tratamento do IAM na gravidez é semelhante ao da população geral, inclusive quanto às técnicas de revascularização. Na dissecção de coronária, o tratamento clínico tem sido a primeira escolha. A intervenção, percutânea ou cirúrgica, fica reservada para os casos de comprometimento do tronco de coronária esquerda ou lesão proximal de descendente anterior.^226^ As complicações mais frequentes são: IC e choque cardiogênico (38%), arritmias (12%), angina recorrente e reinfarto (20%), mortalidade materna (7%) e morte fetal (7%).^52^A conduta para choque cardiogênico e parada cardiorrespiratória (PCR) segue as diretrizes convencionais, com a estratégia de parto de urgência quando houver viabilidade fetal.^52^

O tratamento farmacológico do IAM é semelhante ao recomendado na população em geral. Aspirina em baixas doses é segura;^92^ porém, há pouca informação sobre os inibidores P2Y12.^73^ Clopidogrel é liberado, mas deve ser suspenso sete dias antes do parto. Não há evidências de benefício do uso dessa medicação na dissecção de coronária; além disso, inibidores da glicoproteína IIb/IIIa, bivalirudina, prasugrel e ticagrelor não são recomendados.^73^Os betabloqueadores, exceto o atenolol, têm seu uso já está estabelecido na síndrome coronariana aguda. O ativador do plasminogênio tecidual recombinante (TPA) não atravessa a placenta, mas pode induzir complicações hemorrágicas (sangramento subplacentário).^52^Benefícios da heparinização a curto prazo provavelmente superam o risco de complicações hemorrágicas.

A liberação para “nova” gravidez em pacientes com cardiopatia isquêmica prévia pode ocorrer na ausência de isquemia residual ou sinais de disfunção ventricular. Não há dados de alta qualidade definindo quanto tempo a gravidez deve ser retardada após a síndrome coronariana aguda. Não entanto, a recomendação de 12 meses parece razoável, individualizada de acordo com comorbidades, estado cardiovascular e necessidade de terapia medicamentosa.

### 3.4.1. Pontos-chaves

- A progressiva incidência de DCI na gravidez se deve à maior idade materna e à presença crescente de fatores de risco;
- A incidência de IAM sem supradesnivelamento do segmento ST é maior durante a gravidez, e a artéria descendente anterior é a mais acometida;
- O quadro clínico da dissecção arterial coronária parece ser mais grave durante a gestação quando comparado com o da população geral;
- Vasoespasmo coronário pode ocorrer em consequência das medicações de uso obstétrico;
- Sintomas, ECG, troponina sérica elevada e alterações no ECO definem o diagnóstico da síndrome coronária aguda;
- A cineangiocoronariografia deve ser indicada para definir o diagnóstico e possibilitar o tratamento percutâneo;
- O tratamento segue regras gerais, com eventuais restrições da gestação;

## 3.5. Dislipidemia na Gestação

### 3.5.1. Alterações Lipídicas

Durante a gravidez ocorre substancial aumento na concentração plasmática das lipoproteínas, consequente à elevação de estrogênio e progesterona circulantes. Os triglicerídios aumentam duas a três vezes em relação ao valor pré-gestacional, alcançando o pico no termo da gestação, com retorno progressivo ao basal no final do puerpério. Da mesma maneira, ocorre um aumento progressivo dos níveis de colesterol total, que corresponde a duas a cinco vezes os valores anteriores à gestação, com decréscimo um pouco mais lento que os níveis de triglicerídios com normalização, que pode prolongar além das seis semanas após o parto.^228^

As frações lipoproteicas apresentam também alterações qualitativas, de modo que o colesterol da lipoproteína de alta densidade (HDL-c) e o colesterol da lipoproteína de baixa densidade (LDL-c) têm um quantidade aumentada de triglicerídios. O HDL-c tem comportamento um pouco diferente do colesterol total e dos triglicerídios, pois apresenta um aumento progressivo, chegando ao máximo na 24ª semana, com um acréscimo de 50% nos valores quando se compara com período não gravídico. Em seguida, apresenta uma queda equivalente a 15% acima dos valores antes da gravidez até o período de termo.^228^Os níveis de LDL-c aumentam em sincronismo com os do colesterol total; porém, apresentam um decréscimo mais retardado, podendo ocorrer a queda após a oitava semana de puerpério.

O fator responsável por essas alterações das lipoproteínas é o hormônio estrogênio. A queda do HDL-c após a 24ª semana é explicada pelo aumento da concentração plasmática de insulina, que representa aumento da resistência à insulina. Conclui-se, portanto, que os níveis de HDL-c estão mais relacionados ao nível de estrogênio na primeira fase e à insulina na segunda fase da gestação. Recomenda-se que a dosagem de um perfil lipídico seja adiada em pelo menos 4 ou 6 semanas após a gestação, principalmente naquelas mulheres sem alterações prévias.

Na atualização da Diretriz Brasileira de Dislipidemia e Prevenção da Aterosclerose, as recomendações para mulheres dislipidêmicas em idade fértil incluem orientação dietética e adoção de estilo de vida saudável, além de controle de peso corporal, atividade física e interrupção do tabagismo.^229^ A terapia com estatinas deve ser evitada em mulheres em idade fértil que planejam gravidez (classe II-A; C).

A hipertrigliceridemia gestacional ocorre para suprir demandas energéticas maternas, como precursor de hormônios para a placenta e para fornecer colesterol e ácidos graxos essenciais ao feto. Em gestantes no segundo e terceiro trimestres e em lactantes, a terapia com estatina não deve ser indicada (classe III-C). A contraindicação deve-se aos relatos de teratogenicidade, embora as informações disponíveis na literatura sejam inconclusivas.^104^

Fibratos, ezetimibe, niacina, colesterolamina e ômega-3 são considerados fármacos sem contraindicação absoluta, mas a colestiramina é o único com segurança definida. Os fibratos podem ser empregados nos casos de hipertrigliceridemia muito grave (nível plasmático de triglicérides > 1.000 mg/dl), sob a análise de risco/benefício para as gestantes (alta mortalidade para mãe e feto por pancreatite aguda). Entretanto, o controle dietético deve ser o tratamento de eleição em gestantes (classe IIA; C); em casos extremos, a aférese pode ser recomendada.^230^

Quanto aos ácidos graxos ômega-3, mulheres grávidas e lactantes devem ser aconselhadas a introduzirem na dieta peixes ricos em mineral, de águas profundas e com baixos níveis de mercúrio. Os recomendados são salmão, cavala, arenque, sardinha, atum e truta. Não há estudos sobre suplementação (cápsulas) e fitosteróis durante a gestação.

Merecem considerações as dislipidemias genéticas, tanto a hipertrigliceridemia com frequentes complicações pancreáticas como a hipercolesterolemia familiar. A abordagem terapêutica especial nessas circunstâncias graves é a aférese, sendo que, nos casos de hipercolesterolemia familiar, a aférese é LDL-aférese seletiva.^231^

Admitia-se que a dislipidemia na gravidez deveria ser considerada fisiológica, tanto é que não faz parte da rotina pré-natal o estudo do perfil lipídico. Contudo, recentemente, foram descritas estrias gordurosas em aorta de fetos de mães dislipidêmicas. A partir dessas observações, tem sido sugerido que a disfunção cardiometabólica materna pode não somente contribuir para efeitos maternos a longo prazo, mas também causar um risco de aterosclerose em gerações futuras. Essas considerações sugerem que o diagnóstico e o tratamento das dislipidemias devem ser realizados antes da concepção e ter uma continuidade durante a gestação e o pós-parto.^232^

### 3.5.2. Pontos-chaves

- Aumento de triglicerídios e colesterol ocorre na gravidez;
- O uso de estatinas naõ tem sido recomendado, embora exista controvérsia sobre seus efeitos teratogênicos;
- Dislipidemia materna pode induzir a aterosclerose fetal e também em futuras gerações.

## 3.6. Outras Doenças

### 3.6.1. Arterite de Takayasu

A arterite de Takayasu é uma vasculite crônica e idiopática que afeta de maneira predominante a aorta e seus ramos principais, as artérias coronárias e a artéria pulmonar. O processo inflamatório decorrente causa estreitamento, oclusão e aneurisma dos ramos afetados.^233^ A etiologia da doença é desconhecida, mas vários estudos demonstraram uma associação com antígenos leucocitários humanos, sugerindo predisposição para o processo imunomediado.^234^

***3.6.1.1. Prevalência***

A arterite de Takayasu é uma doença rara, com taxas de prevalência crescentes. As maiores ocorrem no Japão, com 100 a 200 novos casos anuais. As mulheres são mais acometidas, 80 a 90% dos casos, com início da doença entre 10 e 40 anos de idade, portanto no período fértil da vida, sendo a gestação motivo de atenção especial. É a vasculite mais frequente observada na gravidez justamente porque surge em pacientes jovens.^235^ A modulação das funções imunológicas induzidas pelo período gestacional pode influenciar o curso da doença e prejudicar a evolução materno-fetal.^236^

***3.6.1.2. Prognóstico***

A gravidez em pacientes com arterite de Takayasu tem um prognóstico incerto. Embora a maioria das gestações seja bem-sucedida, a incidência de hipertensão grave e pré-eclâmpsia é 40% maior quando comparada aos 8% na população geral. Complicações obstétricas como parto prematuro e natimorto são previstas.^235^ Pacientes com envolvimento de artéria renal e aorta abdominal tiveram complicações mais frequentes de pré-eclâmpsia e RCIU.^235^

Complicações maternas mais raras, porém de muita gravidade, são: aneurisma da aorta, acidente vascular cerebral, IC, insuficiência aórtica, infarto do miocárdio e dissecção da aorta.^235^ Outras complicações, mais comuns, incluem progressão da insuficiência renal, anemia, trombocitopenia e marcadores inflamatórios elevados.^235^

***3.6.1.3. Tratamento***

A conduta no tratamento da vasculite durante a gravidez é a convencional, exceto os três medicamentos teratogênicos que incluem o metotrexato, o micofenolato e a ciclofosfamida.^236^ Os demais são considerados compatíveis com a gestação. É preferível usar imunossupressores não hormonais para controlar a vasculite em atividade, reservando a prednisona para esquema de curta duração em doses moderadas na fase aguda ou na piora da doença. O tratamento pode ser iniciado antes da concepção e mantido durante a gravidez e na lactação.^237^

Os inibidores do fator de necrose tumoral podem ser continuados no período da pré-concepção, da gravidez e da lactação. Esses inibidores, quando são compostos à base de imunoglobulina G (IgG), atravessam a placenta a partir da 16ª semana, com progressivo aumento da transferência quanto mais próximo do termo. Em vista disso, eles não devem ser administrados após a 30ª semana de gestação, mas devem ser reintroduzidos no puerpério.^238^

***3.6.1.4. Pontos-chaves***

- A gravidez é autorizada quando a doença estiver em remissão, porque a vasculite tem prognóstico grave;
- O tratamento com corticosteroides e imunossupressores (azatioprina, ciclosporina e tacrolimus) melhora a evolução materno-fetal;
- Em casos de vasculite sistêmica, visto que o risco de eventos tromboembólicos é elevado, a prevenção com aspirina ou HBPM deve ser considerada;
- Arterite de Takayasu deve ser sempre considerada no diagnóstico diferencial na hipertensão arterial durante a gravidez;
- A contracepção deve ser eficaz e segura durante o tratamento com altas doses de medicamentos citotóxicos.

### 3.6.2. Doença de Kawasaki

A doença de Kawasaki é uma vasculite sistêmica, de etiologia desconhecida, que ocorre em crianças até 5 anos de idade, de prevalência asiática e predomínio do sexo masculino na proporção de 1,5:1. Na fase aguda, o envolvimento inflamatório das artérias coronárias resulta em desfechos clínicos e provoca formações aneurismáticas em 15 a 25% das crianças não tratadas. É uma das principais causas de cardiopatia adquirida na infância.^239^

Os aneurismas das artérias coronárias podem ser detectados precocemente por intermédio do ECO, e a perda do fluxo laminar nessas artérias pode favorecer a formação de coágulos.

O prognóstico da doença está relacionado à presença e ao tamanho dos aneurismas de artéria coronária. Aneurismas pequenos têm prognóstico favorável, com baixo risco de eventos isquêmicos do miocárdio. Por outro lado, aneurismas grandes e gigantes (diâmetro interno > 8 mm) apresentam alto risco de trombose e, consequentemente, de infarto do miocárdio, arritmias e morte súbita.^240^

A falta de diagnóstico e tratamento durante a fase aguda na infância tem contribuído para o achado, na idade fértil e na gravidez, de mulheres com sequelas vasculares da doença de Kawasaki.^241,242^A influência dos estados de hipercoagulabilidade e hipercinético, inerentes à gravidez, ao parto e ao puerpério, representa um potencial risco de graves eventos, como trombose, infarto do miocárdio e morte súbita, na história natural de mulheres portadoras de doença de Kawasaki complicada com aneurisma de coronárias. Acresce que a gravidez, *per se*, propicia o risco de rotura e/ou dissecção de coronárias, decorrentes das mudanças específicas nas paredes das artérias, que incluem: fragmentação das fibras reticulares, redução dos mucopolissacarídeos e perda da ondulação normal das fibras elásticas.

Nessa linha de raciocínio, admite-se que o estado de hipercoagulabilidade da gravidez e puerpério demanda a anticoagulação permanente. Portanto, está indicado o uso de aspirina em baixas doses (80 mg por dia) até 36 semanas, associada ao anticoagulante. Recomenda-se a HBPM no primeiro trimestre e após 36 semanas de gestação, e a varfarina em doses baixas no intervalo entre esses dois períodos. A literatura carece de dados sobre as metas dessa prevenção; contudo, é de consenso que o INR em torno de 2 é seguro e presumivelmente eficaz.

O antecedente de infarto do miocárdio aumenta o risco da gestação e a função ventricular é um fator determinante da evolução materna. O uso de betabloqueador (propranolol ou succinato de metoprolol) em baixas doses propicia menor consumo de oxigênio, em função do menor trabalho cardíaco.

***3.6.2.1. Avaliação Pré-concepção***

Na estratificação do risco para futura gravidez, deve-se considerar a presença de aneurisma de coronária, isquemia miocárdica e disfunção ventricular.

***3.6.2.2. Pontos-chaves***

- A presença de aneurisma de coronária moderado (> 3 mm e < 6 mm) em uma ou mais artérias têm indicação de uso permanente de aspirina em baixas doses;
- Aneurismas gigantes (> 8 mm) ou múltiplos, além da aspirina, necessitam da associação de anticoagulante;
- Em casos de isquemia miocárdica, a associação de aspirina, anticoagulante e/ou antagonistas dos canais de cálcio é recomendada.

### 3.6.3. Hipertensão Pulmonar

A HP é uma condição fisiopatológica que leva a sintomas debilitantes e a menor expectativa de vida, causada pelo comprometimento da circulação pulmonar. É definida quando a pressão média da artéria pulmonar (PAP) em repouso acima é igual a 25 mmHg, medida pelo cateterismo cardíaco direito. É uma doença progressiva, com predomínio no sexo feminino, que pode ocorrer no período reprodutivo. Em geral, acarreta insuficiência ventricular direita com risco de morte durante a gravidez, mas particularmente no puerpério.^243,244^

A gravidez em mulheres com HP é considerada de alto risco e as complicações maternas e neonatais e tem alcançado as taxas 50 a 70%, respectivamente com mortalidade próxima de 30%.^245^ Em vista disso, a gravidez é contraindicada.

As alterações fisiológicas da gestação, com destaque para queda da resistência vascular periférica, aumento do débito cardíaco e hipercoagulabilidade, são as razões da instabilização hemodinâmica materna. Soma-se a isso a ação dos hormônios sexuais na circulação pulmonar, tais como betaestradiol, progesterona e testosterona, que, se por um lado, atenuam a vasoconstrição pulmonar, por outro ativam fatores angiogênicos que estimulam a proliferação de células musculares lisas da vasculatura pulmonar, predispondo ao remodelamento reverso vascular.

Esta complexidade fisiopatológica da HP na gestação se resume em um aspecto primordial, que é a alteração da resposta vasodilatadora fisiológica compensatória da vasculatura pulmonar, tornando-se diminuída ou ausente, o que leva a um aumento significativo da pressão e resistência pulmonares. A incapacidade de o leito vascular pulmonar acomodar o aumento do débito cardíaco resulta em desproporção significativa na pós-carga do ventrículo direito e sua falência.^246^

A classificação da HP era feita de maneira simplista, dividida em dois grupos: primária e secundária, de acordo com a identificação de fatores de risco. Entretanto, desde 1998, a OMS tem proposto modificações na classificação da HP no sentido de permitir que diferentes tipos da doença fossem agrupados com base em sua fisiopatologia, resposta ao tratamento e prognóstico^247^(Tabela 25). Nessa classificação, vale lembrar que o termo HAP é descrito como um subgrupo da HP, caracterizado pela pressão de enchimento de VE (Pd2) < 15 mmHg e resistência vascular pulmonar > 3 unidades Wood.

**Tabela 25:** – Classificação da hipertensão arterial pulmonar

**Categoria 1**	Idiopática
	Hereditária
	Induzida por fármacos e toxinas: anorexígenos, quimioterápicos, inibidores de recaptação da serotonina, cocaína
	Associada a: cardiopatias congênitas, doenças do colágeno, infecção pelo HIV, hipertensão portal, esquistossomose
	Hemangiomas capilares pulmonares ou doença pulmonar veno-oclusiva
	Hipertensão pulmonar persistente do recém-nascido
**Categoria 2 - Hipertensão pulmonar devido a doença cardíaca esquerda**	Disfunção diastólica
	Disfunção sistólica
	Doença valvar
	Obstrução congênita/adquirida do coração esquerdo e obstrução do trato de saída e cardiomiopatias congênitas
**Categoria 3 - Hipertensão pulmonar devido a doença pulmonar e/ou hipoxemia**	Doença pulmonar obstrutiva crônica
	Doença intersticial pulmonar
	Doenças pulmonares com padrão misto, ou seja, restritivo e obstrutivo
	Distúrbios respiratórias obstrutivas so sono
	Hipoventilação alveolar
	Exposição crônica a altitude
	Doenças pulmonares ocupacionais
**Categoria 4**	Hipertensão pulmonar devido a tromboembolismo crônico
**Categoria 5 - Hipertensão pulmonar com mecanismos multifatoriais pouco esclarecidos**	Distúrbios hematológicos: anemia hemolítica crônica, distúrbios mieloproliferativos, esplenectomia
	Distúrbios sistêmicos: sarcoidose, histiocitose pulmonar, linfangioleiomiomatose
	Distúrbios metabólicos: doença de depósito de glicogênio, doença de Gaucher, distúrbios da tireoide
	Outros: obstrução tumoral, mediastinite fibrosante, insuficiência renal crônica, hipertensão pulmonar segmentar

*HIV: vírus da imunodeficiência humana.*

No diagnóstico da HP, sintomas como dispneia, dor torácica, edema dos membros inferiores, palpitação e tosse seca podem ser atribuídos à gravidez, mas a presença de síncope presume maior gravidade da doença.^248^O ECG e a radiografia do tórax mostram sobrecarga de câmaras direitas. O ECO transtorácico estima a PAP, avalia a função do ventrículo direito e identifica outras lesões estruturais do coração, possibilitando classificar o tipo de HP. O diagnóstico definitivo é por meio do cateterismo cardiaco e medidas das pressões ^246,247^

O planejamento familiar em pacientes com HP inclui a contra-indicação à gravidez, esclarecendo os riscos maternos e fetais, bem como a escolha de um método contraceptivo eficaz e seguro. Até o momento, não há evidências sobre o nível de pressão de artéria pulmonar (ponto de corte) para determinar o prognóstico para uma futura gravidez.

Entretanto, o resultado da gravidez é muito diferente quando são considerados subgrupos para classificação da HP.^248^ Vale ressaltar que as pacientes incluídas na categoria 2 (Tabela 25), como aquelas com estenose mitral, estenose aórtica e cardiomiopatias, recebem diferentes tratamento e aconselhamento do que os pacientes incluídos nas outras categorias.

Por esse motivo, a estratificação de risco de acordo com a categoria e a estratégia de tratamento para a gravidez deve receber apoio interdisciplinar em um hospital terciário com especialistas em HP, para que as melhores práticas possam ser adotadas.

Exceto gestantes incluídas na categoria 2, a proposta inicial ao longo do primeiro trimestre em pacientes com HP é a interrupção da gravidez, com ênfase no esclarecimento sobre os riscos impostos do desenvolvimento da gestação e do puerpério bem como os do procedimento de aborto terapêutico. No caso de a paciente não aceitar o aconselhamento, recomenda-se o atual protocolo que segue seguinte prática:^249^

1. Consulta multidisciplinar semanal à partir de 16 semanas de gestação;
2. Terapêutica farmacológica da HP individualizada;
3. A avaliação periódica do ECG e ECO e BNP no segundo e terceiro trimestres;
4. Hospitalização a partir de 28 semanas para terapêutica com oxigénio intermitente de acordo com a saturação arterial de oxigenio, anticoagulação, monitorização materno-fetal e planejamento do parto
5. O parto é de indicação obstétrica;
6. A anestesia geral é preferencial;
7. Contra-indicação à anestesia com bloqueios – peridural ou raquidiana.

A terapêutica farmacológica recomendada é o uso das prostaciclinas e seus análogos e os inibidores da fosfodiesterase tipo 5 que parecem ser seguros durante a gestação. O uso dos bloqueadores dos canais de cálcio é alternativa segura e eficaz para o subgrupo de pacientes que apresentam vasorreatividade documentada em CF I/II sem disfunção grave ventricular. Atentar para os seus efeitos inotrópicos negativos além da hipotensão arterial que podem limitar o uso dos bloqueadores de cálcio.^250,251^

As prostaglandinas parenterais são recomendadas em pacientes em classe funcional IV ou que apresentem evidências de comprometimento grave do ventrículo direito A maior parte da experiência com prostaglandinas parenterais é com epoprostenol intravenoso. Em pacientes com função ventricular preservada que estejam em CF I/II as prostaglandinas inaladas como o Ilosprost pode ser indicada. As prostaglandinas parenterais podem ser combinadas com inibidores da fosfodiesterase oral com resultados satisfatórios.^252^

Os bloqueadores dos receptores de endotelina e estimuladores de guanilato ciclase solúvel são contra-indicados na gravidez.^251,252^As prostaglandinas IV podem ser consideradas no momento do parto com monitorização invasiva tipo cateter venoso central e acesso arterial.

Vale lembrar que grande parte das mortes maternas ocorrem no puerpério e dentre as causas destacam-se a IC devido a falência de ventriculo direito, a hipoxemia e o tromboembolismo (trombose pulmonar ”in situ”).^248^ Por isso *a a*nticoagulação é essencial com doses terapêuticas de HBPM (1 mg kg a cada 12 h) no 1º trimestre da gravidez e após 36 semanas de gestação e nos demais período com a varfarina com dose diária com a meta de INR = 2 (Figure 6).

***3.6.3.1. Pontos Chaves e Recomendações***

- O diagnóstico de HP deve ser definido pelo cateterismo de câmaras direitas
- A gravidez deve ser desaconselhada em mulheres com HP;
- As categorias de HP de acordo com a classificação presente tem prognóstico e tratamento muito diferentes;
- A proposta de interrupção da gravidez deve ser considerada em pacientes com HP durante o de 1º trimestre, exceto em pacientes do grupo 2;
- Caso a gravidez seja mantida ela deve ser seguida em hospital terciário com equipe multidisciplinar especializada em HP;
- A farmacoterapia atual tem auxiliado o sucesso da gravidez em HP.

### 3.6.4. Doenças da Aorta

As doenças da aorta podem estar presentes em mulheres na idade reprodutiva e são consideradas importantes causas de complicação e até mesmo de morte durante a gestação.^253^ Esse fato se deve a três fenômenos fisiológicos da gravidez que causam um impacto prejudicial nas doenças da aorta: hemodinâmico (aumento do débito cardíaco); estrutural (crescimento progressivo da raiz de aorta até o terceiro trimestre); e hormonal (fragilidade da parede vascular). As causas mais frequentes das doenças de aorta em gestantes são: valva bicúspide, síndrome de Marfan, coarctação de aorta, síndrome de Ehlers-Danlos, síndrome de Turner e síndrome de Loeys-Dietz.

***3.6.4.1. Dissecção e Ruptura Aórtica***

A gestação aumenta a suscetibilidade da mulher para a dissecção e a ruptura da aorta. Na população geral, a incidência de dissecção da aorta é de 6 por 100.000 indivíduos/ano, contudo na gravidez esta ocorrência aumenta em 100 vezes, para aproximadamente 0,6%.Por isso, o diagnóstico de dissecção aórtica deve ser considerado em todas as pacientes com dor torácica durante a gravidez. Ela ocorre mais frequentemente no último trimestre (50%) ou no período inicial pós-parto (33%).^52^

A síndrome de Marfan é a doença mais comum do tecido conjuntivo, causada por uma mutação no gene FBN-1, que codifica a glicoproteína fibrilina herdada em um padrão autossômico dominante.^254^ O aumento médio de crescimento do diâmetro da aorta durante a gestação em portadoras da síndrome de Marfan é de 0,3 mm/mês, enquanto, na população em geral com a doença, é de 0,38 mm/ano.^254^ O aumento da taxa de dilatação da aorta diminui após o parto, mas permanece mais elevada do que a taxa pré-gestacional.^253^

O diagnóstico inclui história, exame físico, ECO e ressonância magnética da aorta. A angiotomografia da aorta torácica complementa a investigação quando há forte suspeita pelos exames anteriores de dissecção.

Um dos fatores mais importantes para determinar o risco de disseção da aorta é o seu diâmetro máximo (< 40 mm, risco de dissecção de 1%; > 40 mm, risco de dissecção de 10%).^255^ A gravidez geralmente é contraindicada se o diâmetro da aorta ascendente for maior que 40 mm em pacientes com história familiar de dissecção ou morte súbita, embora a dimensão exata ainda seja uma questão de debate.^254^ Parece haver uma baixa incidência de dissecção se o diâmetro da aorta for inferior a 4,5 cm; no entanto, a gravidez aumenta o risco tardio de complicações aórticas.^52,254,256^

Uma consideração importante é a área de superfície corporal, particularmente em mulheres pequenas. Índice de diâmetro aórtico maior que 27 mm/m^2^ está associado a um alto risco de dissecção, e a substituição profilática de raiz aórtica deve ser considerada.^52^

Problemas cardiovasculares associados também precisam ser avaliados, incluindo a possibilidade de regurgitação aórtica e prolapso da valva mitral com regurgitação associada.

Tem sido demonstrado que os betabloqueadores aumentam a distensibilidade da aorta e reduzem a velocidade da onda de pulso e reduzindo a taxa de complicações, tais como regurgitação, dissecção e IC congestiva. Considera-se a redução de 20% da frequência cardíaca de repouso como objetivo do tratamento.^257^

Recomenda-se vigilância ecocardiográfica periódica a cada 6 a 8 semanas para monitorar o tamanho da raiz da aorta da mãe, com o intervalo dependente dos achados ecocardiográficos iniciais.^254^

O tipo de parto preferível é o cesárea, em pacientes com dilatações de aorta > 45 mm, e deve ser realizado em um centro de atendimento terciário onde haja equipe cirúrgica experiente. Em pacientes com diâmetro < 45 mm e sem evento prévio, o parto pode ser normal, com analgesia precoce e fórceps de alívio.

Aconselhamento na pré-concepção exige determinação da doença de base, avaliação genética e a correção da dilatação da aorta de acordo com os limiares de seus diâmetros (Tabela 26).

**Tabela 26:** – Limiares dos diâmetros de aorta e indicação de intervenção considerando gravidez257

Doença de base	Diâmetro de aorta ascendente
S. de Marfan	45 mm
S. de Loeys-Dietz	40-45 mm
S Ehlers -Danlos IV	Contraindicação à gravidez
Valva bicúspide	50 mm
S. Turner	27 mm/m^2^

Síndrome de Ehlers-Danlos do tipo IV (vascular) cursa com complicações vasculares graves, com características de herança autossômica dominante e risco de transmissão de 50% para a prole.

A mortalidade materna é significativa e relaciona-se a ruptura uterina e dissecção de grandes artérias e veias. A gravidez é, portanto, considerada situação de alto risco e não aconselhada (risco IV-OMS); assim, essas mulheres devem ser aconselhadas em um processo de tomada de decisão compartilhada ao contemplar gravidez.^52^

Na síndrome de Ehlers-Danlos vascular, também uma doença rara e grave do tecido conjuntivo caracterizada por tecido vascular frágil, a ruptura vascular durante a gravidez tem sido relatada em até 50%, com taxas de mortalidade entre 5 e 50% A gestação, nesse caso, está também associada a ruptura prematura das membranas fetais, abortos espontâneos e prematuridade.^52^

A síndrome de Turner^256^ é a anormalidade dos cromossomos sexuais mais comum nas mulheres e ocorre em 1 a cada 1.500 a 2.500 crianças do sexo feminino nascidas vivas. A constituição cromossômica pode ser ausência de um cromossomo X (cariótipo 45,X) ou mosaicismo cromossômico (cariótipo 45,X/46,XX), além de outras anomalias estruturais do cromossomo X.^256^ A síndrome de Turner está associada a um risco aumentado de doença cardíaca, dilatação aórtica, hipertensão, diabetes melito e eventos de doença aterosclerótica.^256^

Estima-se que a dissecção de aorta em pacientes com síndrome de Turner seja 36 em 100.000 casos, porém é seis vezes mais comum em idades mais jovens do que na população geral.^52^Os fatores de risco incluem dilatação da aorta, valva aórtica bicúspide e coarctação da aorta.^52^ A gravidez deve ser evitada quando o índice de tamanho da aorta é > 25 mm/m^2^. Além disso, após a cirurgia de raiz, a paciente permanece em risco de dissecção tipo B.

Gravidez espontânea pode ocorrer em pacientes em mosaico Turner (0,5 a 10%), sendo muito comum secundária a fertilidade assistida. Por isso, recomenda-se avaliação cardiovascular antes de se iniciar o tratamento de fertilidade. Ademais, um bom controle da pressão arterial e da diabetes é obrigatório para todos os pacientes de Turner durante a gravidez.^52^

A síndrome de Loeys-Dietz^258^ é uma condição autossômica dominante rara. Foi descrita pela primeira vez em 2005 e está associada à formação ou dissecção do aneurisma da aorta ou de outras artérias, geralmente em idade precoce.^258^Foi identificada em indivíduos encaminhados para investigação de síndrome de Marfan^257^ ou de Ehlers-Danlos vascular que não apresentavam características clássicas dessas condições, mas sim outras características, incluindo tortuosidade arterial generalizada, hipertelorismo, úvula bífida/ampla ou fenda palatina.^257^

A síndrome resulta de mutações nos genes que codificam componentes da via de sinalização do fator de transformação do crescimento beta (TGF-β). A patologia aórtica é particularmente preocupante nessa condição, mas outras anormalidades vasculares também podem estar presentes.^258^

Morbimortalidade materna significativa tem sido descrita em pacientes com síndrome de Loeys-Dietz, mas é possível a gravidez bem-sucedida e não complicada.^258^No entanto, todas as pacientes com essa condição devem, no presente, ser tratadas como muito alto risco na gravidez e no período pós-parto, até que ferramentas confiáveis de previsão de risco estejam disponíveis.^258^

Não há estudos sobre o benefício e os riscos do parto cesárea quando comparado ao parto vaginal em pacientes com doenças hereditárias da aorta. Contudo, recomenda-se o parto cesárea de acordo com os limites de dilatação de aorta apresentados na Tabela 26. Abaixo desses limites, a via vaginal pode ser considerada.

***3.6.4.2. Pontos-chaves***

- Doenças da aorta constituem causa importante de morte materna no ciclo gravídico-puerperal;
- Gravidez aumenta a suscetibilidade da mulher para a dissecção e a ruptura da aorta;
- O planejamento de gravidez inclui diagnóstico da doença de base, ressonância magnética da aorta e vasos da base, e eventual correção cirúrgica da aorta de acordo com os limites de risco de dissecção ee aconselhamento genético;
- A ocorrência de dissecção de aorta com feto viável (> 28 semanas de gestação) indica cesárea de urgência, contudo se o feto for inviável, procede-se a cirurgia cardíaca com manutenção da gravidez;
- Mulheres portadoras das síndromes de Ehlers-Danlos, Turner e Loeys-Dietz além do alto risco de dissecção de aorta estão exposta a eventos complicadores como hipertensão, diabetes e outros aneurismas, que, em conjunto, representam um significativo aumento de morte materna durante a gravidez.

### 3.6.5. Doença de Chagas

***3.6.5.1. Prevalência***

A estimativa global da prevalência da infecção por *T. cruzi* em gestantes tem variado de 1 a 40%, sendo que aproximadamente 1,8 milhão de mulheres em idade fértil estavam infectadas na América Latina.^259^No Brasil, a prevalência de infecção em gestantes é aceita como sendo de 1,1%, com taxa de transmissão vertical de 1,7%.^259,260^

***3.6.5.2. Diagnóstico e Conduta da Infecção por* T. Cruzi *Durante a Gestação***

A avaliação sorológica para a infecção por *T. cruzi* durante o pré-natal é recomendada para as gestantes que vivem em áreas endêmicas ou são procedentes delas, ou que tenham recebido transfusão de sangue nessas regiões.^259,261^ Os testes mais utilizados fundamentam-se na maior sensibilidade e especificidade para a detecção da infecção pelo T.cruzi e incluem: *Enzyme Linked Immunosorbent Assay* (ELISA); hemaglutinação indireta (HAI) e imunofluorescência indireta (IFI). A transmissão pode ocorrer em qualquer momento da gravidez, mas o tratamento antiparasitário específico da infecção por *T. cruzi* está contraindicado durante a gestação e o aleitamento, em razão da teratogenicidade em animais. A exposição acidental ao benzonidazol não indica efeitos adversos no recém-nascido e não representa um critério para interrupção da gestação.^259^

A parasitemia materna elevada associa-se a maior risco de transmissão vertical e aborto.^261^ Por isso, na fase aguda da doença de Chagas, as gestantes devem ser avaliadas individualmente, e a decisão para o início do tratamento antiparasitário deve ser baseada na relação risco-benefício.

**Figura 7:**
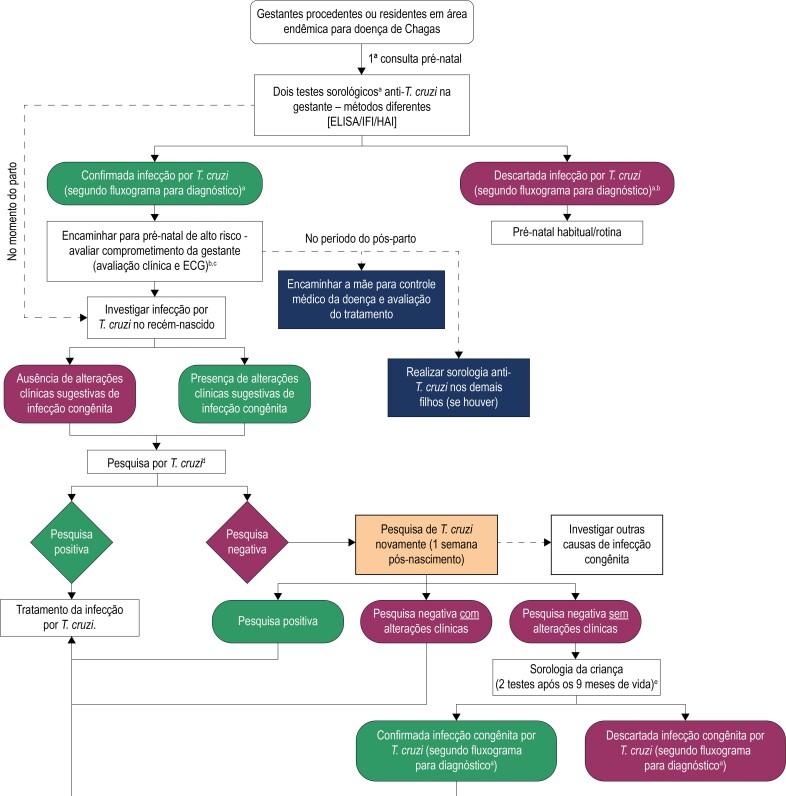
**–** Fluxograma para abordagem da infecção por Trypanosoma cruzi Não binômio mãe/filho. ELISA: Enzyme Linked Immunosorbent Assay; HAI: hemaglutinação indireta; IFI: imunofluorescência indireta. Adaptada de: II Consenso Brasileiro em Doença de Chagas.^259^

A evidência de infecção por *T. cruzi* não justifica a indicação de parto cesárea, embora a infecção congênita por *T. cruzi* possa resultar em restrição de crescimento uterino e prematuridade.^259,261^ Ressalta-se a importância de proceder a avaliações recomendadas durante o pré-natal, incluindo os testes anti-HIV. As infecções simultâneas por *T. cruzi* e por vírus da HIV representa risco aumentado de transmissão congênita de *T. cruzi* em função da elevada parasitemia, também implicando maior morbimortalidade perinatal.^260,261^ Após o parto, a mulher deverá ser encaminhada para avaliação clínica e tratamento específico. A Figura 7 mostra as orientações quanto à conduta da doença de Chagas durante a gravidez.^259^

***3.6.5.3. Cardiopatia Chagásica Crônica***

A cardiopatia chagásica na forma indeterminada não acrescenta riscos adicionais à gravidez, enquanto aquelas com disfunção ventricular ou arritmogênica associam-se a complicações como IC, tromboembolismo e arritmias complexas. Nesses casos, a gravidez é de alto risco e, às vezes, desaconselhada, na dependência do grau de comprometimento cardíaco, que pode ser estabelecido por ECO e Holter de 24 horas.

***3.6.5.4. Transmissão Vertical de* Trypanosoma Cruzi**

A transmissão vertical (de mãe para filho) de *T. cruzi* depende do grau de parasitemia; pela via transplacentária, pode ocorrer em qualquer fase da doença (aguda ou crônica), o que exige tratamento prévio à gestação nas mulheres infectadas em idade fértil. Ressalta-se que a transmissão vertical pode recorrer durante todo o período reprodutivo, e a detecção da transmissão vertical na prática é complicada, já que a maioria dos casos congênitos é assintomática. A doença de Chagas congênita é considerada aguda, e sua notificação é compulsória dentro das ações de vigilância epidemiológica.^259,262,263^

Na fase aguda da doença de Chagas, existe a possibilidade de transmissão pelo leite materno, enquanto na sua fase crônica, a transmissão durante a lactação ocorre em casos de sangramento por fissura do mamilo, e não propriamente pelo leite.

***3.6.5.5. Reativação da Doença de Chagas***

Durante a gestação, mecanismos e alterações imunológicos no organismo materno podem propiciar a reativação da doença de Chagas crônica em casos previamente infectados. A reativação é definida pela positividade dos seguintes exames, independentemente da presença de outros sinais e sintomas:

- Presença do parasito ao exame microscópico direto no sangue ou em secreções biológicas como líquor, pleura, pericárdio e líquido ascético;
- Exame histopatológico de lesões teciduais (paniculite, miocardite, encefalite, enterite, colpite) com encontro de ninhos do parasito em meio a infiltrado inflamatório agudo.

***3.6.5.6. Aleitamento***

Não se recomenda a suspensão da lactação em puérperas com doença de Chagas na fase crônica, exceto nos casos de fissura mamária, em situações de elevada parasitemia ou em mulheres na fase aguda da doença.^259^

Se houver exposição ao leite materno de mãe infectada, seja na forma aguda ou na crônica, e com fissuras no mamilo, o monitoramento de aquisição da infecção por *T. cruzi* pelo lactente deve ser realizado durante o período de exposição. Em alguns desses casos, o tratamento térmico do leite materno antes da administração aos lactentes pode ser considerado.^259,262,263^

A lactação deve ser suspensa nos casos de infecção por *T. cruzi* e HIV, uma vez que a lactação, independentemente da associação com a doença de Chagas, está associada a um risco adicional de 7 a 22% de transmissão do HIV. Por sua vez, em casos de infecção materna aguda por HIV, o aleitamento natural aumenta a probabilidade de transmissão vertical do vírus para 29%. No Brasil, a mãe tem direito a receber fórmula láctea infantil pelo menos até que seu filho complete 6 meses de idade.^259,263^

***3.6.5.7. Pontos-chaves***

- Avaliação sorológica é recomendada para todas as gestantes com epidemiologia positiva;
- O risco para a gestação depende da forma clínica da doença de Chagas;
- Gravidez pode propiciar a reativação da doença;
- O aleitamento não deve ser desaconselhado;
- Tratamento antiparasitário está contraindicado durante a gestação e o aleitamento;
- A indicação do tipo de parto é obstétrica.

## 4. Síndrome Hipertensiva da Gestação

### 4.1. Introdução

A síndrome hipertensiva na gestação é considerada um grave problema de saúde pública, com expressiva taxa de morbimortalidade materna e fetal, tanto nos países desenvolvidos como naqueles em desenvolvimento. É a complicação médica mais comum da gestação e afeta 5 a 10% das gravidezes em todo o mundo.

A pré-eclâmpsia ocorre em aproximadamente 3% de todas as gravidezes nos Estados Unidos, sendo responsável por 9% das mortes maternas,^264^ com aumento de 25% em incidência nas últimas duas décadas. Há registro de aumento da proporção de mulheres com pré-eclâmpsia nos últimos anos na ordem de 2,2%, em 2009, para 5,58% em 2013, sendo que, ao longo dos últimos cinco anos, 22,5% experimentaram uma complicação em geral grave.^265^

Embora pesquisas tenham evoluído na área das síndromes hipertensivas na gestação, sua etiologia permanece desconhecida. Inúmeros são os desafios metodológicos das pesquisas em relação à pré-eclâmpsia, como definições da hipertensão na gravidez, níveis de gravidade e fisiopatologia. Esses dados provavelmente interferem nas pesquisas e nos desfechos, justificando as recomendações a seguir.

### 4.2. Recomendações para Aferição da Pressão Arterial

- Os dispositivos de mensuração da pressão arterial em mulheres grávidas devem ser precisos e validados para esta população especial. O manguito deve ter tamanho apropriado com comprimento de 1,5 vezes a circunferência do braço.
- A pressão arterial deve ser aferida sentada, em repouso de pelo menos cinco minutos. Pode ser também na posição de decúbito lateral esquerdo, em repouso, não devendo diferir da obtida na posição sentada;
- É preciso considerar a fase V de Korotkoff para a determinação da pressão arterial diastólica (PAD);^266^
- A HAB e a hipertensão mascarada (HM) são consideradas apresentações relativamente comuns na gravidez. Ocorrem em até 1/3 das gestantes, de modo que o MAPA e o monitoramento residencial da pressão arterial (MRPA) constituam exames complementares úteis na decisão clínica, fundamentais para evitar o tratamento desnecessário e potencialmente lesivo ao feto;^33,267^
- São consideradas gestantes hipertensas mulheres com PAS ≥ 140 mmHg e/ou PAD ≥ 90 mmHg;
- A gravidade da hipertensão na gravidez é considerada com base na ocorrência de envolvimento de órgão-alvo, bem como no nível da pressão arterial;^268^
- A hipertensão grave é definida com base em níveis tensionais ≥ 160 x 110 mmHg, os quais estão associados ao aumento do risco de acidente vascular cerebral em gestantes.^52,266,268^

### 4.3. Classificação

**Figura 8:**
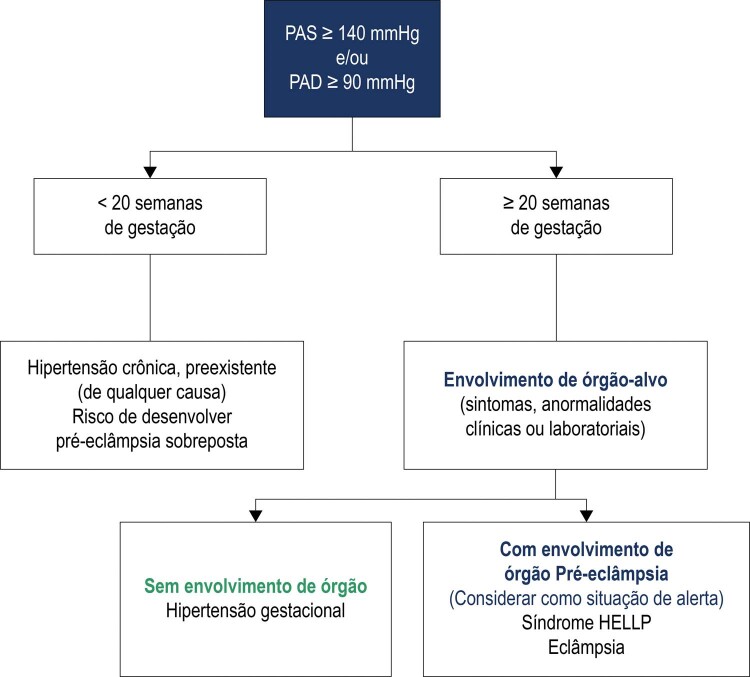
– *Classificação das síndromes hipertensivas. HELLP: hemólise, elevação das enzimas hepáticas, plaquetopenia; PAD: pressão arterial diastólica; PAS: pressão arterial sistólica.*

A classificação mais utilizada das síndromes hipertensivas da gestação é a adotada pelo Report of the American College of Obstetricians and Gynecologists – Task Force on Hypertension in Pregnancy,^269^ aplicada na Diretriz da Sociedade Brasileira de Cardiologia para Gravidez na Mulher Portadora de Cardiopatia (Figura 8).^270^ De acordo com ela, as síndromes são classificadas da seguinte maneira:

- Hipertensão crônica, preexistente (de qualquer causa);
- Pré-eclâmpsia/eclâmpsia;
- Hipertensão crônica com pré-eclâmpsia sobreposta;
- Hipertensão gestacional.

Com base nesse posicionamento, será mantida uma classificação de quatro categorias e enfatizada a importância das outras apresentações da hipertensão arterial, como:

- HAB;
- HM;
- Hipertensão gestacional transitória: ocorre sem o desenvolvimento da pré-eclâmpsia, com normalização da pressão arterial em 12 semanas pós-parto e que se resolve sem tratamento;^267,270^
- Hipertensão pós-parto: surge, em geral, entre 2 semanas e 6 meses após o parto. Leve e lábil, normaliza-se dentro do primeiro ano e pode estar relacionada à persistência de hipertensão gestacional, pré-eclâmpsia ou hipertensão crônica, ou ser secundária a outras causas;^269^
- Hipertensão pré-natal inclassificável: esse termo é usado quando a primeira medida de pressão é registrada após a 20ª semana e não fica evidente se é crônica ou preexistente; apenas a reavaliação pós-parto entre a 6ª e a 12ª semana faz o diagnóstico.

#### 4.3.1. Hipertensão Crônica, Preexistente (Essencial ou Secundária)

Ocorre quando a pressão arterial é ≥ 140 x 90 mmHg (hipertensão preexistente; em geral, hipertensão essencial ou diagnosticada antes da 20ª semana de gravidez). É comum ser diagnosticada por volta do primeiro trimestre ou bem no início do segundo. Está associada a desfechos maternos e fetais adversos; portanto, deve-se ter um controle mais rigoroso da pressão arterial materna (110 a 140/85 mmHg), monitorando o crescimento fetal e avaliando repetidamente o desenvolvimento de pré-eclâmpsia e complicações maternas.^267^

A hipertensão pode não ser diagnosticada em muitas mulheres que se apresentam para o pré-natal pela primeira vez no segundo trimestre. Mulheres grávidas podem ser consideradas normotensas na fase inicial da gestação, devido ao decréscimo fisiológico da pressão arterial na gravidez no primeiro trimestre, assim como uma elevação na pressão arterial pode ser diagnosticada como hipertensão gestacional, porque a pressão não foi verificada antes da 20ª semana de gestação. Habitualmente, a hipertensão crônica persiste após 42 dias pós-parto.^268^

O diagnóstico de hipertensão crônica será feito corretamente somente se a pressão arterial for reavaliada após a 6ª a 12ª semana pós-parto.^271^

#### 4.3.2. Pré-eclâmpsia/Eclâmpsia

É uma síndrome hipertensiva complexa e pode deteriorar-se rapidamente e sem aviso; não se recomenda classificá-la como “leve” ou “grave”. O diagnóstico ocorre com aparecimento de hipertensão, que se instala a partir da 20ª semana de gestação com uma ou mais condições relacionadas:

- Proteinúria (> 0,3 g/24 h) e/ou disfunções orgânicas maternas tipo evidência de lesão renal aguda materna (creatinina ≥ 1 mg/dl);
- Disfunção hepática (transaminases hepáticas elevadas, > 40 UI/L);
- Com ou sem dor abdominal (quadrante superior ou epigástrio);
- Complicações neurológicas (incluem eclâmpsia, estado mental alterado, cegueira, acidente vascular cerebral, clônus, cefaleias intensas, escotoma visual persistente);
- Hemólise ou trombocitopenia e/ou disfunção uteroplacentária (restrição do crescimento fetal, análise anormal da forma de onda do Doppler da artéria umbilical ou natimorto).

A existência de proteinúria não é mandatória para o diagnóstico e pode ocorrer pela primeira vez intraparto, ou precocemente no pós-parto. Desse modo, o Ideal seria identificar gestantes com risco de desenvolver pré-eclâmpsia. As recomendações de *screening*, tipo pesquisar proteinúria para esse fim, são falhas; a única rotina consensual é aferir a pressão arterial rotineiramente nas visitas do pré-natal.^272,273^

***4.3.2.1 Síndrome HELLP (Hemólise, Elevação das Enzimas Hepáticas, Plaquetopenia)***

Trata-se da manifestação grave da pré-eclâmpsia e não deve ser considerada como entidade separada.

#### 4.3.3. Hipertensão Crônica (Preexistente) com Pré-eclâmpsia Sobreposta

Ocorre em 25% das gestantes hipertensas crônicas. O diagnóstico é feito quando uma gestante com hipertensão essencial crônica desenvolve alguma das disfunções orgânicas maternas compatíveis com pré-eclâmpsia. Como após a 20ª semana de gestação pode ocorrer o aumento habitual da pressão arterial, apenas elevações da pressão arterial não habilitam a considerar o diagnóstico de pré-eclâmpsia sobreposta, assim como a restrição do crescimento fetal pode fazer parte do quadro da hipertensão crônica.

Em caso de doença renal com proteinúria de base, um aumento da proteinúria também não é parâmetro diagnóstico de pré-eclâmpsia sobreposta; entretanto, caso não haja proteinúria preexistente, seu aparecimento no contexto da elevação da pressão arterial é suficiente para o diagnóstico.

#### 4.3.4. Hipertensão Gestacional

É uma hipertensão “nova” que surge após a 20ª semana de gestação, na ausência de proteinúria, sem anormalidades bioquímicas ou hematológicas. Geralmente não é acompanhada por RCIU, e os desfechos geralmente são bons; porém, cerca de um quarto das mulheres com hipertensão gestacional (particularmente aquelas que se apresentam com menos de 34 semanas) evolui para pré-eclâmpsia e apresenta desfechos desfavoráveis. Em geral se resolve dentro das 6 semanas pós-parto.^52^

***4.3.4.1. Pontos-chaves***

- Considerar hipertensão quando a medida de pressão arterial for PAS ≥ 140 mmHg e/ou PAD ≥ 90 mmHg;
- Definir como hipertensão grave quando PAS ≥ 160 mmhg e PAD ≥ 110 mmHg. Estes níveis estão associados com aumento de risco de AVC em gestantes;
- Hipertensão arterial crônica, pré-existente (essencial ou secundária) deve ter um controle rigoroso da pressão arterial materna (PAS = 110 a 140 mmhg e PAD = 85 mmHg), monitorização do crescimento fetal e avaliaçao repetida na presunção de ocorrência da pré-eclâmpsia e outras complicações;
- Pré-eclâmpsia/eclâmpsia - síndrome hipertensiva complexa, pode deteriorar rapidamente e sem prenuncio. Não é recomendado classificar a pré-eclâmpsia como "leve" ou "grave”;
- A existência de proteinúria não é essencial para o diagnóstico e pode ocorrer pela primeira vez no período intra-parto ou no pós-parto imediato.

## 4.4. Tratamento da Síndrome Hipertensiva Gestacional

### 4.4.1. Tratamento Não Farmacológico269

Considerando gestantes os níveis tensionais – PAS ≥ 140 mmHg ou PAD ≥ 90 mmHg, as recomendações são:

- Não há indicação de repouso rotineiramente nas gestantes com Tratamento da síndrome hipertensiva gestacional (SHG);^274^
- Exercícios físicos são recomendados por pelo menos 3 dias por semana, em uma média de 50 min por sessão, incluindo atividades aeróbicas e treinamento de força e flexibilidade; A atividade física com exercícios moderados pode ser continuada nas mulheres habituadas a praticá-los;^112^
- A dieta deve ser saudável, rica em nutrientes, proteínas, fibras e cereais;
- A suplementação de cálcio é necessária, 1,5 a 2,0 g diários, principalmente em áreas onde a ingestão dietética de cálcio for baixa;
- O ganho de peso na gestante se baseia no índice de massa corpórea (IMC) antes da gestação:^131^

– IMC de 25 kg/m^2^ (normal): ganho de 11,2 a 15,9 kg;
– IMC de 25 a 29,9 kg/m^2^(sobrepeso): ganho de 6,8 a 11,2 kg;
– IMC ≥ 30 kg/m^2^ (obesas): ganho de 6,8 kg.

**Não são recomendados:**

- Nenhuma dieta hipocalórica, mesmo em mulheres obesas, pois pode levar ao retardo de crescimento fetal;
- Restrição de sal durante a gestação com a intenção de prevenir a SHG ou dietas com baixo teor de sódio (menos de 100 mEq por dia) nas gestantes com hipertensão arterial crônica;
- Uso de suplementos alimentares (magnésio, vitaminas C, E e D, óleos de peixe ou óleos de algas ou alho) com o objetivo de prevenir a SHG.

### 4.4.2. Quando Tratar – Alvo da Pressão Arterial

Existem divergências, nos consensos internacionais, quanto ao início do tratamento farmacológico na SHG.^131,275-279^ Apesar disso, a recomendação predominante é iniciar anti-hipertensivos orais na SHG com a PAS de 140 a 155 mmHg e a PAD de 90 a 105 mmHg, aferidas no consultório, ou ≥ 135/85 mmHg em domicílio. Especificamente, nas hipertensões crônicas (HAC), na hipertensão gestacional (HG) ou na pré-eclâmpsia, a terapia anti-hipertensiva é recomendada se PAS ≥ 140 mmHg ou PAD ≥ 90 mmHg.^273,280^

A meta do tratamento é da pressão arterial ser de 110 a 140/80 a 85 mmHg. Na eventualidade da PAD ≤ 80 mmHg, os anti-hipertensivos devem ser reduzidos ou cessados. A queda abrupta da pressão arterial materna superior a 25% , aumenta o risco de hipoperfusão em órgãos-alvo da mãe e de baixo fluxo sanguíneo placentar, podem contribuir negativamente para a nutrição fetal e / ou oxigenação.

O objetivo primário do tratamento da SHG é a prevenção do acidente vascular cerebral, a progressão da doença renal preexistente ou de outras lesões em órgãos-alvo materno, mantendo a circulação útero-placentária. Os níveis pressóricos devem ser sempre correlacionados ao período gestacional em curso, porque as mudanças fisiológicas , específicas da gestação, ocorrem a cada trimestre gestacional,^281^ como o aumento da taxa de filtração glomerular, que altera a biodisponibilidade dos fármacos na gestação.^61,282^

Nas hipertensas crônicas, até o momento, não há provas suficientes que demonstrem que, ao atingir um nível específico (ideal) da pressão arterial ou do uso de um anti-hipertensivo específico, esteja associado com diminuir o risco do desenvolvimento de pré-eclâmpsia sobreposta.^279-282^

Recentemente, a última revisão sistemática da Cochrane^283^concluiu que os dados são insuficientes para determinar os benefícios materno-fetais do do uso de anti-hipertensivos na hipertensão leve a moderada pressão (PAS 140 a 169 mmHg e/ou PAD 90 a 109 mmHg) durante a gravidez. Entretanto, o tratamento com fármacos anti-hipertensivos reduzem o risco de hipertensão grave. Contudo, não diminui a ocorrência de pré-eclâmpsia, RCIU, descolamento de placenta ou efeitos adversos neonatais. Concluindo, são necessários ensaios clínicos mais adequados para fornecer estimativas confiáveis dos verdadeiros benefícios e efeitos adversos do tratamento anti-hipertensivo para hipertensão leve a moderada.

O ensaio clínico multicêntrico randomizado internacional “Control of Hypertension in Pregnancy Study (CHIPS)”, com gestantes que não apresentavam proteinúria e hipertensão “não grave” (PAS de 140 a 159/90 e PAD de 109 mmHg) demonstrou que o controle pressórico “menos rigoroso” da pressão arterial (PAD de 100 mmHg) comparado ao controle “mais rigoroso” (PAD de 85 mmHg) teve correlação com maior incidência de hipertensão grave (pressão arterial ≥ 160/110 mmHg), com pré-eclâmpsia, perda fetal, recém-nascidos de baixo peso, prematuridade e internação em UTI neonatal.^284,285^

### 4.4.3. Anti-hipertensivos Orais – Hipertensão Crônica/Hipertensão Gestacional

Todos os anti-hipertensivos atravessam a barreira placentária; por isso, o uso terapêutico de fármacos na gravidez exige a análise de risco-benefício com individualização do tratamento.^278,282^

No Brasil, os medicamentos orais disponíveis e usualmente empregados são metildopa, betabloqueadores (exceto atenolol), hidralazina e BCC, nifedipina, anlodipina e verapamil.^275^O tratamento anti-hipertensivo nas gestantes com hipertensão gestacional ou hipertensas crônicas recomenda-se iniciar o tratamento com monoterapia e fármacos de primeira linha,^67-276^ tais como metildopa, BCC e betabloqueadores (exceto atenolol).

Caso os níveis ideais da pressão arterial não sejam alcançados, deve-se considerar a associação com os medicamentos orais de segunda linha: clonidina, hidralazina e diuréticos tiazídicos.^271,274^O potencial dos diuréticos causarem depleção de volume intravascular e, portanto, comprometer circulação útero placentar, o RCIU ou oligohidrâmnio, não é suportada em estudos randomizados mais recentes e numa revisão sistemática de diuréticos para a prevenção de pré-eclâmpsia.^71,286-287^

Fármacos considerados de primeira linha:

- **Agonista dos receptores alfa-2- adrenérgicos de ação centra**l**:** diminuem a pressão arterial por reduzir a resistência periférica vascular. Podem alterar a frequência e o débito cardíaco. A α-metildopa constitui o anti-hipertensivo mais bem estudado na gestação.^67,68^ no entanto, a metildopa tem apenas um efeito anti-hipertensivo leve, com um início de ação lento (3 a 6 h) e com duração média de 6 a 8 horas. Os efeitos colaterais mais comuns maternos dose-dependente são sonolência e boca seca. Já os independentes de dose incluem a elevação das enzimas hepáticas, em até 5% das mulheres e anemia hemolítica auto-imune.^68^ A dose inicial recomendada é de 250 mg, 2 ou 3 vezes ao dia (dose máxima 3 g/dia);
- **Bloqueadores dos canais de cálcio (BCC)**: a nifedipina oral parece não ser teratogênica.^64-66,81-83,288,289^Ensaios clínicos demonstram que não é afetado o fluxo sanguíneo na artéria umbilical. Os efeitos colaterais maternos com o uso de BCC incluem taquicardia, palpitações, edema periférico, dores de cabeça e rubor facial. A experiência com nifedipina tem sido favorável.^276^ Embora não seja licenciado especificamente para a gestação, recomenda-s eo seu uso juntamente com labetalol e metildopa . A dose diária máxima da nifedipina é de 120 mg, fracionada em três ou quatro tomadas ou 30-60 mg uma vez ao dia (liberação prolongada).^270-273^ A administração pela via sublingual é contraindicada por determinar resposta hipotensora imprevisível, excessiva ativação autonômica e isquemia aguda do miocárdio;
- A exposição à amlodipina no início da gravidez não parece estar associada a um aumento da taxa de malformações fetais em comparação com outros agentes anti-hipertensivos^290,291^e o efeito anti-hipertensivo é lento (± 8 horas);
- **Betabloqueadores:** nenhum dos betabloqueadores têm sido associados a teratogenicidade^76-79^ O RCIU e o baixo peso da placenta foram associados ao uso de atenolol.^79,80,271^ A exposição a qualquer betabloqueador está associada a risco de bradicardia e hipoglicemia neonatal, podendo causar sedação, distúrbios do sono e depressão na gestante. Não caso do propranolol, há relatos de RCIU, bradicardia e hipoglicemia neonatal, especialmente com doses altas (160 mg/dia).^81^ O labetalol não é comercializado no Brasil.

Considerados de segunda linha são:

- A clonidina mostra um aumento exagerado da pressão arterial (efeito rebote) quando o tratamento é interrompido abruptamente. Tem um efeito hipotensor maior que a metildopa;
- Hidralazina: é predominantemente usada por via intravenosa no tratamento da hipertensão grave na pré-eclâmpsia;
- Diuréticos: Os tiazídicos podem ser continuados nas gestantes com HAS crônica, desde que não promovam depleção de volume mas a clorotiazida pode aumentar o risco de anomalia congênita e de complicações neonatais.^276,286^

Os seguintes anti-hipertensivos orais são contra-indicados durante a gestação: ^290^

- IECA e BRA, associados a lesão renal aguda fetal e oligoidrâmnio e que devem ser suspensos antes da concepção;
- Atenolol (betabloqueador), que leva a RCIU e baixo peso placentário; ^291,292^
- Espironolactona, que tem um efeito antiandrogênico durante o desenvolvimento fetal; ^293^
- Clorotiazida, que pode aumentar o risco de anomalias congênitas e complicações neonatais.

### 4.4.4. Anti-hipertensivos na Hipertensão Grave/Pré-eclâmpsia275,276,278,279,298-300

O prognóstico materno e fetal está correlacionado diretamente ao atendimento inicial prestado a essas gestantes.^292^ A pré-eclâmpsia grave é a hipertensão sistólica grave é de início agudo quando a pressão sistólica for maior ou igual a 160 mmHg; hipertensão diastólica grave igual ou superior a 110 mmHg; ou ambos, podendo ocorrer no período pré-natal, intraparto ou pós-parto.^277^ É uma emergência obstétrica e requer tratamento imediato com anti-hipertensivos. O objetivo não é normalizar a pressão arterial, mas atingir níveis de 140-150/90-100 mmHg^277^ ou a reduzir de 15% a 25% da PA.^275^

A pré-eclampsia grave está associada à síndrome da encefalopatia reversível posterior (PRES) caracterizada por dor de cabeça, sintomas visuais, consciência prejudicada, crises epilépticas e, ocasionalmente, defeitos neurológicos focais.^301^

A mulher com diagnóstico de pré-eclamspsia grave deve ser encaminhada rapidamente a um centro terciário de referencia para tratamento. Antes da transferência inter-hospitalar, a pressão arterial (PA) deve ser estabilizada e outras medidas iniciadas, como sulfato de magnésio para profilaxia da eclâmpsia.^293^ É recomendado que o sulfato de magnésio deva ser usado para a prevenção e tratamento de convulsões em mulheres com hipertensão gestacional e pré-eclâmpsia com características graves ou iminência de eclâmpsia.A estabilização materna deve ocorrer antes do parto, mesmo em circunstâncias urgentes.

Considerar indicação de internamento em UTI com os seguintes critérios: gestantes com pré-eclâmpsia grave (PAS ≥ 160 mmHg e PAD ≥ 110 mmHg); insuficiência respiratória com necessidade de assistência ventilatória mecânica; eclâmpsia, síndrome HELLP, oligúria, edema agudo de pulmão e complicações neurológicas mais frequentes como o acidente vascular encefálico e a PRES.^294^

A intubação endotraqueal é outro risco na emergência hipertensiva . A indução de anestesia geral e intubação nunca deve ser realizada sem antes tomar medidas para eliminar ou minimizar a resposta hipertensiva à intubação. A monitoração materno-fetal deve ser rigorosa pela equipe médica e de enfermagem durante o tratamento. Após a estabilização inicial, a equipe deve monitorar de perto a PA e instituir a terapia de manutenção, conforme necessário.

O *American College of Obstetricians and Gynecologists*^268,269,280^ faz as seguintes recomendações e conclusões:

- O tratamento com agentes de primeira linha deve ser imediato ou ocorrer o mais rápido possível dentro de 30 a 60 minutos após a hipertensão grave confirmada (pressão arterial maior que 160/110 mmHg e persistente por 15 minutos) para reduzir o risco de derrame materno. A paciente deve ser posicionada em posição sentada ou semi-reclinável, com as costas apoiadas, não devem ser reposicionados para ficarem reclinados ou ficarem de lado para obter uma pressão arterial baixa, pois fornecerá uma leitura falsa da medida pressórica;^292^
- A monitorização materna e fetal por um médico e equipe de enfermagem é recomendada durante o tratamento da hipertensão grave de início agudo;
- Após a estabilização inicial, a equipe deve monitorar de perto a pressão arterial e instituir terapia de manutenção, conforme necessário;
- Labetalol e hidralazina (IV) intravenosos são considerados medicamentos de primeira linha para o tratamento da hipertensão grave de início agudo em gestantes e mulheres no período pós-parto;
- A nifedipina oral de liberação imediata também pode ser considerada como terapia de primeira linha, principalmente quando o acesso IV não está disponível;
- O uso de labetalol IV, hidralazina IV ou nifedipina oral de liberação imediata para o tratamento de hipertensão grave de início agudo em pacientes grávidas ou no pós-parto não requer monitoramento cardíaco;
- Nas raras circunstâncias em que bolus labetalol, hidralazina ou nifedipina oral de liberação imediata falham em aliviar o início agudo, hipertensão grave e são administrados em doses apropriadas sucessivas, consulta emergente com um anestesista, subespecialista em medicina materno-fetal ou subespecialista em cuidados intensivos para discutir a intervenção de segunda linha é recomendado;
- O sulfato de magnésio não é recomendado como agente anti-hipertensivo, mas o sulfato de magnésio continua sendo o medicamento de escolha para a profilaxia das crises em mulheres com hipertensão grave de início agudo durante a gravidez e o período pós-parto. O início do magnésio não deve ser retardado no cenário de hipertensão grave aguda; é recomendado independentemente de o paciente apresentar hipertensão gestacional com características graves, pré-eclâmpsia com características graves ou eclâmpsia.

## 4.5. Conduta na Emergência Hipertensiva em Pré-eclâmpsia (PA ≥ 160/110 mmHg)

Na emergência hipertensiva as drogas com eficácia são a nifedipina, hidralazina e labetalol. Podem existir diferenças sutis em seus perfis de segurança. A evidência é inadequada para outras drogas. Os medicamentos de uso intravenoso são a hidralazina e labetalol intravenoso (não disponível no Brasil). A nifedipina oral, hoje é aceita como de primeira linha. Uma revisão sistemática recente da Cochrane não encontrou diferenças significativas dessas tres drogas no tratamento da crise hipertensiva quanto à eficácia ou segurança entre a hidralazina e o labetalol ou entre a hidralazina e os BCC.^277,295-297^

- **Nifedipina**: dose inicial de 10-20 mg por via oral. O tempo de inicio de ação da nifedipina oral é de 5-10 minutos. A dose deve ser repetida em 20 minutos, se necessário (se pressão arterial for > 155/105 mmHg). Manter 10-20 mg a cada 2-6 horas com a dose diária máxima é de 120 mg. Repetir a medicação se pressão arterial for > 155/105 mmHg e administrar no máximo três doses. Após 20 min da terceira dose e a persistência de hipertensão arterial, administrar fármaco de segunda escolha .Salienta-se que os comprimidos não devem ser mastigados e não devem ser utilizadas as formulações pela via sublingual;
- **Hidralazina**: Dose inicial de 5 mg por via intravenosa (dose máxima de 45 mg) em bólus, lentamente, durante 1 a 2 min, repetir , se necessário, 5mg a cada 20 minutos (obs: A ampola de hidralazina contém 1 ml, na concentração de 20 mg/ml. Diluir uma ampola (1 ml) em 19 ml de água destilada, assim, obtém-se a concentração de 1 mg/ml). O nicio de ação começa dentro de 10 a 30 minutos e dura de 2 a 4 horas. A hidralazina parenteral pode aumentar o risco de hipotensão materna (PA sistólica, 90 mmHg ou menos);^271^
- Nas raras circunstâncias em que o bolus de labetalol (não disponível no Brasil) , de hidralazina ou nifedipina oral (retard) administrados em doses apropriadas e sucessivas não controlarem os níveis tensionais, recomenda-se discutir intervenção com drogas consideradas de segunda linha;^267^
- **Nitroglicerina:** considerar como medicamento de escolha na pré-eclâmpsia associada com o edema agudo de pulmão (infusão intravenosa de 5 mg/min, aumentando gradualmente a cada 3 a 5 min até uma dose máxima de 100 mg/min);
- **Nitroprussiato de sódio:** deve ser considerado como opção preferencial para controle da pressão arterial em situações excepcionais, como hipertensão refratária ou hipertensão grave com risco de morte. O tratamento prolongado com nitroprussiato de sódio está associado ao risco fetal por intoxiacação pelo cianeto, produto metabólico do nitroprussiato de sodio; por isso, ele deve ser iniciado com 0,25 μg/kg/min até o máximo de 4 μg/kg/min, e por não mais de 4 h de infusão contínua.^275^

## 4.6. A Profilaxia da Crise Convulsiva na Pré-eclâmpsia – Eclâmpsia e a Terapêutica com Sulfato de Magnésio293,275,299-303

Desde a publicação dos resultados do The Collaborative Eclâmpsia Trial – Maggie Trial,^302^ o sulfato de magnésio (MgSO4)é considerado o medicamento de escolha para o tratamento da eclâmpsia iminente e da eclâmpsia.^299^ Estudos clinicos randomizados demonstram que o sulfato de magnésio é mais seguro e mais eficaz que a fenitoína, diazepam ou coquetel lítico (clorpromazina, prometazina e petidina) para prevenir convulsões recorrentes na eclâmpsia, além de ser de baixo custo, fácil de administrar e não causar sedação.^300-303^Portanto, o uso de sulfato de magnésio é altamente recomendado para casos de eclâmpsia iminente, eclâmpsia, síndrome HELLP (15% desses pacientes desenvolvem eclâmpsia) e pré-eclâmpsia com deterioração clínica e/ou laboratorial, incluindo hipertensão de difícil controle.^303^

A dose inicial, adequadamente administrada, não oferece riscos de intoxicação. Recomenda-se porém, a monitorização do reflexo patelar , a frequência respiratória e a diurese. Caso haja ausência do reflexo patelar, depressão respiratória (Frequência respiratória < 16 rpm) e diurese inferior a 25 ml/h recomenda-se suspender o MgSO4 por via intravenosa e dosar os seus níveis séricos.

A concentração terapêutica do íon magnésio varia de 4 a 7 mEq/L (4,8 a 8,4 mg/dl). O reflexo patelar fica abolido com 8 a 10 mEq/L, o risco de parada respiratória a partir de 12 mEq/L e cardíaca de 25 mEq/L. O gluconato de cálcio (1 g por via endovenosa – 10 ml a 10% – administrado lentamente) deve ser utilizado nos casos de sinais de intoxicação pelo magnésio. Na parada respiratória, além de gluconato de cálcio, deve-se proceder à intubação endotraqueal e ventilação mecânica. Nas pacientes com insuficiência renal (creatinina ≥ 1,2 mg/dl), a dose de manutenção deve ser a metade da dose recomendada. Deve-se interromper a infusão do sulfato de magnésio apenas se a diurese for inferior a 25 ml. Diante de valores dentro dos limites de normalidade, deve-se manter ou reiniciar o tratamento.^304^

A prevenção das crises convulsivas deve seguir as seguintes recomendações:

- **Dose de ataque**: (MgSO4 50% – ampola com 10 ml – contém 5 g de magnésio) – 4 a 6 g de MgSO_4_ por via intravenosa, em dose única (diluir 8 a 12 ml da solução a 50% em 100 ml de soro glicosado a 5% e ministrar, com bomba de infusão, em 30 min);
- **Dose de manutenção**: 1 a 2 g por hora, por via intravenosa (Diluir 10 de MgSO4 50% (1 ampola) em 490 ml de soro fisiológico a 0,9%. A concentração final terá 1 g/100 ml. Infundir a solução por via intravenosa na velocidade de 100 ml por hora em bomba de infusão contínua.

É preciso manter o MgSO4ão por 24 horas após o parto ou após a última convulsão. Nos casos de recorrência da crise convulsiva, administram-se mais 2 g do sulfato de magnésio por via endovenosa (bolus) e utiliza-se como manutenção a dose de 2 g/h. Se dois desses bolus não controlarem as convulsões, a droga de escolha será a difenil-hidantoína em seu esquema clássico para o tratamento de crises convulsivas. Recomenda-se ainda nesses casos a investigação de complicações cerebrais, principalmente hemorragias intracranianas.

Após as primeiras 24 horas de observação e avaliação, necessário decidir por conduta conservadora ou interrupção da gestação. O parto é a única intervenção que leva à resolução da pré-eclâmpsia e eclâmpsia. Recomenda-se que a conduta expectante seja somente até as 37 semanas de gestação. Após esta data gestacional ou se o diagnóstico de pré-eclâmpsia for realizado a termo, a resolução da gestação deverá ser indicada, reduzindo-se, assim, os riscos maternos, sem alterar os resultados perinatais.

### 4.6.1. Pontos-chaves

- Em mulheres com hipertensão gestacional, hipertensão preexistente sobreposta por hipertensão gestacional ou com lesão em órgãos alvo , recomenda-se o início do tratamento medicamentoso com a PAS ≥ 140 mmHg ou PAD ≥ 90 mmHg;
- A meta para o tratamento de pressão arterial na SHG deve ser para a PAS ≤ 140 e PAD = 80 a 85 mmHg. Nestes níveis os anti-hipertensivos devem ser ajustados;
- Metildopa, betabloqueadores (exceto o atenolol) e os BCC são recomendados como os medicamentos de escolha;
- Inibidores da ECA, BRA ou inibidores diretos da renina não são recomendados;
- A terapêutica com diuréticos geralmente é evitada porque o volume plasmático é reduzido em mulheres que desenvolvem pré-eclâmpsia;
- Considerar PAS ≥ 160 mmHg ou PAD ≥ 110 mmHg como uma emergência hipertensiva na mulher grávida, com indicação de imediata hospitalização;
- O sulfato de magnésio deve ser usado na prevenção e tratamento de convulsões em mulheres com hipertensão gestacional e pré-eclâmpsia com características graves ou eminência de eclâmpsia;
- Na emergência hipertensiva, as drogas indicadas são nifedipina oral, hidralazina intra-venosa e labetalol;
- Na pré-eclâmpsia associada ao edema pulmonar, a nitroglicerina (infusão) deve ser considerada;
- O parto é a intervenção que conduz à resolução da pré-eclâmpsia e eclâmpsia.

## 4.7. Prognóstico e Prevenção da Pré-eclâmpsia

Modelos com base em dados clínicos ou fatores de risco têm baixa sensibilidade na predição da pré-eclâmpsia. Em contrapartida, marcadores bioquímicos, por exemplo, fator pró-angiogênico, PIGF (derivado da placenta), quando em níveis baixos entre 11ª e 13ª semana de gestação ou o fator anti-angiogênico, tirosinoquinase 1 solúvel FMS like- sFlt-1, em níveis elevados, podem predizer a pré-eclâmpsia. Ambos não apresentam sensibilidade suficiente na presunção, contudo os estudos sobre a relação entre ambos (sFlt-1/PIGF), são promissores. No momento, não há nenhum teste laboratorial preditor disponível na prática clínica.^304^

A US com Doppler através da avaliação da pulsatilidade e resistência nas artérias uterinas, pode classificar a gestante como de risco para desenvolvimento de pré-eclâmpsia. A US com Doppler deve ser realizada entre 20 e 22 semanas, tem boa correlação com pré-eclâmpsia tardia (> 34 semanas) e RCIU. Por outro lado, a US com Doppler realizada no final do primeiro trimestre tem menor acurácia; porém, somada com a história clínica e as comorbidades, pode ser útil em identificar as gestantes de maior risco e selecionar aquelas com indicação de medidas de prevenção da pré-eclâmpsia.^305^

Inúmeras estratégias foram estudadas para reduzir a incidência de pré-eclâmpsia. Dieta, perda de peso, atividade física, vitaminas, antioxidantes, nitratos, dipiridamol, heparinas (BPM e HNF) e antiagregantes plaquetários foram investigados, e destes apenas a reposição de cálcio e o ácido acetilsalicílico (AAS) mostraram benefício.

A reposição de cálcio (1,5 a 2,0 g/dia) reduz o risco de pré-eclâmpsia de maneira efetiva na subpopulação com ingesta diária de cálcio abaixo de 600 mg/dia.^306^

O benefício do uso da aspirina (AAS) em baixa dose (entre 75 e 150 mg) na prevenção da pré-eclâmpsia^307^ foi demonstrado e, recentemente, sua recomendação foi incluída em diretrizes internacionais.^278,308,269^ Estudo^309^ controlado que inclui 1.776 pacientes, que utilizaram a dose diária de 150 mg de AAS iniciada entre 11 e 14 semanas, mostrou beneficio significativo na redução de eventos de pré-eclâmpsia, ratificando o efeito protetor do AAS em gestantes de alto risco.

A indicação precisa do AAS é para pacientes classificadas como de alto risco para pré-eclâmpsia (Tabela 27) e deve ser iniciada entre 12 e 16 semanas.

**Tabela 27:** – Recomendações para uso de ácido acetilsalicílico na profilaxia de pré-eclâmpsia

Nível de risco	Fator de risco	Recomendação
Alto	Pré-eclâmpsia prévia com desfecho fetal adverso Gestação multifetal HAS crônica Diabetes melito tipos 1 ou 2 Doença renal Doença autoimune (lúpus/SAAF)	Recomenda-se AAS em baixa dose para um ou mais desses critérios
Moderado	Nuliparidade Obesidade (IMC ≥ 30) História familiar de pré-eclâmpsia (mãe ou irmã) Idade ≥ 35 anos História obstétrica prévia ruim (PIG, prematuridade, baixo peso, mais de 10 anos de intervalo entre as gestações)	Considerar uso de AAS em baixa dose se paciente com mais de um fator de risco

*AAS: ácido acetilsalicílico. HAS: hipertensão arterial sistêmica; IMC: índice de massa corpórea; PIG: pequeno para idade gestacional; SAAF: síndrome do anticorpo antifosfolipídeo.*

### 4.7.1. Pontos-chaves

- A presunção da ocorrência de pré-eclâmpsia em pacientes de baixo risco é muito difícil e depende da avaliação conjunta de história clínica e USG doppler;
- A reposição de cálcio em pacientes com baixa ingesta reduz o risco de pré-eclâmpsia;
- O uso de AAS baixa dose em gestantes de risco moderado a alto reduz o risco de pré-eclâmpsia e deve ser idealmente iniciado entre 12 e 16 semana de gestação.

## 4.8. Hipertensão Arterial no Puerpério

A hipertensão arterial no puerpério é pouco estudada, pois ainda existe um conceito de que a retirada da placenta favorece a resolução da doença. De certo modo, a dequitação marca o momento em que o estímulo da produção de substâncias inflamatórias e vasoconstritoras cessa, levando a um retorno gradual da pressão arterial aos níveis prévios à gestação; contudo, as alterações inflamatórias e de vasoconstrição podem permanecer ainda por alguns dias no organismo materno.

### 4.8.1. Recomendações

De modo geral, a pressão arterial tende a estabilização e o alcance da normalidade dentro de cinco a sete dias, porém, nesse período, ainda há risco de complicações, principalmente nas pacientes com pré-eclâmpsia, além da possibilidade da própria pré-eclâmpsia e até mesmo a eclâmpsia ocorrer no puerpério.

A hipertensão no puerpério pode ser agravada ou prolongada no seu tempo por situações como sobrecarga de volume (hiper-hidratação) e uso de medicações para dor, como anti-inflamatórios não esteróides (vasoconstrição e retenção de sódio), além dos casos em que ocorre acidente vascular cerebral com vasoconstrição reativa e nas pacientes hipertensas crônicas sem diagnóstico prévio.

Nas puérperas com pré-eclâmpsia, pode ocorrer uma nova elevação da pressão arterial entre o 3º e o 6º dia de puerpério, provavelmente pela reabsorção do edema acumulado no terceiro espaço, que é bastante comum na síndrome da pré-eclâmpsia.^310^

O objetivo do tratamento é diminuir o risco de lesão de órgão-alvo (edema agudo dos pulmões, acidente vascular cerebral, dissecção de aorta, doença renal aguda) por emergência hipertensiva. Logo, puérperas com hipertensão leve a moderada (PAS < 160 mmHg e/ou PAD < 110 mmHg) que estejam assintomáticas podem ser acompanhadas sem medicação anti-hipertensiva.

No puerpério não há restrições ao uso da medicação anti-hipertensiva devendo-se priorizar aquelas que passem em quantidade menor pelo leite materno.

Revisão da Cochrane Library em 2013^311^ sugeriu que o uso da furosemida poderia ajudar no controle mais efetivo e abreviar o tempo de hospitalização das pacientes com pré-eclâmpsia. A recomendação para o uso do diurético é após o segundo dia, quando inicia a reabsorção do edema periférico. O site de consultas https://toxnet.nlm.nih.gov, revisa as publicações e atualiza as recomendações para o uso de fármacos durante o aleitamento.^312^

Os IECA captopril e enalapril, que são contraindicados durante a gestação, durante o aleitamento são permitidos por passarem em quantidades muito pequenas pelo leite materno. Quanto ao grupo dos BRA, não há ainda estudos suficientes para liberação do uso dessa classe de medicação. Dentre os BCC, o mais utilizado é a nifedipina, que também passa pouco pelo leite materno. O anlodipino e os demais BCC carecem de estudos para sua liberação sem restrições. Os betabloqueadores devem ser individualizados caso a caso e de forma geral são compatíveis com o aleitamento.

Os diuréticos, como hidroclorotiazida e furosemida, podem depletar o espaço intravascular e diminuir a produção de leite; por isso, devem ser usados em dose baixa. A espironolactona pode ser administrada sem restrição e pode ser indicada para pacientes com hipertensão resistente (hiperaldosteronismo primário).

Um estudo comparando captopril e clonidina para controle de hipertensão grave (PAS ≥ 180 mmHg e PAD ≥ 110 mmHg) verificou que não houve diferença significativa entre essas substâncias, apenas uma tendência de a clonidina ser melhor no terceiro dia do puerpério. Ambas foram consideradas efetivas e seguras para tratar puérperas com emergência hipertensiva.^313^

A alta hospitalar deve ser com nível de PAS < 160 mmHg e PAD < 110 mmHg, pelo menos durante 24 horas prévias, e o acompanhamento ambulatorial deve ser periódico com reavaliação em curto prazo, no máximo de uma a duas semanas após a alta.^314^

### 4.8.2. Pontos-chaves

- A hipertensão costuma melhorar nos primeiros cinco a sete dias, após o parto. Contudo, após esse período podem ocorrer complicações, inclusive pré-eclâmpsia/eclâmpsia;
- Devem ser priorizadas as medicações liberadas para o aleitamento;
- O seguimento ambulatorial é importante, visto que a maioria destas pacientes tem alta hospitalar em uso de medicação anti-hipertensiva.

## 4.9. Hipertensão na Gestação e Risco Cardiovascular Futuro

A pré-eclâmpsia é um fator de risco independente para doença arterial coronária, hipertensão crônica, doença vascular periférica e acidente vascular cerebral. Os mecanismos possíveis para aumento da doença cardiovascular (DCV) incluem as disfunções endotelial, vascular e metabólica encontradas durante a pré-eclâmpsia, que possuem uma ligação comum com outros fatores de risco tradicionais, como dislipidemia, obesidade, diabetes melitos e doença renal.

O estudo CHAMPS,^315^que inclui um número superior a 1.000.000 de mulheres sem DCV após a primeira gestação, mostrou um aumento do risco de revascularização miocárdica e internação por acidente vascular cerebral e doença vascular arterial periférica duas vezes maior nas pacientes que tiveram pré-eclâmpsia, hipertensão gestacional, ruptura ou infarto placentário.

Estudos^316^incluindo mais de 3 milhões de mulheres e quase 200.000 gestantes mostrou risco relativo aumentado de 3,7 para hipertensão arterial crônica; de 2,16 para doença isquêmica cardíaca e de 1,81 para acidente vascular cerebral, em pacientes que apresentaram pré-eclâmpsia.

Desse modo, a hipertensão durante a gestação deve ser encarada como marcador de risco cardiovascular futuro relacionado ao gênero. É preciso colocar na rotina o cuidado com a orientação das mulheres após o parto e intensificar o controle dos demais fatores modificáveis visando diminuir o risco cardiovascular desse grupo especial de mulheres.^317^

### 4.9.1. Pontos-chaves

- A pré-eclâmpsia é um fator de risco para doença arterial coronária, hipertensão crônica, doença vascular periférica e acidente vascular cerebral;
- Pacientes que apresentaram hipertensão arterial durante a gestação devem intensificar o controle dos demais fatores modificáveis visando diminuir o risco cardiovascular futuro.

## 5. Tratamento e Prevenção das Complicações Cardíacas

### 5.1. Arritmias Cardíacas

#### 5.1.1. Epidemiologia

As arritmias são complicações muito frequentes durante a gravidez, associadas ou não a doença cardíaca estrutural ou elétrica. A primeira manifestação pode ser na gestação ou pode ocorrer agravamento de arritmias preexistentes.^318^

A ocorrência de arritmias durante a gestação exige uma investigação com particular atenção em definir ou excluir uma lesão estrutural ou elétrica do coração, conduta fundamental na determinação do tratamento e prognóstico da paciente.^52,318^

O estudo em gestantes hospitalizadas mostrou que: 60% das arritmias corresponderam a bradicardia ou taquicardia sinusal; 19%, a extrassístoles supraventriculares ou ventriculares; 14%, a taquicardias supraventriculares (TSV); 5%, a TV ou FV; e 2%, a outros distúrbios.^319^

A FA e a taquicardia paroxística supraventricular (TPSV) são as TSV sustentadas mais frequentemente diagnosticadas na gestação; bradiarritmias, distúrbios de condução, outras taquicardias atriais, TV e FV são relativamente raras.^320^

Os admitidos riscos dos fármacos antiarrítmicos sobre a organogênese e o desenvolvimento fetal devem ser considerados na prescrição durante a gravidez, uma vez que a maioria das arritmias diagnosticadas não requer tratamento específico. Contudo, as arritmias recorrentes ou persistentes que determinam sintomas importantes ou repercussão hemodinâmica devem ser igualmente tratadas como em mulheres fora da gestação.^321^

O risco inerente à radiação ionizante utilizada para a realização da ablação por cateter pode ser minimizado com o mapeamento eletroanatômico e, em alguns casos de implantes de dispositivos (marca-passo, CDI e ressincronizador), com o uso do ECO bidimensional.^322^

#### 5.1.2. Apresentação Clínica

Palpitações ocorrem com frequência na gravidez, podendo estar relacionadas a arritmias ou ser consequentes às alterações hemodinâmicas da gestação. A avaliação diagnóstica das palpitações em gestantes não difere da realizada em mulheres fora da gestação e tem demonstrado que, em apenas 10% dos casos, as palpitações estão associadas à presença de arritmias.^323^

Bradicardia sinusal sintomática é rara e geralmente está associada à síndrome da hipotensão supina gestacional, cujo tratamento é a colocação da gestante em decúbito lateral esquerdo. Síncope ligada a bloqueios atrioventriculares é igualmente pouco frequente, e o BAVT congênito, principalmente supra-hissiano, com QRS estreito, apresenta boa evolução na gestação. A MCS, rara durante a gestação, apresenta maior risco de ocorrência em mulheres com TV associada a cardiopatia estrutural e, na gestação e no puerpério, naquelas com canalopatias (especialmente portadoras da síndrome do QT Longo).^319,320^

#### 5.1.3. Risco Materno-fetal

Os distúrbios sustentados do ritmo cardíaco podem levar ao comprometimento hemodinâmico materno, ao risco de tromboembolismo e à MCS. Podem também comprometer o desenvolvimento fetal, determinando baixo peso, parto prematuro, anormalidades fetais e mais indicações de parto cesárea. Por essa razão, tais distúrbios devem ser diagnosticados e adequadamente tratados.

A classificação da OMS modificada quanto ao risco materno considera as extrassístoles supraventriculares e ventriculares isoladas como classe I (na qual não há risco detectável de aumento da mortalidade materna, mas há aumento discreto na morbidade materna); as arritmias supraventriculares estão na classe II (na qual há aumento discreto na mortalidade e moderado na morbidade materna); e as TV são incluídas na classe III (na qual há aumento significativo na mortalidade e morbidade maternas).^324^

As recomendações atuais propõem que as arritmias durante a gestação sejam classificadas, de acordo com o potencial comprometimento hemodinâmico, em: Baixo risco para MCS (TPSV e FA com estabilidade hemodinâmica, TV idiopática, síndrome do QT longo de baixo risco, síndrome de Wolff-Parkinson-White); Médio risco para MCS (TSV instável, TV em pacientes com cardiopatia estrutural, síndrome de Brugada, síndrome do QT Longo e TV polimórfica catecolaminérgica de risco moderado); Elevado risco para MCS (TV instável em pacientes com cardiopatia estrutural, *Torsade de pointes* em pacientes com síndrome do QT Longo, síndrome do QT curto, TV polimórfica catecolaminérgica de alto risco).^52,320^

O planejamento do parto no grupo de baixo risco deve ter participação do cardiologista, e o parto deve ser de indicação obstétrica. No grupo de médio risco, o parto se mantém de indicação obstétrica; entretanto, a equipe multiprofissional que acompanha a gestante deve incluir um eletrofisiologista e, durante o parto, deve estar preparada para a utilização de fármacos como adenosina e betabloqueadores, bem como para o uso de um cardioversor-desfibrilador (CD). No grupo de alto risco há indicação de parto cesárea, durante o qual é preciso estar preparado para a utilização do CD e de antiarrítmicos, além dos betabloqueadores; nesse grupo, a gestante poderá necessitar de UTI Não pós-parto.^52^

#### 5.1.4. Tratamento

O tratamento das arritmias em gestantes é semelhante ao realizado em mulheres não gestantes.^325^ De acordo com a indicação, podem ser utilizados os seguintes métodos: cardioversão elétrica, manobras vagais, fármacos antiarrítmicos, implante de dispositivos (marca-passo, CDI, ressincronizador cardíaco) e ablação por cateter (Tabela 28). O tratamento de arritmias cardíacas na sala de emergência será discutido no tópico 5.7.

**Tabela 28:** – Conduta na taquicardia supraventricular aguda

Recomendação
Cardioversão elétrica imediata como primeira escolha para TSV com instabilidade hemodinâmica materna e para FA em gestantes com síndrome de pré-excitação ventricular
Manobras vagais; caso sejam ineficientes, adenosina para reversão aguda da TPSV
Betabloqueadores endovenosos (metoprolol, propranolol) para reversão aguda da TPSV
Verapamil endovenoso para reversão aguda da TPSV quando adenosina e betabloqueadores não são efetivos ou estão contraindicados
Procainamida endovenosa para reversão aguda das TSV
Flecainida ou ibutilida para reversão aguda do flutter e FA em gestantes com coração estruturalmente normal
Amiodarona para reversão aguda das TSV potencialmente graves quando outras terapias não são efetivas ou estão contraindicadas

*FA: fibrilação atrial; TPSV: taquicardia paroxística supraventricular; TSV: taquicardias supraventriculares.*

A carência de estudos clínicos randomizados faz com que a contraindicação ou não ao método seja pautada em dados experimentais em animais, nos registros sobre os efeitos colaterais dos medicamentos usados na prática clínica e em relatos ou séries de casos. Esse fato determina que tais tratamentos devem ser usados apenas quando há comprometimento hemodinâmico materno e fetal em decorrência da arritmia e/ou quando há risco de MCS materna na gravidez e no puerpério. Quando possível, todos os tratamentos devem ser postergados para o segundo ou terceiro trimestre (evitando o período de organogênese); no caso dos fármacos, sempre utilizá-los na menor dose e pelo menor tempo necessário.

A cardioversão elétrica sincronizada, indicada para a reversão de TSV instáveis (FA, flutter atrial, taquicardias atriais, TPSV) e TV instáveis ou estáveis (estas, quando na presença de cardiopatia), é segura em todas as etapas da gestação, não comprometendo o fluxo sanguíneo para o feto. A posição anterolateral das pás deve ser a escolhida, com a colocação da pá lateral abaixo do seio materno esquerdo e monitoramento do ritmo fetal.^326^

As manobras vagais, como manobra de Valsalva, massagem do seio carotídeo, imersão da face em água a 10ºC ou colocação de uma toalha molhada na face, podem ser utilizadas com segurança para reversão aguda de uma TPSV (determinadas por reentrada nodal ou por uma via acessória, sendo esta última característica da síndrome de Wolff-Parkinson-White) na gestação.^52,325^ A manobra de Valsalva costuma ser mais efetiva que a massagem do seio carotídeo. A compressão do globo ocular é potencialmente perigosa e nunca deve ser usada.

Quando as manobras vagais falham na tentativa de reversão aguda da TPSV, a adenosina (6 mg iniciais; dose máxima de 24 mg) é a substância de primeira escolha para gestantes, pois não há evidências de efeitos negativos para o feto, e os efeitos na mãe (desconforto torácico e rubor) são de curta duração.^52,325,327^ Mesmo não sendo fármacos da primeira escolha, betabloqueadores (metoprolol, propranolol), verapamil, procainamida e amiodarona também podem ser utilizados nessa tentativa de reversão.

No manuseio agudo das demais arritmias sustentadas supraventriculares (FA, flutter, taquicardia atrial), betabloqueadores, verapamil e digitálicos são indicados para o controle da resposta ventricular, e os demais fármacos, incluindo flecainida, ibutilida e propafenona, podem ser utilizados para a reversão aguda ao ritmo sinusal.^52,325,327^ Na reversão para ritmo sinusal das TV idiopáticas estáveis, indicam-se betabloqueadores, sotalol, flecainida, procainamida e lidocaína. O marca-passo ventricular com frequência acima da TSV é uma alternativa a ser considerada (Tabela 29).

**Tabela 29:** – Conduta na taquicardia supraventricular crônica

Recomendação
Betabloqueadores ou verapamil para a prevenção de TPSV em gestantes sem pré-excitação ao ECG
Betabloqueadores para o controle da resposta ventricular em gestantes com FA ou taquicardia atrial
Flecainida ou propafenona para prevenção de TPSV em pacientes com síndrome de Wolff-Parkinson-White
Flecainida, propafenona ou sotalol para prevenção de TPSV, taquicardia atrial e FA quando não há resposta aos betabloqueadores
Digoxina ou verapamil para o controle da frequência cardíaca nas taquicardia atrial e FA quando não há resposta aos betabloqueadores
Ablação por cateter com a utilização de sistemas de mapeamento eletroanatômico para as TSV mal toleradas ou refratárias ao tratamento com antiarrítmicos

*ECG: eletrocardiograma; FA: fibrilação atrial; TPSV: taquicardia paroxística supraventricular; TSV: taquicardias supraventriculares.*

O tratamento permanente das TSV e TV deve ser igual ao realizado fora da gestação, salvo as restrições ao uso da amiodarona pelas implicações ao feto (hipotireoidismo, hipertireoidismo, retardo do crescimento e prematuridade). Deve ser considerado que bradicardia e hipoglicemia fetal e baixo peso ao nascer podem estar associados ao uso crônico de betabloqueadores; contudo, esse fato parece ser dose-dependente. A prescrição de betabloqueador deve contemplar o benefício da sua utilização excedendo os riscos, exceto o atenolol, que apresenta um reconhecido efeito teratogênico e, portanto, deve ser evitado durante a gestação. Também há relatos de teratogenicidade com o uso de diltiazem. O sotalol não deve ter uso permanente em gestantes com síndrome de Wolff-Parkinson-White para prevenção dos episódios de TPSV (Tabelas 30 e 31).^52,325,327^

**Tabela 30:** – Conduta na taquicardia ventricular aguda

Recomendação
Cardioversão elétrica imediata como primeira escolha para gestantes com TV sustentada, com e sem instabilidade hemodinâmica
Betabloqueadores, sotalol, flecainida, procainamida ou marcapassamento ventricular com frequência acima da TV (*overdrive ventricular pacing*) para reversão de TV sustentada monomórfica idiopática, hemodinamicamente estável

*TV: taquicardia ventricular.*

**Tabela 31:** – Conduta na taquicardia ventricular crônica

Recomendação
Betabloqueadores em gestantes com síndrome do QT longo e com TV polimórfica catecolaminérgica durante a gestação e o puerpério, incluindo as que estão amamentando
Implante de CDI deve ser realizado antes da gestação; caso seja indicado durante a mesma, deve ser realizado com um mínimo de radiação (guiado por ecocardiograma, por exemplo) e, preferencialmente, após o primeiro trimestre
Betabloqueadores ou verapamil para prevenção dos episódios de TV sustentada idiopática
Sotalol ou flecainida para prevenção dos episódios de TV sustentada idiopática, se outras substâncias não forem efetivas
Ablação por cateter, com a utilização de sistemas de mapeamento eletroanatômico, para as TV sustentadas mal toleradas ou refratárias ao tratamento com antiarrítmicos

*CDI: cardioversor desfibrilador implantável; TV: taquicardia ventricular.*

De maneira geral, a ablação por cateter e o implante de dispositivos, quando possível, devem ser realizados fora do período gestacional, pelos riscos inerentes a tais procedimentos, incluindo aquele relacionado à exposição à radiação ionizante. A ablação por cateter durante a gestação tem sido indicada apenas para gestantes que apresentam taquicardias recorrentes ou persistentes com grave comprometimento hemodinâmico e que não respondem aos tratamentos usuais. Há relatos de casos e de pequenas séries de pacientes com TSV submetidas à ablação por cateter com a utilização de estratégias de mapeamento que utilizam cada vez menos radiação ionizante, aumentando a segurança materna e fetal quanto aos riscos futuros dessa exposição.^328^ Não há relatos de ablação por cateter de TV até o momento.

Mulheres portadoras de marca-passo e CDI apresentam boa evolução durante a gestação; entretanto, as complicações inerentes à doença cardíaca subjacente e ao dispositivo podem estar presentes, determinando a necessidade de um acompanhamento especializado.^329^ Caso sejam absolutamente imprescindíveis, tais dispositivos podem ser implantados com segurança durante a gestação, sem ou com um mínimo de fluoroscopia.^330^

A reprogramação dos dispositivos (marca-passo e CDI) deve ser realizada antes do parto cesárea, devido a interferência no funcionamento do dispositivo causada pelo bisturi elétrico. Caso o parto cesárea seja de emergência, coloca-se um imã sobre a loja do gerador de marca-passo durante o uso do bisturi elétrico, com colocação da placa do cautério longe da região torácica. Para parto vaginal não é necessária tal reprogramação.

Em gestantes com FA ou flutter atrial crônicos não associados a doença cardíaca estrutural, deve ser realizada a estratificação do risco para fenômenos tromboembólicos, por meio da utilização do escore de risco CHA_2_DS_2_-VASc,^331^ incluindo a indicação de anticoagulação quando esse escore for maior ou igual a 2. É controverso se o estado de hipercoagulabilidade aumenta o escore de riscos para a indicação de anticoagulantes na gestação. Deve ser enfatizado que os NOACS (dabigatrana, rivaroxabana, apixabana, edoxabana) não devem ser usados em gestantes.^332,333^

#### 5.1.5. Pontos-chaves

- A conduta inicial em arritmias durante a gravidez é a investigação de lesão cardíaca estrutural;
- Arritmias “novas”, na ausência de lesão cardíaca estrutural, devem ser tratadas de acordo com os sintomas maternos ou com a complexidade da arritmia;
- O sistema de Holter 24 horas é o exame essencial na decisão terapêutica;
- O implante de dispositivos (marca-passo, CD) e ablação de radiofrequência com mapeamento eletroanatômico são seguros na gravidez e devem ser indicados perante a refratariedade farmacológica;
- Os dispositivos como marca-passo, CD e ressincronizador cardíaco devem ser reprogramados após o parto cesárea.

## 5.2. Tromboembolismo

### 5.2.1. Epidemiologia

O evento tromboembólico venoso é importante causa de mortalidade materna e, potencialmente, passível de prevenção.^131,334^ É a principal causa direta de morte materna nos países desenvolvidos e no Brasil; no ano de 2013,^335^ foi a sexta causa, atrás de hemorragia grave, hipertensão na gestação, infecção, complicações do parto e abortamento. Além disso, é uma relevante causa de morbidade pela síndrome pós-trombótica. O diagnóstico tardio, o tratamento postergado ou inadequado e a profilaxia imprópria são responsáveis por cerca de 3,5% das mortes maternas.^336^

O tromboembolismo compreende tanto a trombose venosa profunda (TVP) quanto o TEP, sendo que 75 a 80% dos casos de tromboembolismo associados à gravidez são de TVP e 20 a 25%, de TEP. A verdadeira incidência da doença associada à gestação é desconhecida, mas parece estar entre 7 e 25/10.000 gestações, e a impressão clínica é de que as chances estão aumentadas de 5 a 10 vezes nesse período. O risco parece ser maior no terceiro trimestre, mas é elevado desde o primeiro. No puerpério, ele chega a mais de 20 vezes o risco de uma mulher não grávida e se estende de maneira decrescente até 6 semanas do pós-parto. Entretanto, estudos recentes têm demonstrado um aumento do risco de tromboembolismo em até 180 dias pós-parto em grupos de pacientes com alguns fatores de risco obstétricos, entre eles o parto cesárea e a gestação gemelar.^131,334,335^

### 5.2.2. Fatores de Risco

A Tabela 32 lista os fatores de risco associados a tromboembolismo na gestação, relacionando os preexistentes, os transitórios e os obstétricos. Sugere-se que a presença de dois ou mais destes fatores aumenta mais ainda o risco da doença; entretanto, é a história de trombose prévia o fator de risco individual mais importante. A recorrência de trombose nesse período está aumentada em 3 a 4 vezes, correspondendo a 15 a 25% de todos os caso de tromboembolismo na gestação.^336,337^

**Tabela 32:** – Fatores de risco para tromboembolismo venoso na gestação

Fatores preexistentes	Fatores transitórios	Fatores obstétricos
1. Tromboembolismo prévio	1. Gestação	**Antenatal:**
2. Trombofilias	2. Hiperemese gravídica	1. Reprodução assistida
3. História familiar de tromboembolismo	3. Desidratação	2. Gravidez múltipla
4. Comorbidades: LES, síndrome nefrótica, drepanocitose, câncer, paraplegia	4. Síndrome de hiperestimulação ovariana	3. Pré-eclâmpsia
5. Diabetes melito	5. Infecção	**Parto:**
6. Doenças inflamatórias (especialmente intestinal)	6. Imobilidade	1. Trabalho de parto prolongado
7. Idade acima de 35 anos	7. Viagem de mais de 4 horas	**Cirúrgicos:**
8. Obesidade		2. cesariana, esterilização pós-parto
9. Tabagismo		3. Natimorto
10. Varizes dos membros inferiores		4. Fórceps
11. Paridade ≥ 3		**Pós-parto:**
12. Passado de natimorto		1. Hemorrragia pós-parto
13. Parto pré-termo		2. Hemotransfusão

*LES: lúpus eritematoso sistêmico.*

### 5.2.3. Trombofilias

Trombofilia compreende um estado de hipercoagulabilidade congênita ou adquirida. O problema isolado, mesmo no contexto de uma gravidez, não resulta necessariamente na ocorrência de tromboembolismo,^338^e a raridade de tromboembolismo na gravidez e a elevada incidência de trombofilias hereditárias não justificam o rastreamento sistemático dessa doença.

A trombose venosa é uma doença poligênica de penetração incompleta, tornando o aconselhamento com base genética incerto. O risco de tromboembolismo associado a diferentes trombofilias e sua prevalência na população geral estão na Tabela 33.

**Tabela 33:** – Risco de tromboembolismo venoso associado a diferentes trombofilias

Fator	Prevalência na população geral (%)	Risco na gravidez (%) (sem história prévia)	Risco na gravidez (%) (com história prévia)	Percentual de todos os tromboembolismos
Fator V Leiden heterozigoto	1 a 15	0,5 a 3,1	10	40
Fator V Leiden homozigoto	< 1	2,2 a 14	17	2
G20210A heterozigoto	2 a 5	0,4 a 2,6	> 10	17
G20210A homozigoto	< 1	2,0 a 4,0	> 17	0,5
Fator V Leiden/G20210A heterozigoto	0,01	4,0 a 8,2	> 20	1 a 3
Deficiência de antitrombina	0,02	0,2 a 11,6	40	1
Deficiência de proteína C	0,2 a 0,4	0,1 a 1,7	4 a 17	14
Deficiência de proteína S	0,03 a 0,13	0,3 a 6,6	0 a 22	3

*G20210A: mutação do gene da protrombina.*

O rastreamento das trombofilias tem valor limitado em gestantes com tromboembolismo agudo porque não modifica a conduta clínica. Assim, a pesquisa de trombofilia durante a gestação é recomendada nas seguintes situações,^339^ de acordo com as classes de evidências:

- Fundamentada no risco clínico (classe IB);
- História familiar (parentes de primeiro grau) de tromboembolismo sem causa detectável ou ocorrido durante exposição hormonal, ou fator de risco menor, ou ainda em idade abaixo de 50 anos deve ser pesquisado (classe IIC);
- Tromboembolismo com fator de risco transitório menor, como viagem (classe IIC).

A pesquisa de trombofilia não é recomendada nas seguintes situações:

- Tromboembolismo prévio sem causa aparente (IB) e tromboembolismo relacionado ao uso hormonal ou em gestação anterior (classe IIC) requerem a indicação da tromboprofilaxia;
- História pessoal da doença com fator de risco transitório maior (fratura, cirurgia, imobilidade prolongada) (classe IIB);
- História obstétrica de perdas fetais recorrentes, placenta prévia, RCIU e pré-eclâmpsia.

### 5.2.4. Diagnóstico

O diagnóstico final pode ser prejudicado pelos sinais e sintomas inerentes à gravidez normal, tais como edema, dor em membros inferiores ou dor torácica, palpitação precordial e dispneia. Ainda assim, a clínica é substrato essencial para a busca do diagnóstico conclusivo, porque ainda não há um teste de triagem suficientemente sensível para definir a situação. Além disso, a maioria dos estudos que avaliaram exames de diagnósticos por imagem de tromboembolismo e fluxograma para diagnóstico excluiu gestantes por preocupação com a segurança materno-fetal.

***5.2.4.1. Trombose Venosa Profunda***

O diagnóstico embasado no quadro clínico (anamnese e exame clínico) é preocupante, pois implica a necessidade ou não da terapêutica anticoagulante permanente durante a gestação. Essa situação requer exames subsidiários para a conclusão do diagnóstico, que devem ser agilizados porque a morte súbita não é incomum em gestantes com sinais e sintomas compatíveis com a doença.

Escores de risco estruturados para classificar uma gestante como de risco baixo, intermediário ou alto para TVP, como o de Wells, não foram validados na gestação. Já a regra de LEFT foi proposta como específica para a predição da chance de TVP na gravidez e parece promissora. Se nenhuma dessas variáveis estiver presente, o valor preditivo negativo parece ser de 100%, mas esse método ainda deve ser validado em maiores estudos prospectivos.^340,341^

As variáveis consideradas nos escores de risco para TVP são:

- Apresentação da trombose em membro inferior esquerdo;
- Diferença ≥ 2 cm na circunferência da panturrilha (edema);
- Apresentação no primeiro trimestre da gravidez.

A Tabela 34 lista os exames complementares utilizados para o diagnóstico da TVP, as sensibilidades, especificidades e suas vantagens e desvantagens.

**Tabela 34:** – Exames utilizados para o diagnóstico de trombose venosa profunda

Exames	Acurácia	Vantagens	Desvantagens
Exame físico	S – 25 a 35% E – 30 a 50%	Inócuo, pode sugerir outros diagnósticos	Nenhuma
Dosagem do dímero-D	S – 100% E – 60%	Excelente valor preditivo negativo**	Deve ser associado com US
US de compressão/duplex scan	S – 96% para veias proximais E – 98%	Baixo custo Fácil repetição	nenhuma
AngioRM	S – 91,5%* E – 94,8%*	Trombose pélvica e de veias ilíacas	Custo
AngioTC venosa (venografia por TC)	S – 95,5%* E – 95,2%*	Pode ser realizado junto com angioTC pulmonar	Custo Uso de contraste Radiação

E: especificidade; S: sensibilidade. RM: ressonância magnética; TC: tomografia computadorizada. US: ultrassonografia. * Dados de metanálise de estudos com grande heterogeneidade. ** Não validado na gestação.

***5.2.4.2. Dímero D***

A dosagem do dímero-D está presente no algoritmo clássico para diagnóstico do tromboembolismo; contudo, durante a gravidez, esse marcador perde sua acurácia no diagnóstico do TEP, uma vez que sofre aumento em cerca de 40% a cada trimestre, no puerpério e em complicações como pré-eclâmpsia e descolamento de placenta.^342^ Essas incertezas influenciam na discordância sobre o uso do dímero-D no algoritmo diagnóstico de tromboembolismo na gestação.^336,340,343^

***5.2.4.3. Ultrassonografia Venosa***

Uma abordagem prática diante da suspeita de TVP se inicia com a utilização da US de compressão do membro acometido. A análise da compressibilidade das veias por meio desse exame apresenta sensibilidade de 96% e especificidade de 98% para o diagnóstico de TVP acima do joelho, e um pouco menor para aquelas abaixo do joelho, embora, mesmo nestas tenham chance diagnóstica substancial. O conhecimento de que a TVP frequentemente se apresenta nas veias proximais, mas pode ser isolada nas veias ilíacas, pode limitar a capacidade de exclusão da TVP apenas com a US de compressão em gestantes sintomáticas. Uma vez que as manobras de compressão não podem ser realizadas nas veias ilíacas, um trombo na veia ilíaca é diagnosticado por visualização direta de massa ecogênica intraluminal ou ausência de fluxo venoso espontâneo usando o Doppler.

**Figura 9:**
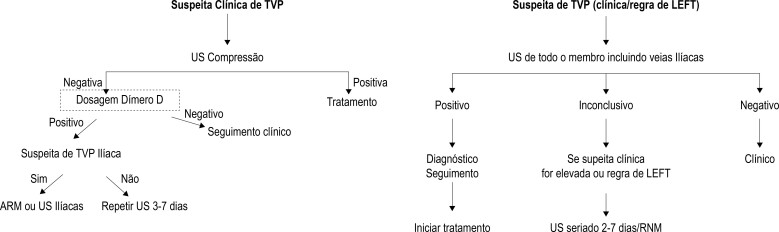
**–** Fluxograma utilizado para pesquisa de trombose venosa profunda (TVP) na gestação. US: ultrassonografia; ARM: antirressonância magnética RM: ressonância magnética.

Se a US é positiva, confirma o diagnóstico, e o tratamento é iniciado imediatamente; caso seja negativa e a paciente permaneça com sintomas, deve-se repetir o exame a cada 3 a 7 dias e iniciar o tratamento quando o diagnóstico se confirmar. A Figura 9 mostra dois fluxogramas para o diagnóstico de TVP na gestação: uma US venosa de compressão a partir das veias femorais e a utilização do dímero-D para avaliar a necessidade de investigação do território ilíaco; e uma US venosa completa da perna, incluindo a avaliação da veia ilíaca.

***5.2.4.4. Ressonância Magnética de Veias Ilíacas***

Quando o quadro clínico sugere trombose ilíaca isolada (edema de todo o membro, com ou sem dor em flanco, nádegas ou lombar), situação em que a US não é de boa resolução, deve-se utilizar a ressonância magnética. Ela pode ser empregada para diagnosticar TVP envolvendo veias ilíacas durante a gravidez, mas depende da perícia do examinador.^336,340,341^

***5.2.4.5. Tromboembolismo Pulmonar***

No momento, a abordagem para o diagnóstico do TEP na gestação é incerta e necessita de maiores estudos. Cerca de apenas sete diretrizes consideraram o diagnóstico do TEP na gestação, e as orientações são muito discordantes em relação ao uso de regras de predição de risco, uso da dosagem do dímero-D e escolha de métodos de imagem. A maioria delas não usa a dosagem de dímero-D no algoritmo diagnóstico para TEP. Em relação à US, algumas utilizam inicialmente a busca do diagnóstico de TVP; porém, como sua positividade é de apenas 20 a 40% na TEP, se ela for negativa, o diagnóstico tem de ser confirmado por outros métodos de imagem.

Os exames de eleição para o diagnóstico de TEP são cintilografia pulmonar de ventilação e perfusão (V/Q) ou ATCP; entretanto, ambos os testes carreiam risco de exposição materna e fetal a radiação. A cintilografia pulmonar V/Q expõe o feto a maior dose de radiação do que a ATCP; assim, se a radiografia de tórax for normal, considera-se apenas a cintilografia de perfusão, reduzindo a dose de radiação. A cintilografia V/Q também expõe a criança a maior risco de neoplasia, e a ATCP expõe a mãe a uma dose maior de radiação, levando a aumento pequeno, mas significativo, do risco de câncer de mama (1 caso em 280.000 *versus* menos de 1 em 1.000.000).

A decisão entre realizar V/Q ou ATCP é divergente. A maioria das recomendações indica a cintilografia V/Q como primeira escolha, especialmente a de perfusão na presença de radiografia de tórax normal. Outras, contudo, recomendam usar a ATCP com baixas doses para o diagnóstico de TEP, embora resultem em maior proporção de resultados inconclusivos na gestação. Cerca de 80% das cintilografias são diagnósticas, isto é, 70% são normais e 5 a 10% são de alta probabilidade. Na Tabela 35 encontra-se a dose de radiação absorvida nos testes diagnósticos para TEP na gravidez.^131,340^

**Tabela 35:** – Radiação absorvida estimada em procedimentos usados para diagnosticar tromboembolismo pulmonar

Teste	Radiação fetal estimada (mSv)	Radiação materna estimada na mama (mSv)
Radiografia de tórax	< 0,01	0,01
**Cintilografia de perfusão pulmonar com Tecnécio 99m:**		
Baixa dose (40 MBq)	0,11 a 0,20	0,28 a 0,50
Alta dose (200 MBq)	0,20 a 0,60	1,20
Cintilografia de ventilação pulmonar	0,10 a 0,30	< 0,01
Angiotomografia pulmonar	0,24 a 0,66	10 a 70

*mSv: milisievert.*

Pregnancy-Adapted YEARS Algorithm^334^ foi aplicado para o diagnóstico de TEP numa população de gestantes e mostrou que, na ausência de fatores como trombose venosa profunda, hemoptise, TEP como diagnóstico mais provável e dímero D não superior a 1000 ng/ml, o diagnóstico de TEP pode ser descartado e, consequentemente, a angiotomografia de tórax poderia ser evitada em 32 a 65% das pacientes.

***5.2.4.6. Diagnóstico Diferencial***

**Figura 10:**
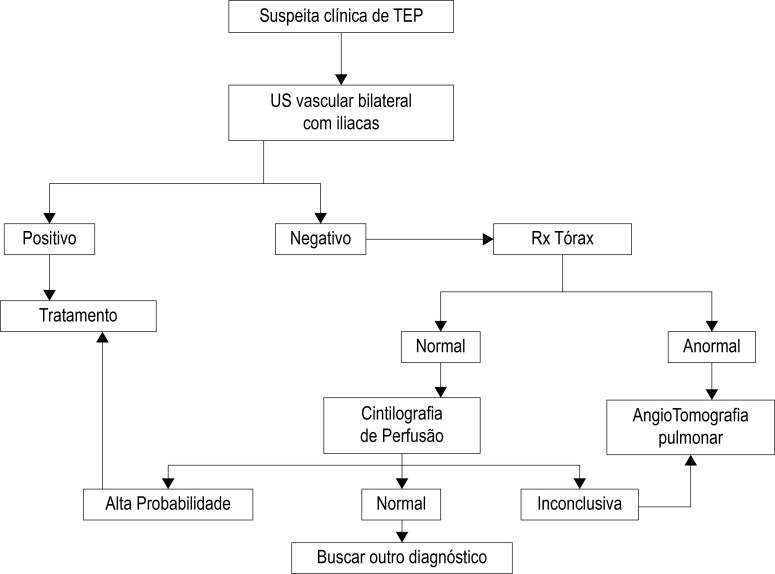
**–** Fluxograma para a investigação diagnóstica do tromboembolismo pulmonar na gestação. TEP: tromboembolismo pulmonar; Rx: radiografia.

O diagnóstico diferencial do TEP é amplo, pois a embolia pulmonar tem manifestações clínicas semelhantes às de pneumonia, IC e IAM. Por isso, é prudente descartar a presença de embolia pulmonar coexistente aos quadros com manifestação pneumônica. Do ponto de vista periférico, a TVP de membros inferiores deve ser diferenciada das doenças osteomusculares, tais como tendinite, distensão muscular, cisto poplíteo, aneurisma de poplítea, hematoma, celulite, linfangite e síndrome pós-trombótica (Figura 10).

### 5.2.5. Tratamento

***5.2.5.1. Abordagem Geral***

Diante de forte suspeita clínica de tromboembolismo, a anticoagulação plena e permanente deve ser iniciada antes da confirmação do diagnóstico, exceto quando ela for contraindicada. A heparina é o anticoagulante preferível, enquanto os “novos” anticoagulantes orais, como dabigatrana, rivaroxabana e apixabana, não estão liberados para uso na gestação e no aleitamento materno. Em casos de alergia ou trombocitopenia induzida pela heparina, o fondaparinux pode ser indicado e parece ser seguro no segundo e terceiro trimestres da gravidez.

***5.2.5.2. Uso da Heparina***

As HBPM e a HNF intravenosa são as opções no tratamento da TEP na gestação. A HBPM é fácil de ser utilizada e parece ser mais segura e eficaz do que a HNF, com dados extrapolados de estudos que não incluíram a gestação. A HNF intravenosa é indicada em pacientes com risco aumentado de sangramento ou hipotensão persistente na vigência de TEP. O uso prolongado da heparina, ou seja, superior a sete semanas, associa-se ao risco de osteoporose, hemorragia, reações alérgicas, necrose de pele e trombocitopenia, sendo menos frequente com o uso das HBPM. A suspensão está indicada diante da queda na contagem de plaquetas abaixo de 150.000 ou equivalente a 50% da contagem inicial. Nesse caso, a substituição pelo fondaparinux, embora controversa, pode ser indicada.

A anticoagulação deve ser continuada durante toda a gravidez e, pelo menos, nas seis primeiras semanas pós-parto. A contagem de plaquetas deverá ser realizada diariamente na busca de trombocitopenia nos primeiros 3 dias de tratamento e, depois, semanalmente.

***5.2.5.2.1. Doses Recomendadas***

- HBPM subcutânea: dalteparina 200 unidades/kg/dia ou 100 unidades/kg/12 em 12 h, ou enoxaparina 1 mg/kg/12 e 12 h. A dose de heparina deve ser controlada pelo nível do fator antiXa na faixa terapêutica entre 0,6 e 1,0 IU/ml quando aplicada a cada 12 h, e na faixa de 1 a 2 UI/ml quando aplicada em dose diária;
- HNF intravenosa: bólus de HNF de 80 unidades/kg seguido de infusão a 18 unidades/kg/hora, ajustada a cada 6 h para manter TTPa entre 1,5 e 2,5 vezes o basal. A estabilização da faixa terapêutica permite o controle diário do TTPa;
- HNF subcutânea: é razoável iniciar com 17.500 UI/12 e 12 h, ajustada a cada 6 h para manter TTPa entre 1,5 e 2,5 vezes o controle. A estabilização da faixa terapêutica permite o controle diário do TTPa.

***5.2.5.2.2. Trabalho de Parto e Parto***

O planejamento do parto de pacientes anticoaguladas exige o envolvimento de equipe multiprofissional, pois riscos de sangramento e de trombose devem ser ponderados nas fases do trabalho de parto, do parto e do puerpério. Nos casos de trabalho de parto espontâneo, a heparina deve ser imediatamente suspensa; no parto programado induzido ou cesárea, a HBPM deve ser suspensa 24 h antes, conduta que possibilita a anestesia neuroaxial. Em pacientes em que se julga arriscado suspender a heparina por 24 h, ela deve ser substituída pela HNF intravenosa que deverá ser interrompida 4 a 6 h antes do parto. A anestesia neuroaxial pode ser realizada quando o TTPa retornar ao normal. No caso de parto sabidamente prematuro (trigemelaridade, ruptura prematura de membranas, dilatação cervical significativa, pré-eclâmpsia, RCIU), deve-se descontinuar a HBPM ou a HNF subcutânea nas 36 semanas e trocar por HNF intravenosa.

Na ocorrência de parto em pacientes sob anticoagulação plena, é previsto maior sangramento no intraparto e no puerpério; além disso, o risco de hematoma espinhal contraindica a anestesia neuroaxial. Nesse sentido, sugere-se o uso da ocitocina no terceiro estágio do trabalho de parto.^342^

***5.2.5.2.3. Puerpério***

A heparina deve ser reiniciada 12 h após um parto cesárea ou 6 h após parto vaginal, se for assegurado que não há sangramento significativo. A varfarina, quando indicada, deve ser iniciada no segundo dia pós-parto junto com a heparina, até o alcance do INR entre 2 e 3 UI. É imprescindível que a paciente esteja em uso de heparina ao ser iniciado o anticoagulante oral, pois, nos primeiros dias, ele pode estimular a coagulação, podendo causar púrpura vascular. O uso do anticoagulante oral não contraindica o aleitamento.

***5.2.5.2.4. Tempo de Anticoagulação***

O prazo do tratamento anticoagulante deve ser individualizado. De acordo com estudos na população geral, a duração total deve ser entre 3 e 6 meses em pacientes com fatores de risco apenas transitórios. A anticoagulação deve estender-se por pelo menos 6 semanas pós-parto; porém, pacientes com fatores de risco persistentes podem requerer uma duração de anticoagulação mais prolongada.^131,342^

***5.2.5.3. Filtros de Veia Cava Inferior***

Os filtros de veia cava inferior removíveis temporários podem ser utilizados na gestação com indicação semelhante à das não grávidas. Isso significa que são contraindicados em casos de anticoagulação convencional, como: acidente vascular cerebral hemorrágico, sangramento ativo e cirurgia recente; tromboembolismo a despeito de anticoagulação plena; necessidade de interrupção da anticoagulação; ou quando a circulação pulmonar estiver significativamente comprometida. O uso do filtro de veia cava é limitado porque está associado a riscos na inserção e remoção, tais como migração do filtro em mais de 20% , fratura do filtro em 5%, perfuração da veia cava inferior em 5% e mortalidade de 0,12 a 0,3%.^131^

***5.2.5.4. Trombólise***

A trombólise é reservada a pacientes com TEP maciça com hipotensão associada. Estima-se que a mortalidade materna é de 1%, as perdas fetais são de 6%, e a hemorragia materna é de 8%. A HNF intravenosa deve ser iniciada logo após a trombólise, e a HBPM só deve ser iniciada quando houver estabilização do quadro clínico.

### 5.2.6. Profilaxia

Os esquemas propostos para profilaxia (Tabela 36) dos fenômenos tromboembólicos na gestação em diversas situações clínicas são:^131,336,338,342^

**Tabela 36:** – Esquema de profilaxia proposto

História clínica	Conduta na gravidez	Conduta pós-parto
História de tromboembolismo com FR transitório não relacionado a uso de estrogênio e gravidez presente	Observação	Profilaxia anticoagulante com dose profilática ou intermediária de HNF/HBPM por 6 semanas
História de tromboembolismo com FR transitório relacionado ao uso de estrogênio ou gravidez* Passado de tromboembolismo idiopático	Dose profilática ou intermediária de HNF/HBPM	Profilaxia anticoagulante com dose profilática ou intermediária de HNF/HBPM por 6 semanas
Pacientes com trombofilias de alto risco** com passado de tromboembolismo	Dose profilática ou intermediária de HNF/HBPM	Dose profilática ou intermediária de HNF/HBPM por 6 semanas
Pacientes com trombofilia de menor risco, sem tromboembolismo prévio e história familiar da doença	Observação ou dose profilática de HNF/HBPM	Dose profilática de HNF/HBPM por 6 semanas
Pacientes de alto risco, sem tromboembolismo prévio e história familiar positiva	Dose profilática ou intermediária	Dose profilática ou intermediária de HNF/HBPM por 6 semanas
Gestantes com tromboembolismo prévio	Meias elásticas	Meias elásticas
Gestantes com síndrome de hiperestimulação ovariana	Dose profilática de HBPM no primeiro trimestre	

* British Society for Haematology recomenda profilaxia anteparto nessa situação. ** Trombofilias de alto risco: deficiências de antitrombina, anticorpo antifosfolipídio positivo, homozigose para fator V Leiden ou mutação do G20210A (gene da protrombina), dupla heterozigoze (fator V Leiden ou mutação G20210A). FR: fator de risco; HBPM: heparina de baixo peso molecular; HNF: heparina não fracionada.

- HNF profilática: 5.000 unidades de HNF subcutânea, de 12 em 12 h;
- HNF dose intermediária: 10.000 unidades de HNF subcutânea, de 12 em 12 h;
- HNF ajustada: HNF subcutânea, de 12 em 12 h com TTPa ajustado em 1,5 a 2,5 vezes o basal;
- HBPM profilática: dalteparina (5.000 unidades subcutânea de dia), enoxaeparina (40 mg ou 0,5 mg/Kg subcutânea), ou tinzaparina (4.500 unidades subcutânea);
- HBPM dose intermediária: dalteparina (5.000 unidades subcutânea, de 12 em 12 h) ou enoxaeparina (40 mg subcutânea, de 12 em 12 h);
- HBPM dose ajustada: dalteparina (200 U/kg ou 100 U/kg de 12 em 12 h) ou enoxaeparina (1 mg/kg de 12 em 12 h) em doses ajustadas a 0,6 a 1,2 fator antiXa;
- Pós-parto: iniciar com HNF intravenosa ou HBPM subcutânea + varfarina até o INR chegar a 2,0. Posteriormente, manter varfarina por 4 a 6 semanas com INR entre 2,0 e 3,0.

### 5.2.7. Pontos-chaves

- O tromboembolismo é uma importante causa de morbimortalidade na gestação;
- A gestação e outros fatores relacionados podem aumentar o risco da doença;
- O diagnóstico do tromboembolismo deve ser confirmado para justificar o tratamento da doença, que é prolongado, requer medidas profiláticas e tem implicação terapêutica futura;
- Na suspeita de tromboembolismo na gestação, a US venosa deve ser o primeiro exame complementar a ser solicitado;
- A dosagem normal do dímero-D parece ter alto valor preditivo negativo, apesar de não validado na gestação;
- A cintilografia pulmonar V/Q ou a ATCP são os exames de escolha para o diagnóstico de TEP na gestação;
- O tratamento de TVP ou TEP de baixo risco na gestação é baseado no uso de HBPM ou HNF;
- O tratamento deve ser mantido por toda a gestação e por, pelo menos, 6 semanas pós-parto;
- Deve-se usar a profilaxia tromboembólica em gestantes com passado de tromboembolismo. Ela também deve ser considerada na presença de outros fatores de risco;
- A investigação de trombofilia deve ser individualizada;
- A ausência de fatores como trombose venosa profunda, hemoptise, TEP como diagnóstico mais provável e dímero D não superior a 1000ng/ml torna o diagnóstico de TEP improvável.

## 5.3. Tratamento e Prevenção

### 5.3.1. Insuficiência Cardíaca

A IC destaca-se como a principal causa de complicação associada a mortalidade materna em mulheres portadoras de cardiopatias. Sua prevalência é de 0,04% na população geral de gestantes e de 12,5% dentre as portadoras de cardiopatias. É importante salientar que cerca de 60% dos casos de IC ocorrem no pós-parto.^344^ No Brasil, o parto, a despeito de serem assintomáticas, 0,85% das mulheres no puerpério pode vir a apresentar disfunção ventricular.^345^ As situações mais frequentes que devem ser consideradas no diagnóstico da IC no ciclo gravídico-puerperal estão apresentadas na Tabela 37.^346^ A IC associada a CMPP foi discutida no tópico 3.3.7.

**Tabela 37:** – Insuficiência cardíaca durante a gravidez

Causas obstétricas
Pré-eclâmpsia
Cardiomiopatia periparto
Embolia amniótica
Causas não obstétricas
Cardiomiopatia
Embolia pulmonar + disfunção de ventrículo direito
Doença valvar obstrutiva (estenoses mitral e áortica)
Próteses valvares (calcificação ou trombose)
Cardiomiopatias por cardiotoxidade (uso de fármacos)

*Adaptada de: John Antony and Karen Sliwa.^346^*

O diagnóstico da IC durante a gestação é difícil porque as alterações fisiológicas adaptativas da gravidez acarretam sinais/sintomas, os quais, quando exacerbados, devem ser considerados. Sendo assim, a interface na interpretação dos sintomas fisiológicos da gravidez *versus* aqueles da IC, como apresentados na Tabela 38, exige a aplicação de conhecimentos específicos para que seja tomada a decisão mais apropriada, quando de uma eventual intervenção terapêutica.

**Tabela 38:** – Sinais e sintomas da gravidez

Sinais/sintomas	Gravidez normal	Gravidez complicada
Tontura, palpitação	Comum	Síncope exercional
Dispneia	Comum (75%) leve, não progressiva	Progressiva ou CF IV (NYHA)
Ortopnéia	Comum, principalmente no final da gestação	–
Diminuição da tolerância ao exercício	Leve e não progressiva	CF IV (NYHA)
Dor torácica	Comum, não progressiva, em geral musculoesquelética	Angina típica ou dor torácica importante na gestação ou no puerpério
Pulso	Aumentado de volume ou frequência	Diminuído de volume ou ascendente
Edema periférico	Comum, leve	Importante ou progressivo
Bulha apical	Hiperdinâmica, levemente lateralizada	Terceira bulha com desdobramento
Frequência cardíaca	Comum, taquicardia sinusal	FA, TSV persistente, arritmias ventriculares sintomáticas
Veias do pescoço	Levemente distendidas	Progressivamente distendidas com onda “v” dominante

*CF: Classe funcional; FA: fibrilação atrial; NYHA: New York Heart Association; TSV: taquicardia supraventricular.*

**Figura 11:**
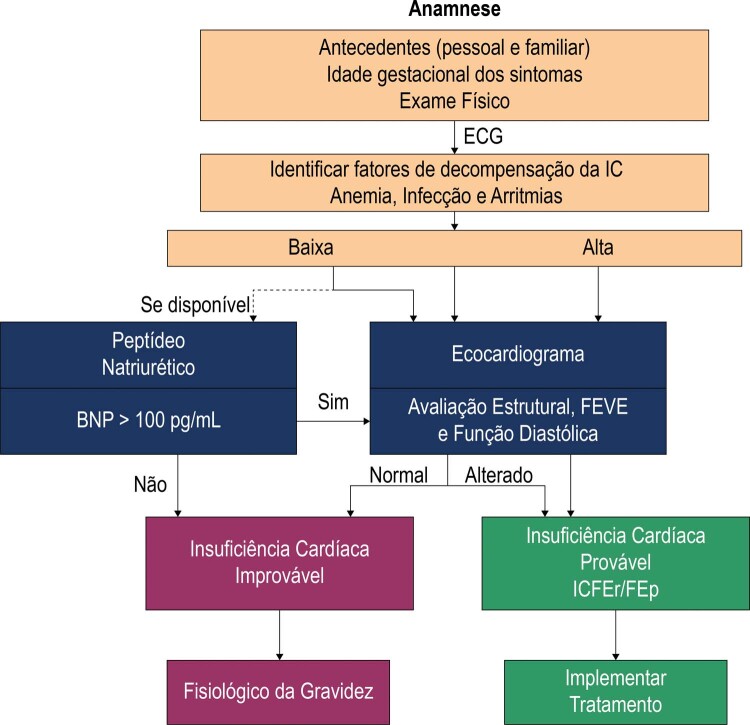
**–** Algoritmo no diagnóstico da insuficiência cardíaca. BNP: peptídeo natriurético; ICFEr: IC com fração de ejeção reduzida; ICFEp: IC com fração de ejeção preservada. Adaptada de Rohde et al., 2018.^345^

Da avaliação inicial ao seguimento clínico, o médico deve voltar sua atenção para os antecedentes pessoais e familiares de cardiopatia, a idade gestacional em que houve a progressão da CF I/II para III/IV e a identificação de fatores como arritmias cardíacas, anemia e infecções (Figura 11).

A gravidez em mulheres com FEVE < 40% em CF III/IV (NYHA), fatores considerados preditores de mortalidade, geralmente é mal tolerada^347^e deve ser desaconselhada. Em casos em que a FEVE seja < 20%, a gravidez deve ser contraindicada, e a sua interrupção, quando em curso do primeiro trimestre, deve ser considerada.

A rotina propedêutica para as gestantes com suspeita de IC deve incluir exames subsidiários básicos, a saber: laboratoriais (hemograma, eletrólitos séricos, função renal, glicemia de jejum, hemoglobina glicosilada, perfil lipídico, função tireoidiana e função hepática); ECG de 12 derivações para identificação de arritmias, sobrecarga de câmaras cardíacas, distúrbios de condução; radiografia de tórax para detecção da congestão pulmonar; e ecodopplercardiograma transtorácico bidimensional com análise dos fluxos pelo método Doppler, que é o teste diagnóstico por imagem preferencial para avaliação inicial, não só por sua ampla disponibilidade, como também por prescindir do uso de radiação ionizante. O ECO identifica alterações estruturais cardíacas, incluindo anormalidade do miocárdio, das valvas e do pericárdio, além de avaliar aspectos hemodinâmicos.^345^

Estudos têm confirmado a validade do BNP como marcador de IC também na gestação.^348^ Os valores acima de 100 pg/ml contribuem para dar sustentabilidade clínica no diagnóstico de IC e facilitar a implementação de medidas terapêuticas apropriadas. A incorporação de níveis seriais de BNP na prática clínica pode ser útil, especificamente no julgamento de eventos cardíacos adversos durante a gravidez.

A avaliação do prognóstico da IC durante a gravidez é semelhante à convencional; contudo, os exames invasivos ecotransesofágico, RMC, cintilografia de perfusão miocárdica, PETscan**,** angiotomografia de coronárias e teste cardiopulmonar devem ser postergados para após a gravidez.

A prevenção da IC durante a gestação exige uma orientação multidisciplinar em conjunto com o obstetra e deve obedecer às seguintes recomendações: (1) visita médica quinzenal ou semanal; (2) controle do peso corporal; (3) insistência em evitar atividades que exijam grandes esforços; (4) restrição hídrica e salina moderada; (5) eventual afastamento das atividades profissionais que exijam grandes esforços; (6) manutenção dos fármacos não teratogênicos; e (7) hospitalização em pacientes que mantêm CF III (NYHA) com medicação otimizada.^349^

A avaliação obstétrica concomitante ao atendimento cardiológico é importante no estabelecimento da idade gestacional. Assim, a condição da vitalidade e do crescimento fetais e a situação do fluxo placentário são fatores que apoiam a terapêutica e refletem a condição hemodinâmica materna.

O tratamento farmacológico da IC com fração de ejeção reduzida (ICFEr) difere daquele realizado na população de cardiopatas em geral quanto a classe de fármacos utilizada, dose diária e metas terapêuticas,^52^ uma vez que os fármacos teratogênicos devem ser substituídos na pré-concepção.

Os betabloqueadores, principalmente os beta-1-cardiosseletivos (metoprolol, bisoprolol e carvedilol), são considerados fármacos de primeira linha, porque determinam benefícios na mortalidade por IC e na MCS, além de melhorarem os sintomas e reduzirem as taxas de re-hospitalizações por IC.^345^ Por tais razões, o uso desses betabloqueadores deve ser mantido durante a gestação em casos de ICFEr.

A literatura carece de dados sobre a dose-alvo para o alcance das metas terapêuticas durante a gestação, que não devem ser as mesmas consideradas na população de cardiopatas em geral. Isso porque a frequência cardíaca reduzida e a queda da pressão arterial consequente a altas doses, que habitualmente são fatores usados na população de pacientes com IC, podem comprometer a circulação uteroplacentária.

De modo geral, é prudente que as doses dos fármacos utilizados durante a gravidez sejam fracionadas, inicialmente baixas e aumentadas gradual e cautelosamente, buscando a maior dose tolerada pela mãe e pelo feto. Assim, recomenda-se uma dose inicial do bisoprolol de 1,25 mg/dia, do carvedilol de 3,125 mg 2 vezes/dia e do succinato de metoprolol de 12,5 mg 2 vezes/dia, de acordo com as recomendações na população de pacientes com IC.^345^

A vitalidade (perfil biofísico e cardiotocografia) e a maturidade fetal devem ser avaliadas com maior frequência quando comparadas às da população de gestantes saudáveis. No período neonatal, a supervisão deve ser durante 24 a 48 horas após o nascimento, considerando os sintomas e sinais mais frequentes, tais como depressão respiratória, bradicardia, hiperbilirrubinemia e hipoglicemia. Por esse motivo, quando próximo do parto, uma medida prudente é a redução progressiva do betabloqueador, buscando a mais baixa dose que tenha eficácia materna.^344^

A ocorrência de congestão pulmonar demanda o uso de diuréticos de alça, preferencialmente furosemida e tiazídicos, na tentativa de otimizar a pré-carga. Caso não haja congestão, eles devem ser evitados, sob risco de causarem redução do fluxo uteroplacentário.^346^ Atenção para os efeitos deletérios do uso permanente de diurético, tais como: piora do fluxo placentário, aumento do ácido úrico (marcador precoce da pré-eclâmpsia), aparecimento de distúrbios eletrolíticos materno-fetais e RCIU.

A hidralazina pode ser usada no tratamento dos sintomas de IC, com ou sem nitratos, como alternativa de tratamento caso a PAS seja > 110 mmHg, principalmente se houver hipertensão arterial associada, disfunção grave de ventrículo esquerdo e/ou evidência de congestão.^52,345^ Contudo, durante a gravidez, a associação hidralazina/nitratos tem sido relacionada a baixa tolerância materna devido à habitual hipotensão arterial.

A digoxina pode ser usada quando persistir a sobrecarga de volume, apesar da terapia com vasodilatadores e diuréticos. O uso do digital, quando necessário em pacientes com ICFEr, tem um papel importante no controle da frequência cardíaca materna, principalmente na presença de FA.^345^

A anticoagulação na IC durante a gravidez é controversa. A HBPM ou HNF podem ser consideradas em pacientes nas situações mais frequentes, tais como CMD com FEVE < 35%, hospitalização prolongada e antecedentes de eventos tromboembólicos. É oportuno considerar que o puerpério acrescenta um maior risco de tromboembolismo, daí a anticoagulação ser indicada nessa fase do ciclo gravídico-puerperal.

Quanto às arritmias na IC, a mais comum é a FA, que pode ser tratada com betabloqueadores; se necessário, acrescenta-se a digoxina para controlar a frequência cardíaca. Quanto às arritmias ventriculares frequentes ou à taquiarritmia ventricular sustentada, o tratamento inclui o uso de amiodarona e, quando de maior risco, estão indicados os CDI.

Quando ocorrer instabilidade hemodinâmica e choque cardiogênico, a paciente deverá ser transferida inicialmente para UTI e, se possível, com SMC^346^e em sequência o parto cesárea de urgência; entretanto, em caso eletivo, a via de parto fica por indicação obstétrica, levando em consideração a paridade da mãe, as comorbidades existentes e a gravidade da lesão cardíaca.

No puerpério, deve-se evitar sobrecarga de volume resultante da infusão de fluidos no intraparto e pós-parto. O uso da ocitocina em baixas doses deve ser considerado, a despeito das suas propriedades vasoativas, e a ergonovina deve ser evitada devido ao seu efeito vasoconstritor periférico.

### 5.3.2. Pontos-chaves

- Os sintomas e sinais fisiológicos da gravidez podem atrasar o diagnóstico de IC;
- O BNP (≤ 100 pg/ml) é um marcador de IC também válido na gravidez;
- O BNP seriado na gestação auxilia no diagnóstico de IC e na terapêutica;
- Os betabloqueadores são considerados os fármacos de primeira linha e devem ser mantidos durante a gestação em casos de ICFEr;
- No planejamento familiar, a gravidez deve ser desaconselhada para mulheres com IC crônica que apresentam FEVE < 40% e contraindicada naquelas em CF III/IV com FEVE < 20%.

## 5.4. Tratamento e Prevenção

### 5.4.1. Endocardite Infecciosa

EI é rara durante a gravidez; ocorre em 0,006% da população geral; porém, em paciente com doença valvar ou cardiopatia congênita, esse percentual chega a 1,2%.^270,350^ As pacientes portadoras de prótese valvar e cardiopatias complexas cianogênicas, bem como as usuárias de drogas ilícitas, constituem o grupo de maior risco.

Trata-se de uma doença grave com mortalidade materna próxima de 33%, consequente IC e fenômeno tromboembólico.^350,351^ Durante a gravidez, deve ser dada atenção especial a febre sem causa aparente e à semiologia do coração, uma vez que o aparecimento de sopros cardíacos inocentes ou funcionais é muito frequente durante a gestação normal.

A abordagem da EI demanda uma assistência multidisciplinar em centro terciário de cardiologia, apoiada nas decisões do “heart team” que esteja capacitado a oferecer os recursos disponíveis para diagnóstico, tratamento e seguimento de acordo com as recomendações convencionais.^350^

A profilaxia da EI durante a gravidez segue as mesmas recomendações utilizadas para pacientes não grávidas.^350,351^ Promover saúde bucal, orientar sobre a higienização e consulta odontológica periódica na vigilância do aparecimento da gengivite gravídica que favorece a doença periodontal é uma orientação básica na prevenção da EI, uma vez que a porta de entrada para os agentes etiológicos mais frequentes é a cavidade oral.

A antibioticoprofilaxia para tratamento odontológico é controversa; contudo, quando indicada, utiliza-se amoxacilina 2 g por via oral ou clindamicina 600 mg por via oral nos pacientes alérgicos à penicilina, 1 h antes da intervenção.

A antibioticoprofilaxia da EI na ocasião do parto vaginal ou cesárea é também controversa,^350^ e a falta de evidências sobre a prevenção da doença pelo uso de antibióticos na ocasião do parto fragiliza tal indicação. Entretanto, deve-se considerar que a ocorrência de EI no pós-parto é grave e as complicações peculiares a esse período, que elevam a bacteremia (extração manual da placenta, curetagem, retenção placentária),^352^ não são previsíveis. Vale lembrar que a infecção puerperal no Brasil é uma das principais causas obstétricas de morte materna. Por isso, a decisão de se realizar a antibioticoprofilaxia para a EI na ocasião do parto deve ficar a critério da equipe que atende a parturiente, de acordo com a individualização de cada caso.

Embora, ainda controverso, as situações clínicas de alto risco para a EI que podem exigir a antibioticoprofilaxia de rotina estão apresentadas na Tabela 39,^350^ e as recomendações quanto ao modo de aplicação estão na Tabela 40.

**Tabela 39:** – Cardiopatias de alto risco para endocardite infecciosa350

Próteses valvares	
Próteses implantadas transcateter	
Material protético usado para plastia valvar, como anéis para anuloplastia e corda artificial	
Endocardite infecciosa prévia	

**Cardiopatia congênita**	**Cianogênica não operada**

	Cardiopatia complexa com lesão residual (*shunts*, regurgitação valvar Não local do enxerto, tubos valvulados)

**Tabela 40:** – Antibióticos e doses utilizados uma hora antes do parto

Antibiótico	Doses
Ampicilina	2,0 g IV ou IM
Associada com gentamicina	1,5 mg/kg VO, IV ou IM
Pacientes alérgicos a penicilina/ampicilina/amoxacilina	
Vancomicina	1,0 g IV em 1 h
Associada com gentamicina	1,5 mg/kg IV ou IM

*IV: via intravenosa; IM: via intramuscular; VO: via oral.*

O diagnóstico clínico da EI resume-se a história de febre, calafrio, queda do estado geral, fenômeno embólico, periférico ou central, fenômeno vascular ou imunológico, glomerulonefrite e aparecimento de novo sopro. Quanto aos exames complementares, o doppler-ECO transtorácico deve ser sempre realizado quando houver a suspeita clínica; o transesofágico é indicado quando o transtorácico for negativo para EI e nos casos de prótese valvar. As hemoculturas devem ser coletadas antes da introdução dos antibióticos, mínimo de três amostras, em um intervalo de 30 min, por meio de técnicas estéreis em punção venosa periférica, independente do pico febril; se possível, repetir em 12 horas. O tratamento deve ser iniciado após a coleta das hemoculturas e precisa ser pautado na epidemiologia, na história clínica e no resultado das hemoculturas e do antibiograma, de acordo com as diretrizes convencionais.^350,351^

Vale lembrar que o agente etiológico mais comum da EI no Brasil é o *Streptococcus viridans* da cavidade oral. A escolha do antibiótico, pela via de administração intravenosa, e o tempo de antibioticoterapia são os mesmos da paciente não grávida, considerando-se os possíveis efeitos tóxicos dos antibióticos sobre o feto.^52,350,351,353^

Nesse sentido, existem três grupos de antibióticos classificados quanto aos riscos para a gestação: (1) os mais seguros, que compreendem ampicilina, penicilina, amoxacilina, oxacilina, eritromicina, daptomicina e cefalosporinas; (2) aqueles que apresentam risco intermediário e que devem ser monitorados, tais como vancomicina, imipenem, rifampicina e teicoplamina; e (3) os contraindicados, que são aminoglicosídeos, quinolonas e tetraciclina.^354^

O tratamento cirúrgico nos casos de EI segue as indicações convencionais, tais como falha do tratamento etiológico, IC refratária, fenômeno embólico de repetição, complicações periprótese, abscesso ou deiscência de prótese. Recomenda-se que o parto seja antes da cirurgia cardíaca nos casos de viabilidade fetal.^351,353^

### 5.4.2. Doença Reumática

A febre reumática (FR) é uma doença com resposta autoimune que ocorre após a infecção da orofaringe pelo *Streptococcus* beta-hemolítico do grupo A, de Lancefield.^355^ O primeiro surto reumático acomete crianças na primeira infância e contribui para um contigente importante de mulheres portadoras de doença valvar na idade reprodutiva e, portanto, na gravidez.

A FR aguda é rara durante a gravidez, mas o seu diagnóstico deve ser considerado em gestantes adolescentes sem profilaxia prévia ou que apresentam quadro de IC grave desproporcional a grau de acometimento valvar.

O diagnóstico é orientado utilizando-se critérios clínicos de Jones e exames complementares.^355^Tanto os critérios maiores (cardite, coreia de Sydenhan, artrite migratória, eritema marginatum, nódulos subcutâneos) como os menores (febre, artralgia) são válidos durante a gestação; contudo, reagentes de fase aguda, como a alfaglicoproteína ácida, a proteína C reativa e a eletroforese das proteínas, podem sofrer influência da gravidez. Por isso, o diagnóstico é fortemente baseado na clínica e na história da paciente.

Nesse sentido, vale considerar que a coreia de Sydenhan é uma frequente causa de coreia em pacientes com manifestação prévia e, devem ter seu diagnóstico diferencial com a coreia gravídica, que pode estar associada a outras morbidades que não a FR. Ambas as manifestações de coreia são ligadas a alto risco obstétrico, como perdas fetais, e requerem tratamento diferenciado.^355^

O mesmo vale dizer sobre a distinção da IC consequente à cardite reumática e da valvopatia crônica: ambas elevam o risco de morte materna e têm tratamentos muito diferentes.^356^

O tratamento do surto reumático, que é raro durante a gravidez, deve ser igual ao da população em geral. A hospitalização é indicada em todos os casos com suspeita de cardite, artrite incapacitante ou coreia grave, e o repouso domiciliar deve ser por um período mínimo de quatro semanas e, eventualmente, até o parto.^357^

A profilaxia secundária da FR deve ser mantida durante a gestação de acordo com as seguintes recomendações: penicilina G benzatina 1.200.000 UI por via intramuscular a cada 21 dias ou fenoximetilpenicilina 250 mg por via oral, 2 vezes por dia. Em pacientes alérgicos a penicilina, recomenda-se eritromicina 250 mg via oral, 2 vezes por dia, ou clindamicina 600 mg/dia.^357^ O uso de sulfadiazina é contraindicado na gravidez.

A duração da profilaxia independe da ocorrência da gravidez e relaciona-se com os seguintes fatores: FR sem cardite prévia (até 21 anos ou 5 anos após o último surto, valendo o que cobrir o maior período); FR com cardite prévia, valvopatia residual leve ou resolução da lesão valvar (até 25 anos ou 10 anos após o último surto, valendo o que cobrir o maior período); lesão valvar residual moderada a grave (até os 40 anos ou por toda a vida); após cirurgia valvar (até 40 anos ou por toda a vida). Pacientes com risco de faringite de repetição, como aquelas que trabalham em creches e casas de saúde, devem fazer a profilaxia secundária por toda a vida.^353,358^

### 5.4.3. Pontos-chaves e Recomendações

- A antibioticoprofilaxia para a EI na ocasião do parto deve ser realizada em pacientes de alto risco para a EI;
- A profilaxia da FR deve ser mantida durante a gravidez.

## 5.5. Cirurgia Cardiovascular na Gravidez

A experiência mundial em cirurgia cardíaca durante a gravidez apresenta resultados controversos. Predominam o caráter retrospectivo e a heterogeneidade dos procedimentos, associada às dificuldades na padronização das técnicas cirúrgicas, o que dificulta a análise judiciosa das variáveis de prognóstico e seus reflexos na conduta durante a gravidez.^359,360^

Admite-se que o risco de morte materna pela cirurgia cardíaca não é modificado pela gravidez.^359^ No entanto, quando a cirurgia tem caráter de emergência, o risco de mortalidade materna aumenta.^361^ Nesse aspecto, verifica-se que o percentual de mortalidade materna de 7,5 a 13,3% foi relativamente elevado quando comparado ao de cirurgia cardíaca na população da mesma faixa etária, e o caráter da cirurgia, ser de emergência, foi a variável preditiva de morte.^359,361,362,363^

Outro aspecto de importância na indicação da cirurgia cardíaca é a idade gestacional. Presume-se que quanto mais precoce é o aparecimento de complicações em portadoras de lesões graves, maior é a tendência em se indicar a cirurgia precoce, porque a deterioração hemodinâmica é um fato com o progredir da gravidez. Essa linha de pensamento justifica que o melhor período para a programação da cirurgia cardíaca é durante o segundo trimestre da gestação, vez que o feto ainda é inviável e as modificações fisiológicas e mecânicas da gravidez ainda não são tão importantes, além de favorecer um período razoável para a recuperação pós-operatória materna para o parto.^362,363^

A cirurgia durante a gravidez exige cuidados específicos, com destaque para a seleção dos anestésicos, o monitoramento materno-fetal contínuo e o controle adequado da anticoagulação. A equipe obstétrica deve iniciar a monitorização fetal, por meio da cardiotocografia, para controle da dinâmica uterina e do seu batimento cardíaco. A indução anestésica deve ser cuidadosa para evitar períodos de hipóxia e hipotensão, e a escolha dos fármacos, sem efeitos teratogênicos.^52^

A técnica da cirurgia cardiovascular durante a gravidez não difere das habituais; contudo, a redução do tempo cirúrgico, principalmente o de CEC, além dos cuidados específicos apresentados na Tabela 41, melhoram os resultados finais.

**Tabela 41:** – Cuidados na cirurgia cardíaca com circulação extracorpórea durante a gravidez

Controle da hemodiluição, que não deve ser inferior ao nível de 25% de hematócrito
Utilização de fluxo superior a 30 a 40% ao fluxo habitual, mantendo pressão arterial média acima de 60 mmHg
Utilização de hipotermia leve ou normotermia, de modo a evitar arritmias do feto, tanto no resfriamento quanto no aquecimento, e diminuir as contrações uterinas
Utilização de glicose acrescida no perfusato, a fim de evitar bradicardia fetal e melhorar as condições energéticas do feto
Controle adequado do equilíbrio acidobásico, evitando a acidose

Habitualmente, ocorre uma queda da frequência cardíaca fetal Não início da instalação da CEC, retornando à normalidade Não seu término.^359^ Esse fato se deve principalmente a mudança para o fluxo contínuo, efeito embólico de microbolhas, hipotensão inicial, hemodiluição, empilhamento de hemácias e alterações na resistência vascular periférica. Tal “disfunção aguda” da placenta consequente ao comprometimento do fluxo uteroplacentário é a razão da alta incidência de perdas fetais, prematuridade, óbito neonatal e malformações.^361,364^

Tem sido recomendado que o parto seja indicado antes da cirurgia cardíaca, se o feto for viável. Vale salientar que o uso de corticoide para maturação pulmonar do feto é muito arriscado para gestantes com quadro hemodinâmico instável e grave, frequente nessa situação. Isso porque o uso do corticoide nas doses preconizadas (duas doses de betametasona, 12 mg intramuscular, 12 h antes do parto) pode levar a agravamento da IC, choque cardiogênico e morte materna.

A prevenção do trabalho de parto prematuro com a progesterona natural (óvulos de 50 mg, a cada 12 h no período do intra e pós-operatório) tem preferência de uso, uma vez que a indometacina pode ocasionar o fechamento do canal arterial, especialmente após 26^a^ semana de gestação.^365^

A cirurgia cardíaca, ainda que constitua alto risco para a gravidez, deve ser indicada nas condições clínicas sem outras opções terapêutica farmacológica ou percutânea para a sobrevida materna. O procedimento cirúrgico em situação de emergência tem correlação significativa com complicação materna no pós-operatório; por isso, o momento da indicação à cirurgia tem implicação direta nos resultados materno-fetais.^361,366^

### 5.5.1. Pontos-chaves

- A cirurgia cardíaca durante a gravidez deve ser indicada nas condições clínicas sem outras opções terapêuticas para a sobrevida materna;
- A cirurgia de emergência tem correlação significativa com a complicação materna no pós-operatório;
- Cirurgia cardíaca durante a gravidez exige cuidados diferenciados e protocolo hospitalar.

## 5.6. Intervenção Cardíaca Percutânea

### 5.6.1. Princípios Gerais

O uso das intervenções percutâneas durante a gestação tem aumentado gradualmente, impulsionado pela sua maior disponibilidade e pelos riscos impostos pela cirurgia com CEC. Em geral, essas intervenções durante a gestação são consideradas em cardiopatias graves sintomáticas que não podem ser adiadas pelo risco de vida materno.^52^

A intervenção percutânea quando possível deve ser realizada no início do segundo trimestre porque considera: (1) organogênese quase completa; (2) função tireoidiana fetal não ativa; (3) volume uterino de moderado aumento (distância maior do feto ao tórax materno); (4) facilidade da utilização de dispositivos de barreira para proteção.^52^

Um método alternativo de proteção ao feto é a utilização do ECO (transtorácico, esofágico ou tridimensional) em substituição à fluoroscopia. Ele possibilita o posicionamento de cateteres e a medida dos diâmetros de orifícios valvares e da posição de saída das coronárias da aorta, além de servir como guia para procedimentos de valvuloplastia por cateter balão, de inserção de próteses valvares incluindo “valve in valve” e de auxílio na liberação de “stents*”* coronarianos.

A fluoroscopia deve respeitar critérios que incluem o emprego de baixas doses de radiação, o uso de blindagem abdominal e o afastamento da radiação direta na região abdominal. Os procedimentos devem ser os mais curtos possíveis, porque o risco de radiação para o feto deve ser sempre considerado. No entanto, essa preocupação não deve impedir o emprego de procedimentos diagnósticos essenciais, utilizando a melhor modalidade disponível para a situação clínica.^52^

### 5.6.2. Intervenções Percutâneas Valvares

***5.6.2.1. Valvoplastia por Cateter-Balão na Estenose Mitral***

A VCB na estenose mitral deve ser realizada, de preferência, no segundo trimestre da gestação e indicada em portadoras de estenose mitral importante, em CF III/IV (NYHA), com resposta insatisfatória ao tratamento clínico convencional.^52^ Os resultados da VCB, obedecidas as suas indicações, demonstraram-se superiores aos da cirurgia convencional, com menor mortalidade e melhora clínica em cerca de 80% dos casos.^367^

Os critérios de indicação da VCB mitral incluem:

- Ausência de: (1) insuficiência mitral grave; (2) outra lesão valvar ou coronariana concomitante com indicação de correção; (3) trombo no átrio esquerdo comprovado pelo ECO transesofágico;
- Condição anatômica da valva mitral compatível ou seja: (1) certa flexibilidade; (2) calcificação não excessiva; (3) fusão comissural; (4) porção subvalvar passível de abordagem;
- Valor do escore ecocardiográfico de Wilkins igual ou inferior a 8, o que permite melhor resultado imediato e tardio.^368^

É controversa a ampliação dos índices do escore de Wilkins para 10 em função da gravidez, porque o potencial de complicações como insuficiência mitral aguda pode ser plenamente fatal. Em situações muito especiais, a VCB mitral com índice acima de 8 requer discussão prévia com *heart team* e a disposição de recursos para a eventual necessidade de cirurgia de emergência.^369^

***5.6.2.2. Estenose Aórtica***

Pacientes que apresentam estenose aórtica grave com manifestação de IC, angina limitante e síncope durante a gravidez têm indicação de intervenção valvar, e a valvoplastia aórtica por cateter balão (VPAo) pode ser realizada por um operador experiente.^370^ Em adolescentes, a VPAo tem bons resultados imediatos e tardios; porém, em pacientes de maior faixa etária, os resultados são piores. Assim, a VPAo pode servir como “ponte”^371^ para melhora temporária da condição clínica, tornando possível alcançar a idade gestacional para o parto seguro em condições hemodinâmicas favoráveis. Vale lembrar que o procedimento deve ser realizado com a disponibilidade da cirurgia convencional de resgate, em casos de emergência. Além disso, é essencial que, após a gestação, essas pacientes sejam acompanhadas com exames clínicos e ecocardiográficos periódicos para determinar a eventual necessidade de correção definitiva da valvopatia.

***5.6.2.3. Estenose Congênita da Valva Pulmonar***

A estenose da valva pulmonar (EP) grave, sintomática, com manifestação de IC, arritmias ou síncope é pouco frequente durante a gravidez. Contudo, nessa situação, a VCB tem sido indicada com sucesso imediato e tardio.^372^

***5.6.2.4. Implante Percutâneo de Próteses Valvares***

Nos últimos anos, temos assistido ao desenvolvimento do implante valvar transaórtico (TAVI). Ele tem o grande mérito de evitar a cirurgia cardíaca com CEC, mas requer o uso intensivo de radiação ionizante em dois momentos essências: (1) tomografia da valva aórtica, para o estudo preliminar das estruturas envolvidas (anel aórtico, diâmetro da prótese, altura da emergência das coronárias e sistema arterial torácico e periférico); (2) radioscopia durante o procedimento, para auxiliar no posicionamento dos cateteres e na visualização da expansão da prótese. Assim, o TAVI convencional não é aprovado durante a gestação, em função da alta carga radioativa sobre o feto.

Contudo, a US arterial na avaliação do sistema arterial (ilíacas, aorta, altura das coronárias) em conjunto com o ECO tridimensional (avaliação do anel valvar) trouxe sucesso ao primeiro caso relatado de TAVI durante a gestação,^373^ no qual se utilizaram curtos períodos de radioscopia para a fixação da prótese. O fato de as gestantes serem mais jovens, com leito vascular arterial saudável, facilita a navegação dos cateteres; mas por outro lado, o grau de calcificação da valva pode não ser adequado para permitir a fixação da prótese, nesta faixa etária.

***5.6.2.5. Procedimento de “Valve in Valve*” *na Disfunção de Prótese Biológica***

A disfunção de PB em mulher jovem é muito frequente e, muitas vezes, requer troca valvar durante a gestação. Nesse cenário, o procedimento do tipo “valve in valve*”* é promissor para evitar a cirurgia com CEC. As próteses são introduzidas por meio de cateteres, utilizando as seguintes vias: (1) artéria femural ou outros acessos arteriais para a aorta; (2) veia femural seguida da punção transseptal e acesso atrial esquerdo; (3) incisão apical do ventrículo esquerdo (transapical). O relato do caso^374^ durante a gravidez descreve o implante transapical de duas próteses, mitral e aórtica, com o auxílio de visão ecocardiográfica transesofágica e uso restrito da fluoroscopia, o que permitiu o alcance do parto vaginal com bom resultado materno-fetal.

***5.6.2.6. Angioplastia Coronariana***

A intervenção coronária percutânea primária é o tratamento selecionado para a síndrome coronariana aguda durante a gestação, sendo a trombólise menos utilizada. A angioplastia coronária com “stents*”* convencionais tem sido considerada segura nos casos de doença arterial obstrutiva por doença aterosclerótica.

Enquanto a segurança dos “stents*”* farmacológicos ainda é desconhecida, a obrigatoriedade da dupla agregação plaquetária por tempo prolongado nesse tipo de *stent* constitui uma considerável restrição ao seu uso durante a gestação, decorrente dos riscos hemorrágicos. Além disso, o clopidogrel deve ser interrompido sete dias antes do parto, o que acrescenta um risco de trombose do “stent”.

Na dissecção espontânea de coronária, a indicação da angioplastia deve considerar as dificuldades técnicas e a fragilidade vascular peculiar a essa situação, o que eleva o risco de extensão do dano coronariano, além de o sucesso ser considerado subótimo.^375^Por isso, a maioria dos casos de dissecção de coronária se beneficia com o tratamento conservador.^376,377^ Em situações em que a angioplastia coronária seja indicada, a opção do uso dos “stents” farmacológicos de nova geração que exigem a dupla agregação por menor tempo (3 meses) pode ser uma opção mais segura.

Contudo, o dilema da decisão é a consideração de ambos os riscos obstétricos (hemorragia materna) e cardíacos (trombose dos “stents”) que deve ser julgada “caso a caso” pela equipe multidisciplinar, porque até o momento não existe consenso sobre estas circunstâncias.

### 5.6.3. Pontos-chaves

- A intervenção percutânea durante a gestação deve ser indicada em casos de complicações refratárias ao tratamento clínico convencional ou nas condições de risco iminente de vida materna.
- A intervenção percutânea deve ser realizada sempre após a discussão com o Heart Team em Serviços terciários de cardiologia.

## 5.7. Emergências Cardiológicas

### 5.7.1. Insuficiência Cardíaca Aguda

**Figura 12:**
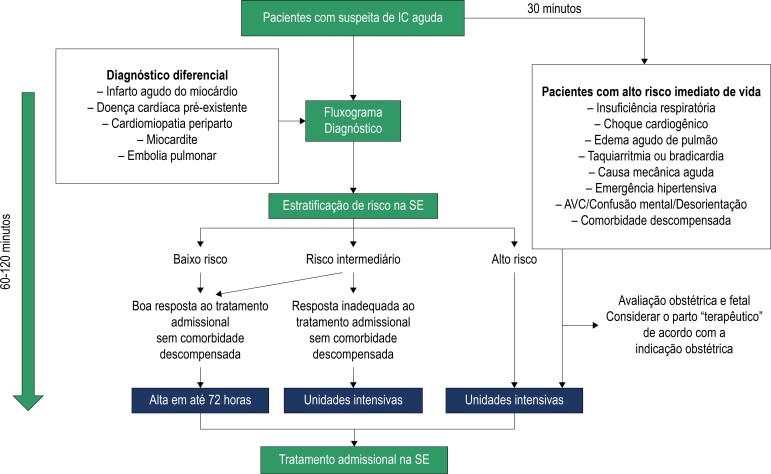
**–** Algoritmo do diagnóstico na suspeita clínica de insuficiência cardíaca aguda. SE: sala de emergência. Adaptada de Rohde et al., 2018.^345^

A conduta perante a insuficiência cardíaca aguda (ICA) durante a gestação procura a melhora dos sintomas e a prevenção da morte materna).^378^ A orientação segue as recomendações de atendimento do paciente em IC na sala de emergência^345^ (Figura 12), mas devem ser considerados os riscos quanto ao uso de medicações sobre a mãe, o feto, o trabalho de parto, a aleitamento e os ajustes necessários de acordo com a idade gestacional.

Vale ressaltar que além dos sintomas de ICA, a identificação da congestão sistêmica e/ou pulmonar, e de baixo débito, apoiados nos exames subsidiários definem a causa determinante na maioria dos casos.^345,378,379^

Exames laboratoriais devem fazer parte da investigação da ICA^348,380^ e incluem: (1) dosagem de eletrólitos; (2) peptídeo natriurético (BNP); (3) provas de função renal; (4) marcadores de necrose miocárdica; (5) perfil tireoidiano; (6) hemograma completo; (7) marcadores de infecção.

A interação com a equipe obstétrica é obrigatória para a determinação da idade gestacional e dos parâmetros de vitalidade e viabilidade fetal. A eventual indicação de parto terapêutico e a via de parto devem fazer parte do algoritmo no atendimento da ICA na gravidez.

A dispneia aguda durante a gravidez deve ter como diagnóstico diferencial: IAM, CMPP, TEP e miocardite.^222^A orientação para o diagnóstico diferencial pode ser resumida como segue:

- IAM dispneia e dor anginosa; idade acima de 35 anos; antecedentes de tabagismo e uso de contracepção com componentes estrogênicos; elevação dos níveis séricos de troponina; ECO com alteração de motilidade segmentar. O diagnóstico definitivo se faz pela cineangiocoronariografia;
- Cardiopatia preexistente: a dispneia é mais frequente no segundo e terceiro trimestres. Os níveis séricos de BNP podem estar elevados e o ECO mostra lesão cardíaca estrutural. No Brasil, é comum o edema agudo dos pulmões como primeira manifestação de estenose mitral durante o segundo trimestre da gestação;
- CMPP: dispneia nas últimas semanas de gestação, próxima ao parto, ou mais frequentemente após o parto, com importante elevação dos níveis de BNP e disfunção sistólica nova dos ventrículos esquerdo e direito;^222^
- TEP: a dispneia é associada a dor pleurítica. Os níveis de troponina e BNP estão elevados, e a disfunção do ventrículo direito e a HP são sinais de maior de gravidade do evento. Vale ressaltar que a sensibilidade e o valor preditivo negativo do dímero-D na suspeita de edema agudo dos pulmões são limitados;^381^
- Miocardite: dispneia está associada a sintomas inespecíficos relacionados com infecção viral. A troponina pode estar elevada (processo inflamatório miocárdico aumenta sua liberação celular), e o ECO pode demonstrar acinesia segmentar ou hipocinesia difusa. A RMC com identificação de edema miocárdico ou fibrose mesocárdica reforçam o diagnóstico.^382,383^

Durante a avaliação clínica, é primordial a determinação do perfil hemodinâmico. Em pacientes classificadas como perfil B (quentes e congestas), o ajuste da volemia com diuréticos e vasodilatadores, na ausência de hipotensão e choque, deve ser considerado com parcimônia, haja vista a contraindicação formal ao uso de IECA e BRA, dando-se preferência para uso de nitratos e hidralazina, quando possível, em terapêutica combinada.

Os diuréticos de alça são seguros, e o mais utilizado é a furosemida, na dose de 20 a 40 mg iniciais, com possibilidade de otimização a depender do uso crônico prévio, da resposta diurética, da melhora da dispneia e da hipoxemia.^384^Os riscos sobre o feto são consequentes à redução do fluxo placentário frente ao ajuste da volemia além do necessário.

Em pacientes mais graves ou em edema agudo de pulmão, sem hipotensão ou choque, opta-se pelo uso de nitroglicerina ou nitroprussiato de sódio em infusão contínua, guiados preferencialmente por monitoramento arterial invasivo. As doses e a velocidade de infusão estão descritas na Tabela 42. O monitoramento fetal contínuo também deve ser realizado, visto que a redução brusca da pressão arterial materna pode comprometer a vitalidade fetal.

**Tabela 42:** – Recomendação de vasodilatadores por via intravenosa na insuficiência cardíaca aguda

Vasodilatador	Posologia	Ajuste
Nitroglicerina	Início: 10 a 20 mcg/min Máximo: 200 mcg/min	A cada 15 min Aumento: 10 a 20 mcg/min
Nitroprussiato de sódio	Início: 0,3 mcg/kg/min Máximo: 5 mcg/kg/min	A cada 15 min Aumento: 0,3 a 0,5 mcg/kg/min

Suporte ventilatório não invasivo (VNI) com pressão positiva está indicado para todas a pacientes com saturação arterial periférica < 90%, com esforço ou desconforto respiratórios, que não apresentaram melhora com uso de oxigenoterapia.^369^ Também está indicado para pacientes com edema agudo de pulmão, uma vez que são conhecidos os benefícios em não gestantes na redução da necessidade de suporte de ventilação mecânica invasivo.^348^

Em pacientes com hipotensão sintomática, sinais de baixo débito com disfunção orgânica ou choque cardiogênico, a associação de vasoconstritores, deve seguir indicações à semelhança das pacientes não gestantes. A dobutamina é o agente inotrópico mais usado porque promove aumento do débito cardíaco dose-dependente, embora seu efeito arritmogênico seja limitante e apresente menor eficácia em casos de uso crônico de betabloqueador. A milrinona, além de aumentar o débito cardíaco, é capaz de reduzir a resistência pulmonar e periférica, e, portanto, é indicada em pacientes portadores de CC e hipertensão pulmonar.^344^ A levosimendana apresenta efeito inotrópico positivo, contudo, por sua ação de vasodilatação periférica, seu uso deve ser mais criterioso em gestantes. A Tabela 43 apresenta os fármacos e as doses recomendadas para o tratamento de ICA durante a gravidez. Em pacientes portadoras de ICA por CMPP, há preferência pela levosimendana, haja vista os efeitos biomoleculares inerentes às catecolaminas. Estudo recente demonstrou efeito benéfico da levosimendana (na dose 0,1 mcg/kg/min) em relação à melhora da função ventricular e da congestão sistêmica em gestantes com ICA devido à CMPP.^385^

**Tabela 43:** – Posologia dos inotrópicos e vasoconstritores

Inotrópico	Posologia	Dose máxima
Dobutamina	2,5 mcg/kg/min Avaliar ajuste a cada 15 min Efeito hemodinâmico em até 2 h	10 a 20 mcg/kg/min
Milrinona	Início: 0,375 mcg/kg/min Ajuste a cada 4 h	0,75 mcg/kg/min 0,5 mcg/kg/min*
Levosimendana	0,1 mcg/kg/min Ajuste a cada 4 h de 0,05 mcg/kg/min Infusão em 24 h	0,15 mcg/kg/min
Norepinefrina	Início: 0,1 a 0,2 mcg/kg/min Ajuste a cada 15 min	1 mcg/kg/min

* Dose em pacientes com insuficiência renal.

A norepinefrina está indicada na ocorrência de hipotensão arterial importante ou choque cardiogênico, porque, além de ter efeito vasoconstritor, modulando a vasoplegia e redistribuindo o fluxo sanguíneo, tem também efeito sobre o débito cardíaco. Em pacientes refratárias, não responsivas às medidas farmacológicas, o sucesso com uso de dispositivos de assistência circulatória mecânica temporária, tais como balão intra-aórtico (BIA) e oxigenação por membrana extracorpórea (ECMO), tem sido descrito.^386^

### 5.7.2. Arritmia

A principal consideração na prática para arritmias mal toleradas com impacto hemodinâmico é priorizar a vida materna. No entanto, o tratamento também deve ser ponderado em relação aos efeitos colaterais dos medicamentos antiarrítmicos no débito cardíaco e fluxo uteroplacentário, efeitos oxitócicos e efeitos pró-arritmogênicos no feto.

A escolha da medicação antiarrítmica e o ajuste das doses devem ser individualizados a depender da instabilidade hemodinâmica,tipo de arritmia, idade gestacional, presença ou não da doença estrutural materna e do risco de morte súbita cardíaca.^387^

Dentre as TSV, a taquicardia de reentrada nodal é a mais comum, seguida da taquicardia atrioventricular. Em pacientes estáveis, opta-se pela manobra vagal, seguida do uso de adenosina, em bólus na dose de 6 mg e seguida de 12 mg, se houver persistência da arritmia. Dentre os bloqueadores de canais de cálcio, verapamil é opção segura do ponto de vsita obstétrico e fetal. Diante de instabilidade hemodinâmica, está indicada a cardioversão elétrica sincronizada,^388^ sem contraindicação acompanhada pela equipe de anestesia.^74^ A indicação de ablação por cateter deve ser considerada em casos refratários à terapêutica farmacológica utilizando o mapeamento eletromecânico.

A ocorrência de fibrilação ou flutter atrial e taquicardia atrial isoladas não são comuns em pacientes sem lesãocardiaca estrutural durante a gestação. Nestas situações com resposta ventricular acelerada, o risco materno e fetal é alto pela deterioração hemodinâmica. Em todas pacientes deve ser investigado causas como infecções, anemia e tireotoxicose.^389^ Para o controle da frequência da FA com alta resposta ventricular, opta-se pelo uso de lanatosídeo-C, verapamil ou metoprolol e, na vigência de instabilidade hemodinâmica a cardioversão elétrica sincronizada está indicada. Pacientes com FA e valvopatia têm indicação precisa de anticoagulação. Nos clinicamente mais estáveis, em que se opta por controle do ritmo, a cardioversão elétrica é preferencial à química, haja vista o efeito teratogênico da amiodarona e a pouca evidência de segurança em relação à propafenona em dose alta. Nesses casos, quando o tempo de instalação da arritmia excede 48 horas, é necessária a realização do ECO transesofágico.^390^

Para paciente com flutter, a preferência é pela cardioversão, uma vez que sua taxa de reversibilidade é alta, respeitando o tempo de surgimento menor de 48 horas ou após a realização de ECO transesofágico para descartar presença de trombo intracavitário.

A TV pode ocorrer em pacientes portadores de doença cardíaca estrutural e disfunção ventricular e a lidocaína é segura e eficaz em pacientes com instabilidade hemodinâmica. O uso da amiodarona deve ser exclusivo em situações isoladas, em que há refratariedade e recorrência da arritmia ventricular após cardioversão elétrica, considerando-se os efeitos dose-dependentes sobre o feto.^391^ O implante de CDI em pacientes que apresentam indicação, deve ser realizado durante a gravidez para assegurar o melhor resultado do parto e puerpério.^392^

### 5.7.3. Infarto Agudo do Miocárdio

O IAM é pouco frequente durante a gravidez, entretanto nas últimas décadas, verificou-se um aumento na sua incidência, decorrente a faixa etária maior das gestantes com maior exposição aos fatores de risco incluindo os contraceptivos hormonais.^224^

De modo geral, a conduta no tratamento do IAM durante a gestação segue as mesmas orientações para a população geral e em mulheres, incluindo revascularização com angioplastia com “*stent”* ou revascularização cirúrgica.^393^O atendimento multidisciplinar inclui a avaliação obstétrica e a monitorização fetal, com avaliação da vitalidade fetal e a cardiotocografia.

O tratamento clínico do IAM na gravidez considera:^394^

- Oxigenoterapia: cateter nasal de O_2_ de 2 a 3 L/min;
- Controle da dor: sulfato de morfina, seguro e eficaz, mas pode levar a depressão respiratória no feto se administrado próximo do parto;
- Nitratos: atenção quanto ao risco de hipotensão materna e consequente hipofluxo uteroplacentário;
- Betabloqueadores: metoprolol, carvedilol ou propranolol. Recomenda-se o monitoramento fetal com cardiotocografia no controle da dinâmica uterina e dos batimentos fetais;
- Aspirina: baixas doses (< 150 mg);
- O clopidogrel é liberado mas deve ser considerada suspensão de sete dias antes do parto;
- Heparinas: HNF e HBPM são empregadas de acordo com a indicação. O fondaparinux somente deve ser usado quando há contraindicação às heparinas.

O tratamento do IAM com supra do segmento ST é a reperfusão coronariana o mais precoce possível,^389,395^ seja por meio de trombolítico^396^ ou, preferencialmente, pela angioplastia coronária primária com “stents*”*. O trombolítico deve ser restrito aos casos em que a sala de hemodinâmica não esteja disponível em tempo hábil. As restrições ao seu uso se devem ao risco de hemorragia placentária. Se a angioplastia percutânea for indicada, ainda existem controversas sobre a preferência dos “stents*”* convencionais aos farmacológicos.^52^

A estratificação de risco para pacientes com síndrome coronariana aguda sem supra de ST é indicada, como acontece em pacientes não gestantes, considerando idade, sinais vitais, fatores de risco, sintomas recentes ou recorrentes, achados eletrocardiográficos e laboratoriais. Em pacientes gestantes de baixo risco, sem sinais de IC, dor refratária ou instabilidade elétrica, o tratamento clínico conservador é indicado. Por sua vez, em gestantes de alto risco, a estratificação invasiva nas primeiras 24 a 48 horas após o início do quadro agudo deve ser priorizada para prosseguir a revascularização miocárdica.^396^

A dissecção espontânea de artéria coronária é causa frequente entre os casos de IAM na mulher, portanto, deve sempre ser a primeira hipótese diante de um evento isquêmico agudo durante a gestação. O tratamento deve obedecer às medidas recomendadas para pacientes não gestantes.^397^

### 5.7.4. Síndrome Aórtica Aguda

A síndromes aórticas agudas geralmente ocorrem em portadoras de doenças prévias à gestação, entretanto, pode acometer pacientes saudáveis. Estima-se que a incidência de dissecção de aorta na população seja de 2,4 a 2,9 em 100.000 pacientes/ano, e em mulheres abaixo de 40 anos parece existir uma forte correlação com a gravidez.^398^

Dor torácica em portadoras de doenças da aorta exige uma investigação com angiotomografia de aorta, de modo a afastar a suspeita de dissecção aguda de aorta. Em pacientes gestantes com dissecção tipo A, com comprometimento da aorta ascendente, há indicação de cirurgia cardíaca de emergência, além do controle pressórico e da frequência cardíaca. O procedimento deve ocorrer em conjunto com a equipe multidisciplinar e o parto cesárea é indicado quando há viabilidade fetal, seguido de correção da dissecção. Na situação de inviabilidade fetal, realiza-se a cirurgia cardiovascular, considerando-se que a prioridade é materna e que o risco de perda fetal é de 20 a 30%.^399^

Em portadoras de dissecção aórtica tipo B, sem o acometimento da aorta ascendente, não complicada, o tratamento inicial é conservador, mantendo adequado controle da pressão arterial e da frequência cardíaca. O tratamento percutâneo deve ser considerado nas situações tais como; (1) dor persistente; (2) hipertensão arterial não controlada; (3) progressão da dissecção; (4) isquemia em órgão-alvo; (5) sintomas de rotura aórtica.^400^O parto deve ser cesárea após a viabilidade fetal assegurada.

### 5.7.5. Trombose de Prótese Valvar

A incidência de trombose de prótese mecânica durante a gravidez varia de acordo com o esquema de anticoagulação utilizado. O diagnóstico deve ser considerado em gestantes que apresentam dispneia, dor torácica e sintomas de hipotensão. O ECO transesofágico é o exame padrão-ouro na sua definição.^401^

O tratamento da trombose valvar durante a gravidez ou o puerpério deve ser considerado como indicação de procedimento de emergência que depende da condição clinica,o tamanho do trombo e a posição da prótese acometida.^96^

O uso de trombolítico deve ser cogitado em pacientes críticos que apresentem grande risco de morte se forem submetidos à cirurgia, em locais onde não há equipe cirúrgica disponível ou no caso de trombose das valvas tricúspide ou pulmonar. As doses recomendadas de trombolíticos são: estreptoquinase, 1.500.000 UI em 60 min sem HNF; ou alteplase (rT-PA), 10 mg em bólus + 90 mg em 90 min com HNF.^151,402^ Nos casos de sucesso parcial, ou seja, persistência e trombo residual, a paciente deve ser encaminhada à cirurgia após 24 horas da descontinuação da infusão do trombolítico.

Recentemente, foi proposto um protocolo com trombolítico em baixa dose e infusão lenta (rT-PA 25 mg, infusão intravenosa em 6 h, repetindo em 24 h e, se necessário, até 6 vezes, alcançando dose máxima de 150 mg, sem bólus ou uso de heparina concomitante) em gestantes com trombose de prótese. Os resultados mostraram trombólise eficaz, sem mortes maternas e taxa de mortalidade fetal em torno de 20%, que foram melhores do que os das estratégias habitualmente empregadas.^403^ Entretanto, com o aprimoramento das técnicas cirúrgicas, não se pode inferir que a trombólise seja melhor que a cirurgia durante a gravidez.

A inconveniência da cirurgia deve-se à alta mortalidade perioperatória (entre 5 e 18%) estreitamente associada à CF (NHYA*)*, principal variável preditora de risco. As pacientes em CF I/III (NHYA*)* apresentam 4 a 7% de mortalidade, enquanto aquelas em CF IV, 17,5 a 31,3%. No entanto, em comparação com a trombólise, a cirurgia apresenta as maiores taxas de sucesso (81 *versus* 70,9%).^399^ Nesse cenário, a cirurgia de emergência deve ser indicada em pacientes em CFIII/IV (NYHA).

Em pacientes com trombos não obstrutivos, estáveis do ponto de vista hemodinâmico e sem sinais de IC descompensada, opta-se por anticoagulação parenteral em doses terapêuticas, com heparina de acordo com o TTPA e controle de imagem ecocardiográfica. Na falha de resposta ao tratamento, deve ser indicada trombólise ou cirurgia convencional.^151,402^

### 5.7.6. Parada Cardiorrespiratória

**Figura 13:**
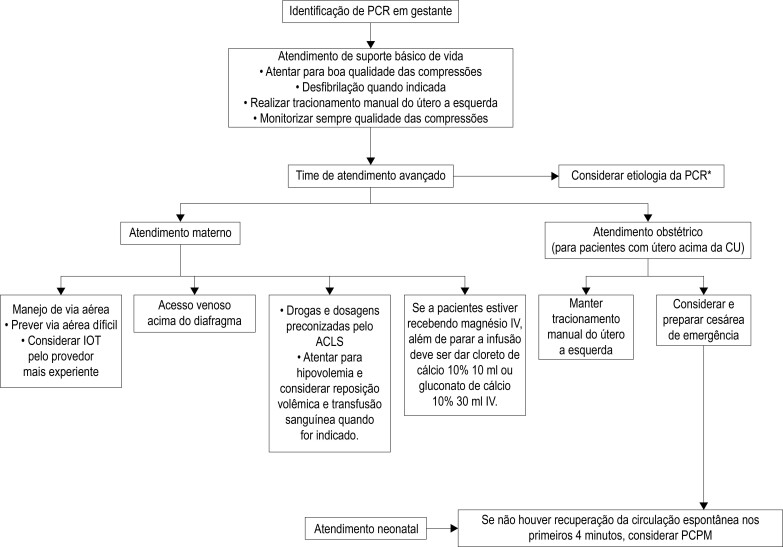
**–** Fluxograma para orientar o atendimento de PCR intra-hospitalar em gestantes. ACLS: Suporte Avançado de Vida em Cardiologia (Advanced cardiology life support); CU: cicatriz umbilical; IOT: intubação orotraqueal; IV: intravenoso; PCPM: parto cesáreo pós-mortem; PCR: parada cardiorrespiratória. * As causas são apresentadas na Tabela 44.

As etapas da ressuscitação cardiopulmonar (RCP) em gestantes são muito similares em relação ao protocolo convencional ditado pelo Suporte Avançado de Vida em Cardiologia (ACLS, *Advanced Cardiac Life Support*), contudo há detalhes resumidos na Figura 13,^404^ que merecem atenção.

É importante lembrar que muitos episódios de PCR são precedidos por sinais de instabilidade hemodinâmica. Por estas razoes, a equipe médica de atendimento que devem receber um treinamento para reconhecer e avaliar as condições clinicas, com intuito da execução sincrônica no atendimento de RCP.^405^

Os efeitos mecânicos do útero gravídico podem agravar a dessaturação e a hipotensão na compressão aortocaval favorecendo o colapso cardiorrespiratório. Na tentativa de reduzir esses efeitos deve ser considerado a manobra manual de tracionamento do útero à esquerda durante todo o atendimento e os cuidados após PCR.^406^

Quando indicado, a desfibrilação deve ser realizada imediatamente, sem demora ou questionamento. Sabe-se que não faz mal ao feto; é completamente seguro, e as doses de energia estabelecidas pelos protocolos atuais devem ser mantidas.^407^

Tal qual a indicação de desfibrilação quanto às doses de energia, as medicações e suas doses devem ser as mesmas definidas pelos protocolos utilizados em adultos.^405,407,408^

A realização por acessos venosos devem ser acima do diafragma, minimizando então os efeitos mecânicos do útero na compressão aortocaval, o que dificulta a recirculação da medicação.^409^

Além de considerar as clássicas causas de PCR preconizadas pelo protocolo do ACLS em gestantes, que utiliza uma regra mnemônica com letras de A a H, há diversas outras condições que podem favorecer o colapso cardiorrespiratório e que podem ser corrigidas^409^ (Tabela 44).

**Tabela 44:** – Principais causas de parada cardiorrespiratória em gestantes e mortalidade materna

Letra	Causas	Etiologia
A	Acidentes complicações anestésicas	Bloqueio neuroaxial mais alto Hipotensão Broncoaspiração Depressão respiratória Obstrução de via respiratória Trauma Suicídio
B	Sangramento (*bleeding*)	Coagulopatia Atonia uterina Placenta acreta Placenta prévia Rotura uterina Descolamento prematuro de placenta Reação transfusional Retenção de produtos da concepção
C	Causas cardiovasculares	Infarto agudo Dissecção de aorta Cardiomiopatia Arritmias Doença valvar Doença cardíaca congênita
D	Substâncias (*drugs*)	Ocitocina Magnésio Drogas ilícitas Opioides Insulina
E	Causas embólicas	Embolia amniótica Embolia pulmonar Evento cerebrovascular
F	Febre	Infecção Sepse
G	Geral	H’s (hipovolemia, hipóxia, hipoglicemia, hipocalemia, hipercalemia, hipotermia) T’s (pneumotórax, tamponamento cardíaco, toxicidade, infarto e tromboembolia pulmonar)
H	Hipertensão	Pré-eclâmpsia Eclâmpsia Síndrome HELLP Sangramento intraparenquimatoso

*HELLP: hemólise, elevação das enzimas hepáticas, plaquetopenia*

A identificação da PCR na gestação, deve considerar a realização do parto cesáreo “perimortem” em gestante com útero acima da cicatriz umbilical.^410^Essa medida se caracteriza por realização de parto cesárea e nascimento do feto após a PCR da mãe, na maioria das vezes durante o período de RCP. Admite-se que o parto cesárea “perimortem*”* está relacionado à sobrevida materna em 31,7% e sem efeito prejudicial à mãe.^411^

Um dos propósitos da cesárea “perimortem*”* é desobstruir a aorta e veia cava dos efeitos compressivos do útero gravídico, quando a sua lateralização é insuficiente para a recirculação materna. O outro propósito é que com o nascimento há redução dos riscos da anóxia fetal durante o período de PCR e, portanto, suas sequelas neurológicas.^412^

A decisão em realizar o parto cesárea de urgência deve ser tomada até os primeiros quatro minutos após a PCR. O parto deve ser no mesmo local do atendimento da RCP, uma vez que a transferência da paciente pode atrasar o atendimento, aumentando os riscos para o feto e comprometendo as manobras de ressuscitação.^409^Vale ressaltar que todo o protocolo de RCP deve ser mantido enquanto o procedimento é realizado. Em situações em que o quadro materno é considerado irreversível, o parto cesárea “perimortem*”* deve ser feito imediatamente.

### 5.7.7. Pontos-chaves

- Em casos de emergência, a conduta deve considerar prioridade materna;
- A conduta de emergência cardíaca durante a gravidez deve obedecer aos protocolos convencionais, como o ACLS;
- Considera-se a cesárea “perimortem” em gestante com altura uterina acima da cicatriz umbilical, no intuito de melhor prognóstico materno-fetal.

## 6. Planejamento Familiar

### 6.1. Aconselhamento à Gravidez e Estratificação de Risco Materno

O aconselhamento na preconcepção é essencial para a mulher cardiopata na idade reprodutiva, com ênfase nos riscos materno e fetal relacionados à gestação e na informação sobre a segurança e eficácia da contracepção. Os critérios de avaliação funcional para se permitir ou contraindicar uma gestação incluem anamnese, exame clínico e exames subsidiários, como ECG, radiografia de tórax, ECO transtorácico ou transesofágico, RMC, teste ergoespirométrico e outros mais específicos. A intervenção invasiva para o eventual tratamento de lesões cardíacas, se indicada, deve ser realizada antes da gestação.

Uma vez determinado o diagnóstico da cardiopatia (anatômico, funcional e sindrômico), pondera-se o risco da gravidez junto ao casal ou aos familiares.^270^ A identificação de preditivos de risco para gravidez contribui para a determinação do prognóstico materno e a tomada de decisões como consentir ou desaconselhar a concepção.

O estudo prospectivo e multicêntrico conhecido como CARPREG^190^ considerou uma casuística constituída por 75% de cardiopatas congênitas e 25% de adquiridas e verificou 13% de complicações cardiovasculares que incluem três mortes maternas. Os preditivos de mortalidade materna propostos nesse estudo estão descritos na Tabela 45.

**Tabela 45:** – Variáveis Preditivas de Eventos Maternos e Escore de Risco do Estudo CARPREG

1. Evento cardíaco prévio (IC, ataque isquêmico transitório, acidente vascular pulmonar prévio à gestação ou arritmia)
2. CF NYHA > II ou cianose
3. Obstrução do coração esquerdo (área mitral < 2 cm^2^, área valvar aórtica < 1,5 cm^2^, gradiente pico na via de saída de ventrículo esquerdo > 30 mmHg)
4. Função sistólica ventricular reduzida (< 40%)
**Escore de risco CARPREG (cada variável soma 1 ponto):**
• pontos – 5% de risco
• 1 ponto – 27% de risco
• Mais de 1 ponto – 75% de risco

*CF: classe funcional; IC: insuficiência cardíaca; NYHA: New York Heart Association.*

Em sequência, o estudo ZAHARA^413,414^ definiu preditivos independentes de mortalidade para portadoras de cardiopatia congênita, gerando uma estimativa de risco muito específica. A taxa de eventos em 1.300 mulheres estudadas foi de 7,6%, e as complicações mais frequentes foram arritmia (4,7%) e IC (1,6%) (Tabela 46), respectivamente.

**Tabela 46:** – Variáveis Preditivas de Risco Materno do Estudo ZAHARA

História de arritmia antes da gestação – **1,5 pontos**
IC com CF NYHA > II – **0,75 pontos**
Obstrução de coração esquerdo (estenose de valva aórtica com gradiente pico > 50 mmHg ou área valvar < 1cm^2^) – **2,5 pontos**
Prótese valvar mecânica – **4,25 pontos**
Regurgitação de valva atrioventricular sistêmica de moderada a grave (possivelmente por disfunção ventricular) – **0,75 pontos**
Regurgitação de valva atrioventricular subpulmonar de moderada a grave (possivelmente por disfunção ventricular) – **0,75 pontos**
Uso de medicação cardiovascular antes da gestação – **1,5 pontos**
Doença cardíaca cianogênica reparada ou não reparada – **1 ponto**
Score de risco **ZAHARA**:
0 a 0,5 – 2,9% de risco
0,51 a 1,5 – 7,5% de risco
1,51 a 2,5 – 17,5% de risco
2,51 a 3,5 – 43,1% de risco
> 3,5 – 70% de risco

*CF: classe funcional; IC: insuficiência cardíaca; NYHA: New York Heart Association.*

A OMS classifica as cardiopatias em nível crescente de gravidade em: (1) Risco I, que inclui as cardiopatias de baixo risco (admitido como igual ao da população geral); (2) Risco II, quando há pequeno risco de mortalidade e moderado risco de morbidade; (3) Risco III, quando existe significativo risco de mortalidade ou grave morbidade; (4) Risco IV, quando a cardiopatia associa-se a alto risco de mortalidade materna (Tabela 47).^415^

**Tabela 47:** – Classificação da OMS modificada

Risco I
• Estenose pulmonar, PCA, prolapso de valva mitral não complicados, de graus leve ou moderados
• CIA, CIV, PCA, drenagem anômala de veias pulmonares não complicadas e reparadas com sucesso
• Extrassístoles atriais ou ventriculares isoladas
**Risco II (não complicadas):**
• CIA e CIV não operadas não complicadas
• Tetralogia de Fallot corrigida
• Maioria das arritmias
**Risco II-III (avaliação individualizada)**
• Leve comprometimento do ventrículo esquerdo
• Cardiomiopatia hipertrófica
• Doença valvar nativa ou protética (não incluída em risco I ou IV [OMS])
• Síndrome de Marfan sem dilatação de aorta
• Valva aórtica bicúspide com diâmetros de aorta < 45 mm
• Coarctação de aorta corrigida
**Risco III**
• Prótese valvar mecânica
• Ventrículo direito sistêmico
• Circulação de Fontan
• Doença cardíaca cianogênica (não reparada)
• Cardiopatias congênitas complexas
• Síndrome de Marfan com diâmetros de aorta entre 40 a 45 mm
• Valva aórtica bicúspide com diâmetros de aorta entre 45 a 50 mm
**Risco IV (contraindicação à gravidez):**
• Hipertensão arterial pulmonar de qualquer etiologia
• Disfunção grave de ventrículo sistêmico (FEVE < 30%, CF III/IV [NYHA])
• Cardiomiopatia periparto com disfunção ventricular
• Estenose mitral grave e estenose aórtica, graves sintomáticas
• Síndrome de Marfan com aorta dilatada > 45 mm
• Dilatação de aorta associada a valva bicúspide > 50 mm
• Síndrome de Turner com aorta indexada > 25 mm/m^2^
• Tetralogia de Fallot com aorta > 50 mm
• Síndrome de Ehlers-Danlos
• Circulação de Fontan com qualquer complicação
• Coarctação de aorta grave

*CF: classe funcional; FEVE: fração de ejeção do ventrículo esquerdo; CIA: comunicação interatrial; CIV: comunicação interventricular, NYHA: New York Heart Association; PCA: persistência do canal arterial.*

A comparação entre os três estudos,^324^ que considerou escore de CARPREG, ZAHARA e OMS, revalidou a classificação pela OMS como a mais aceita e confiável na presunção de risco das cardiopatias à gravidez, apresentada na Tabela 47.

Pacientes incluídas no risco IV-OMS devem ser desaconselhadas à engravidar.^324^ O *Registry of Pregnancy and Cardiac Disease* (ROPAC) validou a classificação da OMS modificada,^416^ que inclui uma categoria intermediária (risco II/III-OMS) que significa risco moderado de morbidade e mortalidade. Esse estudo também mostrou diferenças entre países desenvolvidos e emergentes quanto às características das cardiopatias e aos índices de complicações que podem levar a distorções na interpretação do escore de risco. As Diretrizes da ESC^52^sugerem utilização da classificação da OMS modificada para estabelecimento do risco materno.

O Posicionamento da SBC-2020 considera a classificação da OMS como mais aceita e deve ser aplicada para a estratificação do risco das cardiopatias para a gravidez. Vale considerar que fatores complicadores esperados na história natural das cardiopatias tais como arritmias complexas, antecedentes de IC, tromboembolismo ou EI, modificam a pontuação de risco materno. Os recursos de atendimento e distinção da equipe multidisciplinar especializada também devem ser considerados e individualizados no aconselhamento à gestação.

A Diretriz da ESC^52^ acrescentou as doenças de aorta associadas a síndrome de Turner (tamanho de aorta indexado de 25 mm/m^2^); tetralogia de Fallot (diâmetro de aorta > 50 mm), síndrome vascular de Ehlers-Danlos; e circulação de Fontan complicada, no risco IV-OMS.

#### 6.1.1. Pontos-chaves e Recomendações

- O planejamento familiar é essencial para a portadora de cardiopatia tanto na estratificação de risco à gravidez como na escolha da anticoncepção;
- As variáveis preditivas de risco devem ser definidas antes da gestação;
- A classificação de risco elaborada pela OMS é, no momento, a mais aceita;
- Os recursos de atendimento e a disponibilidade da equipe multidisciplinar especializada devem ser considerados no aconselhamento da gravidez.

## 6.2. Contracepção Não Paciente com Doença Cardiovascular

### 6.2.1. Diferentes Métodos Anticoncepcionais

Contracepção é o uso de métodos e técnicas com a finalidade de impedir que o relacionamento sexual resulte em gravidez. É um recurso de planejamento familiar para a constituição de prole desejada e programada de modo consciente. Atualmente, são conhecidas inúmeras estratégias de anticoncepção, que podem ser agrupadas em métodos hormonais, dispositivos intrauterinos (DIU), comportamentais, de barreira e cirúrgicos.

Os métodos hormonais compreendem aos combinados que são compostos de estrogênio e progestagênio e os de progestagênio isolados. Dentre os combinados, encontram-se as pílulas, os anéis vaginais, os adesivos e os injetáveis mensais e dentre os progestagênio isolados, encontram-se pílulas, os injetáveis trimestrais, o implante subdérmico de etonogestrel e o DIU liberador de levonorgestrel.

A compreensão de que diferentes meios de contracepção apresentam diferentes mecanismos de ação, perfis de eventos adversos, efeitos não contraceptivos benéficos, que variam de acordo com qualquer contexto clínico, é a base para selecionar o método contraceptivo mais adequado; também é indispensável avaliar os desejos e expectativas dos pacientes, além de suas crenças sobre o método, a fim de otimizar a adesão.

**Figura 14:**
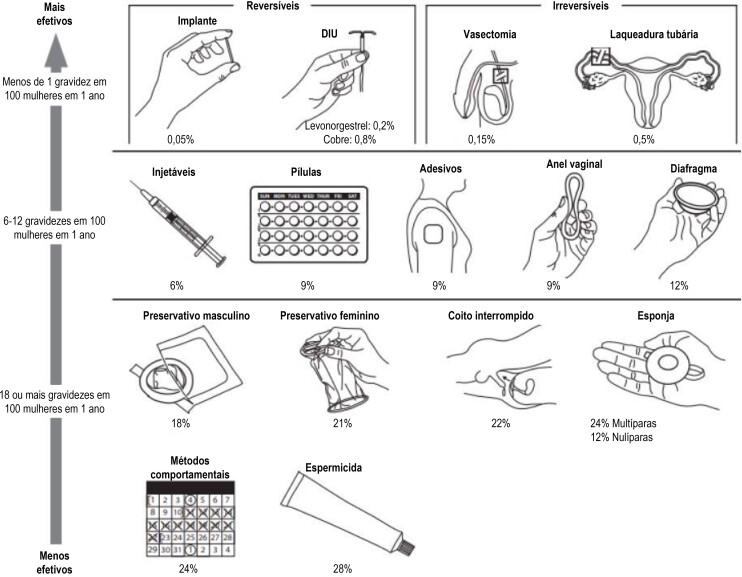
**–** Índices de Pearl dos principais métodos contraceptivos. Adaptada e traduzida de Curtis et al.^417^

Na escolha do métodos considera-se 1) segurança, apoiada nos critérios de elegibilidade dos métodos existentes e disponíveis ; 2) condição clinica da paciente; e, 3) eficácia, baseada no índice de Pearl (número de gestações que ocorrem em cada 100 mulheres utilizando o método ao longo de 12 mesesl^417^ (Figura 14).

Pacientes incluídas no risco III/IV-OMS devem receber orientação para um método contraceptivo seguro, com baixo índice falha e boa aceitação.^418^ Vale lembrar, que estas pacientes têm maior risco cirúrgico, motivo pelo qual os métodos irreversíveis (laqueadura tubária laparotômica, laparoscópica ou histeroscópica) não devem ser recomendados, visto que há outras alternativas de alta eficácia.

Nos últimos anos, um enfoque especial tem sido dado aos métodos de contracepção reversível de longa ação (LARC, “long acting reversible contraception*”*), em razão de: (1) maior adesão porque não dependem da lembrança da usuária; (2) maior eficácia contraceptiva, com menor número de falhas, (3) não conter estrogênio. Essa categoria inclui os dois tipos de DIU (cobre e levonorgestrel) e o implante subdérmico de etonogestrel.^419,420^

### 6.2.2. Critérios de Elegibilidade Médica

A OMS analisou a segurança dos diferentes métodos contraceptivos levando em consideração cada condição clínica e suas características relevantes, incluindo: se o método piora a condição preexistente ou se cria um risco adicional à saúde; e se a condição faz o método contraceptivo menos efetivo.^421^ A segurança deve ser sempre ponderada em comparação com o risco de uma gravidez não planejada. É imprescindível lembrar que, ao recusarmos o acesso das pacientes a todos os métodos contraceptivos por receios relacionados às doenças que possuam, aumenta-se o risco de descompensação dessas doenças caso ocorra uma gravidez.

A Tabela 48 evidencia um resumo das categorias dos critérios de elegibilidade médica para a escolha contraceptiva.

**Tabela 48:** – Categorias dos critérios de elegibilidade médica para escolha contraceptiva

• Condição para a qual não há restrição quanto ao uso do método contraceptivo
• Condição em que as vantagens de usar o método geralmente superam os riscos teóricos ou comprovados
• Condição em que os riscos teóricos ou comprovados geralmente superam as vantagens de usar o método
• Condição que representa um risco à saúde inaceitável se o método contraceptivo for usado

*Adaptada e traduzida de World Health Organization, 2015.^421^*

O acompanhamento de portadora de cardiopatia em idade fértil exige decisões sobre a aplicação de métodos de planejamento familiar e, portanto, orientação sobre contracepção. O estudo pioneiro sobre a eficácia e segurança de contraceptivos que incluiu o combinado oral de baixa dose, o progestágeno injetável trimestral e o DIU em pacientes cardiopatas mostrou boa tolerância e segurança em pacientes que obedeceram aos critérios de elegilibidade.^422^

### 6.2.3. Contracepção em Diferentes Condições

***6.2.3.1. Hipertensão***

Em pacientes hipertensas, o uso de métodos contraceptivos combinados pode piorar o controle pressórico. O etinilestradiol aumenta a síntese hepática do angiotensinogênio, o que leva a um aumento de angiotensina II e aldosterona, com maior volume sistólico e maior débito cardíaco, além de aumento da resistência vascular periférica, resultando em maior pressão arterial. Em pacientes suscetíveis, esse aumento pode ser considerável, causando a descompensação clínica.^423^ Por esse motivo, pacientes com hipertensão, mesmo que controlada, não devem usar métodos combinados; no entanto, não há contraindicação ao uso de métodos contendo somente progestagênio em pacientes com hipertensão controlada e, naquelas não controladas, apenas o injetável trimestral deveria ser evitado. A Tabela 49 evidencia os critérios médicos de elegibilidade dos diferentes tipos de anticoncepção em relação a pacientes com HAS.

**Tabela 49:** – Critérios médicos de elegibilidade dos diferentes tipos de anticoncepção em relação a pacientes com hipertensão arterial sistêmica

	Contracepção hormonal combinada				Contracepção somente com progestagênio			Dispositivo intrauterino	

	Oral	Adesivo	Anel vaginal	Injetável mensal	Oral	Injetável trimestral	Implante subdérmico	Cobre	Levonorgestrel
Antecedente de HAS que não se conhece a PA atual	3	3	3	3	2	2	2	1	2
HAS controlada	3	3	3	3	1	2	1	1	1
HA elevada PAS 140 a 159 mmHg e/ou PAD 90 a 99 mmHg - PAS ≥ 160 mmHg e/ou PAD ≥ 100 mmHg	3	3	3	3	1	2	1	1	1
	4	4	4	4	2	3	2	1	2
Doença de órgão alvo	4	4	4	4	2	3	2	1	2

*HA: hipertensão arterial; HAS: hipertensão arterial sistêmica; PAD: pressão arterial diastólica. Adaptada e traduzida de World Health Organization, 2015.^421^*

***6.2.3.2. Diabetes Melito***

A paciente diabética tem maior risco de eventos cardiovasculares quando comparada com a mulher saudável e está mais exposta a desfechos desfavoráveis durante a gravidez.^424^ Por esse motivo, a contracepção em pacientes diabéticas deve ser orientada pelas melhores evidências disponíveis.^425^ A Tabela 50 resume os critérios de elegibilidade dos diferentes métodos contraceptivos em pacientes diabéticas.

**Tabela 50:** – Critérios de elegibilidade dos diferentes métodos contraceptivos em pacientes diabéticas

	Contracepção hormonal combinada			Contracepção somente com progestagênio			Dispositivo intrauterino	

	Adesivo	Anel vaginal	Injetável mensal	Oral	Injetável trimestral	Implante subdérmico	Cobre	Levonorgestrel
Sem lesão	2	2	2	2	2	2	1	2
2								
Vascular Nefropatia, Neuropatia Ou Retinopatia	3 / 4	3 / 4	3 / 4	2	3	2	1	2
3 / 4								
Outra Vasculopatia	3 / 4	3 / 4	3 / 4	2	3	2	1	2
3 / 4								
ou > 20 anos de doença								

*Adaptada e traduzida de World Health Organization. Medical Eligibility Criteria for Contraceptive Use. 5th ed. Geneva: World Health Organization; 2015.^421^*

Há uma preocupação teórica de que, por seu efeito glicocorticoide, o acetato de medroxiprogesterona de depósito (injetável trimestral) possa contribuir para a piora do controle glicêmico e, nas pacientes com vasculopatia, possa aumentar o risco de eventos tromboembólicos e cardiovasculares, motivo pelo qual é descrito como categoria 3.

***6.2.3.3. Doença Valvar***

As cardiopatias valvares complicadas são classificadas pela OMS como risco III/IV par a gravidez não programada.^415,426^ Apesar disso, vários estudos têm mostrado taxas expressivamente baixas de uso de métodos contraceptivos por pacientes cardiopatas.^422,427^ Para entender os critérios resumidos na Tabela 51, dividem-se as valvopatias em complicadas e não complicadas. São consideradas complicadas aquelas que são acompanhadas de HP, risco de FA e história de endocardite bacteriana subaguda. A Tabela 51 evidencia os critérios médicos de elegibilidade dos diferentes tipos de anticoncepção em relação a pacientes com doença cardíaca valvar.

**Tabela 51:** – Critérios médicos de elegibilidade dos diferentes tipos de anticoncepção em relação a pacientes com doença cardíaca valvar

	Contracepção hormonal combinada				Contracepção somente com progestagênio			Dispositivo intrauterino	

	Oral	Adesivo	Anel vaginal	Injetável mensal	Oral	Injetável trimestral	Implante subdérmico	Cobre	Levonorgestrel
Não complicada	2	2	2	2	1	1	1	1	1
Complicada	4	4	4	4	1	1	1	2	2

*Adaptada e traduzida de World Health Organization. Medical Eligibility Criteria for Contraceptive Use. 5th ed. Geneva: World Health Organization, 2015.^421^*

Até o momento, a indicação de antibioticoprofilaxia no momento da inserção do DIU é controversa, e as evidências disponíveis não parecem justificar obrigatoriedade. A decisão quanto a utilizar ou não fica a cargo do médico assistente, considerando riscos e benefícios associados. No entanto, é imprescindível lembrar que a melhor maneira de evitar infecção pélvica é realizando antissepsia adequadamente.

***6.2.3.4. Evento Cardiovascular Prévio***

Mulheres com coronariopatia isquêmica ou acidente vascular cerebral podem iniciar contracepção contendo somente progestagênio com segurança, com exceção do injetável trimestral; no entanto, se elas apresentarem o evento após a introdução do método contraceptivo hormonal, este deverá ser desaconselhado, e a contracepção não hormonal deverá ser discutida com a paciente. Nesse contexto clínico, os métodos combinados devem ser evitados.^428,429^ A Tabela 52 evidencia os critérios médicos de elegibilidade dos diferentes métodos anticoncepcionais em relação a pacientes com eventos cardiovasculares prévios.

**Tabela 52:** – Critérios médicos de elegibilidade dos diferentes métodos anticoncepcionais em relação a pacientes com eventos cardiovasculares prévios

	Contracepção hormonal combinada				Contracepção somente com progestagênio			Dispositivo intrauterino	

	Oral	Adesivo	Anel vaginal	Injetável mensal	Oral	Injetável trimestral	Implante subdérmico	Cobre	Levonorgestrel
Cardiopatia isquêmica	4	4	4	4	I: 2, C: 3	3	I: 2, C: 3	1	I: 2, C: 3
Acidente vascular cerebral	4	4	4	4	I: 2, C: 3	3	I: 2, C: 3	1	2

*I: iniciar o método; C: continuar o método. Adaptada e traduzida de World Health Organization, 2015.^421^*

***6.2.3.5. Obesidade***

Na ausência de outras condições clínicas, as pacientes obesas não apresentam contraindicação ao uso de nenhum método. Além disso, mesmo que se imponha a investigação de síndrome metabólica e o rastreio de outras condições cardiovasculares pela obesidade, o resultado de exames complementares não deve postergar a introdução de método contraceptivo.^430^

No que diz respeito ao injetável trimestral (dose de 150 mg de acetato de medroxiprogesterona de depósito intramuscular), há casuística brasileira evidenciando ganho de peso significativamente maior em usuárias do injetável trimestral em comparação com as do DIU de cobre.^431^ Por esse motivo, o injetável trimestral não costuma ser nossa primeira escolha; no entanto, não é uma contraindicação formal, e o método pode ser usado.

Especificamente nas obesas, existe uma preocupação teórica de que os métodos possam ser menos eficazes. Ainda que o sejam, sua eficácia continua alta, motivo pelo qual eles não devem ser desaconselhados.

***6.2.3.6. Cardiopatia Congênita***

A orientação à contracepção em portadoras de CC inicia na menarca, com orientação quanto aos riscos da gestação e à seleção do método de contracepção. As CC não são listadas explicitamente nos critérios de elegibilidade da OMS e devem ser entendidas dentro da fisiopatologia que predomina em cada grupo de cardiopatia e do risco da gravidez não planejada (Tabela 53). As CC complexas apresentam lesões estruturais diversas que dificultam a estratificação do risco aos contraceptivos.^415,427^ De todo modo, as cardiopatias cianogênicas, as que apresentam HAP, a síndrome de Eisenmenger e aquelas com risco elevado de tromboembolismo constituem contraindicação absoluta ao uso de métodos combinados. Para esses grupos de pacientes riscoIII/IV-OMS, recomenda-se o uso de progestágenos isolados. Em casos de menor risco de tromboembolismo o injetável mensal pode ser considerado.^432-434^Em pacientes selecionadas a inserção do DIU deve ser hospitalar pelo risco da dor, efeito vagal e arritmias cardíacas, com possibilidade de socorro rápido por anestesiologista com experiência em pacientes cardiopatas.

**Tabela 53:** – Recomendações para o uso de anticoncepcional para pacientes com cardiopatias congênitas

	COs	Mini Pílula	Implante	Depo Provera	DIU	Barreira
**1. Defeitos cirurgicamente corrigidos:**						
Sem lesão residual: CIA/CIV/PCA	1	1	1	1	1	
“Shunt” e/ou obstrução residual	3	1	1	1	3	1
Prótese valvar, tubos, enxertos	2	1	1	1	2	1
Hipertensão pulmonar e/ou sistêmica	4	2	2	2	3	1
**2. Defeitos não corrigidos, resíduo pós-operatório:**						
CIV pequena	2	1	1	1	4	1
“Shunt” discreto à moderado (CIA, CIV, PCA)	4	1	1	1	4	2
Hipertensão residual pulmonar ou sistêmica (CoAo)	2	1	1	1	4	3
Cardiopatias congênitas complexas	4	1	1	1	4	1
**3. Defeitos complicados por:**						
Cianose	4	1	1	1	-	1
Disfunção ventricular	3	1	1	1	-	1
Fibrilação/flutter atrial	4	2	2	2-	4	2
Síndrome de Eisenmenger	4	2	2	2	4	4

*CIA: comunicação interatrial; CIV: comunicação interventricular; CoAo: coarctação de aorta; Cos: pílulas hormonais combinadas; DIU: dispositivo intra-uterino; PCA: persistência do canal arterial.*

***6.2.3.7. Hipertensão Pulmonar***

Esta condição, risco IV-OMS, não está incluída nos critérios de elegilibidade porque a experiência na literatura é muito limitada e está na categoria de contra-indicação à gestação. A contracepção deve ser eficaz, com baixo índice de falha, com boa tolerância para melhor adesão e continuidade do método. Por isso, os métodos de barreira ou com base na “percepção de fertilidade” não são recomendados porque o índice de falha é muito elevado. Dentre os contraceptivos hormonais reversíveis, os compostos que contenham estrogênio não são recomendados devido ao risco de TEP, restando os progestágenos isolados na forma injetável, via oral ou implante subcutâneo, que são os mais indicados.^419,420^ Os DIU-T de cobre têm risco de metrorragia, enquanto os tratados com levonorgestrel (LARC) podem ser recomendados quando a paciente não apresenta lesão cardíaca estrutural.

A gravidez não planejada é muito frequente em mulheres cardiopatas, especialmente pela falta de orientação adequada e o apropriado aconselhamento da contracepção. Na verdade, mitos sobre riscos e o desconhecimento sobre a eficácia e a aplicação dos critérios de elegibilidade são fatores que favorecem a gravidez não programada e a morte materna por cardiopatia na gravidez.^435^

### 6.2.4. Contracepção na Adolescência

A idade isoladamente não representa contraindicação aos diferentes métodos de contracepção, contudo, na adolescência, podem surgir dúvidas quanto à estratégia de apresentação e prescrição dos contraceptivos. A indicação dos métodos deve se apoiar nos critérios de elegibilidade e o atendimento da adolescente deve considerar aspectos ético-legais envolvidos, por vezes desconhecidos.

A Constituição Brasileira, no artigo 226, garante o direito ao planejamento familiar livre de coerção e o Estatuto da Criança e do Adolescente (Lei Nº 8069 de 13 de julho de 1990) dispõe claramente sobre questões importantes no atendimento de adolescentes que requerem métodos contraceptivos, fundamentados nos direitos de privacidade e confidencialidade.

A adolescente tem direito a privacidade, ou seja, de ser atendida sozinha, em espaço privado de consulta. Por sua vez, confidencialidade é definida como um acordo entre médico e paciente, em que as informações discutidas durante e depois da consulta não podem ser informadas a seus pais e ou responsáveis sem a permissão expressa do adolescente.^420^

A confidencialidade apoia-se em regras da bioética médica, através de princípios morais de autonomia (artigo 103 do Código de Ética Médica). Dessa forma, a adolescente tem direito à educação sexual, ao acesso à informação sobre contracepção, à confidencialidade e ao sigilo sobre sua atividade sexual e sobre a prescrição de métodos anticoncepcionais, não havendo infração ética ao profissional que assim procede.

A orientação contraceptiva envolvendo métodos de curta duração, como pílulas, geralmente é realizada sem problemas, seguindo esses preceitos. Por outro lado, os métodos de longa ação (métodos intrauterinos e implantes), por necessitarem de procedimento médico para a inserção, podem suscitar dúvidas. A FEBRASGO sugere que, para esses métodos, deve-se considerar o consentimento da adolescente e do responsável, reforçando o aconselhamento contraceptivo.^436^

No que diz respeito à adolescente portadora de cardiopatia, a anticoncepção deve ser segura e eficaz, no entanto, há uma grande barreira ao conhecimento de diferentes opções e a seu acesso, muitas vezes pelo alto custo inicial. Quando se orienta a contracepção para adolescente com risco III/IV-OMS para a gravidez, há necessidade de apresentar todos os métodos disponíveis que tenham índices baixos de Pearl, boa tolerância e aceitação para continuidade do método, tais como os DIU e implantes. Contudo, as formas mais populares de contracepção em adolescentes são preservativos e o coito interrompido, o que representa um alto índice de falha.

A falta de conhecimento, aconselhamento inadequado, tabus socioculturais, restrições legais e atitudes moralistas quanto a sexualidade na adolescência são comuns, mesmo entre as pacientes que escolhem ou desejam um método contraceptivo. Embora os métodos de longa ação (DIU e implante) sejam priorizados por entidades médicas,^419^ as dificuldades no acesso e na aceitabilidade pela adolescente mostram que métodos tradicionais, como os contraceptivos orais combinados e preservativos, devem também ser foco da orientação, visando melhora das taxas de continuidade e, em última análise, redução da possibilidade de gestações de alto risco e da mortalidade materna por cardiopatia.

***6.2.4.1. Pontos-chaves***

- Existem inúmeros métodos de anticoncepção (hormonais, dispositivos intrauterinos, cirúrgicos comportamentais, de barreira) que podem ser prescritos para a mulher cardiopata;
- A escolha do método de anticoncepção deve considerar a individualidade da paciente (desejo, tolerância), nos critérios de elegilibilidade propostos pela OMS e no índice de falha de Pearl;
- Aspectos éticos e legais devem ser considerados na anticoncepção da adolescente.

## 6.3. Aspectos Bioéticos

O progresso da medicina tornou o provérbio de Michel Peter ultrapassado: *“*Mulher cardiopata, não case; e, se casar, não engravide.” Essa era a verdade há dois séculos para a preservação da vida da mulher jovem portadora de cardiopatia. Atualmente, vivencia-se uma nova era, em que os riscos da gestação, são em geral menores, com recursos para enfrentar a maior parte das eventuais complicações.

A doença cardíaca e gravidez deve ser um tema abrangente, embasado na ética médica, integrando várias fases durante as quais se estabelece a interface multidisciplinar, no atendimento da gestante e do seu recém-nascido. Os médicos devem aplicar rigor científico, com base em recomendações clínicas validadas, esclarecem os benefícios e eventuais riscos e respeitam o direito do paciente de participar livre e ativamente no processo de tomada de decisão, obtendo consentimento informado para todas as situações.

Desde momentos antes da concepção, ocorrem situações que requerem reflexões sobre a segurança materna para a gestação. A avaliação cardiológica pode revelar graus distintos de riscos materno-fetais devido a situações patológicas, clínicas e terapêuticas. As tomadas de decisões resultam em eventuais conflitos na relação médico-paciente, que exigem a referida combinação de aplicação de fundamentos da bioética. A prudência deve predominar, ou seja, a visão do futuro e a previsão da evolução que possam merecer condutas em prol dos benefícios e, no caminho dessa decisão, o consentimento da paciente, irmanado ao seu direito de responder sim ou não.

Além disso, a formação da equipe multidisciplinar é fundamental no que diz respeito ao aconselhamento reprodutivo, com base na estratificação do risco materno, considerando-se o Artigo 226 da Constituição da República Federativa do Brasil de 1988, que determina: “Fundado nos princípios da dignidade da pessoa humana e da paternidade responsável, o planejamento familiar é livre decisão do casal, competindo ao Estado propiciar recursos educacionais e científicos para o exercício desse direito, vedada qualquer forma coercitiva por parte de instituições oficiais ou privadas” (grifos nossos). Essa norma remete a outros itens: a) dignidade da pessoa humana (artigo 1º, III) e b) direito de liberdade (artigo 5°, *caput).*^*”*^

No decorrer da gestação, a relação médico-paciente exige total acolhimento pelo médico e aderência pela paciente, obviamente com adequada disponibilidade de recursos institucionais e do sistema de saúde.

A interdisciplinaridade é desejável em todos os momentos do ciclo gravídico-puerperal; porém, ela amplia seu valor na aproximação do parto, quando é imprescindível a competência profissional da equipe de atendimento. A seleção do momento e tipo de parto, a busca por apoio tecnológico e de infraestrutura em geral são bem auxiliadas pela aplicação da combinação dos fundamentos da bioética.

O puerpério tem peculiaridades específicas sendo que a mãe portadora de cardiopatia demanda um nível de atendimento superior ao habitual, enquanto o recém-nascido já tem vida própria, com suas exigências particulares. Assim, ocorrendo conflitos, como, por exemplo, o não consentimento para a realização de uma recomendação médica, cabe ao médico – ou ao Serviço – fazer uma reavaliação crítica, fundamentada na Bioética à Beira do Leito, para o caso específico. O acordo feito com a paciente deverá ser rigorosamente cumprido pelo médico.
