# Supplementary material for: Posicionamento da Sociedade Brasileira de Cardiologia para Gravidez e Planejamento Familiar na Mulher Portadora de Cardiopatia – 2020
Source: Arq Bras Cardiol. 2020 May 22;114(5):849–942. [Article in Portuguese] doi: 10.36660/abc.20200406 (PMC8386991; doi:10.36660/abc.20200406)
Supplement: Translation

# Translation: Brazilian Cardiology Society Statement for Management of Pregnancy and Family Planning in Women with Heart Disease – 2020

**Authors:** Walkiria Samuel Avila, Elizabeth Regina Giunco Alexandre, Marildes Luiza de Castro, Alexandre Jorge Gomes de Lucena, Celi Marques-Santos, Claudia Maria Vilas Freire, Eduardo Giusti Rossi, Felipe Favorette Campanharo, Ivan Romero Rivera, Maria Elizabeth Navegantes Caetano Costa, Maria Alayde Mendonça Rivera, Regina Coeli Marques de Carvalho, Alexandre Abzaid, Antonio Fernandes Moron, Auristela Isabel de Oliveira Ramos, Carlos Japhet da Mata Albuquerque, Claudine Maia Alves Feio, Daniel Born, Fábio Bruno da Silva, Fernando Souza Nani, Flavio Tarasoutchi, José de Ribamar Costa, José Xavier de Melo, Leila Katz, Maria Cristina Costa Almeida, Max Grinberg, Melania Maria Ramos de Amorim, Nilson Roberto de Melo, Orlando Otávio de Medeiros, Pablo Maria Alberto Pomerantzeff, Sérgio Luiz Navarro Braga, Sonia Conde Cristino, Tania Leme da Rocha Martinez, Tatiana de Carvalho Andreuci Torres Leal

**Affiliations:** 1 Brasil Instituto do Coração (Incor) do Hospital das Clínicas da Faculdade de Medicina da Universidade de São Paulo (HC-FMUSP), São Paulo, SP – Brasil; 2 Brasil Hospital do Coração (HCor), São Paulo, SP – Brasil; 3 Brasil Hospital das Clínicas da Faculdade de Medicina da Universidade Federal de Minas gerais (UFMG), Belo Horizonte, MG – Brasil; 4 Brasil Hospital Agamenom Magalhães, Recife, PE – Brasil; 5 Brasil Universidade Tiradentes, Aracaju, SE – Brasil; 6 Brasil Hospital São Lucas, Rede D'Or Aracaju, Aracaju, SE – Brasil; 7 Brasil Universidade Federal de Minas Gerais (UFMG), Belo Horizonte, MG – Brasil; 8 Brasil Universidade Federal de São Paulo (UNIFESP), Escola Paulista de Medicina (EPM), São Paulo, SP – Brasil; 9 Brasil Hospital Israelita Albert Einstein, São Paulo, SP – Brasil; 10 Brasil Universidade Federal de Alagoas, Maceió, AL – Brasil; 11 Brasil Cardio Diagnóstico, Belém, PA – Brasil; 12 Brasil Centro Universitário Metropolitano da Amazônia (UNIFAMAZ), Belém, PA – Brasil; 13 Brasil Centro Universitário do Estado Pará (CESUPA), Belém, PA – Brasil; 14 Brasil Secretaria de Saúde do Estado do Ceará, Fortaleza, CE – Brasil; 15 Brasil Instituto Dante Pazzanese de Cardiologia, São Paulo, SP – Brasil; 16 Brasil Hospital Metropolitano Oeste Pelópitas Silveira, Recife, PE – Brasil; 17 Brasil Instituto de Medicina Integral Professor Fernando Figueira (IMIP), Recife, PE – Brasil; 18 Brasil Hospital Barão de Lucena, Recife, PE – Brasil; 19 Brasil Hospital EMCOR, Recife, PE – Brasil; 20 Brasil Diagnósticos do Coração LTDA, Recife, PE – Brasil; 21 Brasil Universidade Federal do Pará, Belém, PA – Brasil; 22 Brasil UDI Hospital, Rede D'Or, São Luís, MA – Brasil; 23 Brasil Centro Universitário de Belo Horizonte, Belo Horizonte, MG – Brasil; 24 Brasil Departamento de Obstetrícia e Ginecologia da Faculdade de Medicina da Universidade de São Paulo (USP), São Paulo, SP – Brasil; 25 Brasil Ministério da Saúde, Brasília, DF – Brasil; 26 Brasil Universidade de São Paulo (USP), São Paulo, SP – Brasil

**Development:** Department of Women's Cardiology of the Brazilian Society of Cardiology

**Norms and Guidelines Council:** Brivaldo Markman Filho, Antonio Carlos Sobral Sousa, Aurora Felice Castro Issa, Bruno Ramos Nascimento, Harry Correa Filho, Marcelo Luiz Campos Vieira

**Norms and Guidelines Coordinator:** Brivaldo Markman Filho

**Coordenadoras do Posicionamento:** Walkiria Samuel Avila, Elizabeth Regina Giunco Alexandre, Marildes Luiza de Castro

**Comitê de Redação:** Alexandre Jorge Gomes de Lucena, Celi Marques-Santos, Claudia Maria Vilas Freire, Eduardo Giusti Rossi, Elizabeth Regina Giunco Alexandre, Felipe Favorette Campanharo, Ivan Romero Rivera, Maria Elizabeth Navegantes Caetano Costa, Maria Alayde Mendonça Rivera, Marildes Luiza de Castro, Regina Coeli Marques de Carvalho, Walkiria Samuel Avila

**Table:** 

Declaration of potential conflict of interests of authors/collaborators of the Brazilian Cardiology Society Statement for Management of Pregnancy and Family Planning in Women with Heart Disease – 2020 If, within the last 3 years, the author/collaborator of the statement:							

Names of statement collaborators	Participated in clinical and/or experimental studies sponsored by pharmaceutical or equipment companies related to this statement	Spoke at events or activities sponsored by industry related to this statement	Was (is) a member of a board of advisors or a board of directors of a pharmaceutical or equipment industry	Participated in normative committees of scientific research sponsored by industry	Received personal or institutional funding from industry	Wrote scientific papers in journals sponsored by industry	Owns stocks in industry
Alexandre Abzaid	No	No	No	Boston Scientific, Medtronic, Edwards, Elixir	No	No	No
Alexandre Jorge Gomes de Lucena	No	No	No	No	No	No	No
Antonio Fernandes Moron	No	No	No	No	No	No	No
Auristela Isabel de Oliveira Ramos	No	No	No	No	No	No	No
Carlos Japhet da Matta Albuquerque	No	No	No	No	No	No	No
Celi Marques-Santos	No	No	No	No	No	No	No
Cláudia Maria Vilas Freire	No	No	No	No	No	No	No
Claudine Maia Alves Feio	No	No	No	No	No	No	No
Daniel Born	No	No	No	No	No	No	No
Eduardo Giusti Rossi	No	No	No	No	No	No	No
Elizabeth Regina Giunco Alexandre	No	No	No	No	Novo Nordisk, Servier, AstraZeneca	No	No
Fábio Bruno da Silva	No	No	No	No	No	No	No
Felipe Favorette Campanharo	No	No	No	No	No	No	No
Fernando Souza Nani	No	Boehringer	No	No	No	No	No
Flavio Tarasoutchi	No	No	No	No	No	No	No
Ivan Romero Rivera	No	No	No	No	No	No	No
José de Ribamar Costa Junior	No	No	No	No	No	No	No
José Xavier de Melo Filho	No	No	No	No	Servier, Novartis	No	No
Leila Katz	No	No	No	No	No	No	No
Maria Alayde Mendonça Rivera	No	No	No	No	No	No	No
Maria Cristina Costa Almeida	No	No	No	No	No	No	No
Maria Elizabeth Navegantes Caetano Costa	No	No	No	No	Biolab, Pfizer, Boehringer	No	Speak, Biolab, Bouehringer
Marildes Luiza de Castro	No	No	No	No	No	No	No
Max Grinberg	No	No	No	No	No	No	No
Melania Maria Ramos de Amorim	No	No	No	No	No	No	No
Nilson Roberto de Melo	No	No	No	No	No	No	No
Orlando Otávio de Medeiros	No	No	No	No	No	No	No
Pablo Maria Alberto Pomerantzeff	No	No	No	No	No	No	No
Regina Coeli Marques de Carvalho	No	No	No	No	No	No	No
Sérgio Luiz Navarro Braga	No	No	No	No	No	No	No
Sonia Conde Cristino	No	No	No	No	No	No	No
Tania Leme da Rocha Martinez	No	No	No	No	No	No	No
Tatiana de Carvalho Andreuci Torres Leal	No	No	No	No	No	No	No
Walkiria Samuel Avila	No	No	No	No	No	No	No

Content

1. Introduction 853

2. General Considerations 854

2.1. Physiological Adaptation to Pregnancy, Labor and Delivery 854

**2.1.1. Hemodynamic Modifications 854**

**2.1.2 Modifications in Blood Coagulation 856**

**2.1.3. Respiratory Changes 856**

**2.1.4. Structural Vascular Changes 857**

**2.1.5. Key Points 857**

2.2. Maternal and Fetal Assessment 858

**2.2.1. Maternal Clinical Evaluation 858**

***2.2.1.1. Anamnesis and Physical Examination* 858**

***2.2.1.2. Key Points* 858**

**2.2.2. Fetal and Obstetric Evaluation 858**

***2.2.2.1. Key Points* 860**

2.3. Complementary Cardiovascular Assessment 860

**2.3.1. Electrocardiography 860**

**2.3.2. Echocardiography 860**

**2.3.3. Ambulatory Blood Pressure Monitoring 861**

**2.3.4. 24-hour Holter Monitoring 861**

**2.3.5. Exercise Test 861**

**2.3.6. Key Points 862**

2.4. Ionizing Imaging Cardiovascular Assessment 862

**2.4.1. Administration of Contrast Agents 863**

**2.4.2. Nuclear Magnetic Resonance 863**

**2.4.3. Key Points 864**

2.5. Cardiovascular Drugs during Pregnancy and Breastfeeding 864

**2.5.1. Antihypertensive Drugs 864**

**2.5.2. Antiarrhythmic Drugs 866**

**2.5.3. Drugs in Heart Failure 866**

**2.5.4. Antiplatelet 866**

**2.5.5. Thrombolytics 867**

**2.5.6. Anticoagulants 867**

**2.5.7. Hypolipidemic Agents 868**

**2.5.8. Key Points 869**

2.6. Practice Recommendations during Pregnancy 869

**2.6.1. Lifestyle 869**

**2.6.2. Physical Activity 870**

**2.6.3. Diet 870**

**2.6.4. Professional Activity 870**

**2.6.5. Key Points 870**

2.7. Management of Delivery and Postpartum Period 871

**2.7.1. Practice during Delivery 871**

**2.7.2. Practice during the Postpartum Period 872**

**2.7.3. Key Points 872**

2.8. Obstetrical Anesthesia 873

**2.8.1. Fasting 874**

**2.8.2. Anticoagulation and Neuraxial Block 874**

**2.8.3. Unfractionated Heparin (Subcutaneous) 874**

**2.8.4. Low Molecular Weight Heparin (Subcutaneous) 874**

**2.8.5. Hemodynamic Monitoring 874**

**2.8.6. Intrapartum Uterotonic Drugs 874**

**2.8.7. Postpartum 875**

**2.8.8. Key Points 875**

3. Assessment and Management of Specific Heart Diseases 875

3.1. Valvular heart disease 875

**3.1.1. General Considerations for Treatment 875**

**3.1.2. Key Points 876**

**3.1.3. Valve Prosthesis 876**

**3.1.4. Maternal Risk 877**

**3.1.5. Fetal Risks 878**

**3.1.6. Key Points 879**

3.2. Congenital Heart Disease 880

**3.2.1. Pregnancy Management 881**

**3.2.2. Key Points 883**

3.3. Cardiomyopathies 883

**3.3.1. Dilated Cardiomyopathy 883**

**3.3.2. Hypertrophic Cardiomyopathy 883**

**3.3.3. Arrhythmogenic Right Ventricular Dysplasia 884**

**3.3.4. Non-compaction Cardiomyopathy 884**

**3.3.5. Restrictive Cardiomyopathy 884**

**3.3.6. Key Points 884**

**3.3.7. Peripartum Cardiomyopathy 884**

***3.3.7.1. Key Points* 886**

3.4. Ischemic Heart Disease 887

**3.4.1. Key Points 888**

3.5. Dyslipidemia 888

**3.5.1. Lipid Changes 888**

**3.5.2. Key Points 888**

3.6. Other Diseases 889

**3.6.1. Takayasu Arteritis 889**

***3.6.1.1. Prevalence* 889**

***3.6.1.2. Prognosis* 889**

***3.6.1.3. Treatment* 889**

***3.6.1.4. Key Points* 889**

**3.6.2. Kawasaki Disease 889**

***3.6.2.1. Preconception Evaluation* 890**

***3.6.2.2. Key Points* 890**

**3.6.3. Pulmonary Hypertension 890**

***3.6.3.1. Key Points* 891**

**3.6.4. Aortic Diseases 891**

***3.6.4.1. Aortic Dissection and Rupture* 892**

***3.6.4.2. Key Points* 893**

**3.6.5. Chagas Disease 893**

***3.6.5.1. Prevalence* 893**

***3.6.5.2. Diagnosis and Management of* T. cruzi *Infection during pregnancy* 893**

***3.6.5.3. Chronic Chagas Heart Disease* 893**

***3.6.5.4. Vertical Transmission of* Trypanosoma cruzi 893**

***3.6.5.5. Reactivation of Chagas Disease* 893**

***3.6.5.6. Breastfeeding* 894**

***3.6.5.7. Key Points* 894**

4. Hypertensive syndrome of pregnancy 895

4.1. Introduction 895

4.2. Recommendations for Measuring Arterial Pressure 895

4.3. Classification 895

**4.3.1. Chronic Hypertension, Preexisting (Essential or Secondary) 895**

**4.3.2. Preeclampsia/Eclampsia 895**

***4.3.2.1 HELLP Syndrome (Hemolysis, Elevated Liver Enzymes, and Low Platelet Count)* 866**

**4.3.3. Chronic (Preexisting) Hypertension with Superimposed Preeclampsia 896**

**4.3.4. Gestational Hypertension 896**

***4.3.4.1. Key Points* 896**

4.4. Treatment of Gestational Hypertension Syndrome (GHS) 897

**4.4.1. Non-pharmacological Treatment 897**

**4.4.2. When to Treat – Target Arterial Pressure 897**

**4.4.3. Oral Anti-hypertensive Drugs- Chronic Hypertension /Gestational Hypertension 897**

**4.4.4 Anti-hypertensive Drugs for Severe Hypertension in Preeclampsia 898**

4.5. Practice for Hypertensive Emergency in Preeclampsia (PA ≥ 160/110 mmHg) 899

4.6. Prevention of Seizure in Preeclampsia-Eclampsia and Magnesium SulfateTherapy 900

**4.6.1. Key Points 900**

4.7. Prognosis and Prevention of Preeclampsia 900

**4.7.1. Key Points 901**

4.8. Hypertension during Postpartum Period 901

**4.8.1. Recomendations 901**

**4.8.2. Key Points 902**

4.9. Hypertension during gestational and Future Cardiovascular Risk 902

**4.9.1. Key points 902**

5. Treatment and Prevention of Cardiac Complications 902

5.1. Cardiac Arrhythmias 902

**5.1.1. Epidemiology 902**

**5.1.2. Clinical Presentation 903**

**5.1.3. Maternal-fetal Risks 903**

**5.1.4. Treatment 903**

**5.1.5. Key Points 904**

5.2. Thromboembolism 904

**5.2.1. Epidemiology 904**

**5.2.2. Risk Factors 905**

**5.2.3. Thrombophilia 905**

**5.2.4. Diagnosis 906**

***5.2.4.1. Deep Vein Thrombosis* 906**

***5.2.4.2. D-dimer* 907**

***5.2.4.3. Venous Ultrasound* 907**

***5.2.4.4. Iliac Vein Magnetic Resonance* 907**

***5.2.4.5. Pulmonary Thromboembolism* 907**

***5.2.4.6. Differential Diagnosis* 908**

**5.2.5. Treatment 909**

***5.2.5.1. General Approach* 909**

***5.2.5.2. Heparin Use* 909**

***5.2.5.2.1. Recommended Doses* 909**

***5.2.5.2.2. Labor and Delivery* 909**

***5.2.5.2.3. The Postpartum Period* 910**

***5.2.5.2.4. Period of Anticoagulation* 910**

***5.2.5.3. Inferior Vena Cava Filters* 910**

***5.2.5.4. Thrombolysis* 910**

**5.2.6. Prophylaxis 910**

**5.2.7. Key Points 910**

5.3. Therapy and Prevention 910

**5.3.1. Heart Failure 910**

**5.3.2 Key Points 913**

5.4. Therapy and Prevention 913

**5.4.1 Infective endocarditis 913**

**5.4.2. Rheumatic Disease 914**

**5.4.3. Key Points 915**

5.5. Cardiovascular Surgery During Pregnancy 915

**5.5.1. Key Points 916**

5.6. Percutaneous Cardiac Intervention 916

**5.6.1. General Principles 916**

**5.6.2. Percutaneous Valve Interventions 916**

***5.6.2.1. Balloon Catheter Valvuloplasty in Mitral Stenosis* 916**

***5.6.2.2. Aortic Stenosis* 916**

***5.6.2.3. Congenital Pulmonary Valve Stenosis* 916**

***5.6.2.4. Percutaneous Implantation of Prosthetic Valve* 916**

***5.6.2.5. Valve-in-Valve Procedure for Bioprosthetic Valve Dysfunction* 917**

***5.6.2.6. Coronary Angioplasty* 917**

**5.6.3. Key Points 917**

5.7. Cardiovascular Emergencies 917

**5.7.1. Acute Heart Failure 917**

**5.7.2. Arrhythmia 919**

**5.7.3. Acute Myocardial Infarction 920**

**5.7.4. Acute Aortic Syndrome 920**

**5.7.5. Prosthetic Valve Thrombosis 920**

**5.7.6. Cardiorespiratory Arrest 921**

**5.7.7. Key Points 921**

6. Family Planning 921

6.1. Pregnancy Counseling and Maternal Risk Stratification 921

**6.1.1. Key Points 923**

6.2. Contraception in Patients with Cardiovascular Disease 923

**6.2.1. Different Contraceptive Methods 923**

**6.2.2. Medical Eligibility Criteria 924**

**6.2.3. Contraception in Adverse Conditions 925**

***6.2.3.1. Hypertension* 925**

***6.2.3.2. Diabetes Melittus* 925**

***6.2.3.3. Heart Valve Disease* 925**

***6.2.3.4. Previous Cardiovascular Events* 925**

***6.2.3.5. Obesity* 926**

***6.2.3.6. Congenital Heart Disease* 926**

***6.2.3.7. Pulmonary Hypertension* 927**

**6.2.4. Contraception and Adolescence 928**

***6.2.4.1. Key Points* 929**

6.3. Ethical Considerations 929

References 930

## 1. Introduction

The Women’s Cardiology Department (DCM, acronym in Portuguese) presents this document, composed in accordance with the norms established by the Brazilian Cardiology Society (SBC, acronym in Portuguese), with the aim of discussing the most prevalent cardiovascular diseases that affect women during the pregnancy and for which substantial evidence or randomized clinical trials do not exist.

In 1999, with the support of the SBC, what was at that time the Department of Heart Disease and Pregnancy published the First Consensus on Heart Disease and Pregnancy, which was groundbreaking worldwide. It drew attention to the evolution of gestation in women with heart disease, at a time when the prevailing maxim was “Women with heart disease should not get pregnant because maternal mortality is prohibitive.” After 10 years had passed, the experience of the department that has gone on to become the DCM made it necessary to reconsider the restrictions on pregnancy in women with heart disease. For this reason, in 2009, SBC Guidelines for Pregnancy in Women with Heart Disease published the therapeutic strategies available at that time, in a specific and adequate management of clinical situations.

Two decades after the first publication, the DCM validates its dedication by publishing the First Statement for Management of Pregnancy and Family Planning in Women with Heart Disease, which is the result of the experience and work of specialists who write protocols that contribute to therapeutic decisions during the gestational period, as well as counseling for pregnancy and contraception for women with heart disease.

A country’s maternal mortality rate is one of the most sensitive indicators of its population’s living conditions, and it especially reflects the quality of healthcare provided to women during prenatal care. Although the rates continue to be higher than what was estimated for this millennium, over the past three decades Brazil has registered an important reduction in the rate of maternal mortality due to complications during the pregnancy and postpartum time.

Occurring in 4% of gestations, heart disease in itself continues to be the main non-obstetric cause of maternal mortality worldwide. Nonetheless, cardiology’s advancements in improving diagnostic methods and therapeutic alternatives have promoted significant change in prognosis of cardiovascular diseases and in the characteristics of heart diseases that occur during reproductive age. This has made better life expectancy and quality of life possible for women with heart disease, thus encouraging maternity and promoting safer pregnancies with lower risks.

Medicine is increasingly individualizing the approach to diverse diseases, especially in relation to gender, given that the female organism differs greatly from the male one, especially during the pregnancy-postpartum cycle.

The updating of this document fulfills the universal responsibility regarding improving maternal-fetal prognosis. It is, thus, undeniable that the DCM’s accumulated experience contributes to the establishment of protocols that guide therapeutic practice during pregnancy, to the counseling of future pregnancies, to improvements in life expectancy with quality, and to the reduction in maternal mortality due to heart disease.

In consonance with the international literature, this document discusses new concepts of heart disease *versus* pregnancy, including the following: maternal risk stratification based on the recommendations of the World Health Organization (WHO); aspects of arterial hypertension; reinforcing interdisciplinary approaches, with the participation of a heart team; therapeutic proposals for complications; changes in the classification of maternal-fetal risks with respect to drugs used during pregnancy and breastfeeding; and contraception.

The objectives of this publication are to standardize routine and to divulge yet another tool that will be useful in daily clinical practice. The DCM hopes that the recommendations included in this document will have positive impact throughout Brazil and that they will contribute to better treatment and consequent reduction of cardiovascular risks in childbearing women with heart disease.

## 2. General Considerations

### 2.1. Physiological Adaptation to Pregnancy, Labor and Delivery

Interaction between the embryo and the maternal uterus provokes intrinsic hormonal stimulation in the organism and alterations in the physiology of the cardiovascular system, which are fundamental to the adequate development of pregnancy.^1^These changes, however, lead to a hemodynamic overload that may reveal previously unrecognized heart diseases or aggravate the functional state of underlying heart diseases. For this reason, it is fundamental to comprehend the hemodynamic, blood coagulation, and respiratory modifications that occur during the pregnancy, labor and delivery in order to understand the maternal clinical condition, to predict risks of gestation, and to evaluate fetal health.

#### 2.1.1. Hemodynamic Modifications (Table 1)

**Table 1:** – Hemodynamic alterations during gestation

Parameter	Alteration
Cardiac output	Increased 30% to 50% (2l/min)
Heart rate	Increased 15% to 20% (15 bpm)
Blood volume	Increased 20% to 30% (1.8 l)
Average arterial pressure	Reduced at least 5%
Systemic vascular resistance	Reduced 20% to 30% (320 dynes-s/cm5)
Pulmonary vascular resistance	Reduced 30% (40 dynes-s/cm5)
Central venous pressure	Unaltered
Lower limb venous pressure	Increased 15%

Cardiac output, which is calculated by the product of systolic volume and heart rate, progressively increases, on average, 40% higher than preconception values, beginning of the first trimester of gestation, reaching the greatest increasing in onset of third trimester and tending to reduce at the term of pregnancy^2^(Figure 1). The magnitude of the increase in cardiac output varies individually, and it is 15% greater in multiple pregnancies. Plasma volume is principal responsible for the increase in cardiac output during the first half of gestation. From that moment onwards, heart rate, which does not usually exceed 100 beats per minute (bpm), plays an important role in this increase until the term of pregnancy.

**Figure 1:** – *Variation in cardiac output and peripheral vascular resistance (PVR) during and after pregnancy. Adapted from Sanghavi and Rutheford, 2014.^3^*

The disproportion between increased plasma volume and the production of red blood cells results in haemoglobin dilution or physiological anemia of pregnancy. This is most evident during the end of the second trimester, when plasma volume reaches its peak in relation to the volume of blood cells. When the renal function is normal, blood volume and others elements return to preconception values on account of diuresis, eight weeks after delivery, while hemoglobin begins to increase on the third postpartum day.^2,3^

At the term of pregnancy, blood volume is estimated at 100 ml/kg, almost two times higher than the value of 65 to 70 ml/kg found in women who are not pregnant. Erythrocyte mass begins to increase between the eighth and tenth week of pregnancy, induced by the elevation of plasma erythropoietin.

The hormonal mechanisms of the hypervolemia during pregnancy include increasing levels of estrogen and progesterone, which increases renin levels, causing retention of sodium and total body water; prolactin; placental lactogen; prostaglandins; and the growth hormone.

After the second half of pregnancy, variations may be observed in resting cardiac output consequential to the position adopted by pregnant women. The change from dorsal decubitus to left lateral, for instance, produces an approximately 22% increase in cardiac output, an approximately 6% reduction in heart rate, and a 27% increase in systolic volume. Compression of the inferior vena cava by the enlarged uterus in the supine position provokes what is known as supine hypotensive syndrome, which may manifest with dizziness and/or syncope.^4^

During pregnancy, a reduction occurs in plasma colloid osmotic pressure in approximately 12% to 18% of cases, as a consequence of the drop in circulating albumin concentrations, which are observed in lower levels during week 24 of pregnancy. This decline prompts edema in lower limbs, and it predisposes pregnant and parturient women who have received excessive intravenous crystalloid infusion to pulmonary congestion.^5^

The decrease in peripheral vascular resistance during the beginning of pregnancy is not limited to the uterine plexus, and it has a greater magnitude than the concomitant elevation in cardiac output. During the second half of pregnancy, resistance reaches its lowest values, at the moment when cardiac output reaches its maximum values (Figure 1).^6^ Arteriolar dilation during pregnancy has been attributed to estrogenic components, prolactin, and increased levels of circulating prostaglandin (PGE2 and PGI2), a substance that is responsible for reducing vascular response to exogenous angiotensin.

A decrease in prostaglandin synthesis or an increase in its metabolism may result in increased vascular responsiveness to angiotensin II, a characteristic that has been observed in pregnant women who develop hypertension. Progesterone and its metabolites also appear to participate in modulation of vascular response to angiotensin II during pregnancy. It has recently been demonstrated that alterations in vascular tone during pregnancy may be partly attributed to changes in the synthesis of endothelium-derived vasoactive substances, especially endothelin, which is theoretically capable of mediating prostaglandin synthesis, and to the reduction in nitric oxide, which has been related to vasodilation during pregnancy.^7^

It is worth emphasizing that, during pregnancy, the arterial system undergoes remodeling in order to accommodate increased blood volume. Estrogen promotes collagen deposition in the middle layer of the large and medium arteries; circulating elastase favors rupture of the elastic lamina and weakening of the middle layer of vessel walls; and relaxin, an insulin-like growth factor hormone (detected in the plasma), causes a reduction in collagen synthesis. All of these factors explain the predisposition to artery dissection during pregnancy.

Systemic arterial pressure (SAP) decreases from the beginning to the middle of pregnancy,^8^ particularly at the expense of diastolic pressure, and it subsequently rises to pre-gestation values as the term approaches (Figure 2). SAP rises during uterine contractions, especially during the second stage of delivery.

**Figure 2:** – *Variation in systemic arterial pressure during the pregnancy-postpartum cycle. Adapted from Sanghavi and Rutheford, 2014.^3^*

A clinical picture of orthostatic hypotension may occasionally occur, secondary to reduced venous return when a pregnant woman is in the supine position, with a consequent drop in cardiac output. Considering pulmonary output equal to aortic output in normal adults, changes in pulmonary vascular resistance are parallel to those in systemic vascular resistance.^9^Recent studies have challenged this “dogma,” showing a tendency toward increased arterial pressure in women with body mass index (BMI) > 25 kg/m^2^ and women who were obese prior to gestation.^2^

**Normal labor** is associated with significant hemodynamic alterations, due to anxiety, exertion, pain, uterine contractions, maternal posture (left lateral *versus* supine), uterine involution, and bleeding. During labor, blood from the uterine sinusoids is released into systemic circulation with each contraction, increasing the preload by about 500 ml of blood, which leads to increased cardiac output and blood pressure. Thus, during the second stage of labor, cardiac output is around 50% higher in relation to pre-delivery, and, during fetal expulsion, it is 60% to 80% higher than pre-gestational levels. This abrupt change in cardiac output is transient. It remains elevated during the immediate postpartum period, and it is not accompanied by variations in arterial pressure. During normal delivery, around 400 ml of blood are lost. In cesarean section, blood loss may be greater, namely, around 800 ml. After the delivery, a sudden increase occurs in venous return, due to “auto transfusion” of the uterine plexus, decompression in the flow of the inferior vena cava, and reduction in venous system capacity. In addition to this, peripheral vascular resistance is increased by sustained contraction of the uterus, occluding the vessels that open on the maternal placenta surface. The continuous “auto transfusion” that occurs during 24 to 72 hours after delivery represents a high risk of pulmonary congestion in women with heart disease.^10^

Cardiovascular effects during delivery are also influenced by the eventual occurrence of infection, hemorrhage, and use of anesthetics drugs.^11^

In general, the patterns of alteration in maternal blood volume during labor, the expulsion period, and the postpartum period are described by the following phases:

1. Blood concentration during labor, varying with the degree of uterine activity and maternal dehydration;
2. Reduced blood volume during and immediately after delivery, proportional to blood lose volume;
3. Immediate, transient elevation in blood volume following placental clearance, attributed to fluid inflow into the intravascular territory, due to uterine emptying;
4. Slight elevation in blood volume between the second and third days after delivey, secondary to the transient increase in aldosterone secretion;
5. Reduced plasma volume one week after delivery, in a manner that maternal systolic volume may present a slight drop during this period, returning to normal within a short term.

#### 2.1.2 Modifications in Blood Coagulation

During pregnancy, activation occurs in the synthesis of coagulation factors II, VII, VIII, IX, and X and fibrinogen, as well as a reduction of endogenous anticoagulants (especially antithrombin and protein S), all of which are determinants of the state of hypercoagulability, which is characteristic of a healthy pregnancy.^12^ These modifications occur progressively after the first trimester of gestation, with shortening of prothrombin, partial thromboplastin, and thrombin times, favoring the weakening of the anticoagulant function.^13^ Considering these mechanisms, in conjunction with the mechanical compression of the venous plexus on the lower limbs by the gravid uterus, the characteristic predisposition to thromboembolism during pregnancy is justified. (Figure 3).

**Figure 3:** – *Activation of coagulation factors during pregnancy. F: factor. Adapted from Bremme et al., 2003.^12^*

#### 2.1.3. Respiratory Changes (Figure 4)

**Figure 4:** – *Respiratory changes during pregnancy. ERV: expiratory reserve volume; FRC: functional residual capacity; IC: inspiratory capacity; RV: residual volume; TPC: total pulmonary capacity; VC: vital capacity. Adapted from Hegewald and Crapo, 2011.^16^*

Oxygen consumption increases by around 50%, especially during the last 2 trimesters of gestation, and this is not proportional to maternal weight gain. Weight gain during gestation includes not only fetal metabolic activity, but also the weight of amniotic fluid and the increase of fluid in maternal tissues, both of which are considered metabolically inert. During labor, oxygen consumption increases by 250 to 750 ml/min with each contraction.^14^

The normal respiratory tract undergoes modifications during pregnancy, which induce respiratory alkalosis, with higher arterial oxygen partial pressure (PaO_2_) and lower arterial carbon dioxide partial pressure (PaCO_2_), in comparison with the non-pregnant state. Lower PaCO_2_ favors a diffusion gradient that facilitates the fetus’ ability to eliminate products of aerobic metabolism.^3^

Increased minute ventilation is accompanied by an increase in tidal volume, without modifying respiratory rate. Maternal hyperventilation is considered to be a protective mechanism for the fetus against the detrimental effects of excessive tissue CO_2_ concentration, at the same time that PaO_2_ increases to 100 mmHg.

Modifications in the chest occur with uterine enlargement and diaphragm elevation. Thoracic circumference increases by around 5 to 7 cm; the substernal angle widens, and vertical diameter decreases. These modifications are accompanied by alterations in the distribution of air throughout the diverse pulmonary compartments.

Histological examination of the upper respiratory tract mucosa during pregnancy reveals: hyperemia, glandular hyperactivity, increased phagocytic activity, and increased mucopolysaccharide content. Nasal congestion and epistaxis, which are frequent during gestation, are possibly caused by these changes.^15^ Airway and respiratory function is preserved during pregnancy, as reflected by an unchanged forced expiratory volume in one second (FEV1) and an unchanged ratio of FEV1 to forced vital capacity (FVC).^15^

The 25% reduction in lung functional residual capacity (FRC) is associated with a similar increase in inspiratory capacity (IC). Consequently, vital capacity (VC) does not show any modifications during pregnancy.

The decrease in FRC to 300 ml during pregnancy is not accompanied by increased airway resistance, which, on the contrary, undergoes a significant reduction, possibly due to relaxation of smooth muscle tone secondary to hormonal action. This reduction serves to decrease work of breathing.

With hyperventilation, an increase in PaO_2_ occurs, and the hemoglobin dissociation curve shifts to the right. Normal blood gases in pregnant women should have a pH between 7.40 and 7.47, PaCO_2_ between 30 and 32, and a slight increase in PaO_2_. Respiratory alkalosis is partially offset by increased renal excretion of bicarbonate, which maintains serum levels of HCO_3_ between 18 and 21 mEq/L (a baseline deficit of 3 to 4 mEq/L). The decrease in pulmonary FRC and the increase in oxygen consumption reduce the maternal oxygen reserve, which, in the event of respiratory failure, represents a state of alert for adopting early measures of respiratory or ventilatory support, in order to avoid harm to the fetus or the mother.^16^

The mechanism behind dyspnea during normal pregnancy is not completely clear. Hyperventilation induced by progesterone is probably at least partially responsible, perhaps due to the elevation of ventilation above the level necessary to meet the increased metabolic demand.

#### 2.1.4. Structural Vascular Changes

Hormonal changes during pregnancy may alter the structure of the vascular, resulting in weakening of the arterial walls. Estrogen influences abnormal collagen deposition inside the middle layer of the large and medium arteries. Circulating elastase may provoke a rupture of the elastic lamina and weakening of the middle layer of vessel walls. In addition to this, relaxin, an insulin-like growth factor hormone (detected in the plasma), causes a redction in the synthesis of collagen and predisposes pregnant women to artery dissection.^17^

#### 2.1.5. Key Points

Knowledge regarding physiological modifications related to the pregnancy and postpartum period is fundamental to clinical practice for management of pregnancy and risk stratification of women with heart disease.

## 2.2. Maternal and Fetal Assessment

### 2.2.1. Maternal Clinical Evaluation

***2.2.1.1. Anamnesis and Physical Examination***

Initial clinical investigation of pregnant women with heart disease requires questions about family history with respect to genetically transmittable heart diseases. Family history of the following stand out: premature sudden death, cardiomyopathy, congenital heart disease, Marfan syndrome, long QT syndrome, catecholaminergic ventricular tachycardia (VT), and Brugada syndrome.

Physiological modifications during pregnancy influence the evaluation of cardiovascular status, and specialized knowledge is required to differentiate between healthy and unhealthy patients (Table 2).

**Table 2:** – Clinical evaluation of normal pregnant women

Symptoms	Signs
Diminished physical exercise capacity	Hyperventilation
Dyspnea	Limb edema
Fatigue	Distention of neck veins
Palpitation	Pulmonary base crackles
Dizziness	Ictus cordis shifted to the left
Orthopnea	Palpable right ventricular impulse
Swelling in the legs	Pulmonary trunk impulse

*Adapted from Davies et al., 2007.^19^*

Complaints of shortness of breath (hyperventilation), easy fatigue, decreased functional exercise capacity, and basal crackles that disappear with coughing or deep breathing are symptoms that arise with uterine growth and its mechanical effect on diaphragm compression, especially toward the end of gestation. In addition to this, peripheral edema and varicose veins are frequent during later stages of gestation. Systemic arterial pulse is characterized by a rapid increase and a rapid collapse (“small battering ram”) starting with the first trimester.

During chest palpation, cardiac ictus is noted to be shifted to the left, anterior, and rotated in the direction of a transverse position to the extent that the uterus enlarges. As a result, the apical impulse is shifted to the fourth intercostal space, laterally to the hemiclavicular line. The left ventricular impulse is relatively hyperdynamic, but it is not sustained; the right ventricle may be palpable, because, like the left ventricle, it supports a greater volume of blood, which is ejected against relatively low resistance. As pregnancy advances, enlargement of the breasts and the abdomen makes precise heart palpation difficult and at times impossible.^18^

The changes in auscultation that accompany normal gestation begin at the end of the first trimester and generally subside within one week of delivery. Higher basal heart rate, higher precordial heart sounds, split first and second sounds in the third trimester, and systolic ejection murmurs (as high as grade 2/6) above the pulmonary and tricuspid areas are regularly detected during cardiac auscultation. The third sound may be present in the majority of pregnant women; the fourth heart sound is rarely heard, and it is, in general, pathological. Venous hum is almost universal in healthy women during normal gestation, and it is most audible over the right sternal border. The hum is attributable to an increase in venous return. Breast murmur (systolic or continuous) is audible over the anterior thorax at the end of gestation, and it is peculiar to pregnancy due to increased mammary blood flow. It is especially common after childbirth in breastfeeding women.^20^

Diastolic murmurs are not common in normal gestations. When they occur, they may reflect an increase in flow through the tricuspid or mitral valve or physiological dilatation of the pulmonary artery. Alternatively, these murmurs may represent a pathological condition, requiring investigation with further examinations.^20^

The hyperdynamic state of pregnancy may manifest with episodes of tachycardia, and baseline resting heart rate may oscillate around 90 bpm. Bradycardias are rare; when they occur, more detailed investigation is necessary. Sinus rhythm should be prevalent among pregnant women, but the presence of supraventricular or ventricular extrasystoles is very common.

When measuring pregnant women’s blood pressure, the fourth Korotkoff sound is accepted as diagnosis of diastolic pressure. After this point, the sounds begin to change, and it is not easily reproducible at times. For this reason, it is fundamental to measure arterial pressure in the left lateral decubitus position using a standardized method. Arterial hypotension is a common finding during the first trimester, continuing until week 22 to 24, with arterial pressure returning to pre-pregnancy levels near the term of pregnancy.

***2.2.1.2. Key Points***

- Detailed anamnesis considering current and past symptoms;
- Family history;
- Detailed physical examination to differentiate between normal and heart disease.

### 2.2.2. Fetal and Obstetric Evaluation

Obstetrical and perinatal complications are significantly greater in women with heart disease – the leading cause of maternal death during the pregnancy-postpartum cycle. The lack of healthcare protocols for pregnant women with heart disease and fragile multidisciplinary interaction contribute to these poor outcomes in pregnancy. Within this scenario, it is necessary to develop healthcare protocols aligned with prevention and treatment of complications during pregnancy, delivery, and the postpartum period, for pregnant women with cardiac disease. The Heart Disease and Pregnancy Service of the Obstetrics Department of Universidaddse Federal de São Paulo, in this document, have proposed a protocol presented in Figure 5.

**Figure 5:** – *Evaluation and practice for women with cardiovascular disease. ECG: electrocardiography; MFM: Maternal-fetal medicine; NYHA: New York Heart Association; WHO: World Health Organization.*

The care plan includes the following: preparation and readiness for delivery at a reference hospital; routine compliance on the part of anticoagulated patients and patients in premature labor; prevention of postpartum hemorrhage (PPH); and infective endocarditis (IE) prophylaxis.

The principal maternal factors that affect fetal growth and development are low cardiac output (heart failure [HF] and obstructive cardiac lesions), hypoxemia (pulmonary hypertension [PH] or cyanotic heart disease), medication use (anicoagulants, beta-blockers, diuretics, or antiarrhythmics), heredity (genetic transmission), maternal infections (by *Trypanosoma cruzi* [*T. cruzi*]), and obstetric complications (Table 3).

**Table 3:** – Predictors of neonatal events in pregnant women with heart disease.

NYHA functional class III/IV
Cyanosis
Obstructive cardiac lesions
Tobacco use
Hypoxemia – oxygen saturation < 90%
Need for permanent anticoagulation
Abnormal uteroplacental blood flow – by Doppler scan
Maternal infections (*by Trypanosoma cruzi*, human immunodeficiency virus, or toxoplasmosis)
Parents with congenital heart disease
Medication use during pregnancy (ACEI, ARB, or beta-blockers)
Obstetric complications – arterial hypertension, gestational diabetes

*ACEI: angiotensin converting enzyme inhibitors; ARB: angiotensin receptor blockers; NYHA: New York Heart Association.*

Fetal consequences include greater frequency of prematurity, intrauterine growth restriction (IUGR), miscarriages, cardiac and non-cardiac anomalies, and death. Maternal clinical complications associated with low cardiac output lead to a greater frequency of low birth weight –, with an average weight 300 g lower when compared to pregnancies that progressed without complications – and Apgar score less than 7.^21^

Maternal hypoxemia in women with cyanotic heart disease increases fetal risk, even though there is a compensation mechanism to facilitate fetal oxygen delivery. Most newborns with maternal hypoxemia are small for gestational age and premature. A higher frequency of miscarriages has also been observed, proportional to the elevated hematocrit and maternal hemoglobin levels.

Anticoagulant use during pregnancy causes expressed fetal loss. It is estimated that the incidence of spontaneous abortion during the first trimester is 28.6% *versus* 9.2% in pregnant women using warfarin *versus* heparin, respectively.^22^ Sodium warfarin, when used during the first trimester, causes fetal warfarin syndrome in 5% to 10% of cases. This occurs between the sixth and ninth week of gestation^23^ (Table 4). The incidence is variable, as, in many cases, the syndrome may not be identified from the clinical point of view; according to geneticists’ evaluation, however, the frequency is much higher. The risk of warfarin syndrome, when compared to the general population, is OR 3.86 (1.86-8.00- IC 95%). For these reasons, these children should receive detailed genetic evaluation during early childhood, and their scholar development should be followed.

**Table 4:** – Fetal warfarin syndrome23

Affected bones/cartilage (chondrodysplasia punctata)
Hypoplasia of extremities (dwarfism and bone dystrophy) Optical defects: blindness, optic atrophy, microphthalmia
Central nervous system: mental retardation, deafness
Intrauterine growth restriction
Scoliosis
Congenital heart disease
Death

Newborns whose mothers have used amiodarone, sotalol, angiotensin converting enzyme inhibitors (ACEI), angiotensin receptor blockers (ARB), or other medications during gestation should receive specific assessment of these drug-related abnormalities during the neonatal period. For patients who have been previously operated or who have undergone previous blood transfusion, it is essential to investigate hepatitis B and human immunodeficiency virus (HIV) infections, given that the use of drugs for these conditions may decrease vertical transmission.

The frequency of fetal evaluation should be decided by the obstetrician according to case severity and the parameters to be evaluated. Severe patients, including those with New York Heart Association (NYHA) functional class (FC) III/IV, severe obstructive valve disease, cyanotic heart disease, complex congenital heart disease, and PH, may require fetal reassessment by ultrasound, as often as weekly. Fetal Doppler of uterine arteries during the second trimester aims to predict preeclampsia. This includes evaluation of the umbilical, middle cerebral, and uterine arteries, the cerebroplacental ratio, and the ductus venosus (Table 5).

**Table 5:** – Fetal procedures

First trimester ultrasound (establish gestational age)
Second trimester ultrasound (analysis of fetal morphology)
Doppler ultrasound of uterine arteries to predict preeclampsia
Fetal Doppler ultrasound starting at week 26 (biweekly or weekly in severe cases)
Fetal echocardiography (pregnant women with congenital heart disease): week 20
Third trimester ultrasound (fetal growth profile and fetal biophysical profile) biweekly starting at week 26 in severe cases

During fetal ultrasound evaluation, it is important to estimate gestational age, vitality, morphology, amniotic fluid volume, and fetal growth profile. In the event that an abnormality is detected, evaluation should be complemented by more specific examinations, such as fetal Doppler, fetal biophysical profile, and fetal echocardiography (echo).^24^The latter should be performed routinely, always after week 20, when there are maternal or fetal indications. Maternal indications are diabetes mellitus, a parent with congenital heart disease, maternal infection related to teratogenicity (rubella, cytomegalovirus, HIV), Chagas disease and toxoplasmosis (related to cardiomyopathy or fetal myocarditis), maternal age > 35 years, phenylketonuria, connective tissue disease (most associated with fetal atrioventricular block), and exposure to teratogenic agents. Fetal indications for complementary evaluation include findings of other abnormalities on morphological study, chromosomal disease, and fetal arrhythmias.

***2.2.2.1. Key Points***

- Perinatal morbimortality is higher in newborns whose mothers have heart disease, in comparison with the general population;
- Multiple maternal factors are associated with the higher incidence of fetal loss, malformations, IUGR, and prematurity;
- Obstetricians and neonatologists must be attentive to neonatal complications related to maternal heart disease.

## 2.3. Complementary Cardiovascular Assessment

### 2.3.1. Electrocardiography

Electrocardiography (ECG) is the first method used for diagnostic confirmation in clinical cardiology practice. The criteria for performing ECG in pregnant women are the same as those defined for the general population; it should not, however, be part of routine prenatal screening for heart disease. ECG should serve as evaluation and follow up for pregnant women with previous heart disease and for investigation of arrhythmias.^25^

Physiological changes during gestation should be considered in the interpretation of the ECG record. The following stand out: electrical axis slightly shifted to the left; T-wave inversion in the DIII, V1, V2, and, at times, V3 leads; prominent q wave in the inferior and anterolateral walls; increased P-wave duration and prolonged QT interval.^26^Measurements of P-wave duration and QT interval during the 3 trimesters of pregnancy have shown prolongation of the P wave during the second trimester, followed by a plateau, as well as prolongation of the maximum QT interval at the term of the gestation.^27^

ST-segment depression may be observed in 25% to 47% of pregnant women during cesarean delivery or 30 minutes afterwards, regardless of the type of anesthesia utilized. No alterations suggestive of ischemia have been observed during vaginal birth in healthy pregnant women.^26,27^

### 2.3.2. Echocardiography

Echo is the examination of choice for diagnostic investigation of most heart diseases, owing to its easy use, the absence of maternal-fetal risks, and lower costs when compared to other methods. Indications are the same as those for the general population,^28^ for initial diagnosis when heart disease is suspected, for risk stratification by measurement of ejection fraction or global longitudinal strain, and for determining clinical therapeutic practice or percutaneous or surgical intervention in cases with important stenoses in mitral and aortic valves.

Pregnancy hypervolemia may cause slight dilatation of the cardiac chambers (up to 20% in the right chambers and 10% to 12% in the left chambers), mild mitral and tricuspid valve reflux, appearance of minimal physiological transvalvular gradients, and increase in prior valve gradients, as in obstructive lesions of the heart.^29^

Transesophageal echo is relatively safe, and its conventional indications continue to apply;^29^ the risk of vomiting and aspiration mainly increases after week 20 of gestation. This requires the presence of an anesthetist, who will assist in selecting the most appropriate sedation, in controlling ventilation, and in fetal monitoring during the procedure.

During the final periods of gestation, small pericardial effusions may be perceived as a consequence of excessive hydrosaline retention, which disappears during the postpartum period. These effusions are not pathologically significant, and they are usually asymptomatic; they do, however, require reevaluation 6 weeks after delivery.

Fetal echo may be used to detect congenital heart disease, and it may be performed transvaginal beginning with week 12 of gestation and transabdominally beginning with week 18. Although the main indication for fetal echo is the presence of an alteration in the routine ultrasound examination, some maternal indications are important, including pre-gestational diabetes mellitus or diabetes identified during the first trimester of gestation, phenylketonuria, systemic lupus erythematosus (SLE), and Sjögren syndrome with positive anti-SSA and/or anti-SSB antibodies.^30^ In these cases, both the presence of fetal complete atrioventricular block during a previous gestation and neonatal SLE increase the possibility of fetal involvement during a subsequent gestation, or, at times, alteration in fetal cardiac rhythm determines better maternal evaluation for investigating autoantibodies.

Other indications are cases of assisted reproduction, maternal congenital heart disease, infections such as rubella during the first trimester of gestation, or other viruses, when there is a suspicion of associated fetal myocarditis or pericarditis. Less precise indications refer to maternal medication use during the first trimester of gestation, such as anticonvulsive drugs, lithium, ACEI, retinoic acid, vitamin A, paroxetine, and non-hormonal anti-inflammatory drugs, due to risks of both fetal malformation and ductal constriction.^31^

### 2.3.3. Ambulatory Blood Pressure Monitoring

Ambulatory blood pressure monitoring (ABPM) is considered a safe examination. It is mainly indicated for identification of early arterial hypertension, which occurs during the first 20 weeks of gestation. It is estimated that approximately one third of pregnant women present white coat hypertension, almost half of whom may develop true arterial hypertension which requires treatment.^32^

Blood pressure monitoring during different trimesters has shown conflicting results regarding pressure behavior, and it has little utility for identifying pregnant women who develop late hypertension or even for predicting adverse events in hypertensive patients. Reference values are the same as those used for the general population, and there are no studies which recommend routine use of ABPM for diagnosing or monitoring blood pressure as a substitute for conventional measurement with a tensiometer.^33^

### 2.3.4. 24-hour Holter Monitoring

Holter monitoring is mainly used for detecting or stratifying arrhythmias during gestation. It is mainly indicated for investigation of palpitation, unexplained syncope, or pre-syncope, or, less frequently, investigation of neurological events in whose etiology atrial fibrillation (AF) may be implicated.^34^

Holter monitoring is an examination method for identifying and characterizing arrhythmias as simple or complex and symptomatic or asymptomatic, which is fundamental information for practice during pregnancy. It is particularly indicated for investigating paroxysmal AF, other tachyarrhythmias, symptomatic sinus bradyarrhythmias, and different degrees of atrioventricular block. Holter is also of great value for evaluation of patients with a pacemaker or an implantable cardioverter-defibrillator (ICD) when symptoms such as palpitation, syncope, or pre-syncope occur, or when there is suspicion of device command failure.

### 2.3.5. Exercise Test

The main indication for ergometric test during gestation would be investigation of ischemic coronary disease. Performance of a submaximal test, reaching 80% of maximum expected heart rate, seems to be a safe method during gestation, but the lack of studies does not make it possible to validate its indication for defining ischemic disease. For this reason, there are no recommendations for performing exercise tes during gestation to investigate ischemic coronary disease. In the same manner, the use of stress with dobutamine should be contraindicated during pregnancy.

In contrast, during preconception, abnormal chronotropic response identified on ergometric test in women with heart disease seems to be predictive of adverse events during future pregnancies. In the same line of investigation, ergospirometry test is valid for evaluation of myocardial reserve, especially in women with congenital heart disease.^35^

### 2.3.6. Key Points

- ECG and echo should be indicated when heart disease is suspected;
- Fetal echo is indicated for congenital heart disease or when fetal involvement is assumed due to maternal disease;
- 24-hour Holter monitor assists in identification and stratification of cardiac arrhythmias;
- The main indication for ABPM is identification of “early” arterial hypertension, which occurs during the first 20 of gestation;
- Exercice testing is not indicated for investigation of ischemic coronary disease during gestation;
- Ergospirometry testing assess to risk stratification for women with heart disease when planning gestation.

## 2.4. Ionizing Imaging Cardiovascular Assessment

The use of ionizing imaging diagnosis in adult with heart disease corresponds to 12% of all examinations to which these patients are exposed^36^ and to 40% of the total dose of radiation which they will receive during their lifetime.^37^ For this reason, they are a cause of concern regarding the safety of examinations that emit radiation during pregnancy and lactation.

Measurements of ionizing radiation may be in sieverts (Sv), which express the equivalent dose of radiation in the tissue, or in gray (Gy), which indicate total radiation dose. Sv is the measure of greatest biological significance.^38^

There are two biological effects of radiation, namely, the deterministic effect, which leads to cell death when the maximum recommended dose of radiation is exceeded and which becomes evident after a few days, weeks, or months of the procedure (cataract, leukopenia, anemia, sterility, and others); and the stochastic effect, which causes cellular transformation with random alteration in single-cell DNA (deoxyribonucleic acid) that continues to reproduce. When the damage occurs in germ cells, genetic or hereditary effects may occur. There is no dose threshold, and damage may be caused by a minimal radiation dose. Moreover, the effects are difficult to measure experimentally due to the long latency period. The main examples include cancer (leukemia from 5 to 7 years, solid tumors from 5 to 10 years or more) and genetic effects. It has been verified that stochastic effects are highest in children and higher in women than in men, and they are reduced by 50% among octogenarian men.^39^

During pregnancy, the biological effects of radiation on the embryo depend on dose and gestational age, and they may be divided into the following 4 categories: intrauterine death, malformations, growth and developmental disorders, and mutagenic and carcinogenic effects.^40,41^

It is accepted that the non-carcinogenic risk, which includes miscarriage and malformations, is insignificant at doses below 50 mGy, in comparison with other risks of pregnancy. In contrast, it is estimated^42^ that doses above 100 mGy present potential effects on the fetus/embryo in accordance with gestational age, such as fetal death when exposure occurs between the first and second week of gestation; severe abnormalities in the central nervous system (hydrocephalus, microcephaly, and mental retardation) between weeks 3 and 15; mental retardation, microcephaly, and fetal growth restriction between weeks 16 and 30; following week 32 of gestation, teratogenic effects are absent, but there continues to be an increased risk of developing malignancy during childhood and adulthood. Indication for interrupting gestation may be considered in cases of radiation doses between 100 and 500 mGy, based on individual circumstances, such as maternal malignant diseases that require serial imaging during gestation, interventional procedures, or radiation therapy.^43^

Accordingly, it is important to remember that the natural incidence of congenital anomalies in the general population generally varies between 0.5% and 5%, and exposure to a radiation dose of 10 mGy is associated with 0.5%, 0.4%, and 0.1% probabilities of malformations, microcephaly, and mental retardation, respectively.^41^In this line of investigation, studies have demonstrated that uterine exposure to even low radiation doses (20 mGy) increases the risk of cancer during childhood and the occurrence of leukemia, by a factor of 1.5 to 2.0, when compared to the natural incidence of these diseases.^43^ The main radiological methods and the doses of radiation absorbed by the fetus, the patient, and the breasts (during lactation) are shown in Table 6.

**Table 6:** – Radiation doses associated with radiological examinations

Modality	Fetal dose (mGy)	Maternal dose (mSv)	Breast dose (mGy)
**Tomography**			
Pulmonary angiography	0.01 to 0.66	2.7 to 40	8 to 70
Abdomen and pelvis	13 to 25	3 to 45	–
Angiography of thoracic and abdominal aorta, with or without contrast agent	6.7 to 56	4 to 68	16 to 130
Coronary artery angiography	0.1 to 3	7 to 39	10 to 90
Simple abdomen and pelvis computed tomography	10 to 11	3 to 10	–
**Nuclear medicine**			
Low-dose perfusion scintigraphy	0.1 to 0.5	0.6 to 1.0	0.1 to 0.3
V/Q scintigraphy	0.1 to 0.8	1.2 to 2.8	0.2 to 0.7
Myocardial viability PET with ^18^F-FDG	6.8 to 8.1	7	–
Myocardial perfusion with ^99m^Tc-sestamibi	17	11.4 to 14.8	–
Myocardial perfusion with ^99m^Tc-tetrofosmin	8.45	9.3 to 11.6	–
**Radiography**			
Mammography, 2 positions	0.001 to 0.01	0.1 to 0.7	3
Thorax radiography, 2 positions	0.0005 to 0.01	0.06 to 0.29	< 0.4
Abdominal radiography	0.1 to 0.3	0.01 to 1.1	–

*FDG: fluorodeoxyglucose; PET: positron emission tomography; V/Q: ventilation/perfusion. Note: Estimated doses vary according to protocol, radiotracer, dosage, dose calculation method, and patient-dependent factors (e.g. body weight and tissue percentage of the mammary gland).*

It is necessary to remember that no single radiological examination exposes the fetus to doses above 250 mGy, which could occur as a result of a combination of examinations or during the course of a treatment that is essential to the mother.

When they do not directly involve the uterus or direct abdominal exposure, fluoroscopy, radiography, cardiac catheterization, and interventional radiology result in radiation doses that are not very significant to the fetus. Accordingly, it is necessary to consider strategies^44^ that may reduce radiation, at times by around 30% to 65%. The following stand out: use of lead protectors on the abdomen, X-ray beam collimation in the area of interest, use of permanently calibrated and measured equipment, preference for digital radiography, and reduction of fluoroscopy time and number of images acquired. Furthermore, enlargements should be carried out using a lower number of images and exposures.

In nuclear scintigraphy examinations, fetal ionizing radiation exposure comes from accumulated radioactivity in the maternal organism and from radiopharmaceutical transport and diffusion through the placenta.^45^ Ventilation/perfusion (V/Q) scintigraphy is the most frequent scintigraphy imaging method with reduced maternal dose, compared to computed tomography pulmonary angiography (CTPA). CTPA, however, provides lower doses when the fetus is still small and farther from the field of view or the thorax.

V/Q scintigraphy and CTPA are efficacious for diagnosing pulmonary embolism during pregnancy, although CTPA demonstrates advantages for identifying other pulmonary diseases. When clinical suspicion of pulmonary embolism exists, simple chest X-ray and bilateral lower limb Doppler ultrasound are considered to be the initial examinations for guiding indication for V/Q scintigraphy, which should be preferable to CTPA when both are available.^45^ Pharmacological stress with the use of vasodilators, either adenosine or dipyridamole, is not recommended during gestation, owing to the risks resulting from orthostatic hypotension.

### 2.4.1. Administration of Contrast Agents

Iodinated contrast agents do not present any teratogenic effects, and they may be used orally or intravenously in cases where examination information is important for immediate management; otherwise, the examination should be postponed until after delivery.^46^ This is due to the fact that fetal thyroid maturation begins at week 12, and it functions minimally at week 20 of gestation. There is, thus, a concern that iodinated contrast agents might induce development of hypothyroidism, even though, over the past 3 decades, there have been no reports of this occurring in this situation. In cases of allergic reaction to the contrast, phenylephrine and corticosteroids may be used safely. In preventive situations, prednisone and dexamethasone should be considered, given that most of these agents are metabolized in the placenta before reaching the fetus. There are, however, case reports of fetal adrenal suppression with corticosteroid use, and methylprednisone has been correlated to cleft lip when used before 10 weeks of gestation.^43^

More recently, multislice tomography, with multiple rows of detectors, has been used, providing undeniable advantages, especially related to speed and definition in abdominal and angiographic studies (CT angiography). These benefits, however, have been accompanied by a significant increase in absorbed radiation doses in abdominal organs of around 90% to 180%, when compared to helical devices with a single row of detectors. At the same time that multislice technology is consolidated as an extremely useful tool for thoracic-abdominal studies, it is necessary to invest in optimizing and adjusting protocols with the aim of controlling and limiting emitted radiation dose, especially during gestation.

### 2.4.2. Nuclear Magnetic Resonance

Cardiac magnetic resonance (CMR) is advisable in cases where other non-invasive methods have not been sufficient to define diagnosis, and it is preferable to imaging examinations that emit ionizing radiation. Exposure during the first trimester of gestation has not been associated with harmful effects to fetuses or children during early childhood.

Evidence regarding the use of gadolinium contrast during pregnancy is controversial. Gadolinium (Gd+3) is a paramagnetic metal ion whose pharmacological behavior in the organism is similar to that of iodinated contrast medium, i.e., it acts as an extracellular agent, rapidly spreading from the intravascular compartment to the interstitial space. No mutation or teratogenic effects have been documented following inadvertent administration of gadolinium-based contrast media during pregnancy. Nevertheless, depending on the dose, its use appears to be associated with greater risks of rheumatic, inflammatory, and infiltrative cutaneous manifestations, in addition to fetal loss.^47^

In its free form, the gadolinium ion is neurotoxic; its bond to a chelating agent, however, forms a stable complex, thus protecting the organism from adverse effects. Gadolinium chelates cross the placental barrier, and they may accumulate in the amniotic cavity; nonetheless, some studies have shown that only 0.01% of the dose is present in fetal circulation 4 hours after contrast administration, and only traces are detected after 24 hours.

During lactation, both iodinated contrast agents and gadolinium have low lipid solubility, and their concentration in breast milk is lower than 1% and 0.04%, respectively.^46^ For this reason, the American Academy of Pediatrics and the WHO recommend not suspending lactation.

Obtaining patients’ informed consent and clarifying the inherent risks of tests that are necessary for medical practice are essential measures that should be part of the interdisciplinary decision to indicate radiation examination during gestation, which involves the obstetrician and the radiology team.

### 2.4.3. Key Points

- Indication for a radiological examination should consider the real benefit for determining therapeutic practice during pregnancy and the impossibility of substitution with an alternative radiation-free method (ultrasound, echo, and magnetic resonance);
- The radiologist is the professional who is most prepared to evaluate the best diagnostic option in a given clinical situation, ensuring the safety of the pregnant woman and the fetus;
- Radiological examinations should be performed in institutions that are able to guarantee the adoption of effective protection measures and that have modern equipment that is regularly calibrated and measured;
- CMR is a complementary examination for defining diagnosis of heart disease. It is safe during gestation. Nevertheless, the use of gadolinium should be avoided;
- The need for an examination with radiation demands interdisciplinary discussion involving a radiologist, a cardiologist, and an obstetrician, in addition to the patient’s informed consent.

## 2.5. Cardiovascular Drugs during Pregnancy and Breastfeeding

Requirement of pharmacological therapy is very frequent during pregnancy and lactation.^48^It is estimated 34% of pregnant women with heart disease use cardiovascular medications, with the following distribution: beta-blockers (22%), antiplatelet drugs (8%), diuretics (7%), ACEI (2.8%), and statins (0.5%).^49^ In this series, the prevalence of adverse events to the fetus, especially IUGR, was twice as high when compared to the women who did not take medication.^50^

It is estimated that 10% to 15% of women with heart disease present cardiac complications that lead to medication treatment during gestation like systemic arterial hypertension (SAH), cardiac arrhythmias, HF, and thromboembolism.^51,52^ However, prescription drugs during pregnancy requires basic knowledge of pharmacokinetics and drug classification for maternal and fetal safety during pregnancy and lactation.

The pharmacokinetics of medications are influenced by the physiological changes of pregnancy, often leading to a reduction in drug plasma concentration such that any dose adjustments should be considered to achieve therapeutic efficacy.^53^Table 7 summarizes^54^ the aspects deserve the following considerations:

**Table 7:** – Pharmacokinetics during pregnancy

**Decreased absorption**	Delayed intestinal motility
**Increased distribution volume**	Reduced peak concentration of hydrophilic and lipophilic drugs and half-life variations
**Increased hepatic metabolism**	Reduced plasma concentration of drugs that pass through the liver
**Increased renal clearance**	Reduced plasma concentration of drugs with renal excretion. Tubular absorption/excretion function is variable

*Adapted from Feghali et al., 2015.^54^*

- Absorption of orally administered drugs is reduced due to delayed intestinal motility.^55^ Besides, the use of antacids and iron as a supplement appears to induce drug chelation at increased gastric pH, resulting in reduced drug bioavailability;^56^
- The volume of drug distribution is increased during pregnancy due to plasma volume expansion contributing to a reduction in peak drug concentration;^57^
- Liver metabolism is accelerated during pregnancy because liver perfusion is greater. This means that the fraction of the drug removed from the liver circulation is increased such that drugs such as propranolol, nitroglycerine, and verapamil are extracted faster from the systemic circulation.^54^Drugs such as warfarin, which do not depend on flow but on liver activity and plasma free fraction are not influenced their concentration during pregnancy. On the other hand, nifedipine and metoprolol plasma levels are reduced in pregnancy due to increased enzyme catalytic activity;^58^
- 85% increase in renal blood flow compared to pre-gestational levels.^59,60^ However, a tubular function is variable, with a reduction in uric acid excretion and glucose absorption, and an increase in protein excretion.^61^

As for safety, most drug studies in pregnancy are performed on animals and have little applicability because the effects are generally species-specific. Human studies are almost always retrospective and include small series. Pregnant women, except in rare circumstances, are excluded from large clinical trials. Thus, the medical literature on drugs in pregnancy has, for the most part, questionable scientific evidence.

In 1979, the Food and Drug Administration (FDA)^62^ introduced the classification of drugs according to categories A through X, which are widely used in daily practice.^55^ This classification labeled drugs according to animal and female studies in categories that they ranged from drugs that did not pose a risk to the fetus (category A) to teratogenic ones (category X).

In 2015, the classification (A, B, C, D, and X) was replaced by the so-called Pregnancy and Lactation Labeling Rule (PLLR),^63^ which is currently being accepted more. It provides a descriptive summary and detailed information on animal studies and clinical trials, as outlined in Table 8.

**Table 8:** – Pregnancy and Lactation Labeling Rule – Food and Drug Administration

Required information
**Related to pregnancy:** Risk of medication use, compatibility with lactation, reproductive potential in men and women, information about pregnancy tests and contraceptive use
**Risk summary:** Systemic absorption of the drug during pregnancy, labeled data from studies in humans and animals and adverse fetal outcomes, including fetal loss and malformation
**Contraindicated during pregnancy:** Structural anomaly, embryopathy or fetal and neonatal mortality, functional impairment (multiple organ toxicity), growth alterations, retardation, or prematurity
**Clinical considerations:** Essential guidelines for prescription considering dose adjustments during pregnancy and postpartum, associated maternal disease and/or risk of fetal embryopathy, adverse maternal and fetal reactions, and effects of medication during labor and delivery
**Additional data:** Information from studies in humans and animals that support previously presented declarations of risk
**Pregnancy exposure registry:** Information for healthcare professionals, with toll-free telephone number for obtaining information about the registry
• Data
• Human
• Animal

### 2.5.1. Antihypertensive Drugs (Table 9)

**Table 9:** – Effects of antihypertensive drug use during pregnancy and lactation

Drugs	Used during pregnancy	Maternal-fetal effects	Lactation
ACEI and ARB	No	Dysgenesis and renal insufficiency Congenital cardiovascular and neurological malformation	Compatible (captopril, enalapril, losartan)
Amlodipine	Yes	Non-teratogenic	Probably compatible
		Limited data in humans	
Atenolol	No	IUGR	Compatible, but with caution (safer options)
		Bradycardia and fetal hypoglycemia	
Metoprolol succinate	Yes	Low birth weight and IUGR Bradycardia and fetal hypoglycemia	Compatible, but with caution (effects of the beta-blocker on the newborn)
Nifedipine	Yes	Probably low risk during all phases of gestation	Compatible
Methyldopa	Yes	Probably low risk during all phases of gestation	Compatible
Clonidine	Yes	Probably low risk during all phases of gestation	Compatible
Verapamil	Yes	Probably low risk during all phases of gestation	Compatible
Sodium nitroprusside	Yes – risk of fetal cyanide exposure	Congenital malformations have not been described Cyanide accumulation	Not compatible
Furosemide	Yes	Reduced amniotic fluid	Compatible*
Hydrochlorothiazide	Yes	No evidence of teratogenicity Risk of hypovolemia	Compatible*
Hydralazine	Yes	Neonatal thrombocytopenia and lupus-like syndrome	Compatible
Spironolactone	No (antiandrogenic activity)	No evidence of teratogenicity Antiandrogenic activity (feminization of male fetus)	Not recommended

*ACEI: angiotensin converting enzyme inhibitors; ARB: angiotensin receptor blockers; IUGR: intrauterine growth restriction.*

**Nifedipine**: hypotensive and tocolytic action; not teratogenic. May require shortening of intake range or higher dose due to CYP3A4-mediated accelerated hepatic metabolism. Increased hypotension with concomitant magnesium sulfate use.^64-66^

**Methyldopa (**β**2-adrenergic receptor agonist):** Non-teratogenic, considered safe and effective in the treatment of gestational hypertensive disease with favorable outcomes in primary and secondary outcomes such as blood pressure control, fetal growth, and prematurity. Warning maternal effects such as postural hypotension, lupus-like syndrome, depression, nasal congestion, drowsiness, and liver toxicity have been reported in 1% of treated cases.^67,68^

**Hydralazine:** direct arteriolar vasodilator for oral or intravenous use in hypertensive emergencies. Adverse effects are maternal symptoms of "like" lupus and fetal thrombocytopenia.^69^

**Clonidine:** alpha-2 agonist has divergent hemodynamic effect in reducing vascular resistance versus reduction in cardiac output, and the consequent impact on fetal growth. Abrupt suspension may cause rebound hypertension. It is not teratogenic. It is available transdermal.^70^

**Diuretics** are indicated in hypervolemia and HF; however, the reduction in plasma volume, cardiac output, and placental flow is the major restriction on diuretic use during pregnancy. Its use during pregnancy has not been related to detrimental effects to the fetus. Furosemide is the most commonly used, while hydrochlorothiazide has been related to lower birth weight, jaundice, and neonatal thrombocytopenia.^71^

**Beta-blocker:** atenolol is not recommended because its use is associated with IUGR and low birth weight newborns.^71^

**Amlodipine:** may be considered second-line treatment without reference to being teratogenic when used in the first trimester of pregnancy.^71^

**ACEI, ARB, direct renin inhibitors and aldosterone antagonists** are contraindicated in pregnancy and should not be prescribed in women who wish to become pregnant. These medications cause renal dysgenesis, oligohydramnios, renal failure, IUGR, neonatal anuria, and fetal death, particularly in the second and third trimesters of pregnancy.^72^However, ACE inhibitors may be used in lactation. Aldosterone antagonists have antiandrogenic effects on the male fetus and are contraindicated in lactation.^52,73^

### 2.5.2. Antiarrhythmic Drugs (Table 10)

**Table 10:** – Effects of the use of antiarrhythmic drugs during pregnancy and lactation

Drugs	Used during pregnancy	Maternal-fetal effects	Lactation
Lidocaine	Yes	Non-teratogenic; in high doses, respiratory depression and fetal acidosis have been described	Compatible
Propafenone	Yes	No data during the first trimester; no complications during the other trimesters	Probably compatible
Propranolol	Yes	Low birth weight and IUGR Bradycardia and fetal hypoglycemia	Compatible, but with caution (effects of the beta-blocker on the newborn)
Sotalol	No	Low weight, IUGR, torsades de pointes when associated with hypomagnesemia	No
Amiodarone	No	Fetal hypo- and hyperthyroidism, low birth weight, long QT	No

*IUGR: intrauterine growth restriction.*

**Adenosine:** nucleoside with a half-life of seconds. It is safe, but adverse effects include bradyarrhythmias, dyspnea, chest pain and flushing.^74,75^

**Beta-blockers**: These are the most commonly used drugs during pregnancy. They are not teratogenic. Controlled studies show a higher frequency of neonatal bradycardia and hypoglycemia, as well as a higher risk of prematurity and small newborns for gestational age.^76-78^

**Atenolol:** hydrophilic with renal elimination, is contraindicated by the high risk of IUGR.^79,80^**Propranolol** is safe; however, depending on the dose, IUGR, hypoglycemia, polycythemia, and hyperbilirubinemia may occur.^81^**Metoprolol** is well tolerated, with high clearance in the second half of pregnancy. Succinate is safer than tartrate because doses are lower and maybe fractionated.^82,83^ Sotalol is associated with point torsades due to QT interval prolongation. **Sotalol** is higher in breast milk and should be suspended during lactation. In cases of lactation maintenance, electrocardiographic control should be performed in the mother and the newborn. According to ESC Guidelines, 2018,^52^sotalol was contraindicated in pregnancy and lactation because of the risk of sudden maternal-fetal death. The proposal is a replacement for propafenone or flecainide. However, the restriction on the use of sotalol during pregnancy and lactation is still controversial, as the results in controlling complex arrhythmias have been satisfactory in the practice of the specialists. Although there are no adequate studies, sotalol appears to be safer compared to amiodarone.^84-85^

**Amiodarone**: lipophilic, accumulates in skeletal muscle and adipose tissue, with half-life from weeks to months. Warning effects are thyroid dysfunction (causing neonatal hypothyroidism in 17 to 25% of cases) and impaired neurological development. Should be contraindicated in pregnancy.^86-87^

**Lidocaine**: more studied as an anesthetic agent than antiarrhythmic. Sixty percent of it is bound to plasma protein and rapidly entering the maternal circulation and placenta. It may lead to depression of the fetal central nervous system when used at high doses.^73,88^

**Propafenone**: recommended for the prevention of supraventricular tachycardia in patients with Wolff Parkinson White syndrome, atrial tachycardia and atrial fibrillation refractory to nodal blocking agents.^52^

**Procainamide**: associated with maternal lupus syndrome.^89^

### 2.5.3. Drugs in Heart Failure (Table 11)

**Table 11:** – Effects of heart failure treatment during pregnancy and lactation

Drugs	Used during pregnancy	Maternal-fetal effects	Lactation
Isosorbide mononitrate	Yes	Headache, hypotension, non-teratogenic	Compatible
Hydralazine	Yes	Neonatal thrombocytopenia and lupus-like syndrome	Compatible
Carvedilol	Yes	Low birth weight and IUGR Bradycardia and fetal hypoglycemia	Compatible
Metoprolol succinate	Yes	Low birth weight and IUGR	Compatible, but with caution (effects of the beta-blocker on the newborn)
		Bradycardia and fetal hypoglycemia	
Bisoprolol	No	Low birth weight and IUGR	Compatible (effects of the beta-blocker on the newborn)
	Risk/benefit	Bradycardia and fetal hypoglycemia	
Digoxin	Yes	Non-teratogenic	Compatible
Dobutamine	Yes	Non-teratogenic in animals	Probably compatible
Milrinone	No	Risk in animals	Probably compatible
	Risk/benefit	No evidence in humans	
Sacubutril/valsartan	No	The same as ARB; inadequate data on sacubitril	No
Ivabradine	No	Cardiac defects in animals	No
		IUGR	
		Bradycardia in newborns	

*ARB: angiotensin receptor blockers; IUGR: intrauterine growth restriction.*

**Carvedilol:** There is a lack of studies. It is the first choice of cardioselective beta-blocker. It is not teratogenic and has not been associated with IUGR.^90^

**Bisoprolol**: Not associated with an increased risk of miscarriage or fetal malformation when used in the first trimester of pregnancy. However, IUGR cannot be ruled out during prolonged use throughout pregnancy.^52,78^

**Hydralazine:** A drug that replaces ACEI and ARB.^52^

**Nitrates:** Not routinely used and not teratogenic. Low maternal tolerance due to hypotension and headache.^52^

**Sacubitril / valsartan:** is contraindicated during pregnancy and although there are no studies on human milk excretion, there is no recommendation for use during breast-feeding.

**Ivabradine**: Animal studies show its association with malformation, bradycardia and altered fetal growth.

### 2.5.4. Antiplatelet (Table 12)

**Table 12:** – Effects of antiplatelet use during pregnancy and lactation

Drugs	May be used during pregnancy	Maternal-fetal observations and effects	Lactation
Aspirin	Yes	Hemorrhage	Compatible
Clopidogrel	Yes (benefit greater than risk)	Hemorrhage	Probably compatible
Prasugrel	No Risk/benefit	No evidence in humans	Probably compatible
Ticagrelor	No Risk/benefit	No evidence in humans	Probably compatible
Ticlopidine	No	Thrombocytopenia, neutropenia	No
Tirofiban	Yes (benefit greater than risk)	Hemorrhage	Compatible
Abciximab	Yes (benefit greater than risk)	Hemorrhage	Compatible
Epifibatide	Yes (benefit greater than risk)	Hemorrhage	Compatible

**Aspirin:** It is safe at low doses at any stage of pregnancy.^52,91,92^ Not teratogenic, risk of maternal and fetal bleeding. Should be discontinued five days before delivery.^52,93,94^

**Clopidogrel:** There are no studies to ensure its use during pregnancy. It does not appear to be teratogenic. The use of clopidogrel should be recommended in very specific cases, as discontinuation of clopidogrel may impair the treatment of the disease for which it is indicated.^93,94^

### 2.5.5. Thrombolytics (Table 13)

**Table 13:** – Effects of thrombolytic drug use during pregnancy and lactation

Drugs	May be used during pregnancy	Maternal-fetal observations and effects	Lactation
Streptokinase	Yes (benefit greater than risk)	Risk of hemorrhage	Compatible
Tenecteplase	Yes (benefit greater than risk)	Risk of hemorrhage	Compatible
Alteplase	Yes (benefit greater than risk)	Risk of hemorrhage	Compatible
Urokinase	Yes	Proteinase inhibitors in the placenta inactivate urokinase	Compatible

They do not cross the placental barrier but are at risk of maternal bleeding.^52^

### 2.5.6. Anticoagulants (Table 14)

**Table 14:** – Effects of anticoagulant use during pregnancy and lactation.

Drugs	May be used during pregnancy	Maternal-fetal observations and effects	Lactation
Warfarin	No	Warfarin syndrome during the first trimester, other congenital and neurological anomalies during the other trimesters	Compatible
	Risk/benefit		
Heparin	Yes	Thrombocytopenia	Compatible
Enoxaparin	Yes		Compatible
Fondaparinux	Yes	Various reviews suggest safety during pregnancy	Compatible
Apixaban	No	Low risk in animals, evidence does not support safety in humans	No
Dabigatran	No	Moderate risk in animals, evidence does not support safety in humans	No
Rivaroxiban	No	Low risk in animals, evidence does not support safety in humans	No

When used in the first trimester, causes fetal warfarin syndrome in 5 to 10% of cases.^95^ The incidence is variable because the syndrome from the clinical view can often be inconspicuous, although in the opinion of geneticists. , its frequency is much higher. The risk of spontaneous abortion (less than 20 weeks of gestation) is almost 30% and that of stillbirth (more than 20 weeks of gestation) is 10%, both caused by warfarin poisoning.^96^ Maternal hemorrhage in delivery patients warfarin is serious; more severe, however, is neonatal intracranial hemorrhage and its sequelae.^96,97^ For patients on oral anticoagulants who undergo premature labor, cesarean section is indicated.

The hypothesis that doses of less than 5 mg warfarin may cause a lower risk of embryopathy^52^ is not supported by appropriate studies to guide anticoagulation guidance in the first trimester of pregnancy. The teratogenic property of a drug is understood to be independent of its dose. Recent reports have shown the occurrence of embryopathy even at doses below 5 mg warfarin.^98^ It is concluded that it is “good practice” that the warfarin dose is adequate in pursuit of the therapeutic goal, controlled by the index. International Standardized Study (INR) and individualized for each clinical situation.

**Heparin**: does not cross the placental barrier. Table 15 presents the advantages of low molecular weight heparin (LMWH) over unfractionated heparin (UFH). Both are associated with a 10 to 15% risk of miscarriages due to placental bleeding.^100^ Permanent use of UFH during pregnancy presents maternal risks such as bleeding (2%); osteoporosis (30%); spontaneous fractures (2%) and thrombocytopenia (5 to 15%)^101^ However, it appears that these adverse effects are minor with LMWH. Control of UFH anticoagulation should be daily, according to activated partial thromboplastin time (TTPA), with a target of 1.5 to 2 times greater than baseline. Control of LMWH should be weekly according to therapeutic values between 0.6 and 1.2 IU/ml of anti Xa factor in patients with mechanical valve prostheses.

**Table 15:** – Comparison between unfractionated heparin and low molecular weight heparin

Peculiarities	UFH	LMWH
Molecular weight	12,000 to 14,000	4,000 to 6,000
Anticoagulant action	Thrombin and Xa	Xa
Bioavailability	30%	100%
Half-life following application	45 to 60 min	12 h
Absorption following SC injection	Variable	100%
Thrombocytopenia	27%	0%
Monitoring	APTT	Anti-Xa factor
Cost	Low	High
Control frequency	Higher	Lower
Control	1.5 to 2 × baseline	7 to 12 u/ml

*APTT: activated partial thromboplastin time; LMWH: low molecular weight heparin; SC: subcutaneous; UFH: unfractionated heparin. Adapted from Ginsberg et al., 2003.^100^*

Anticoagulation is still a therapeutic challenge. It requires knowledge of the risk of thrombosis for each clinical situation and the side effects of anticoagulants at various moments of the pregnancy-puerperal cycle. The fact is that when there is an indication of anticoagulation, pregnancy should not influence the accuracy and conventional goals. Fondaparinux has proven to be a safe alternative when heparins are not tolerated.^52,102^

**New oral anticoagulants (NOACS):** There is no data available on exposed pregnant women. These drugs should not be used during pregnancy**.**

### 2.5.7. Hypolipidemic Agents (Table 16)

**Table 16:** – Effects of hypolipidemic agent use during pregnancy and lactation

Drugs	Used during pregnancy	Maternal-fetal effects	Lactation
Statins	No	Low risk of teratogenicity and toxicity	No
Fibrates	No	Teratogenicity in animals, with no evidence in humans	No
Ezetimibe	No	Low risk in animals, current evidence does not support use during gestation	No
Alirocumab	No	Low risk in animals, current evidence does not support use during gestation	No
Cholestyramine	Yes	Possible reduction in vitamin absorption	Probably compatible

The first choice is cholestyramine, considered the safest.^103^Statins do not appear to be teratogenic. Its correlation with congenital anomalies is not clear; however, due to the lack of studies, its use should be discouraged during pregnancy and should be discontinued at conception.^104-106^ Gemfibrozil, fenofibrate and ezetimibe are considered to have teratogenic potential.^73^

The treatment of **pulmonary arterial hypertension** (PAH) will be discussed in topic 3.6.3. Drugs released for use during pregnancy include:^73,107^

- Prostaglandins: epoprostenol, treprostinil, Iloprost;
- Phosphodiesterase 5 inhibitors: sildenafil and tadalafil;
- Calcium channel blockers (BCC): diltiazem and nifedipine;
- Nitric oxide: via inhalation.

**Contraindicated drugs** during pregnancy are:^73,107^

- Endothelin receptor antagonists: bosentan, ambrisentan, and macitentan;
- Guanylate cyclase stimulators: riociguat.

### 2.5.8. Key Points

The basic recommendations for prescribing medications during pregnancy, when possible, are:

- Considerer pharmacokinetics of drugs during pregnancy, before prescription;
- Prescribe when treatment is indicated, and its benefit outweighs the potential risk;
- Guide to the prescription by the PLLR classification;
- Avoid medication in the first trimester of pregnancy;
- Use the lowest dose, as long as it is effective, for the shortest time, and, fractionate the daily dose;
- Use drugs that are already widely accepted and safe in pregnancy;
- Consider that drugs with molecular weight less than 1,500 Da cross the placenta and reach fetal circulation;
- Guide preconception for women who make permanent use of medicines;
- Consider that the priority of treatment is maternal, but obstetric and fetal risks should be considered.

## 2.6. Practice Recommendations during Pregnancy

### 2.6.1. Lifestyle

Pregnancy is an ideal moment for lifestyle change. This is because pregnant women, owing to concerns about their children’s health, are motivated to improve unhealthy habits, for example, quitting smoking and alcoholism, consuming a more balanced diet, or controlling weight.

Tobacco use is related to complications such as placenta praevia, premature placental detachment, low fetal weight, and prematurity, even in passive smokers.^108^ Consumption of alcoholic beverages should be avoided, because it can cause fetal growth retardation, as well as abnormalities in the face and the central nervous system.^109^

Pregnancy in women with heart disease should be accompanied by a multidisciplinary team. Consultations with a cardiologist should be monthly during the first half of gestation, biweekly following week 21, and weekly until delivery, and they must respect constant interaction with an obstetrician, which ensures the best practice during the various stages of gestation.

These routines should, however, be adjusted according to case severity. Accordingly, pregnant women who permanently use anticoagulants are recommended to undergo weekly evaluation for clinical and laboratory control. Women with WHO class IV heart disease should be hospitalized during the third trimester of gestation for clinical stabilization and delivery planning. In addition, care should be recommended regarding diet, physical activity, sleep quality, and reduced stress and workload, depending on the patient’s profession and heart disease. Furthermore, review and adjustment of continuously used medications, as well as suspension or replacement of drugs that are harmful to the fetus, are practices that should take place before conception, i.e., during the pregnancy planning phase.

### 2.6.2. Physical Activity

During pregnancies without complications, the benefits of physical activity are unquestionable. They include improved physical resistance and cardiorespiratory function; reduced stress, anxiety, and risk of comorbidities related to sedentarism; and weight gain.^110,111^ Nevertheless, the American College of Obstetricians and Gynecologists contraindicates exercise during pregnancy in patients with heart disease with hemodynamic repercussions, patients classified as WHO risk III or IV, and in cases of preeclampsia, pregnancy-induced hypertension, severe anemia, and restrictive pulmonary disease.^112^

### 2.6.3. Diet

A balanced diet provides nutrients that are essential to fetal development, and it prevents complications related to weight loss in pregnant women. Obesity is associated with miscarriage, newborns with low birth weight, macrosomia, gestational diabetes, thromboembolism, and gestational hypertension, whereas malnutrition is linked to low birth weight and perinatal death.^113^ Notwithstanding an adequate diet, nutritional goals also require oral supplementation, as shown in Table 17. Consumption of foods rich in folic acid and supplementation with doses of 1 to 5 mg daily before conception and during the first trimester prevent neural tube defects in 72% of cases.^114^ Calcium supplementation (≥ 1 g daily) is associated with a significant decrease in the risk of preeclampsia (especially in women with low calcium consumption), as well as a decrease in prematurity and the occurrence of the composite outcome of “maternal death or severe morbidity.”^115^ The WHO recommends 1.5 to 2 g of calcium daily for pregnant women with low calcium consumption in their diets.

**Table 17:** – Dietary recommendations during gestation

Daily caloric ingestion – 340 to 450 additional kcal	2,200 to 2,900 kcal daily

Additional dietary supplements	
Folic acid	1 to 5 mg daily, preconception
Iron	27 mg > week 20
Calcium	250 to 1,000 mg daily
Folic acid	0.4 mg; 0.6 mg during the second and third trimesters
Iodine	150 mcg daily
Vitamin D	200 to 600 IU
Vitamins A, E, C, B and Zinc	Variable quantities, second and third trimesters

Fish consumption is the main source of non-occupational maternal exposure to methylmercury, which is found in all fish tissues and is absorbed in over 95%. Notwithstanding the risk of mercury poisoning, cohort studies have shown that greater maternal consumption of fish during the prenatal period was associated with better neurological development in newborns^116^ and that moderate consumption (up to 3 meals weekly) before week 22 of gestation was linked to reduced repeat prematurity. However, a recent systematic review and meta-analysis of randomized trials did not show statistical significance for the effect of long chain polyunsaturated fatty acids (n-3 PUFA) on reducing prematurity or any other fetal defects, such as neurological, cognitive, or visual acuity development.^117,118^

Regarding caffeine consumption, given the lack of adequate studies, it is recommended to limit consumption to less than 200 mg daily.^119^It is important to emphasize that coffee, which the main source of caffeine in many countries, contains 50% to 70% more caffeine than tea and other products. It is accepted that there is a theoretical relationship between caffeine and arrhythmogenesis, especially in women with heart disease.

Saline consumption, with no significant restrictions on salt, is generally recommended, especially close to delivery. However, pregnant women with risk of HF should be instructed to consume of 3 to 4 g of sodium chloride daily, without adding salt to food after cooking and avoiding salty items. Diets with 2 g of sodium daily should be restricted to more severe cases (NYHA FC III/IV), in addition to instructions regarding water restriction in these cases.

### 2.6.4. Professional Activity

Currently, most pregnant women work until one month before delivery, and only a small percentage suspend their professional activities earlier. The risk of developing complications is not related to work activity or to the psychosocial stress of work.^120^ However, demands and working conditions should be evaluated individually in women with limiting heart disease or obstetric situations, such as preeclampsia and IUGR. Changes or adaptations in work activities, reduced stress at work, or increased rest and relaxation periods are often beneficial measures, especially if the condition worsens or limiting symptoms appear.

Pregnant women with symptomatic heart disease or heart disease with hemodynamic repercussion should rest at home from the beginning of the third trimester of pregnancy, or be it, week 28 of gestation. During this phase, cardiac reserve limited by heart disease is insufficient to adapt to maximum hemodynamic changes, thus favoring the occurrence of HF, arrhythmias, IUGR, and prematurity.

Under pregnancy protection laws, all women are entitled to at least 6 leaves to perform examinations and consultations, proven by a medical certificate, for the time necessary to perform the procedures, as specified by the certificate.^121^

### 2.6.5. Key Points

- Prenatal care for women with heart disease is multidisciplinary, with regular consultations with a cardiologist. Frequency should be in accordance with disease severity and/or possible complications;
- Lifestyle orientations should be individualized in accordance with cardiac risk, as classified by the WHO;
- Pregnant women should be made aware of diet, weight control, physical activity restrictions, and controlling stress at work. Measures such as ceasing tobacco use, ceasing consumption of alcoholic beverages, and consuming caffeine moderately are essential during the prenatal period;
- Nutritional orientations include controlled calorie intake and a balanced and nutrient-rich diet, avoiding consumption of industrialized products and undercooked and poorly washed foods;
- Supplementation of essential vitamins and minerals should be a part of the prenatal routine for women with heart disease.

## 2.7. Management of Delivery and Postpartum Period

The interdisciplinary view of delivery and the postpartum period in women with heart disease should consider clinical and obstetric evolution during pregnancy and the functional status preceding delivery. Programming delivery in women with heart disease requires prior hemodynamic stabilization; screening for possible intercurrences such as infection, anemia, arterial hypertension, and arrhythmias; and adjustment of cardiovascular therapy.

From the obstetric point of view, the following are mandatory: discussion with a cardiologist and an anesthetist regarding timing and route of delivery, maternal-fetal monitoring during labor, and special attention to water balance. Accordingly, pregnant women with WHO class III/IV heart disease require assistance in tertiary hospitals with possibility of transfer to intensive care unit (ICU) during the postpartum period.

The labor team for women with heart disease should be prepared to prevent and treat the main complications during the intrapartum and postpartum period. The most frequent cardiac complications that stand out are HF, acute pulmonary edema, arrhythmias, thromboembolism, and dissection of the aorta, while preeclampsia, hemorrhage, and infections are included among obstetric complications.

### 2.7.1. Practice during Delivery

The general consensus is that route of delivery should be indicated by the obstetrician. However, in patients considered WHO risk I/II^122^with favorable clinical and hemodynamic conditions, spontaneous delivery at term of gestation is recommended. The consensus regarding type of delivery is based on the opinion of specialists, who believe that vaginal delivery is more advantageous, because it is associated with less blood loss, quicker recovery, and lower risk of thrombosis and infection. For this reason, it is the preferred type of delivery for women with heart disease whose clinical pictures are stable and uncomplicated.

Regarding cesarean delivery, its indication for patients with heart disease varies between 21% and 55%, worldwide.^123^ Available Brazilian data indicate that the rate of cesarean delivery in the general population is around 52%, and, according to the Brazilian Network for Surveillance of SevereMaternal Morbidity, the rate reached 76% among women with heart disease.^124^ There is no plausible clinical justification or explanation for such a high rate.

The rate of cesarean deliveries reflects the level of access to this intervention and its use; the task of defining the “desirable” rate in a given population, however, continues to pose a great challenge, as this number would adhere to medical indications, while avoiding “unnecessary” cesarean sections.

The European Registry of Pregnancy and Cardiac Disease has shown that the frequency of scheduled vaginal delivery was 69%; among cesarean deliveries, 44% had cardiac indication.^52^In its conclusions, the registry showed that, in terms of maternal results, programmed cesarean section showed no benefits over vaginal birth, and it was associated with worse fetal evolution.^125^

Maternal indications for cesarean delivery include very specific clinical situations, such as labor in patients under oral anticoagulation, diseases with increased aortic diameters (WHO risk III/IV), severe coarctation of the aorta, Takayasu arteritis, dissection of the aorta, PAH, acute HF, peripartum cardiomyopathy (PPCM) with severe HF, or other clinical situations in which maternal condition is critical.^52^

Although it is controversial, there are recommendations for assisted delivery, either by vacuum extraction or forceps, in situations where there are real maternal-fetal benefits to shortening the active phase of the second stage of labor and the efforts of a prolonged expulsive period. The recumbent left lateral position is recommended to avoid compression of the aorta and the inferior vena cava by the gravid uterus, thus favoring better maternal venous return and facilitating effort during the expulsive period.

Basic monitoring during delivery includes non-invasive blood pressure measurement, pulse oximetry, and continuous electrocardiography, in addition to fetal monitoring (auscultation of fetal heartbeats by Doppler sonar every 15 minutes during the first stage and every 5 minutes during the second stage, or continuous cardiotocography). The need for additional monitoring should be determined on a case-by-base basis. Excessive fluid infusion should be restricted in order to avoid excessive hydration and pulmonary congestion.

The benefits of analgesia for preventing arterial hypertension and tachycardia and reducing cardiac stress are unquestionable. A safe and effective way to reduce anxiety during this moment is with humanized delivery care, i.e., authorizing the presence of a companion chosen by the parturient and allowing her to ambulate freely and to choose the most comfortable position during labor.

Delivery in patients under oral anticoagulation should be scheduled from week 37 of gestation. Patients with high thrombotic risks need to use UFH around 36 hours before delivery, and the infusion should be interrupted 4 to 6 hours before birth and reintroduced 6 afterward, with APTT control. In cases with low thrombotic risks, LMWH is used until the day before delivery, and the night dose should be omitted if induced delivery or cesarean section is scheduled for the following morning. Regional block is possible in cases where 24 hours have elapsed since the last dose.

Induction of delivery should be considered at week 40 of gestation in all women with heart disease, because the benefits of this practice outweigh the eventual risks.^126^ Mode of induction mainly depends on evaluation of the uterine cervix and fetal vitality. Both misoprostol (PGE1)^127^ and dinoprostone (PGE2) are recommended for preparing the uterine cervix. The Krause method (balloon), amniotomy, and oxytocin infusion are also considered safe.^128^

In contrast, inhibition of premature labor should be considered with great caution and even contraindicated in women with heart disease. The degree of prematurity should be weighed with the risks of tocolysis and corticosteroid therapy, given that both may lead to complications, such as severe HF and cardiac arrhythmias.

When indicated, tocolysis should be maintained for 48 hours, which is sufficient time for the action of the corticoid, with the aim of reducing the occurrence of respiratory distress syndrome, peri- and intraventricular hemorrhage, and necrotizing enterocolitis in the newborn. The drugs used for inhibition, such as nifedipine, may induce hypotension, and they are synergistic when used in conjunction with magnesium sulfate. Terbutaline has intense beta-mimetic effect, and it may lead to maternal HF. In this situation, atosiban, a competitive oxytocin receptor antagonist, has been the safest tocolytic agent, when used in intravenous infusion of about 400 ml of solution (0.9% saline solution, Ringer lactate solution, or 5% glucose solution) for 48 hours (approximately 200 ml/24 h).

### 2.7.2. Practice during the Postpartum Period

Maternal care should be intensified during the postpartum period, and preventive measures for the main complications (HF, PPH, and thromboembolism) should be part of high-risk maternity hospitals’ protocols.

Maternal blood volume undergoes significant variations during the immediate postpartum period, due either to increased venous return following placental clearance or to estimated blood loss, which is as high as 500 ml in vaginal delivery and 1000 ml in cesarean delivery (as defined by the WHO and the Pan American Health Organization). The impact of these maternal hemodynamic oscillations explains the occurrence of severe complications, such as HF, acute pulmonary edema, and cardiogenic shock. Negligence regarding hemodynamic oscillations during the postpartum period is, in part, responsible for maternal mortality; for this reason, it is mandatory for patients with severe heart disease, even when they are stable, to remain in the ICU 24 to 48 hours after delivery for effective hemodynamic monitoring.

PPH is equally important; it occurs in approximately 10% of vaginal deliveries, and it is considered severe in approximately 3%. In women with heart disease, the incidence of PPH reaches 21%, and it is related to cesarean delivery, assisted delivery with forceps, general anesthesia, and use of heparin before delivery.^129^ The increase in maternal morbidity due to transfusion, infection, and thromboembolism is, in fact, the leading cause of death in women with heart disease.

For this reason, all maternity hospitals should have specific conduct protocols for preventing and treating PPH, including the use of uterotonic drugs, which are recommended during the third phase in both types of delivery, in order to prevent PPH.

Oxytocin is the recommended drug, given its benefit in preventing hemorrhage; it should be administered intramuscularly, at a dose of 10 IU for vaginal or cesarean delivery. Intravenous administration is also an option, especially during cesarean delivery, in doses of ≤ 5 IU and slow infusion (> 30 seconds) every 3 minutes, up to 3 infusions. Intravenous prophylaxis should be associated with continuous-infusion maintenance dose.

Misoprostol (600 to 1,000 µg) may be used safely for both prophylaxis and treatment of PPH, but oxytocin administered via bolus should be avoided due to the risk of hypotension. Ergometrine and methylergometrine should be avoided due to their association with coronary vasoconstriction and SAH.

It is accepted that there is a high risk of thrombosis during the postpartum period; therefore, measures such as early ambulation, which more feasible with vaginal delivery, and heparin anticoagulation should be recommended within the first 48 hours after delivery. Nevertheless, thromboembolism prevention should be individualized, and it will be discussed subsequently.

When there is an indication for definitive sterilization, bilateral salpingectomy may be performed via infraumbilical incision during the first 48 to 72 hours following vaginal birth. Generally speaking, discussions about contraception should take place before discharge from the maternity hospital.

### 2.7.3. Key Points

- Multidisciplinary assistance during delivery and the postpartum period should take the following into consideration: risk stratification of heart disease and elaboration of protocols for prevention and treatment of HF, PPH, infection, and thromboembolism;
- Assistance during delivery and the postpartum period should take place at a high-risk maternity hospital;
- Spontaneous vaginal delivery at term is recommended for most women with heart disease;
- Maternal indications for cesarean delivery are the following: severe HF, aortic disease with significant dilatation, severe obstructions of the left heart, severe forms of PH, and ventricular dysfunction;
- Cesarean delivery is indicated in patients who have gone into spontaneous labor while using oral anticoagulants (vitamin K antagonists) or who have suspended them for a period of less than 15 days;
- Delivery in patients under oral anticoagulation should be scheduled from week 37 of gestation, with adjustments to anticoagulation, using heparin as an intra-delivery “bridge”;
- Indications for preparing the cervix and inducing delivery are misoprostol (PGE1) and dinoprostone (PGE2);
- Indications for inhibiting labor are, initially, contraindicated. In exceptional cases, atosiban is the indicated tocolytic agent;
- It is mandatory for maternity hospitals to have a specific protocol for preventing and treating PPH;
- Women with severe heart disease should remain in the ICU for a 24- to 48-hour period following delivery;
- Contraception should be discussed before discharge from the maternity hospital;
- There should be awareness that the postpartum period is as important and risky as pregnancy;
- Breastfeeding should always be encouraged.

## 2.8. Obstetrical Anesthesia

Obstetric anesthesia plays a fundamental role in reducing maternal-fetal morbi-mortality,^130^ especially in pregnant women with heart disease. The complexity of heart disease requires the involvement of an anesthesiologist in multidisciplinary discussions during prenatal planning and the intrapartum and postpartum periods.

Frequency of evaluations should be individualized according to the risk of the heart disease and the patient’s clinical situation. In general, evaluation should take place trimesterly in patients in WHO risk class II and in 2- to 4-week intervals in patients in WHO classes III and IV.^131^Formal planning should be discussed between weeks 32 and 34 of gestation by the whole team,^132^so that the patient will be admitted to delivery with consolidated advice. This assists in the flow of attendance, and it reduces team stress during emergencies and the chance of negative outcomes.

The anesthesia team will have an opportunity to get to know the evolution of the gestation and its eventual complications, to adjust medications when selecting anesthesia and analgesia, and to make it possible for the patient to interact with greater clarity in understanding the conduct adopted during the intrapartum period. It is important not to neglect the risks of airway management and aspiration of gastric contents, adjustments of eventual medications, and administration of uterotonic drugs during the intrapartum period.

The current indication for pregnant women with heart disease is vaginal delivery with neuraxial analgesia. This type of anesthesia is more efficacious for controlling pain during labor analgesia than other techniques, such as the use of systemic opioids or inhaled nitrous oxide.^133,134^ When effective, spinal analgesia decreases circulating endogenous catecholamines, considering that partial sympathectomy induced by the effect of local anesthesia on the neuroaxis leads to decreased systemic vascular resistance and alterations in heart rate related to sympathetic block and cardiac reflexes.

During cesarean delivery, neuraxial analgesia has become prominent for managing pregnant women with heart disease, due to the growing expertise of anesthesiologists in using spinal block techniques that allow for better measurement of anesthesia in a gradual manner, thus decreasing hemodynamic impact.^135^ World literature demonstrates rates above 60% in elective cesarean deliveries performed with neuraxial block; this number is lower in emergency deliveries, in which case general anesthesia is chosen due to the complexity of factors to be evaluated for decision making.^136,137^

Among the neuraxial techniques most used for pregnant women with heart disease, both of the following stand out:

- Sequential epidural anesthesia;
- Sequential combined spinal-epidural anesthesia, with low dose in the spinal component.

These techniques are called “sequential,” because they allow the installation and cephalic progression of the sympathetic block to be performed gradually, preventing sudden installation with its cardiovascular repercussions. Small doses of local anesthesia associated with opioids are initially used, and additional supplements are performed through the epidural catheter until T6 sensory level is reached.^138^

In cases with contraindications to the use of neuraxial block for cesarean section, general anesthesia should be performed. The main objective of managing and planning with this anesthesia is to minimize the deleterious hemodynamic effects of systemic anesthetics and the hypertensive response to laryngoscopy, which is the reflex to sudden, exacerbated sympathetic stimulation. In this scenario, preanesthesia evaluation contributes to the identification of dural anomalies and severe scoliosis, which are common in Marfan, Loeys-Dietz, and Ehlers-Danlos syndromes.

Fast-acting drugs with short half-lives are used in doses adapted to maternal hemodynamics, in order to attenuate sympathetic responses to laryngoscopy, avoid large blood pressure variations, and prevent increased heart rate. Opioids such as alfentanil or remifentanil, as well as the use of other classes of drugs, may be useful for managing this. In adequate doses, short-acting beta-blockers and local anesthetics, such as esmolol and lidocaine, respectively, may be presented as good options. Additionally, in some circumstances, the use of inducers, such as ketamine and etomidate, may be a better option than propofol, which has higher cardiodepressive potential when used in bolus or in unadjusted doses.

When maintaining general anesthesia, it is necessary to pay attention to the potential negative inotropism of inhaled anesthetics and the decrease in systemic vascular resistance, which also occur with venous anesthetics. Dose-dependent uterine hypotonia, related to the use of inhaled anesthetics, may also occur, with greater potential for bleeding.^139,140^

In cases where general anesthesia has been utilized, a good plan for postoperative analgesia should be performed with the objective of reducing circulating catecholamines. In these cases, there are some options, including spinal options or epidural analgesia, abdominal wall block (transversus abdominis block and lumbar block), or use of systemic analgesia.

### 2.8.1. Fasting

For elective cesarean delivery, it is recommended to fast from solids for 6 to 8 hours, depending on the fat content ingested and on eventual anatomical or physiological alterations that cause longer delay in gastric emptying. Clear liquids may be consumed up to 2 hours before surgery. Pharmacological prophylaxis for aspiration of gastric contents is recommended, with non-particulate antacids, H_2_ receptor antagonists, and dopaminergic antagonists. During labor, women with low risks may consume moderate quantities of clear liquids,^141^such as water, tea, gelatin, and isotonic beverages.

In the event of maternal hemodynamic instability, the team should maintain the mother’s fast until it is safe to reintroduce liquids. Delivery analgesia and proximity to the second phase of labor are points that clearly exemplify this practice, given the possibility of hemodynamic instability and bleeding, respectively. In the most severe cases or when faced with a greater probability of cesarean delivery, the patient should continue fasting.

## 2.8.2. Anticoagulation and Neuraxial Block

It is estimated that spinal hematoma occurs in 1:200.000 to 1:250.000^142,143^deliveries. Although rare, it is a severe event; therefore, strategies should be taken to prevent it. Current recommendations consider that doses of anticoagulants and prior period of suspension are parameters for ensuring safe neuraxial anesthesia. This is the case for patients receiving a single anticoagulant, weighing more than 40 kg, with normal renal function, and without any other conditions that would contraindicate neuraxial block.^144^ In summary, the recommendations are hereafter explained.

### 2.8.3. Unfractionated Heparin (Subcutaneous)144

- Low dose (5,000 IU, 2 to 3 times daily): Wait 4 to 6 hours, with normal APTT or undetectable anti-Xa factor;
- Intermediate dose (7,500 to 10,000 IU, 2 times daily; up to 20,000 IU daily): Wait 12 hours or more, with normal APTT or undetectable anti-Xa factor;
- High dose (More than 10,000 IU per dose; more than 20,000 IU daily): Wait 24 hours or more, with normal APTT or undetectable anti-Xa factor.

### 2.8.4. Low Molecular Weight Heparin (Subcutaneous)144

- Prophylactic dose (enoxaparin 40 mg or deltaparin 5,000 IU, once daily): Wait 12 hours or more;
- Therapeutic dose (enoxaparin 1 mg/kg, twice daily or deltaparin 120 IU/kg, twice daily, or 200 IU/kg, single dose): Wait 24 hours.

Any anticoagulation regimes different from those mentioned above should be evaluated and individualized by the team, taking not only the risk of spinal hematoma into consideration, but also the thromboembolic risks, fasting time, maternal-fetal conditions, and the evaluation of predictors of difficult intubation for general anesthesia.

Time period for reinitiating anticoagulation should mandatorily include the participation of the anesthesiologist in cases where the approach is neuraxial. Reintroduction of anticoagulation should be individualized, because there are technical conditions for performing spinal or epidural anesthesia that interfere with the practice of reintroducing anticoagulation.

### 2.8.5. Hemodynamic Monitoring

The use of invasive monitoring in high-risk patients should be necessary, with the objective of reducing time between recognition of hemodynamic deterioration and their respective treatments. Before performing neuraxial block and inducing general anesthesia in patients in WHO-risks II/IV, invasive blood pressure (IBP) monitoring may be fundamental to a better outcome.^140^

Lack of validation of the use of non-invasive methods for monitoring cardiac output during delivery in women with heart disease makes it possible to individualize cases and indicate the utilization of invasive methods, such as central venous catheter (CVC) and pulmonary artery catheter (PAC). Nevertheless, the information obtained may be imprecise due to the complexity of the heart disease, in addition to the risk of inducing arrhythmias and other risks of complications. For this reason, there is low adherence to these forms of monitoring. In cases of cesarean delivery with general anesthesia, intermittent transthoracic echo and transesophageal echo have gained prominence as options for monitoring, and they may assist in the evaluation of ventricular function and filling.^140^

### 2.8.6. Intrapartum Uterotonic Drugs

The most used uterotonic drug during the peripartum period is oxytocin, which has an immediate effect on systemic vascular resistance, when administered in high doses or rapid transfusion. These regimes should be avoided for all pregnant women, especially in those with heart disease. The infusion of 2 IU of the drug for 10 minutes appears to be effective and not to have significant cardiovascular effects in pregnant women with heart disease.^135^ In general, it is possible to maintain an infusion of 2 to 5 IU for an interval of 15 to 30 minutes with low cardiovascular effects. Ergot derivatives induce smooth muscle contraction with vasoconstriction and consequent hypertension. Misoprostol may cause hyperthermia and tremors, and it results in increased oxygen consumption, which is harmful at times, especially in women with severe heart disease.

The rule that is most commonly used by anesthetists for oxytocin administration in patients without comorbidities is controversial, namely, the “rule of 3s,” consisting of 3 IU, every 3 minutes, up to 3 times, whereas an infusion of oxytocin at 2 IU for 10 minutes appears to be too slow.^139,142^

### 2.8.7. Postpartum

Follow up of pregnant women with intermediate to high risks should take place in the ICU for 24 to 48 hours. This observation period is important, keeping in mind that most deaths occur during the postpartum period. Inadequate monitoring and inappropriate blood volume management may lead to cardiovascular dysfunction.^140^

### 2.8.8. Key Points

- Anesthesia planning for delivery in women with heart disease should be discussed with a multidisciplinary team between weeks 32 and 34 of gestation;
- The indication for pregnant women with heart disease is vaginal delivery with neuraxial analgesia;
- In cases of cesarean delivery, neuraxial analgesia has gained prominence, when using spinal block techniques;
- General anesthesia is indicated in cases of severe heart disease;
- Anesthesia should be individualized in patients under anticoagulation;
- Maternal monitoring is indispensable during delivery and during the immediate postpartum period.

## 3. Assessment and Management of Specific Heart Diseases

### 3.1. Valvular heart disease

In Brazil, rheumatic disease is the most frequent cause of heart disease during pregnancy, with an estimated incidence of 50% in relation to other heart diseases.^145^ Rheumatic fever is an episode from early childhood and/or adolescence, the onset of the clinical phase coincides with fertile age in women.

Cardiovascular adaptation of heart valve diseases to the increase in cardiac output directly influences flow through the heart valves, with functional worsening of stenotic lesions. On the other hand, the drop in peripheral vascular resistance reduces the volume of regurgitation in insufficient valves. For these reasons, the evolution of stenotic lesions is generally worse, and it is correlated to the anatomical degree of the valve lesion, whereas, in patients with regurgitation, it is related to the condition of ventricular function.^146^

These initial considerations assist in risk stratification of valve disease, both for adequate family planning counseling and care during gestation. Accordingly, the classification elaborated by WHO was adapted for pregnancy in women with heart valve disease.

The WHO considers that patients classified as I and II present acceptable or low risks that do not impose serious restrictions to gestation, whereas risk III would make pregnancy inadvisable, and risk IV would contraindicate it.^147^ In this position paper, women with heart valve disease are classified in the following manner: risk I, acceptable; risks II and III, intermediate; and risk IV, high risk to pregnancy (Table 18).

**Table 18:** – Risk classification for heart valve disease during pregnancy.

High risk	Intermediate risk	Acceptable risk
Severe mitral stenosis	BPV with moderate dysfunction	Mild heart valve disease
Severe aortic stenosis	Severe pulmonary stenosis	BPV without dysfunction
Stenotic/calcified BPV		
MPV with dysfunction		
Heart valve disease + significant PH (PAP ≥ 50 mmHg)	MPV	Heart valve disease + LVEF normal
	Mitral MPV > risk of aortic MPV	
Aortic insufficiency + aortic disease	Aortic insufficiency + aortic disease	Heart valve disease without unfavorable factors
Marfan syndrome (AAD > 45 mm)	Marfan syndrome (AAD between 40 and 45 mm)	
Bicuspid aortic valve (AAD > 50 mm)	Bicuspid aortic valve (AAD 45 to 50 mm)	
Heart valve disease + LVEF < 35%	Patient requires use of anticoagulants	

*AAD: ascending aorta diameter; BPV: bioprosthetic valve; LVEF: left ventricular ejection fraction left ventricular ejection fraction; MPV: mechanical prosthetic valve; PAP: pulmonary artery pressure; PH: pulmonary hypertension. Severe mitral and aortic stenosis are considered mitral valve area ≤ 1.0 cm^2^ and aortic valve area < 1.0 cm^2^, respectively.*

It is worth adding that a high-risk situation in heart valve disease does not fulfill the criteria for indicating interruption of gestation (therapeutic abortion), given that these patients may be treated by either surgical or percutaneous intervention following the embryogenesis phase.

During family planning, evaluation of heart valve disease should establish etiological, anatomical, and functional diagnosis and investigate the presence of unfavorable factors that are part of the natural history of heart valve disease and previous surgical correction.^148^ These factors modify maternal prognosis, and they do not depend on structural cardiac injury per se; the following deserve special emphasis:

- AF;
- PH;
- Ventricular dysfunction;
- Associated aortic diseases;
- History of HF, thromboembolism, or infectious endocarditis (IE).

Cardiovascular evaluation before gestation should be assess the history, physical examination, and subsidiary tests that support in classification of risk of pregnancy, such as the following:

- ECG: evaluates rhythm and heart chamber overload;
- Echo: informs the type and severity of heart valve disease, degree of ventricular dilatation, presence of ventricular dysfunction, PH, and associated defects;
- CMR: useful when heart valve disease is associated with aortic disease;
- Ergometric test: valid for estimating functional capacity and arterial pressure in severe aortic stenosis in asymptomatic patients and when there is a dissociation between symptoms and the anatomical degree of mitral stenosis, indicated only during pregnancy planning;
- Biomarkers: a controversial application in heart valve disease.
- The recommendations put forth by Brazilian^149,150^ and International^151^ Guidelines for practice during family planning and pregnancy in cases of acquired, congenital, and prosthetic heart valve disease are shown in Tables 19 to 22.

**Table 19:** – Recommendations for clinical practice in acquired and congenital native valve disease.149,150

Heart valve disease	Preconception counseling	Gestation		

		Maternal risk	Fetal risk	Intervention
**Severe rheumatic** mitral stenosis	FC ≥ II **or** asymptomatic + PH > 50 mmHg **or** AF recent onset **consider** BCV or CPB	Increased risk **if** • HF • AF Death < 3%	Prematurity 20% to 30% IUGR 5% to 20% Stillbirth Increases with maternal FC III/IV	Beta-blocker diuretic Anticoagulation if AF **if** Refractory FC III/IV **consider** BCV or CPB
**Severe rheumatic aortic** stenosis congenital (bicuspid) degenerative	Symptomatic **or** asymptomatic + altered ET **or** EF < 50% **or** AVA < 0.7 cm^2^ average gradient > 60 mmHg **or** Bicuspid valve + AAD > 45 mm **consider** VCP or CPB	Increased risk HF - 10% Arrhythmia 3% to 25% Syncope Sudden death	Complications - 25% Prematurity IUGR Low birth weight Stillbirth	Rest diuretics with criterion **If** AF beta-blocker or CCB Anticoagulation Severe HF or syncope **consider** BCV or CPB
**Mitral insufficiency** Significant rheumatic degenerative valve prolapse	FC ≥ II **or** Complicated asymptomatic + EF ≤ 60% + SPAP ≥ 50 mmHg + LVSD ≥ 40 mm **consider** CPB (plasty or prosthesis)	HF AF Risk increases with EF < 35%	Low risk	Diuretic hydralazine digoxin **If** refractory HF **consider** CPB or “mitraclip”
**Significant rheumatic** aortic insufficiency congenital (bicuspid) degenerative	Symptomatic FC ≥ II **or** Unfavorable factors EF < 50% LVDD > 70 mm (75 if rheumatic) LVSD > 50 mm (55 if rheumatic) **consider CPB If** Bicuspid valve isolated AAD > 45 mm **consider** Proximal aortic intervention	Low risk Asymptomatic normal EF FC > II or EF < 35% HF and/or AF	Low risk	Diuretic hydralazine digoxin **If** refractory HF **consider If** Bicuspid valve AAD > 45 mm **consider** Proximal aortic intervention

*AAD: aorta diameter; AF: atrial fibrillation; AoS: aortic stenosis; AVA: aortic valve area; AVM: mitral valve area; BCV: balloon catheter valvuloplasty; CCB: calcium channel blocker; CPB: cardiac surgery with cardiopulmonary bypass; echo: echocardiography; EF: echocardiographic ejection fraction; ET: ergometric test; FC: functional class; HF: heart failure; IUGR: intrauterine growth restriction; LVDD: left ventricular diastolic diameter; LVSD: left ventricular systolic diameter; ME: mitral stenosis; NYHA: New York Heart Association; PH: pulmonary hypertension; SPAP: systolic pulmonary artery pressure. Severe mitral and aortic stenosis are considered MVA ≤ 1.0 cm^2^ and AVA < 1.0 cm^2^, respectively.*

**Table 20:** – Recommendations for clinical practice in congenital or acquired heart valve disease due to infectious endocarditis149,150

Heart valve disease	Preconception counseling intervention	Gestation		

		Maternal risk	Fetal risk	Intervention
**Structural TI** *Ebstein anomaly*	Severe symptomatic TI Significant RV dilatation/dysfunction **consider** conservative surgery (plasty) **or** BPV implant	Moderate/severe lesions Right HF Supraventricular arrhythmias	Low risk	Diuretic Digoxin **If** Severe right HF **consider** conservative surgery (plasty) BPV implant
Severe pulmonary stenosis	Effort dyspnea/fatigue Hypoxemia Atypical angina Right HF (secondary TI) BCV or CPB	Syncope Right HF Atrial arrhythmia Hypoxemia	Low risk	**If** Hypoxia/severe HF **Consider** BCV

*BCV: balloon catheter valvuloplasty; BPV bioprosthetic valve; CPB: cardiopulmonary bypass; HF: heart failure; IE: infectious endocarditis; TI: tricuspid insufficiency.*

**Table 21:** – Valve prosthesis with normal function and risks to gestation

Biological prosthesis with normal EF		Mechanical prosthesis with normal EF	

Maternal risk	Fetal risk	Maternal risk	Fetal results
Patient does not require anticoagulation Low risk	Low risk	**Patient requires anticoagulation** Intermediate risk	High risk Warfarin embryopathy Miscarriage Prematurity Stillbirth Perinatal Hemorrhage
		Anticoagulation favors Hemorrhage Systemic embolism **If** Prosthesis thrombosis **consider** Emergency treatment Thrombolysis or CPB	

*CPB: cardiopulmonary bypass; EF: ejection fraction.*

**Table 22:** – Clinical practice in prostheses with dysfunction during gestation149,150

Biological prosthesis		Mechanical prosthesis	

Maternal risk	Fetal risk	Maternal risk	Fetal risk
Dysfunction with predominance of insufficiency, FC I/II and normal EF Pharmacological measures	Low risk	Dysfunction with mild to moderate “paravalvular” insufficiency without significant hemolysis or severe HF **consider** Pharmacological measures for HF and anemia **If** Severe insufficiency or significant hemolysis **consider** Intervention If symptomatic HF and/or hemolysis **consider** Percutaneous paravalvular leak closure by means of a plug device **or** CPB (high risk of relapse)	High fetal risk if CPB
Dysfunction with predominance of valve stenosis with calcification (mitral, aortic, or tricuspid) Risks of severe HF, shock, sudden death **Always consider Emergency** Percutaneous implant **or** new transapical valve-in-valve* BPV **or** CPB	High fetal risk Fetal loss Prematurity Stillbirth	Aortic or mitral MPV stenosis due to intravalvular endothelial growth – “Pannus”: Need for intervention is rare If indicated, **consider** CPB MPV stenosis (generally aortic) “mismatch” Need for intervention is rare If indicated, **consider** CPB	High fetal risk if CPB

*BCV: balloon catheter valvuloplasty; BPV: bioprosthetic valve; CPB: cardiopulmonary bypass; HF: heart failure; MPV: mechanical prosthesis.*

#### 3.1.1. General Considerations for Treatment

Moderate restrictions on salt and physical activity, weight gain control (not exceeding 10 kg), and iron supplementation after week 20 of gestation are initial recommendations, taking care to rule out factors such as anemia, infection, hyperthyroidism, and cardiac arrhythmias. Prevention of rheumatic attacks should be maintained with 1,200,000 IU benzathine penicillin every 21 days or, for patients who are allergic to penicillin, 500 mg erythromycin stearate every 12 hours. Sulfadiazine is contraindicated. Prevention of IE for delivery is done with 2 g intravenous ampicillin associated with 1.5 mg/kg intramuscular gentamicin (with a maximum dose of 120 mg) 1 hour before delivery. The safety and efficacy of pharmacological treatment requires periodic dose adjustments.

Before conception, drugs with recognized teratogenic effects should be substituted. In women with mitral stenosis, the use of propranolol or metoprolol in doses that do not exceed 80 and 75 mg, respectively, stand out for preventing and controlling pulmonary congestion, always paying attention to perinatal side effects, such as hypoglycemia, hyperbilirubinemia, and polycythemia, which have not been verified in these recommended doses.

Acute AF should be promptly reversed by electric cardioversion in women with mitral heart valve disease, given that this procedure is considered harmless to the fetus, and it has the advantage of avoiding use of drugs at levels that are, at times, toxic. Additionally, atrial or ventricular ectopic beats and asymptomatic atrial tachycardia do not require the use of antiarrhythmic drugs. In order to control heart rate in patients with permanent AF, beta-blockers, or non-dihydropyridine calcium channel blocker (CCB) should be considered, in addition to anticoagulation.

The need for intervention in heart valve disease during gestation is due to cases that are refractory to clinical treatment. Percutaneous procedures should be preferable to cardiopulmonary bypass (CPB) surgery. In aortic stenosis, balloon catheter valvuloplasty (BCV) has been indicated for heart valve disease whose etiology is congenital or in attempts to save the mother’s life in extremely severe cases. In mitral stenosis, it requires the absence of thrombi in the left atrium, at most mild mitral insufficiency, and Wilkins echocardiographic score ≤ 8.^149^

#### 3.1.2. Key Points

- Stenotic valve lesion leads to more complications than regurgitation;
- NYHA FC I/II in stenotic lesions do not guarantee good maternal evolution;
- Complicating factors significantly increase the risk of heart valve disease;
- Percutaneous intervention should be considered before gestation in women with severe mitral and aortic stenosis, even in asymptomatic patients;
- Pregnancy does not change the criteria for indicating BCV;
- Pharmacological treatment of complications during gestation should be considered as the first therapeutic option;
- Prophylaxis of rheumatic disease should be maintained throughout gestation;
- Postpartum consultation, in addition to maternal clinical examination and evaluation of the baby’s health, includes medication adjustments, lactation stimulation, and contraceptive counseling.

#### 3.1.3. Valve Prosthesis

The prevalence of rheumatic disease in Brazil and the growing number of patients with congenital heart disease who require valve replacement have led to an increase in women with valve prostheses in childbearing age. One favorable factor, in this age range, is left ventricular performance, which is generally preserved.

From the hemodynamic point of view, valve prostheses improve functional capacity and promote clinical evolution during pregnancy. Biological prostheses have attributes that are favorable to evolution of pregnancy, as they do not require anticoagulation, and they are considered WHO-risk II. Nevertheless, they have limited durability, with the possibility of short-term reoperation, including during pregnancy.

Bioprosthetic valve (BPV) dysfunction due to calcification has poor evolution, and it leads to pulmonary congestion and low cardiac output, both of which are refractory to clinical treatment, in addition to causing a high risk of sudden death (WHO-risk IV). The occurrence of BPV calcification during pregnancy makes surgical indication for valve replacement mandatory, regardless of gestational age.^152^

In contrast, gestation in women with mechanical valve prostheses (MPV) is considered WHO risk III. The risk of thrombosis due to maternal hypercoagulability and difficulties with long-term anticoagulation are associated with a variable incidence of embolic accidents, spontaneous abortion, warfarin embryopathy, and maternal and neonatal hemorrhagic phenomena.^96^

It is estimated that the probability of pregnancy to be maternal-fetal free of events is 58.3% for BPV and 46.9% for MPV^96,153^ and no differences in mortality rates for both prosthesis types, were observed . Although it is controversial, BPV may be considered the more adequate form of replacement for women of fertile age, except in adolescent patients, where premature calcification of BPV would favor the choice of MPV.

The following factors are related to prognosis of pregnancy:

- Prosthesis functional status;
- Cardiac rhythm (AF);
- Ventricular dysfunction;
- NYHA FC;
- Prior incidence of IE, HF, or thromboembolism.

#### 3.1.4. Maternal Risk

Women with MPV present an estimated risk of 5% for valve thrombosis during gestation; maternal mortality varies between 9% and 20% associated with thromboembolic complications.^96^This incidence of thrombosis in MPV varies according to the anticoagulation regime, but it is signicantly higher during heparin use^154-157^The incidence of thromboembolism with LMWH is due to fluctuations in anti-Xa factor that occur over 24 hours,^158^ even with the therapeutic value (0.6 to 1.2 IU/ml) during peak action 4 hours after application,^159^ resulting in a suboptimal anticoagulation level. Regarding UFH, prolonged use is associated with thrombocytopenia and osteoporosis;^154,155^its efficacy is inferior to that of LMWH, and its subcutaneous use for anticoagulation practice has been prohibited. Owing to the high incidence of thromboembolism with heparins (UFH and LMWH), there is a tendency to prioritize the use of warfarin throughout the entire pregnancy, as it is believed to be safer for maternal-fetal outcomes.^154-157^

#### 3.1.5. Fetal Risks

In all anticoagulation regimes, the obstetric risks of hemorrhage, placental abruption, prematurity and fetal death are very high.^155-157^The warfarin cross the placental barrier, it is teratogenic when used in the first trimester of pregnancy and causes embryopathy in 0,6 a 10% of cases,^160^ even at doses less than 5 mg.^161-163^

The anticoagulation regime for women with MPV who wish to become pregnant or who are in the course of gestation during the first consultation continues to be controversial. Factors that should be taken into consideration when deciding on the best anticoagulation treatment include the patients’ preferences, the attending physician’s expertise, and availability of adequate coagulation control.

Recommendations for preventing thromboembolism in mechanical prostheses intend to meet to the ideal requirements of a position based on documentation in the literature and on the authors’ experience, in a manner that is effective for the reality which different healthcare services face. It is understood that permanent anticoagulation should be divided into five different stages, including preconception, each trimester, and the postpartum period, as explained hereafter.

**First Stage: Preconception. Patient/couple awareness. Advice regarding early diagnosis of pregnancy*.*** Patients in pregnancy planning should receive clarification regarding the need to maintain anticoagulation, the regimes available, and their risks during all phases of gestation, childbirth, and the postpartum period. In order to achieve this, frank dialogue with the couple is fundamental. Counseling also includes information regarding the importance of early diagnosis of pregnancy in order to reduce the occurrence of embryopathy. During this consultation, the patient receives an examination request for chorionic gonadotropin beta dosage, which should be taken at the first sign of delayed menstruation.

**Second Stage: First trimester. Anticoagulant substitution (avoiding teratogenesis).** Substituting warfarin with heparin makes it possible to reconcile the benefits of preventing maternal thrombosis and the harmful effects of embryopathy. During this period, there are different options, which are shown in Figure 6. The first choice is to use LMWH, which requires weekly anti-Xa factor control. If this option is not available, intravenous UFH is indicated between the sixth and ninth week of gestation. In patients whose first medical consultation takes place after week 6 of gestational, warfarin should not be suspended. In these cases, the couple should be informed regarding the possibility of embryopathy and that the risks of substituting with heparin are no longer justified.

**Figure 6:** – *Recommendations for anticoagulation in patients with mechanical valve prostheses during gestation. CGH: chorionic gonadotropin hormone; INR: international normalized ratios; LMWH SC: subcutaneous low molecular weight heparin; UFH IV: intravenous unfractionated heparin; VKA: vitamin K antagonists. LMWH SC every 12 hours = 1 mg/kg/dose; UFH IV = 18 IU/kg/h. Dose targets and controls: LMWH SC = anti-Xa factor between 0.6 and 1.2 U/ml, weekly; UFH IV APTT: 2 times normal value, daily; VKA = INR 2.5 to 3.5, biweekly.*

**Third Period: Second and third trimesters. Resuming oral anticoagulant, and anticoagulation control.** Resuming warfarin use is justified by the assumption that shortening the use of heparin reduces adverse effects to the mother and leads to a lower risk of embryopathy. The proposal is to maintain warfarin doses in accordance with pre-gestation goals, with weekly or biweekly INR control. Reintroduction of warfarin should follow the dynamics of transition, or be it, simultaneous with subcutaneous LMWH or intravenous UFH until INR target value has been reached (Figure 6).

**Fourth Stage: Delivery planning. Consider hospitalization, redirect to parenteral anticoagulation, control anticoagulation and plan delivery.** Hospitalization should be scheduled at week 36 of gestation for use of subcutaneous LMWH or intravenous UFH in therapeutic doses (Table 23). Route of delivery must be discussed with the obstetrician; vaginal delivery is considered safer due to the fact that there is less bleeding and to the advantages of analgesic techniques. In cases of premature delivery under anticoagulation, route of delivery is cesarean, and the use of prothrombin complex concentrate may be considered.

**Table 23:** – Recommendations for anticoagulation dose and control in patients with mechanical prostheses during pregnancy

Gestational age( week)	Anticoagulant	Control
Between 6^th^ and 9^th^	LMWH 1.0 mg/kg SC every 12 hours UFH IV 18 IU/kg/h infusion pump (< 30,000 IU IV)	AntiXa: 0.6 to 1.2 U/ml APTT 1.5 to 2.0 times/NV
12^th^ to 36^th^	Warfarin according to INR	Aortic INR between 2.5 and 3.0 Mitral INR between 3.0 and 3.5
After week 36^th^ until delivery	LMWH 1.0 mg/kg SC every 12 hours UFH IV 18 IU/kg/h infusion pump (< 30,000 IU IV)	AntiXa: 0.6 to 1.2 U/ml APTT 1.5 to 2.0 times/NV
The postpartum period	LMWH 1.0 mg/kg SC every 12 hours UFH IV 18 IU/kg/h infusion pump (< 30,000 IU IV) Warfarin reaching target INR for hospital discharge	AntiXa: 0.6 to 1.2 U/ml APTT 1.5 to 2.0 times/NV INR between 2.0 and 2.5

*APTT: activated partial thromboplastin time; INR: international normalized ratio; IU: international units; IV: intravenous; LMWH: low molecular weight heparin; NV: normal value; SC: subcutaneous; UFH: unfractionated heparin.*

**Fifth Stage: The postpartum period. Reintroduction of oral anticoagulation and hospital discharge*.*** Six hours after delivery, in the absence of maternal complications, intravenous UFH or subcutaneous LMWH may be reintroduced in therapeutic doses. Warfarin should be prescribed 48 hours after delivery, following the dynamic of transition, in conjunction with heparin, until INR value reaches 2.0, at which point the patient is discharged from the hospital.

#### 3.1.6. Key Points

- BPV do not require anticoagulation, except in patients with AF or previous thromboembolic accident;
- Pregnancy does not influence structural degeneration of BPV;
- Calcified, stenotic BPV are indicated for surgery regardless of gestational age;
- MPV require anticoagulation with permanent adjustments seeking to meet conventional goals;
- MPV thrombosis requires immediate intervention with a thrombolytic agent or emergency surgery with CPB, regardless of gestational age;
- Choice of BPV as a preferable substitute for a woman who plans pregnancy, considering that it does not requires anticoagulation and the future perspective of percutaneous valve-in-valve replacement;
- Percutaneous valve-in-valve procedures require a specialized center with a heart team and resources for valve and arterial tomography, 3-dimensional esophageal echo, and an interventional hemodynamic and surgery team on standby;
- Patients with MPV should be referred to tertiary services and reference centers in valve disease for follow up during pregnancy;
- Permanent anticoagulation in patients with mechanical prostheses or mitral valve disease with AF should follow the algorithm which divides the pregnancy and postpartum into five stages;
- Notwithstanding adequate and effective anticoagulation control at all times, there are still uncertainties regarding the success of pregnancy in women with MPV;
- A multidisciplinary team should discuss choice of valve prosthesis and prospects for future pregnancy together with the patient.

## 3.2. Congenital Heart Disease

Advances in clinical and surgical cardiology treatments have demonstrated that a progressively higher number of women with congenital heart disease are able to reach childbearing age^164^ then they wish to become pregnant with a great likelihood of successful maternal-fetal outcome.^165^

In Brazil, a growing tendency has been observed in the percent of pregnant women with congenital heart disease, similar to European countries. They are considered the second-leading indirect cause of maternal mortality, accounting for up to 20% of deaths due to heart disease.^166^

Preconception assessment risk should be based on following variables: (1) time of heart disease diagnosis; (2) prior palliative or corrective surgery; (3) NYHA functional class; (4) laboratorial tests such as: hematocrit, hemoglobin, oxygen saturation, natriuretic peptide values; and liver and thyroid function tests.

Structural and functional diagnosis is defined by electrocardiography, transthoracic echo, magnetic resonance and cardiopulmonary testing.

The WHO classification has been very well accepted as a parameter for evaluating maternal-fetal risk according to structural cardiac injury. In addition to this classification, there are clinical conditions that are predicted over the natural history of congenital heart disease (which modify the prognosis of pregnancy and are independent of structural cardiac injury), which are shown in Table 24.

**Table 24:** – Factors associated with maternal prognosis in congenital heart disease

Pulmonary arterial hypertension
Cyanosis
Severe obstructive lesions
Ventricular dysfunction
Permanent anticoagulation requirement
Symptomatic patients indicated for intervention in heart disease

**Eisenmenger syndrome:** It is considered to have a high risk for maternal death, which reaches 50% during the pregnancy e postpartum.^167^ Pulmonary arterial disease restricts circulatory adaptation to the variations in cardiac output and to the drop in peripheral vascular resistance during pregnancy and postpartum period. This leads to the main complications that cause death, such as HF, hypoxia crises, and arrhythmias. The risk during the postpartum period is as high as during gestation, due to hemorrhage and thromboembolism.^168^ Patients with Eisenmenger syndrome appear to be predisposed to thrombocytopenia, deficiency in vitamin K-dependent coagulation factors, and bleeding. The fetal risks of spontaneous abortion, prematurity, and perinatal mortality are proportional to the degree of cyanosis.

**Cyanosis:** Almost 30% of patients with cyanotic congenital heart disease, whether or not they undergo previous surgery, present complications during gestation, such as: HF, systemic-pulmonary thrombosis, arrhythmias, and hypoxemia, are presumptive. The degree of arterial oxygen saturation is a prognostic factor for maternal-fetal survival, and the hypoxia has a significant correlation with maternal death, spontaneous abortion, and perinatal death.^169^ The indication of phlebotomy in maternal erythrocytosis is only been performed with hematocrit above 65% in patients with symptoms of headache, fatigue, visual or cognitive impairment, and myalgia. Fetal outcome, including miscarriage, prematurity, and perinatal death, is related to the degree of arterial oxygen saturation. It is estimated that oxygen saturation < 85% is associated with only 12% live newborns.^170^

**HF:** dyspnea is a clinical parameter used to aid practice and estimate prognosis of congenital heart disease, however, have limitations when applied to pregnancy. Dyspnea may be consequence of hypoxemia or pulmonary congestion, which is related to congenital heart disease involving the left heart.^52^

**Cardiac arrhythmias:** Frequent in adults with congenital heart disease, arrhythmias are the result of sequelae of cardiac defects such as ventricular dysfunction, myocardial hypertrophy, fibrosis or surgical injure, conduction tissue trauma, and the presence of endocardial grafts.

**Previous intervention of pregnancy:** Surgical or percutaneous correction of congenital heart disease is associated with better maternal-fetal prognoses in comparison with patients who have not undergone operation for heart disease. The eventual need for intervention should be taken into account before conception.

### 3.2.1. Pregnancy Management

Giving continuity to preconception evaluation, initial prenatal visit should include (1) history; (2) type of corrective or palliative surgery; (3) immediate or late postoperative evolution; (4) current clinical and functional situation; and (5) periodic laboratory examinations (hematocrit, hemoglobin, oxygen saturation, and natriuretic peptide).^171^

Attending during gestation, delivery, and the postpartum period for patients with congenital heart disease should rely on a team of specialists, a tertiary hospital, and periodic attendance. It is worthwhile to recall that the hereditary nature of congenital heart disease makes routine fetal echo necessary from the second trimester of gestation on.^171^

Pregnant women classified as WHO risks III/IV should receive advice regarding routine hospitalization starting between 28 and 32 weeks of gestation, for compensation of maternal condition, continuous fetal monitoring, therapy adjustment, and delivery planning. Decisions regarding the management of delivery and anesthesia should be made jointly, in accordance with the mother’s clinical situation and fetal vitality and maturity.

**Congenital heart diseases associated with PH:** It is recommended the interruption of pregnancy in women with PH and Eisenmenger syndrome during the first trimester of gestation. Nonetheless, when the patient decides to continue with the pregnancy, the multidisciplinary team should follow the protocols^172^ that include hospitalization after week 28 of gestation, enoxaparin (LMWH) use in a prophylactic dose (1 mg/kg daily), and oxygen therapy (supplemental oxygen for saturation below 92%) are essential measures for controlling hypotension, hypoxemia, and metabolic acidosis.

Specific vasodilators, such as phosphodiesterase inhibitors (sildenafil), may lead to arterial hypotension, and they should be indicated individually in accordance with clinical situation and maternal tolerance.^173,174^ Sildenafil or other phosphodiesterase inhibitors have been used, as well as the eventual addition of prostaglandins when symptoms persist. Endothelin receptor antagonists should be suspended during pregnancy.^175,176^

Full-dose or prophylactic LMWH should be considered as substitute of warfarin during the first trimester and after 36^th^ week of gestation for patients whose already using it before conception (Figure 6). The antiplatelet agents (such as aspirin) or LMWH should be prescribed with great caution, because patients with PH present a high risk of hemoptysis and thrombocytopenia.

**Congenital heart diseases with obstructive structural lesions:** Patients with severe left ventricular outflow tract obstructions should be advised to surgical or percutaneous correction previous of gestation. If the patient is, however, already pregnant, the triad of symptoms (HF, angina pectoris, and syncope) percutaneous or surgical intervention should be considered, even during gestation.^171^ In patients with severe valve pulmonary stenosis whose present heart failure, the percutaneous balloon valvuloplasty is indicated, and it is safest during the second trimester of pregnancy, when the embryogenesis phase has passed; the fetal thyroid is still inactive, and the uterus still has a small volume, allowing for greater distance between the ionizing radiation and the conceptus during the procedure.

**Cyanotic heart diseases without pulmonary hypertension:** General measures include restricting physical activity, supplementing oxygen, and preventing venous stasis due to the known risk of paradoxical embolism. The use of LMWH in prophylactic doses is recommended, because thromboembolism is one of the main complications. Iron supplementation may be used, depending on polycythemia, similarly to Eisenmenger syndrome.^175,176^

**Heart disease with shunt without pulmonary hypertension:** Atrial septal defect (ASD) is well tolerated during pregnancy and is considered WHO- risk I.^177^ Arrhythmias, which are generally supraventricular, are common and they may be controlled with digoxin, beta-blockers (propranolol or metoprolol), or CCB (verapamil) in fractionated low doses. Patients with uncorrected ASD are considered to present a risk of thromboembolism, which may suggest that LMWH should be used. Although it is not routine, symptomatic patients with left-right flow and hemodynamic instability may benefit from percutaneous closure of this defect.

Patients with small or operated interventricular communication (VSD) tolerate pregnancy well and are considered WHO-risk I, especially when ventricular function is normal.

The evolution of atrioventricular septal or canal defects that have not been corrected depends on the magnitude of valve regurgitation and the size of communication between chambers. It is considered WHO- risk I. The most frequent complications are arrhythmias, pulmonary congestion, and HF in patients with ventricular dysfunction. Treatment includes use of digoxin, diuretics (furosemide), vasodilators (hydralazine), or beta-blockers (carvedilol).

**Coarctation of the aorta:** Pregnancy is tolerated in patients with corrected coarctation of the aorta, which is considered WHO-risk II.^178,179^Nonetheless, in when it has not been corrected prior to conception, there are associated complications which lead to high risks to pregnancy, such as arterial hypertension with the additional risk of preeclampsia, aortic aneurysm, dissection of the aorta, and rupture of cerebral aneurysm, which goes on to become WHO-risk IV. It is fundamental to control arterial pressure, using conventional therapy.

**Tetralogy of Fallot:** Tetralogy of Fallot is the most common cyanotic heart disease in adults, and patients whose have undergone corrected surgery they tolerate pregnancy very well. In this group, risk factors are right ventricular dysfunction and pulmonary insufficiency, which adequately adapt to pregnancy in most cases.^179^ The current practice of replacing the pulmonary valve in the population of patients with significant right ventricular dilatation has contributed to an increasing contingent of pregnant women with pulmonary biological prostheses. Cardiac arrhythmias are common events during the late postoperative period but do not compromise obstetric and fetal outcomes.^180^ Experience with unoperated tetralogy of Fallot is very limited, and it should follow the recommendations for cyanotic heart diseases.

**Ebstein anomaly:** Prognosis for pregnant women with Ebstein anomaly is related to the presence or absence of cyanosis and HF. Hemodynamic instability is associated with tricuspid insufficiency or right ventricular dysfunction. Pregnancy should be planned following surgical correction in symptomatic patients with HF or cyanosis. Pre-excitation syndrome is commonly associated with the anomaly, and arrhythmias may be a complicating factor during gestation, even in patients who have undergone operation.^181^

**Transposition of the great arteries (TGA):** In cases of dextro-TGA, late evolution following atrial (Senning or Mustard procedure) or arterial (Jatene surgery) switch has been positive, and pregnancy is well tolerated.^182^ The presence of right ventricular dysfunction or significant tricuspid insufficiency is an important factor for poor prognosis and restricting pregnancy.^175^Practice for treating complications should follow conventional recommendations. In cases with levo-TGA, also known as ventriculoarterial and atrioventricular discordance or ventricular inversion, evolution of pregnancy depends on FC, systemic right ventricular function, arrhythmias, and associated lesions.^183^ In adults, the main concern is ventricular dysfunction.^184^ For these young women, pregnancy should be advised against.

**Fontan procedure:** Successful gestations have been reported in patients who have undergone Fontan surgery, although there is a risk if Fontan circulation is not adequate, at which point the consequent complications of low cardiac output, arrhythmia, or hepatic disease arise;^185^ these cases are considered WHO risk III. Pregnancy is not advised for patients whose arterial oxygen saturation is lower than 85% or patients with severe atrioventricular insufficiency, depressed ventricular function, or enteric protein loss; these cases are considered WHO-risk IV. Practice is to treat and prevent HF, arrhythmias, and thromboembolism. Obstetric and fetal evolution in patients who have undergone the Fontan procedure is uncertain and complicated due to the high incidence of spontaneous abortion, prematurity, small for gestational age newborns, and neonatal death. There is also a high risk of PPH, which is peculiar to this clinical situation.^185,186^

**Heredity:** Children of mothers with congenital heart disease have a higher risk of presenting congenital cardiac lesions, which vary according to the type of maternal defect and which are not necessarily the same as the maternal structural lesion. Fetal echo is used to detect the recurrence of congenital heart disease, which is around 2.7% to 10% of cases.^187^ It has been verified that there are genetic syndromes associated which specific defects, such as IAC in Holt-Oram syndrome, conotruncal anomaly in DiGeorge syndrome, among others, which are transmissible. These data reinforce the recommendation for fetal echo as part of the prenatal routine for the group of women with hereditary congenital heart disease.

### 3.2.2. Key Points

- Pregnancy planning requires determination of structural and functional diagnosis of heart disease based on laboratory and imaging examinations;
- Preconception counseling should be based on WHO risk classification;
- The presence of PH, cyanosis, arrhythmias, ventricular dysfunction, previous thromboembolic events, or HF adds risks to the WHO categories;
- When indicated, surgical or percutaneous intervention should be performed before conception;
- Pregnant women with WHO risk III/IV should be referred for specialized care in tertiary centers with the support of a Pregnancy Heart Team;
- Heredity of congenital heart disease requires the performance of fetal echo as well as genetic and preconception counseling.

## 3.3. Cardiomyopathies

Cardiomyopathies are cardiac muscle diseases that structurally and functionally compromise the heart in the absence of coronary artery disease, arterial hypertension, or valvular or congenital heart disease, which would justify the observed myocardial abnormality. According to phenotype, cardiomyopathies are classified as hypertrophic, dilated, restrictive, arrhythmogenic right ventricular, and non-compaction.^188^This classification is fundamental for risk assessment and estimating prognosis of gestation, regardless of probable etiology. PPCM will be discussed subsequently.

A retrospective study on cardiomyopathies during gestation has shown a 35% incidence of complications with 11% maternal mortality, data which are related to the form and the degree of myocardial impairment.^189,190^ WHO risks III/IV includes cardiomyopathies with reduced left ventricular ejection fraction (LVEF) below 30%, with manifestations of HF, PH, and complex arrhythmias.^52^

HF is the main complication, especially after the second trimester of gestation and during labor. During the immediate postpartum period, which is as sensitive as gestation, the following recommendations should be followed: caution with the use of oxytocic drugs; moderation when infusing fluids during the intrapartum period; attention to PPH, pain control, and infection prevention; and transfer to the ICU within the first 24 to 48 hours after delivery.

### 3.3.1. Dilated Cardiomyopathy

Approximately 50% of cases of dilated cardiomyopathy are idiopathic, and 20% to 35% are hereditary; genetic mutations have been identified in almost 40%.^191^ The following acquired causes stand out: viral myocarditis (coxsackievirus, parvovirus, echovirus, adenovirus), H1N1, Epstein-Barr virus, and other causes related to drug use.

During family planning, when the patient intends to become pregnant, the following are recommended: (1) adjustments to maternal therapy regarding HF control, considering that essential drugs which are contraindicated during pregnancy (ACEI, ARB, neprilysin inhibitors, spirolactone, ivabradine) should be substituted; (2) patient awareness of the possible immediate and long-term impact of pregnancy on heart disease; (3) genetic counseling, given that the disease is associated with autosomal dominant inheritance, followed by autosomal recessive inheritance, and to X-chromosome linked diseases.^191^

### 3.3.2. Hypertrophic Cardiomyopathy

Global prevalence of hypertrophic cardiomyopathy (HCM) is around 0.02% to 0.2% of the population,^192^ and it was estimated at 0.015% in a cohort study of pregnant women with heart disease.^145^ Pregnancy constitutes a potential risk for women with HCM; its prognosis, however, is still uncertain.

The great variation in the rate of cardiovascular complications during pregnancy, estimated between 5% and 40%, has been attributed to the heterogeneity of the phenotype of this heart disease.^193-195^Although they are often asymptomatic, the most frequent complaints of pregnant women with HCM are chest pain, dyspnea, syncope, and palpitation. The factors associated with worse prognosis for pregnancy are history of HF, ventricular arrhythmia, and sudden death in the family. Complications during pregnancy result from left ventricular outflow tract obstruction, diastolic dysfunction, and myocardial ischemia.

Among the most frequent arrhythmias, the following stand out: atrial extrasystoles, sustained supraventricular tachyarrhythmias, and AF, which favor maternal hemodynamic instability. From the obstetric point of view, the most frequent complications are spontaneous abortion in approximately 20% of cases and low birth weight in 10%.^193-195^

Another important issue is the risk of transmitting the disease to the fetus, because HCM is an autosomal dominant Mendelian trait, which is also caused by mutations that encode the components of the cardiac sarcomere.^196^ The complexity of this disease still does not allow for determination of its true incidence in apparently healthy newborns who do not present abnormalities on 2-dimensional echo. In most cases, echo during the neonatal period does not identify HCM because myocardial hypertrophy occurs over the course of development, only becoming apparent after adolescence. Nonetheless, it is worth highlighting that the disease’s obstructive form and family history of sudden cardiac death (SCD) are risk factors for early manifestation of hypertrophy in children.^131,197^

Genetic study of asymptomatic children and adolescents with family history of HCM may identify “healthy” carriers of the mutation. There are, however, important obstacles to the clinical application of this investigation, such as genetic plurality, low frequency of the mutation responsible in the diseased population, difficulties in techniques for identifying the pathogenic mutation, and high costs.

In symptomatic patients, initial pharmacological treatment is with the use beta-blockers, propranolol, or metoprolol succinate, which may or may not be associated with CCB, such as verapamil.^52^Association of these drugs requires caution with respect to maternal tolerance, arterial pressure, and fetal vitality. The use of prostaglandins to induce delivery is not advisable, due to their vasodilating effects. Vaginal delivery is considered to be safe, whereas cesarean delivery is reserved for special situations. Epidural or spinal anesthesia should be contraindicated in severe obstructive forms.

During pregnancy planning in patients with arrhythmias that are difficult to control pharmacologically, it is necessary to consider discussing the possibility of percutaneous intervention with an electrophysiologist. Examples include radiofrequency ablation in cases of complex and/or symptomatic tachycardias or even ICD in patients included in conventional class IA recommendations.

### 3.3.3. Arrhythmogenic Right Ventricular Dysplasia

Arrhythmogenic right ventricular dysplasia is a hereditary, autosomal dominant cardiomyopathy, with reduced penetrance and variable expressivity. For these reasons, genetic counseling is mandatory.

Pregnancy is well tolerated in women with this disease, but patients with preexisting biventricular disease have a higher risk of developing HF as pregnancy progresses.^198^ Symptom control and prevention are done with beta-blockers (propranolol, metoprolol succinate). In the event that they are necessary, antiarrhythmic drugs should be maintained, respecting fetal toxicity limits. If indicated, ICD implantation should preferably take place before gestation.^199^

### 3.3.4. Non-compaction Cardiomyopathy

The non-compacted myocardium is characterized by distinctly trabeculated myocardial morphology. It is a family disease in up to 60% of cases, with autosomal dominant inheritance. Its prevalence is unknown, and evidence regarding practice during pregnancy is limited.^200^ The clinical picture is highly variable, ranging from asymptomatic patients to patients with refractory HF and severe arrhythmias. There is no specific treatment for non-compaction cardiomyopathy, and therapeutic conduct should be supported by experience with other cardiomyopathies. The risk of thromboembolism, however, is considered to be greater due to the myocardial morphology in itself, which justifies permanent anticoagulation during gestation.

### 3.3.5. Restrictive Cardiomyopathy

Idiopathic restrictive cardiomyopathy is characterized by non-hypertrophic, non-dilated ventricles, with diastolic dysfunction, resulting in atrial dilatation. It may be idiopathic or associated with other diseases, such as amyloidosis, endomyocardial fibrosis, sarcoidosis, and hemochromatosis. Scarcity of experience in the literature, limited and controversial therapy, and frequently severe clinical evolution are factors which make pregnancy unadvisable.

### 3.3.6. Key Points

- Women with cardiomyopathy should participate in family planning, including genetic counseling;
- Risk stratification for subsequent pregnancies should consider the functional and structural status of the cardiomyopathy;
- Children of women with HCM, even when they are apparently healthy, should receive differentiated follow up until adolescence;
- Therapeutic optimization should follow conventional guidelines, considering classical drug contraindications during gestation;
- Permanent anticoagulation should be practiced in pregnant women with noncompaction or dilated cardiomyopathy, intracavitary thrombus, or prior embolic event;
- Genetic studies are promising for changing prognosis in cardiomyopathies.

### 3.3.7. Peripartum Cardiomyopathy

PMFC is defined as an idiopathic form of cardiomyopathy that manifests with HF secondary to left ventricular systolic dysfunction, with LVEF (< 45%), which occurs in late pregnancy or months after delivery or miscarriage, when none other cause of HF has been found.^201^

The pathophysiology of PPCC, not yet fully understood, is based on hypotheses that suggest hormonal, inflammatory, autoimmune, infectious, genetic and environmental mechanisms.^201^ New concepts on etiopathogenesis have been presented, involving oxidative stress, angiogenic imbalance, and prolactin in the genesis of PPCM.^202,203^

The most recent studies show that PPCM is triggered by increased oxidative stress in pregnancy,^204^ in combination with lower expression of angiogenesis regulators. Oxidative cleavage of prolactin by cathepsin D, the major endoprotease responsible for the generation of adenohypophyseal vasoinibins, generates an antiangiogenic subfragment, prolactin 16kDa, with apoptotic and proinflammatory properties. The 16kDa prolactin in endothelial cells suppresses vasodilation depends on nitric oxide (ON) and angiogenesis. In addition, the endothelial cell secretes microparticle exosomes (microRNAs), specifically miRNA (miRNA), which, when absorbed by cardiomyocytes, interferes with their cellular metabolism and, consequently, leads to cellular apoptosis.^205,206^ MicroRNA-146a is a highly specific marker for the diagnosis of CMPP^207^ Prolactin blockade by bromocriptine or cabergoline, a dopamine-D2 receptor agonist, has shown promising results in therapy and recovery of myocardial function in PPCM.^207-211^

The main risk factors for PPCM are hypertensive pregnancy syndromes^212^ (gestational hypertension, preeclampsia, eclampsia or HELLP syndrome), chronic hypertension, multiple pregnancies, obesity, smoking, pre-diabetes and diabetes mellitus, advanced age or adolescence and prolonged use of beta agonists.^213^

Mortality rates may be lower than 5%, or they may be as high as 50% of cases. The causes of maternal death are HF, ventricular arrhythmia, and thromboembolism, which mainly occur during the first 6 months of the disease until the first postpartum year (late maternal death), which may lead to underreporting of the disease.^214,215^

The main clinical manifestations are progressive or sudden dyspnea with acute pulmonary edema or cardiogenic shock. Cardiac arrest, severe arrhythmias or thromboembolic events [stroke, mesenteric ischemia or acute myocardial infarction (AMI)] and cardiogenic shock as the first manifestation of the disease are not uncommon.^216^

The diagnosis of PPCM should always be considered when cardiac decompensation occurs in the last months of pregnancy or the months following delivery in previously healthy women.^201^ The diagnosis of PPCM is by exclusion and should have differential diagnosis with myocarditis, acute myocardial infarction, pulmonary thromboembolism (PTE), severe preeclampsia, amniotic fluid embolism, pre-existing cardiomyopathies, Takotsubo syndrome, congenital or valvular preexisting disease, and systemic infections. Do not value the symptoms, such as exertion, chest pain, or fatigue, which usually occur in late pregnancy and postpartum, contribute to delay in the diagnosis of PPMC , and consequently, a worse prognosis and less chance of recovery of myocardial systolic function.^201,215,217,222^

Complementary examinations include the following:^201^

- ECG: in most cases presents nonspecific changes in ventricular repolarization, sinus tachycardia or ventricular arrhythmias. Normal ECG does not exclude the diagnosis of PPCM;
- Chest X-ray: The most frequent alterations are cardiomegaly, redistribution of blood flow the pulmonary apices, and “butterfly” pattern;
- Biomarkers: Natriuretic peptides (B-type natriuretic peptide [BNP] / or NT-proBNP) are valid markers in HF investigation because, when elevated, they help establish the diagnosis and, when normal, exclude the diagnosis. BNP level is not significantly elevated in pregnancy and postpartum, the significant increase in BNP or NT-proBNP levels in pregnancy can diagnose PPCM; reference values for HF diagnosis are NT-proBNP > 300 pg/ml and BNP > 100 pg/ml; BNP has a good predictive value for persistent left ventricular systolic dysfunction after delivery and is correlated with left ventricle echocardiographic parameters;
- Troponins: Troponin may be slightly elevated in PPCM; have predictive value for persistence of ventricular dysfunction 6 months after the onset of the disease;
- Transthoracic Doppler echocardiography is the “gold standard” examination for diagnosing PPCM. Left ventricular hypokinesis findings predominate, with LVEF below 45% and may present with regurgitation of the atrioventricular valves and pericardial effusion. LVEF below 30% and final left ventricular diastolic diameter > 60 mm are correlated with worse maternal prognosis;
- CMR provides information on the degree of myocardial involvement and should be considered for estimation of prognosis and treatment in the late course of the disease;
- Coronary cineangiography and myocardial biopsy are not indicated for diagnosing PPCM.

Time to diagnose PPCM is crucial for patient survival. The immediate goals in acute treatment are to stabilize the hemodynamic state, providing symptomatic relief and ensuring maternal and fetal well-being. Emergency physicians should be aware of PPCM in the differential diagnosis of dyspnea in pregnancy-related emergencies and play a role in early diagnosis. Care should be provided by a multidisciplinary team including cardiologists, intensivists, obstetricians, neonatologists, anesthetists and cardiac surgeons. For rapid diagnosis and decision making in all pregnant women with acute heart failure, a pre-specified management algorithm and the establishment of a multidisciplinary team is crucial.^221,222^

The pharmacological treatment of PPCM^218,219^ follows the guidelines of HF with reduced echocardiographic ejection fraction (EF). Beta-blockers, preferably β1-selective (carvedilol, bisoprolol, and metoprolol), are initiation with low doses associated with loop diuretics; digoxin may be considered in heart rate control indicated at initially low doses associated with loop diuretics; digoxin may be considered in heart rate control. It is important emphasis that ACEI, ARB, sacubitril/valsartan, ivabradine, spironolactone, and warfarin are contraindicated during gestation, but they may be considered during lactation. Is recommend anticoagulation with heparin to avoid cardio-embolic complications in patients with LVEF ≤ 35% with LMWH or oral anticoagulation at least in prophylactic dose.

The use of bromocriptine (ergot alkaloid) and cabergoline (dopamine D2 receptor agonist) has shown satisfactory results in the immediate response and late recovery of PPCM ventricular dysfunction.^208-211^ The eventual contraindication to the use of these medications should also be weighed. If bromocriptine is not available, cabergoline may be used as an alternative to bromocriptine. As thromboembolic events have been reported during the use of bromocriptine (albeit mostly at higher dosages), bromocriptine treatment should always be accompanied by anticoagulation at least in prophylactic dosages heparin; full doses of heparin (fractional/unfractionated) is mandatory in the presence of intracardiac thrombus or systemic embolism, as well as in paroxysmal or persistent AF.^222^ The proposed schedule shows that safe doses with good tolerance and efficacy are 2.5 mg twice daily for 2 weeks, followed by 2.5 mg once daily for 6 weeks for bromocriptine; and 1 mg single dose for cabergoline for its prolonged effect from 14 to 21 days.^218-219^The abbreviation **BOARD** has been proposed for chronic treatment of patients with PPCM following delivery. The abbreviation stands for **B**romocriptine, **O**ptimization of HF therapy, **A**nticoagulation, Vaso**R**elaxants, and **D**iuretics.^218^

Bromocriptine treatment must always be accompanied by anticoagulation with heparin (LMWH or UFH), at least in prophylactic dosages; in patients with intracardiac thrombus detected by imaging or evidence of systemic embolism, as ell as in patients with paroxysmal or persistent atrial fibrillation.^222^

Regarding the non-pharmacological treatment of PPCM, ICD, cardiac resynchronization, ventricular assist devices, and cardiac transplantation are considered.^220-222^ ICD has been considered for primary prevention of sudden death, following guidelines for patients with ventricular EF below 35%. Wearable defibrillator cardioverter is an alternative during the first months following diagnosis with PPCM, considering that most of these patients recover ventricular function 6 months after the acute phase of the disease.

Cardiac resynchronization may be proposed 6 months after the onset of the disease, in accordance with conventional indication criteria, or be it, advanced HF, NYHA FC III-IV with optimized treatment, sinus rhythm, EF below 35%, QRS > 150 ms, or QRS > 120 ms with desynchronization on echo or magnetic resonance.

Left ventricular assist devices may be an option in critically severe patients as a “bridge to transplant” or a “bridge to recovery.” Cardiac transplant is indicated in approximately 10% of PPCM cases in patients who do not recover after 12 months with mechanical circulatory support.

During long-term clinical follow-up, the following recommendations should be followed:^221^

1. If the cardiac function does not improve, maintain beta-blocker, ACEI or ARB; spironolactone if EF < 40%, ivabradine if heart rate > 75 bpm, with a maximum dose of beta-blocker (reaching heart rate < 60 bpm); diuretics if there is edema / pulmonary congestion;
2. If ventricular function shows complete and sustained recovery, supported by bi-annual echocardiographic follow-up, maintain pharmacological treatment (beta-blocker, ACEI, spironolactone) for at least 6 months and diuretics only if there are symptoms of congestion or lower limb edema; during the period between 6 and 12 months thereafter, discontinue spironolactone and ivabradine (if in use), but continue beta-blocker and ACEI/ARB for at least 6 months following discontinuation of spironolactone; after 12 months, gradually reduce and discontinue ACEI/ARB, and maintain beta-blocker for 6 more months; after 18 months, suspension of the beta-blocker is controversial, because some studies claim that it should be maintained for at least 5 years;
3. Advising against subsequent pregnancy in patients who have completely recovered left ventricular systolic function following PPCM is controversial, giving that there is no conclusive evidence supporting this advice in medical practice.^218^

The following points resume recommendations for practice in cases of acute HF:^208,222^

1. Transcutaneous monitoring of oxygen saturation;
2. Oxygen therapy: oxygen saturation < 90% (pulse oximetry); PaO_2_ < 60 mmHg (arterial-blood gas test);
3. Endotracheal intubation performed in acute respiratory insufficiency with hypoxemia (PaO_2_ < 60 mmHg), hypercapnia (PaCO_2_ > 50 mmHg), and acidosis (pH < 7.35);
4. Diuretics if there are signs of congestion (furosemide, 20 to 40 mg) in an intermittent bolus or a continuous infusion;
5. Vasodilators if SAP > 110 mmHg; intravenous nitroglycerin, at an initial dose of 10 to 20 μg/min, up to a maximum of 200 μg/min;
6. Inotropic agents (dobutamine, dopamine, levosimendan, phosphodiesterase III (PDE III) inhibitors in hypotensive patients (SBP < 90 mmHg) and/or signs of low cardiac output; experimental evidence and clinical experience suggest that catecholamines such as dobutamine are less favorable in patients with PPCM due to metabolic impairment levosimendan can be considered as the preferred inotropic agent, with continuous infusion of 0.1 µg/kg/h for 24 h without an initial bolus dose for patients with severe PPCM; if levosimendan is not available, dobutamine is the other option, while adrenaline should be avoided.
7. Vasopressor agents for cardiogenic shock; noradrenaline should be the first-line vasopressor.
8. Anticoagulation with full-dose LMWH, provided there are no contraindications;
9. Mechanical circulatory support as a “bridge to decision” for cardiac transplantation.

**3.3.7.1. Key Points**

- The etiopathogenesis of PPCM has yet to be fully clarified;
- Immediate diagnosis and treatment at the onset of symptoms are fundamental to ventricular recovery;
- The use of prolactin inhibitors (bromocriptine or cabergoline), combined with optimized treatment for HF is differential for the recovery of ventricular function;
- Approximately 50% of patients with PPCM recover myocardial function within 6-month period with therapy for HF;
- Even if ventricular function is recovered, follow up should be periodic for at least 5 to 10 years, following diagnosis;
- As a result of the lack of evidence regarding the actual recurrence of PPCM during subsequent gestations, there is no justification for advising against conception in patients who have truly recovered ventricular function;
- Patients with PPCM who have received transplantation have an immediate and late postoperative prognosis like that of patients with other forms of dilated cardiomyopathy.

## 3.4. Ischemic Heart Disease

Ischemic heart disease (IHD) is not common during pregnancy; most publications consider acute coronary syndrome rather than stable ischemic disease.^223^ Data from the WHO have shown that the rate of acute infarction is 3.34 events per 100,000 pregnancies, it being most frequent during the third trimester of gestation.^224^ The incidence of infarction without ST-segment elevation is higher during gestation.^52^

Risk factors for IHD during gestation are maternal age (over 40 years old; for each year of life, there is a 20% increase in the risk of infarction), family history of premature coronary disease, tobacco use, arterial hypertension, dyslipidemia, and diabetes mellitus.^52^

Additional risk factors include preeclampsia, thrombophilia, postpartum infection, cocaine use, multiparity, autoimmune diseases, aortic valve stenosis/aortic valve prosthesis thrombosis, mitral stenosis, and PPH.^52^

The etiology of IHD during gestation differs from the general population. In a contemporary review,^225^ the mechanisms related to infarction were identified with following incidences: spontaneous coronary artery dissection (43%), atherosclerosis (27%), coronary thrombosis (17%), normal arteries on angiography (9%), vasospasm (2%), and Takotsubo syndrome (2%).

**Spontaneous coronary artery dissection** is the most common cause of AMI during gestation and the postpartum period, with a prevalence of around 1.81 events per 100,000 pregnancies, occurring most frequently during the third trimester. The outcome of dissection associated with pregnancy appears to have a worse prognosis than dissection unrelated to pregnancy.^226^

Demographic variables and associated comorbidities include the following: black race, chronic hypertension, gestational hypertension, preeclampsia, lipid abnormalities, chronic depression, migraine, advanced maternal age, first delivery, and infertility treatment.^226^

The etiology of coronary dissection has yet to be made clear, but it appears to be related to degradation and weakening of arterial walls, as a consequence of the influence of hormones during gestation. The most common maternal complications described are cardiogenic shock (24%), ventricular fibrillation (VF) (16%), and mechanical support (28%), which result in hospital death in 4% of cases.^226^

**Atherosclerosis:** IHD caused by atherosclerosis is linked to the presence of classic risk factors and to those referred to as emerging risk factors, including gestational hypertensive disease, gestational diabetes, history of premature delivery, autoimmune diseases (lupus erythematosus, rheumatoid arthritis, scleroderma), treatment with thorax radiotherapy/chemotherapy, and depression/general anxiety.^227^

**Thrombosis:** Coronary thrombosis, in the absence of atherosclerosis, is more probable due to hypercoagulability during pregnancy, and it may result in paradoxical embolization.

**Normal arteries:** Mechanisms of AMI with normal coronary arteries continue to be unclear; they include transitory coronary spasm (increased vascular reactivity and/or use of ergotamine derivatives) or undetected coronary dissection, reflecting the limitations of the diagnosis.^52^

**Vasospasm:** It may be spontaneous or induced by drugs, hypertensive syndromes during pregnancy, increased vascular reactivity to angiotensin II and norepinephrine, endothelial dysfunction, or renin release by the gravid uterus. Vasospasm may be induced by routine obstetric drugs, such as beta-agonists (terbutaline, salbutamol), inhibition of premature labor, ergot derivatives for labor induction or PPH prevention, and bromocriptine, indicated for inhibiting lactation.^227^

**Other causes**: coronary artery aneurysm related to Kawasaki disease.^52^

Diagnosis of AMI is not influenced by the status of pregnancy, and it includes symptoms (dyspnea and chest pain), laboratory examinations (increased troponin), ECG (specific and classic alterations of AMI), and echo (alterations in segmental wall contractility). Differential diagnosis of AMI during pregnancy should be done with pulmonary embolism, amniotic fluid embolism, dissection of the aorta, PPCM, and myocarditis. Additional examinations for diagnosis risk stratification and treatment of AMI include scintigraphy, magnetic resonance, and coronary angiography.

Patients with acute coronary syndrome should receive defined diagnosis and treatment before delivery. Therefore, in cases with chest pain or suspected acute ischemic disease, we are in favor of indicating coronary angiography, which, in addition to concluding diagnosis, increases the chance of treating the artery “responsible” for the acute ischemic condition. The risks of angiography are relatively low in relation to the benefits for planning delivery and anesthesia in these patients.

Treatment for AMI during pregnancy is similar to that of the general population, including revascularization techniques. In cases of coronary dissection, clinical treatment has been the first choice. Percutaneous or surgical intervention is reserved for cases with left coronary trunk involvement or proximal anterior descending lesion.^226^ The most frequent complications are HF and cardiogenic shock (38%), arrhythmias (12%), recurring angina and reinfarction (20%), maternal mortality (7%), and fetal death (7%).^52^Clinical practice for cardiogenic shock and cardiorespiratory arrest follows conventional guidelines, with the strategy of emergency delivery in cases with fetal viability.^52^

Pharmacological treatment of AMI is similar to that recommended for the general population. Aspirin is safe in low doses;^92^ there is, however, little information regarding P2Y12 inhibitors.^73^ Clopidogrel is approved for use, but it should be suspended 7 days before delivery. There is no evidence on the benefits of using this medication for coronary dissection; additionally, glycoprotein IIb/IIIa inhibitors, bivalirudin, prasugrel, and ticagrelor are not recommended.^73^The use of beta-blockers, excluding atenolol, has already been established for acute coronary syndrome. Recombinant tissue plasminogen activator (TPA) does not cross the placenta, but it may induce hemorrhagic complications (subplacental bleeding).^52^Benefits of short-term heparinization probably outweigh the risk of hemorrhagic complications.

Patients with previous IHD may receive approval for subsequent pregnancy if there are no residual ischemia or signs of ventricular dysfunction. There are no high-quality data defining how much time pregnancy should be delayed following acute coronary syndrome. However, the recommendation of 12 months seems reasonable; it should be individualized according to comorbidities, cardiovascular status, and need for medical therapy.

### 3.4.1. Key Points

- The growing incidence of IHD during pregnancy is due to higher maternal age and the growing presence of risk factors;
- The incidence of AMI without ST-segment elevation is higher during pregnancy, and the anterior descending artery is the most affected;
- The clinical picture of coronary artery dissection seems to be more severe during gestation, in comparison with the general population;
- Coronary vasospasm may occur as a consequence of obstetric medications;
- Symptoms, ECG, elevated serum troponin, and alterations on echo define diagnosis of acute coronary syndrome;
- Coronary cineangiography should be indicated to define diagnosis and make percutaneous treatment possible;
- Treatment follows the general rules, with eventual restrictions on gestation;

## 3.5. Dyslipidemia

### 3.5.1. Lipid Changes

During pregnancy, a substantial increase occurs in plasma concentration of lipoproteins, as result of the increase in circulating estrogen and progesterone. Triglycerides increase 2- or 3-fold in relation to pre-gestational values, reaching their peak by the end of gestation, with a progressive return to baseline values at the end of the postpartum period. In the same manner, there is a progressive increase in total cholesterol levels, corresponding to 2- to 5-fold before pregnancy values. Their decrease is slightly slower than that of triglycerides, and they may take longer than 6 weeks after delivery to normalize.^228^

Lipoprotein fractions also present qualitative increase of high density lipoprotein cholesterol (HDL-c), low density lipoprotein cholesterol (LDL-c) and amount of triglycerides. The HDL-c has slightly different behavior from total cholesterol and triglycerides, because it raises a progressive values at 24^th^ week, of 50% in comparison with preconception period. Subsequently, it presents a drop, equivalent to 15% higher than pre-gestational values until the end of pregnancy.^228^LDL-c levels increase in synchronization with total cholesterol. However, they showed decrease and may fall after the eighth week postpartum.

The factor responsible for these alterations in lipoproteins is the hormone estrogen. The drop in HDL-c after week 24 is understood by the increase in plasma concentration of insulin, which represents an increase in insulin resistance. It is, therefore possible to conclude that HDL-c levels are more related to estrogen levels during the first phase of gestation and to insulin during the second phase. It isrecommended that lipid profile test by postponed to, at least, 4 to 6 weeks after gestation, especially in women without previous alterations.

In an update to the Brazilian Guidelines on Dyslipidemia and Atherosclerosis Prevention, the recommendations for women of fertile age with dyslipidemia include dietary orientation and adoption of a healthy lifestyle, in addition to weight control, physical activity, and ceasing tobacco use.^229^ Therapy with statins should be avoided in women of fertile age who plan to become pregnant (class II-A; C).

Gestational hypertriglyceridemia occurs to meet maternal energy demands, as a precursor of hormones for the placenta and to provide cholesterol and essential fatty acids to the fetus. Statin therapy should not be indicated for pregnant women in the second and third trimester or in breastfeeding women (class III-C). This contraindication is due to reports of teratogenicity, although the information available in the literature is inconclusive.^104^

Fibrates, ezetimibe, niacin, cholesterolamine and omega-3 are considered to be drugs without contraindication, but cholesterolamine is the only one whose safety has been established. Fibrates may be used in cases of very severe hypertriglyceridemia (plasma level of triglyceride > 1,000 mg/dL), with risk/benefit analysis for pregnant women (high maternal and fetal mortality due to acute pancreatitis). However, dietary control should be the treatment of choice for pregnant women (class IIA; C); in extreme cases, apheresis may be recommended.^230^

Regarding omega-3 fatty acids, pregnant and breastfeeding women should advised to introduce omega-3-rich fish, from deep water and with low mercury levels, into their diets. Salmon, mackerel, herring, sardines, tuna and trout are recommended. There are no studies on supplementation (capsules) and phytosterols during gestation.

Genetic dyslipidemias should be considered, both hypertriglyceridemia with frequent pancreatic complications and familial hypercholesterolemia. Apheresis is a special therapeutic approach to these severe circumstances; in familial cases, selective LDL-apheresis is used.^231^

Until recently it was accepted that dyslipidemia during pregnancy should be considered physiological, to the extent that lipid profile testing is not part of the prenatal routine. Recently, however, fatty striae have been described in the aorta of dyslipidemic mother fetuses. Based on these observations, it has been suggested that maternal cardiometabolic dysfunction may not only contribute to long-term maternal effects, but it may also lead to a risk of atherosclerosis in future generations. These considerations suggest that diagnosis and treatment of dyslipidemias should be performed prior to conception, and they should continue during gestation and the postpartum period.^232^

### 3.5.2. Key Points

- Increases occur in triglycerides and cholesterol during pregnancy;
- The use of statins is not recommended, although there is some controversy regarding their teratogenic effects;
- Maternal dyslipidemia may induce fetal atherosclerosis and atherosclerosis in future generations.

## 3.6. Other Diseases

### 3.6.1. Takayasu Arteritis

Takayasu arteritis is a chronic, idiopathic vasculitis that predominantly affects the aorta and its main branches, coronary arteries, and the pulmonary artery. The resulting inflammatory process causes narrowing, occlusion, and aneurysm in the affected branches.^233^ Etiology of the disease is unknown, but several studies have demonstrated an association with human leukocyte antigens, suggesting a predisposition to the immune-mediated process.^234^

***3.6.1.1. Prevalence***

Takayasu arteritis is a rare disease, with growing rates of prevalence. The highest rates occur in Japan, with 100 to 200 new cases annually. Women are more affected, in 80% to 90% of cases; the onset of the disease occurs between 10 and 40 years of age, overlapping with the fertile period of life, and gestation demands special attention. It is the most frequently observed form of vasculitis during pregnancy, precisely because it appears in young patients.^235^ Maternal immune activation during pregnancy may influence the course of the disease and impair maternal and fetal outcome.^236^

***3.6.1.2. Prognosis***

Pregnancy in patients with Takayasu arteritis has an uncertain prognosis. Although the majority of gestations are successful, the incidence of severe hypertension and preeclampsia is 40% higher, when compared to 8% in the general population. Obstetric complications, such as premature delivery and stillbirth, are foreseen.^235^ Patients with renal artery and abdominal aorta involvement more frequently have complications of preeclampsia and IUGR.^235^

The rarest maternal complications, which are, however, very severe, are aortic aneurysm, stroke, HF, aortic insufficiency, myocardial infarction, and dissection of the aorta.^235^ Other, more common complications include progression of renal insufficiency, anemia, thrombocytopenia, and elevated inflammatory markers.^235^

***3.6.1.3. Treatment***

Treatment of vasculitis during pregnancy is conventional, excluding three teratogenic medications, namely, methotrexate, mycophenolate and cyclophosphamide.^236^ Other medications are considered compatible with gestation. It is preferable to use immunosuppressive drugs to control active vasculitis, reserving prednisone for a short-duration regime in moderate doses during the acute phase or in cases where the disease worsens. Treatment may be initiated before conception and maintained during pregnancy and lactation.^237^

Tumor necrosis factor inhibitors may be continued during the preconception, pregnancy and lactation. These inhibitors, when their composition is based on immunoglobulin G (IgG), cross the placenta from 16^th^ of gestation with a progressive increase in transference nearby term of gestation. Therefore, these drugs should not be administered after 30^th^ week of gestation, but they should be reintroduced in the postpartum period.^238^

***3.6.1.4. Key Points***

- Pregnancy is allowed when disease is in remission, because vasculitis has severe prognosis;
- Treatment with corticosteroids and immunosuppressive drugs (azathioprine, cyclosporine, and tacrolimus) improves maternal-fetal evolution;
- In cases of systemic vasculitis, seeing that the risk of thromboembolic events is elevated, prevention with aspirin or LMWH should be considered;
- Takayasu arteritis should always be considered in differential diagnosis of arterial hypertension during pregnancy;
- Contraception should be efficacious and safe during treatment with high doses of cytotoxic drugs.

### 3.6.2. Kawasaki Disease

Kawasaki disease is a systemic vasculitis of unknown etiology that occurs in children up to 5 years of age, with Asian prevalence and a male predominance of 1.5 para 1. During the acute phase, inflammatory involvement of coronary arteries results in clinical outcomes and provokes aneurysm formations in 15% to 25% of untreated children. It is one of the main causes of heart disease acquired during childhood.^239^

Coronary artery aneurysms may be detected early on echo and loss of laminar flow in these arteries may favor clot formation.

Disease prognosis is related to presence and size of coronary artery aneurysms. Small aneurysms have favorable prognosis, with low risk of myocardial ischemic events. In contrast, large and giant aneurysms (internal diameter > 8 mm) present a high risk of thrombosis and, consequently, myocardial infarction, arrhythmias, and sudden death.^240^

Lack of diagnosis and treatment during the acute phase in childhood has contributed to the finding of women with vascular sequelae of Kawasaki disease during fertile age and pregnancy.^241,242^The influence of the hypercoagulable and hyperkinetic states inherent to pregnancy, delivery, and the postpartum period represents a potential risk of severe events, such as thrombosis, myocardial infarction, and sudden death, throughout the natural history of women with complicated Kawasaki disease with coronary aneurysms. In addition to this, pregnancy, *per se*, favors the risk of coronary artery rupture and/or dissection, as a result of specific changes in the artery walls, which include fragmentation of reticular fibers, reduction of mucopolysaccharides and loss of normal elastic fibers ripple.

In keeping with this logic, it is accepted that the state of hypercoagulability during pregnancy and the postpartum period requires permanent anticoagulation. Therefore, low-dose aspirin (80 mg daily) up 36^th^ week of gestation combined with anticoagulation, should be considered. LMWH is recommended during the first trimester and after week 36 of gestation, with low doses of warfarin in the interval between these 2 periods. In the literature, there is a lack of data regarding targets for prevention; nonetheless, the consensus is that INR around 2 is safe and presumably efficacious.

Previous myocardial infarction increases the risk of gestation, and ventricular function is a determining factor for maternal evolution. Beta-blocker (propranolol or metoprolol succinate) use in low doses favors lower oxygen consumption, as a function of less cardiac work.

***3.6.2.1. Preconception Evaluation***

In risk stratification for future pregnancy, the presence of coronary artery aneurysm, myocardial ischemia, and ventricular dysfunction should be considered.

***3.6.2.2. Key Points***

- Existence of moderate coronary aneurysm (> 3 mm and < 6 mm) in one or more arteries indicates permanent use of low doses of aspirin;
- Giant (> 8 mm) or multiple aneurysms, in addition to aspirin, require association with an anticoagulant;
- In cases of myocardial ischemia, association of aspirin, an anticoagulant, and/or CCB is recommended.

### 3.6.3. Pulmonary Hypertension

PH is a physiopathological condition that leads to debilitating symptoms and lower life expectancy, caused by compromised pulmonary circulation. It is defined as average resting pulmonary artery pressure (PAP) ≥ 25 mmHg, measured by right heart catheterization. It is a progressive disease, predominant in the female sex, and it may occur during the reproductive period. In general, it leads to right ventricular insufficiency with a risk of death during pregnancy, but especially during the postpartum period.^243,244^

Pregnancy in women with PH is considered high risk and maternal and the neonatal complications rate achieve 50 to 70% respectively, and it has been associated with mortality rates reaching nearly 30%.^245^ In view of this, pregnancy is contraindicated.

Physiological changes of pregnancy, especially the decreased peripheral vascular resistance, increased cardiac output, and hypercoagulability, are reasons for maternal hemodynamic instability. In addition to this, there is the activity of sex hormones, such as beta-estradiol, progesterone, and testosterone, in pulmonary circulation; on one hand, they attenuate pulmonary vasoconstriction, and, on the other hand, they activate angiogenic factors that stimulate the proliferation of smooth muscle cells in pulmonary vasculature, predisposing them to reverse vascular remodeling.

This physiopathological complexity of PH during gestation may be resumed in a single primary aspect, namely, the compensatory physiological vasodilatory response of pulmonary vasculature, which becomes decreased or absent, leading to a significant increase in pulmonary pressure and resistance. The inability of the pulmonary vascular bed to accommodate increased cardiac output results in significant disproportion in right ventricular afterload and failure.^246^

The classification of PH was simplistic, divided into two groups: primary and secondary, according to identification of risk factors. However, since 1998, the WHO has proposed modifications to the classification of PH in order to allow different types of the disease to be grouped based on their physiopathology, response to treatment, and prognosis^247^(Table 25). It is worth remembering that, in this classification, the term PAH is described as a subgroup of PH, characterized by left ventricular filling pressure < 15 mmHg and pulmonary vascular resistance > 3 Wood units.

**Table 25:** – Classification of pulmonary arterial hypertension

**Category 1**	Idiopathic
	Hereditary
	Induced by drugs and toxins: anorectic agents, chemotherapy, serotonin reuptake inhibitors, cocaine
	Associated with congenital heart disease, collagen disease, HIV infection, portal hypertension, schistosomiasis
	Pulmonary capillary hemangiomas or veno-occlusive pulmonary disease
	Persistent pulmonary hypertension in the newborn
**Category 2 - Pulmonary hypertension due to left heart disease**	Diastolic dysfunction
	Systolic dysfunction
	Valve disease
	Congenital/acquired left heart obstruction and outflow tract obstruction and congenital cardiomyopathies
**Category 3 - Pulmonary hypertension due to pulmonary disease and/or hypoxemia**	Chronic obstructive pulmonary disease
	Interstitial pulmonary disease
	Pulmonary diseases with mixed patterns, i.e, restrictive and obstructive
	Obstructive sleep-disordered breathing
	Alveolar hypoventilation
	Chronic exposure to high altitudes
	Occupational pulmonary diseases
**Category 4**	Pulmonary hypertension due to chronic thromboembolism
**Category 5 - Pulmonary hypertension with unclear multifactorial mechanisms**	Hematologic disorders: chronic hemolytic anemia, myeloproliferative disorders, splenectomy
	Systemic disorders: sarcoidosis, pulmonary histiocytosis, lymphangioleimoyomatosis
	Metabolic disorders: glycogen storage disease, Gaucher disease, thyroid disorders
	Others: tumoral obstruction, fibrosing mediastinitis, chronic renal insufficiency, segmental pulmonary hypertension

*HIV: human immunodeficiency virus.*

Regarding diagnosis of PH, symptoms such as dyspnea, chest pain, lower limb edema, palpitation, and dry cough may be attributed to pregnancy, but the presence of syncope attributes more severity to the disease.^248^ECG and chest X-ray show right chamber overload. Transthoracic echo estimates PAP, evaluates right ventricular function, and identifies other structural heart diseases, making it possible to classify the type of PH. Definitive diagnosis is done by means of right heart catheterization and pressure measurements.^246,247^

Family planning in patients with PH includes advising against pregnancy by clarifying the maternal and fetal risks, as well as the choice of an efficacious and safe means of contraception. There is no evidence to date regarding a pulmonary arterial pressure level (cutoff point) for determining prognosis for a future pregnancy.

However, the pregnancy outcome is very different when subgroups for classification of PH are taken into consideration.^248^ It is worth emphasizing that patients included in category 2 (Table 25), such as those with mitral stenosis, aortic stenosis, and cardiomyopathies, receive different treatment and counseling than patients included in the other categories.

For this reason, risk stratification according to category and treatment strategy for pregnancy should receive interdisciplinary support in a tertiary hospital that has specialists in PH, so that the best practice may be adopted.

Excluding pregnant women included in category 2, the first proposal over the course of the first trimester in patients with PAH is to interrupt pregnancy, with an emphasis on clarifying the risks of maintaining pregnancy and those of therapeutic abortion procedure. In the event that the patient does not accept this advice, the following practice is currently recommended:^249^

1. Weekly interdisciplinary consultation starting at week 16 of gestation;
2. Individualized pharmacological therapy for PH;
3. Periodic evaluation of ECG, echo, and BNP during the second and third trimesters;
4. Hospitalization starting at week 28 for therapy with intermittent oxygen in accordance with arterial oxygen saturation, anticoagulation, maternal-fetal monitoring, and delivery planning;
5. Route of delivery is indicated by the obstetrician;
6. General anesthesia is preferable;
7. Anesthesia with blocks (epidural or spinal anesthesia) is contraindicated.

Recommended pharmacological therapy is use of prostacyclins and their analogues and type 5 phosphodiesterase inhibitors, which seem to be safe during gestation. CCB are a safe and efficacious alternative for the subgroup of patients who present documented vasoreactivity and NYHA FC I/II without severe ventricular dysfunction; nevertheless, it is necessary to be attentive to their negative inotropic effects, in addition to arterial hypotension, which may limit their use.^250,251^

Parenteral prostaglandins are recommended in patients in NYHA FC IV or patients who show evidence of severe right ventricular involvement. Most experience is with intravenous epoprostenol. In patients with preserved ventricular function who are in NYHA FC I/II, inhaled prostaglandins, such as Ilosprost, may be indicated. Parenteral prostaglandins may be combined with oral phosphodiesterase inhibitors, with satisfactory results.^252^

Endothelin receptor blockers and soluble guanylate cyclase stimulators are contraindicated in pregnancy.^251,252^Intravenous prostaglandins may be considered during delivery, with invasive monitoring via CVC and arterial access.

It is worth remembering that a large number of maternal deaths occur during the postpartum period, with the following causes standing out: HF due to right ventricular failure, hypoxemia, and thromboembolism (pulmonary thrombosis *in situ*).^248^ Therefore, anticoagulation is essential, with therapeutic doses of LMWH (1 mg/kg every 12 h) during the first trimester and after 36th week of gestation and, warfarin in a daily dose, INR target of 2, another other periods (Figura 6).

***3.6.3.1. Key Points***

- Diagnosis of PAH should be confirmed by right chamber catheterization.
- Pregnancy should be advised against in women with PAH;
- Categories of PH according to current classification have different prognoses and treatments;
- The proposal to interrupt pregnancy should be considered in patients with PH during the first trimester, except in patients in category 2;
- In pregnancy in maintained, the prenatal care and delivery should be at a tertiary hospital with specialized PH staff;
- Current pharmacological therapy has assisted in successful pregnancy in PH.

### 3.6.4. Aortic Diseases

Aortic diseases may be present in women of reproductive age, and they are considered to be important causes of complication and even death during gestation.^253^ This is due to 3 physiological phenomena of pregnancy that have detrimental impacts of aortic diseases. These phenomena are hemodynamic (increased cardiac output), structural (progressive aortic root growth until the third trimester), and hormonal (vascular wall fragility). The following are the most frequent causes of aortic disease in pregnant women: bicuspid valve, Marfan syndrome, coarctation of the aorta, Ehlers-Danlos syndrome, Turner syndrome, and Loeys-Dietz syndrome.

***3.6.4.1. Aortic Dissection and Rupture***

Gestation increases a woman’s susceptibility to dissection and rupture of the aorta. In the general population, the incidence of aortic dissection is 6 cases per 100,000 individuals yearly; nevertheless, during pregnancy, the occurrence increases 100-fold, to approximately 0.6%.For this reason, diagnosis of aortic dissection should be considered in all patients with chest pain during pregnancy. It occurs more frequently during the last trimester (50%) or the initial postpartum period (33%).^52^

Marfan syndrome is the most common conjunctive tissue disease, caused by a mutation in the FBN-1 gene, which codifies the inherited glycoprotein fibrillin in an autosomal dominant pattern.^254^ Average increase in aortic diameter growth during gestation in patients with Marfan syndrome is 0.3 mm/month, while in the general population with Marfan syndrome, it is 0.38 mm/year.^254^ This increased rate of aortic dilatation decreases after delivery, but it remains higher than the pre-gestational rate.^253^

Diagnosis includes history, physical examination, echo, and magnetic resonance of the aorta. Thoracic aortic angiography complements investigation when there is strong suspicion of dissection based on previous examinations.

One of the most important factors for determining the risk of dissection of the aorta is maximum diameter (< 40 mm, 1% risk of dissection; > 40 mm, 10% risk of dissection).^255^ Pregnancy is generally contraindicated if the ascending aorta diameter is greater than 40 mm in patients with family history of dissection or sudden death, even though the exact dimension is still a question of debate.^254^ There seems to be a low incidence of dissection if aorta diameter is lower than 4.5 cm; pregnancy, however, increases the late risk of aortic complications.^52,254,256^

One important consideration is body surface area, especially in small women. Aorta diameter index higher than 27 mm/m^2^ is associated with a high risk of dissection, and prophylactic replacement of the aortic root should be considered.^52^

It is also necessary to evaluate associated cardiovascular problems, including the possibility of aortic regurgitation and mitral valve prolapse with associated regurgitation.

Beta-blockers have been shown to increase aortic distensibility and reduce pulse wave velocity and reducing the rate of complications such as regurgitation, dissection, and congestive HF. A 20% decrease in resting heart rate is considered to be the objective of treatment.^257^

Periodic echocardiographic monitoring is recommended every 6 to 8 weeks to monitor the size of the mother’s aortic root; the interval depends on initial echocardiographic findings.^254^

The preferred route of delivery is cesarean in patients with aortic dilatation > 45 mm, and delivery should take place in a tertiary center where there is an experienced surgery team. In patients with diameters < 45 mm, with no previous events, delivery may be vaginal, with early analgesia and relief forceps.

Preconception counseling requires determination of the underlying disease, genetic evaluation, and aortic dilatation correction in accordance withdiameter thresholds (Table 26).

**Table 26:** – Aorta diameter thresholds and indication for intervention in patients considering pregnancy257

Underlying disease	Ascending aorta diameter
Marfan syndrome	45 mm
Loeys-Dietz syndrome	40 – 45 mm
Ehlers-Danlos syndrome type IV	Pregnancy contraindicated
Bicuspid valve	50 mm
Turner syndrome	27 mm/m^2^

Ehlers-Danlos syndrome type IV occurs with severe vascular complications, with characteristics of autosomal dominant inheritance and 50% risk of transmission to offspring.

Maternal mortality is significant, and it is related to uterine rupture and dissection of the great arteries and veins. Pregnancy is, therefore, considered a high-risk situation, and it is not advised (WHO risk IV); in this manner, when contemplating pregnancy, these women should be advised in a shared decision-making process.^52^

In vascular Ehlers-Danlos syndrome, also a rare severe conjunctive tissue disease, characterized by fragile vascular tissue, vascular rupture has been related during pregnancy in up to 50% of cases, with mortality rates between 5% and 50%. Gestation, in these cases, is also associated with premature rupture of fetal membranes, spontaneous abortion, and prematurity.^52^

Turner syndrome^256^ is the most common sexual chromosome abnormality in women, and it occurs in 1 of every 1,500 to 2,500 female live births. Chromosome constitution may be absence of an X chromosome (karyotype 45,X) or chromosome mosaicism (karyotype 45,X/46,XX), as well as other structural anomalies of chromosome X.^256^Turner syndrome is associated with increased risk of heart disease, aortic dilatation, hypertension, diabetes mellitus, and atherosclerotic disease events.^256^

Dissection of the aorta in patients with Turner syndrome is estimated to be 36 in 100,000 cases, but it is 6 times more common in younger age ranges than in the general population.^52^Risk factors include dilatation of the aorta, bicuspid aortic valve, and coarctation of the aorta.^52^Pregnancy should be avoided when aortic size index is > 25 mm/m^2^. Furthermore, after aortic root surgery, patients continue to be at a risk of a type B dissection.

Although spontaneous pregnancy may occur in patients with Turner mosaic (0.5% to 10%), it is more common with assisted fertility. For this reason, cardiovascular evaluation is recommended before beginning fertility treatment. Furthermore, good blood pressure and diabetes control during pregnancy is mandatory for all patients with Turner syndrome.^52^

Loeys-Dietz syndrome^258^ is an autosomal dominant condition. It was described for the first time in 2005, and it is associated with formation or dissection of an aneurysm in the aorta or in other arteries, generally at a young age.^258^It has been identified in individuals referred for investigation for Marfan syndrome^257^ or vascular Ehlers-Danlos syndrome who did not present the classical characteristics of these conditions, but rather others characteristics, including general arterial tortuosity, hypertelorism, bifid/broad uvula, or cleft palate.^257^

The syndrome results in mutations in the genes that codify components of the transforming growth factor beta (TGF-beta) signaling pathway. Aortic pathology is particularly concerning in this condition, but other vascular abnormalities may also be present.^258^

Significant maternal morbimortality has been described in patients with Loeys-Dietz syndrome, but it is possible for pregnancy to be successful and free of complications.^258^Nonetheless, all patients with this condition should, at present, be treated as high-risk during pregnancy and the postpartum period, until reliable risk prediction tools become available.^258^

There are no studies on the benefits and risks of cesarean delivery in comparison with vaginal delivery in patients with hereditary aortic disease. Cesarean delivery is, nonetheless, recommended, according to the aortic dilatation thresholds shown in Table 26. Vaginal delivery may be considered in cases below these limits.

***3.6.4.2. Key Points***

Aortic diseases constitute an important cause of maternal death during the pregnancy-postpartum cycle;

Pregnancy increases women’s susceptibility to aortic dissection and rupture;

Pregnancy planning includes diagnosis of the underlying disease, magnetic resonance of the aorta and base vessels, eventual corrective aortic surgery in accordance with limits for risk of dissection, and genetic counseling;

The occurrence of dissection of the aorta with a viable fetus (> 28 weeks of gestation) indicates emergency cesarean delivery; if, however, the fetus is not viable, the case should proceed to cardiac surgery and maintain the pregnancy;

Women with Ehlers-Danlos, Turner, or Loeys-Dietz syndrome, in addition to the high risk of dissection of the aorta, are exposed to complicated events, such as hypertension, diabetes, and other aneurysms, which, in conjunction, represent a significant increase in maternal death during pregnancy.

### 3.6.5. Chagas Disease

***3.6.5.1. Prevalence***

Global estimated prevalence of *T. cruzi* infection in pregnant women has varied from 1% to 40%, with approximately 1.8 million women of fertile age infected in Latin America.^259^In Brazil, the prevalence of infection in pregnant women is accepted to be 1.1%, with a vertical transmission rate of 1.7%.^259,260^

***3.6.5.2. Diagnosis and Practice for Cases with T. cruzi Infection during Gestation***

Serological evaluation for *T. cruzi* infection is recommended during prenatal care in pregnant women who reside in or come from endemic areas and in those who have received blood transfusions in these regions.^259,261^ The most frequently used tests are based on higher sensitivity and specificity for detecting *T. cruzi* infection. They include enzyme-linked immunosorbent assay (ELISA); indirect hemagglutination (IHA), and indirect immunofluorescence (IF). Transmission may occur at any moment during pregnancy, but specific antiparasitic treatment for *T. cruzi* infection is contraindicated during gestation and breastfeeding, owing to teratogenicity in animals. Accidental exposure to benzonidazol does not indicate adverse effects in the newborn, and it is not a criterion for interrupting gestation.^259^

Elevated maternal parasitemia is associated with a greater risk of vertical transmission and miscarriage.^261^ For this reason, during the acute phase of Chagas disease, pregnant women should be individually evaluated, and the decision to initiate antiparasitic treatment should be based on the risk-benefit ratio.

Evidence of *T. cruzi* infection does not justify indicating cesarean delivery, even though congenital *T. cruzi* infection may result in uterine growth restriction and prematurity.^259,261^ It is worth emphasizing the importance of proceeding to recommended evaluations during prenatal care, including anti-HIV tests. Simultaneous infection with *T. cruzi* and HIV represents an increased risk of congenital transmission of *T. cruzi* owing to elevated parasitemia, which also implies higher perinatal morbimortality.^260,261^ After delivery, women should be referred for clinical evaluation and specific treatment. Figure 7 shows indications for practice in Chagas disease during pregnancy.^259^

**Figure 7:** – *Flowchart for approaching Trypanosoma cruzi infection in the mother/child binomial. ELISA: Enzyme Linked Immunosorbent Assay; IF: indirect immunofluorescence; IHA indirect hemagglutination. Adapted from: Second Brazilian Consensus on Chagas Disease.^259^*

***3.6.5.3. Chronic Chagas Heart Disease***

Chagas heart disease, in its indeterminate form, does not present any additional risks to pregnancy, whereas forms with ventricular or arrhythmogenic dysfunction are associated with complications such as HF, thromboembolism, and complex arrhythmias. In these cases, pregnancy is considered high-risk, and it is advised against at times, depending on the degree of cardiac involvement, which may be established by echo with 24-Holter monitoring.

***3.6.5.4. Vertical Transmission of Trypanosoma cruzi***

Vertical transmission (from mother to child) of *T. cruzi* depends on the degree of parasitemia; transplacentally, it may occur at any stage (acute or chronic) of the disease, which requires treatment prior to gestation in infected women of fertile age. It is worth emphasizing that vertical transmission may recur during the reproductive period, and detection of vertical transmission in practice is complicated, given that most congenital cases are asymptomatic. Cases of congenital Chagas disease are considered acute, and it is compulsory to notify them within disease surveillance programs.^259,262,263^

During the acute phase of Chagas disease, there exists a possibility of transmission through breast milk, whereas, during the chronic phase, transmission occurs during lactation in cases of bleeding from fissures in the nipple rather than through milk itself.

***3.6.5.5. Reactivation of Chagas Disease***

During gestation, mechanisms and immunological alterations in the maternal organism may favor the reactivation of chronic Chagas disease in previously infected cases. Reactivation is defined by positivity on the following examinations, regardless of other signs and symptoms:

- Presence of the parasite in direct microscopic examination of blood or biological secretions, such as cerebrospinal fluid, pleura, pericardium, and ascitic fluid;
- Histopathological examination of tissue lesions (panniculitis, myocarditis, encephalitis, enteritis, colpitis) with parasite nests found in acute inflammatory infiltrates.

***3.6.5.6. Breastfeeding***

Suspending lactation is not recommended in women in the postpartum period with chronic Chagas disease, except in cases with breast fissure, in situations with elevated parasitemia, or in women in the acute phase of the disease.^259^

If a breastfeeding child is exposed to milk from an infected mother, either in the acute or chronic form, with nipple fissures, the child should be monitored for acquisition of *T. cruzi* infection during the exposure period. In some cases, it is possible to consider thermal treatment of breast milk before administration to the child.^259,262,263^

Lactation should be suspended in cases of *T. cruzi* and HIV coinfection, given that lactation, regardless of association with Chagas disease, is associated with a 7% to 22% additional risk of HIV transmission. Similarly, in cases of acute maternal HIV infection, natural breastfeeding increases the probability of vertical transmission of the virus to 29%. In Brazil, mothers have the right to receive infant milk formula until their children are at least 6 months old.^259,263^

***3.6.5.7. Key Points***

- Serological evaluation is recommended for all pregnant women who are positive for the disease;
- The risks to gestation depend on the clinical form of Chagas disease;
- Pregnancy may favor reactivation of the disease;
- Breastfeeding should not be advised against;
- Antiparasitic treatment is contraindicated during gestation and breastfeeding;
- Route of delivery is indicated by the obstetrician.

## 4. Hypertensive syndromes

### 4.1. Introduction

Hypertensive syndromes during gestation are considered to be a public health problem, with an expressive rate of maternal and fetal mortality, in both developed and developing countries. It is the most common medical complication, and it affects 5% to 10% of pregnancies worldwide.

Preeclampsia occurs in approximately 3% of all pregnancies in the United States, where it is responsible for 9% of maternal deaths.^264^ Its incidence has shown a 25% increase over the past 2 decades. In recent years, an increase has been registered in the proportion of women with preeclampsia. In 2009, it was 2.2%; in 2013, it was 5.58%, and over the past 5 years, 22.5% suffered a severe general complication.^265^

Although research has evolved in the area of hypertensive syndromes during gestation, its etiology remains unknown. The methodological challenges to research related to preeclampsia are numerous; they include defining hypertension, level of severity, and physiopathology during pregnancy. These data probably interfere with research and outcomes, which justifies the following recommendations.

### 4.2. Recommendations for Measuring Arterial Pressure

- Blood pressure measurement devices in pregnant women must be accurate and validated for this special population. The cuff should be appropriately sized 1.5 times the circumference of the arm.
- Blood pressure should be measured with the patient sitting. The patient should rest for at least five minutes before measurement. Measurement may also take place in the left lateral decubitus position, while resting, and it should not differ from the measurement taken in the seated position;
- It is necessary to consider Korotkoff phase V to determine diastolic arterial pressure (DAP);^266^
- White coat hypertension and masked hypertension are considered to be relatively common presentations during pregnancy. They occur in at least 1/3 of pregnant women, to the extent that ABPM and home blood pressure monitoring (HBPM) are useful complementary examinations for making the clinical decisions which are fundamental to avoiding treatments which are unnecessary and potentially harmful to the fetus;^33,267^
- Pregnant women with SAP ≥ 140 mmHg and/or DAP ≥ 90 mmHg are considered hypertensive;
- Severity of hypertension during pregnancy is assessed based on the occurrence of target organ involvement, as well as arterial pressure level;^268^
- Severe hypertension is defined based on pressure levels ≥ 160/110 mmHg, which are associated with increased risk of stroke in pregnant women.^52,266,268^

### 4.3. Classification

The most widely used classification for hypertensive syndromes during gestation is the one adopted by the Report of the American College of Obstetricians and Gynecologists’ Task Force on Hypertension in Pregnancy,^269^ which is also applied in the Brazilian Cardiology Society’s Guidelines on Pregnancy in Women with Heart Disease (Figure 8).^270^ In accordance with this classification, syndromes are classified in the following manner:

**Figure 8:** – *Classification of hypertensive syndromes. HELLP: hemolysis, elevated liver enzymes, and low platelet count; DAP: diastolic arterial pressure; SAP: systolic arterial pressure.*

- Chronic, preexisting hypertension (due to any cause);
- Preeclampsia/eclampsia;
- Chronic hypertension with superimposed preeclampsia;
- Gestational hypertension.

Based on this position, classification into 4 categories will be maintained, emphasizing the importance of other presentations of arterial hypertension, such as the following:

- White coat hypertension;
- Masked hypertension;
- Transitory gestational hypertension occurs without the development of preeclampsia; arterial pressure normalizes within 12 weeks postpartum and it is resolved without treatment;^267,270^
- Postpartum hypertension generally arises between 2 weeks and 6 months after delivery. It is mild and labile, and it normalizes within the first year. It may be related to persistent gestational hypertension, preeclampsia, or chronic hypertension, or it may be secondary to other causes;^269^
- Unclassified prenatal hypertension is the term used when the first pressure measurement is recorded after week 20, and it is not clear if it is chronic or preexisting; diagnosis is only established during postpartum reevaluation between weeks 6 and 12.^52^

#### 4.3.1. Chronic, Preexisting (Essential or Secondary) Hypertension

This occurs when arterial pressure is ≥ 140/90 mmHg (preexisting hypertension; in general, essential hypertension or hypertension diagnosed before week 20 of pregnancy). It is commonly diagnosed around the first trimester or right at the beginning of the second. It is associated with adverse maternal and fetal outcomes; there should, therefore, be more rigorous control of maternal arterial pressure (110 to 140/85 mmHg), monitoring fetal growth and repeatedly evaluating the development of preeclampsia and maternal complications.^267^

Hypertension may not be diagnosed in many women whose first prenatal consultations occur during the second trimester. Pregnant women may be considered normotensive during the initial phase of gestation, due to the physiological decrease in arterial pressure during the first trimester of pregnancy, in the same manner that an increase in arterial pressure may be diagnosed as gestational hypertension, because pressure levels were not verified before week 20 of gestation. Chronic hypertension usually persists until 42 days postpartum.^268^

Diagnosis of chronic hypertension can only be made correctly once arterial pressure has been reevaluated after 6 to 12 weeks postpartum.^271^

#### 4.3.2. Preeclampsia/Eclampsia

This is a complex hypertensive syndrome, and it may deteriorate rapidly and without warning; classifying it as “mild” or “severe” is not recommended. Diagnosis occurs with the appearance of hypertension, with onset from week 20 of gestation, with one or more of the following related conditions:

- Proteinuria (> 0.3 g/24 h) and/or maternal organic dysfunctions, such as evidence of maternal acute renal lesions (creatinine ≥ 1 mg/dL);
- Hepatic dysfunction (elevated hepatic transaminases, > 40 IU/L);
- With or without abdominal pain (upper quadrant or epigastric);
- Neurological complications (including eclampsia, altered mental state, blindness, stroke, clonus, intense headaches, persistent visual scotoma);
- Hemolysis or thrombocytopenia and/or uteroplacental dysfunction (restricted fetal growth, abnormal analysis of umbilical artery Doppler waveform or stillbirth).

The existence of proteinuria is not mandatory for diagnosis, and it may occur for the first time during the intrapartum period. In this manner, it is ideal to identify pregnant women with a risk of developing preeclampsia. Recommendations for screening, such as investigating proteinuria to this end, are fallible; the only consensual routine is to measure arterial pressure regularly during prenatal consultations.^272,273^

***4.3.2.1 HELLP Syndrome (Hemolysis, Elevated Liver Enzymes, and Low Platelet Count)***

This is a severe manifestation of preeclampsia, and it should not be considered as a separate entity.

#### 4.3.3. Chronic (Preexisting) Hypertension with Superimposed Preeclampsia

This occurs in 25% of pregnant women with chronic hypertension. It is diagnosed when a pregnant woman with chronic essential hypertension develops maternal organic dysfunctions compatible with preeclampsia. As a routine increase in arterial pressure may occur after week 20 of gestation, elevations in arterial pressure alone do not qualify for diagnosis of superimposed preeclampsia, in the same manner that restricted fetal growth may be part of the clinical picture of chronic hypertension.

In cases of kidney disease with underlying proteinuria, an increase in proteinuria is also not a diagnostic parameter for superimposed preeclampsia; if, however, there is no preexisting proteinuria, its appearance within the context of elevated arterial pressure is sufficient for diagnosis.

#### 4.3.4. Gestational Hypertension

Gestational hypertension is a recent hypertension that arises after week 20 of gestation, in the absence of proteinuria, without any biochemical or hematological abnormalities. It is generally not accompanied by IUGR, and outcomes are frequently positive; however, approximately one quarter of women with gestational hypertension (especially those who present before week 34) evolve to preeclampsia and present unfavorable outcomes. In general, it resolves itself within 6 weeks postpartum.^52^

***4.3.4.1. Key Points***

- Consider hypertensive pregnant women, when SBP ≥ 140 mmHg and/or DBP ≥ 90 mmHg;
- Define as severe hypertension when blood pressure levels ≥ 160 x ≥ 110 mmHg. These levels are associated with increased risk of stroke in pregnant women;
- Pre-existing Chronic Hypertension (Essential or Secondary) should have tighter control of maternal blood pressure (BP = 110 - 140/85 mmHg), monitoring fetal growth and repeatedly evaluating the development of preeclampsia and maternal complications;
- Preeclampsia/Eclampsia – complex hypertensive syndrome, may deteriorate rapidly and without warning. Not recommend classifying it as "mild" or "severe";
- Proteinuria is not mandatory for diagnosis and may occur for the first time during the intrapartum period or early postpartum.

## 4.4. Treatment of Gestational Hypertension Syndrome

### 4.4.1. Non-pharmacological Treatment269

Considering pregnant women with SAP ≥ 140 mmHg or DAP ≥ 90 mmHg hypertensive, the following recommendations are applied:

- Routinely, there is no indication for rest in pregnant women with gestational hypertension syndrome (GHS);^274^
- Physical exercise is recommended for at least 3 days per week, with an average of 50 min per session, including aerobic, strength, and flexibility training;
- Physical activity with moderate exercise may be continued in women who are already accustomed to practice;^112^
- Diet should be healthy, rich in nutrients, proteins, fibers, and cereals;
- Calcium supplementation, between 1.5 and 2.0 g daily, is necessary, especially in areas with low dietary calcium ingestion;
- Weight gain in pregnant women is based on pre-gestational body mass index (BMI):^131^

– BMI of 25 kg/m^2^ (normal): weight gain from 11.2 to 15.9 kg;
– BMI of 25 to 29.9 kg/m^2^(overweight): weight gain from 6.8 to 11.2 kg;
– BMI ≥ 30 kg/m^2^ (obese): weight gain of 6.8 kg.

**The following are not recommended:**

- Any type of low-calorie diet, even in obese women, because low-calorie diets may lead to fetal growth retardation;
- Salt restriction during gestation with the intention of preventing GHS or low sodium (less than 100 mEq daily) diets in pregnant women with chronic arterial hypertension;
- Use of dietary supplements (magnesium; vitamins C, E, and D; fish or algae oil; or garlic) with the goal of preventing GHS.

### 4.4.2. When to Treat – Target Arterial Pressure

In international consensuses, there are points of divergence regarding the beginning of pharmacological treatment for GHS.^131,275-279^This notwithstanding, the prevailing recommendation is to begin oral anti-hypertensive drugs in GHS when SAP is 140 to 155 mmHg and DAP is 90 to 105 mmHg, measured during a consultation, or when arterial pressure is ≥ 135/85 mmHg at home. Specifically, in cases of chronic hypertension, gestational hypertension, or preeclampsia, anti-hypertensive therapy is recommended if SAP is ≥ 140 mmHg or DAP ≥ 90 mmHg.^273,280^

Treatment with anti-hypertensive drugs should maintain arterial pressure at 110 to 140/80 to 85 mmHg, and treatment should be reduced or ceased if DAP is ≤ 80 mmHg. An abrupt drop in maternal arterial pressure, by more than 25% of the initial value, increases the risk of hypoperfusion in maternal target organs and low blood flow to the fetus.

The primary objective of treating hypertension in GHS is to prevent stroke, progression of preexisting kidney disease, or other lesions in target organs, while preserving uteroplacental circulation. Pressure levels should be correlated to the gestational period in course, observing the physiological changes that occur with each gestational trimester,^281^ such as the increased glomerular filtration rate, which interferes in bioavailability of drugs during gestation.^61,282^

In women with chronic hypertension, to date, there is not enough evidence to demonstrate that, by reaching or maintaining a specific (ideal) arterial pressure level or by using a specific anti-hypertensive drug, it is possible to decrease the risk of developing superimposed preeclampsia.^279-282^

The latest systematic review by Cochrane^283^ concluded that data are insufficient to determine the benefits of anti-hypertensive medications for mild to moderate hypertension (SAP from 140 to 169 mmHg and DAP from 90 to 109 mmHg) during gestation; more research is, therefore, necessary. Treatment with anti-hypertensive drugs, however, decreases the risk of severe arterial hypertension, but not of preeclampsia, IUGR, premature placental detachment, or adverse neonatal outcomes.

The international multicenter randomized clinical trial Control of Hypertension in Pregnancy Study (CHIPS) with pregnant women who were non proteinuric and whose hypertension was “non severe” (arterial pressure = 140 to 159/90 to 109 mmHg), demonstrated that “less tight” pressure control, with DAP target of 100 mmHg *versus* “tight” control with DAP target of 85 mmHg showed a correlation with a higher incidence of severe hypertension (arterial pressure ≥ 160/110 mmHg), with preeclampsia, fetal loss, low birth weight, prematurity, and hospitalization in neonatal ICU.^284,285^

### 4.4.3. Oral Anti-hypertensive Drugs- Chronic Hypertension /Gestational Hypertension

All anti-hypertensive medications cross the placental barrier; for this reasons, the use of pharmacological therapy during pregnancy requires risk-benefit analysis with individualized treatment.^278,282^

In Brazil, the available oral medications that are usually used are methyldopa, beta-blockers (except atenolol), hydralazine, and CCB (nifedipine, amlodipine, and verapamil).^275^Initial anti-hypertensive therapy to pregnant women with gestational hypertension or chronic hypertension should be with monotherapy, with first-line drugs,^67-276^ such as methyldopa, CCB, long-acting oral nifedipine, and beta-blockers (except atenolol).

If ideal blood pressure levels are not achieved, the association with second-line oral medications: clonidine, hydralazine and thiazide diuretics should be considered.^271,274^The potential of diuretics to cause depletion of intravascular volume and therefore compromise placental uterine circulation, IUGR or oligohydramnios, is not supported in more recent randomized studies and in a systematic review of diuretics for the prevention of preeclampsia.^71,286-287^

1st line drugs should be considered:

- **Sympathetic nervous system inhibitors (centrally acting alpha-2-adrenergic receptor agonist):** decrease blood pressure by reducing peripheral vascular resistance. They can change the heart rate and output. A-Methyldopa is the best studied antihypertensive drug in pregnancy.^67,68^ However, methyldopa has only a mild antihypertensive effect, with a slow onset of action (3 to 6 h) and with an average duration of 6 hours. to 8 hours. The most common dose-dependent maternal side effects are drowsiness and dry mouth. Dose independent agents include elevated liver enzymes in up to 5% of women and autoimmune hemolytic anemia.^68^ The recommended starting dose is 250 mg, 2 or 3 times a day (maximum dose 3 g/day);
- **Calcium channel blockers (BCC):** oral nifedipine does not appear to be teratogenic.^64-66,81-83,288,289^ Clinical trials demonstrate that blood flow in the umbilical artery is not affected. Maternal side effects with the use of BCC include tachycardia, palpitations, peripheral edema, headaches and facial flushing. Experience with nifedipine has been favorable.^276^ Although not specifically licensed for pregnancy, it is recommended and its use together with labetalol and methyldopa. The maximum daily dose of nifedipine is 120 mg, divided into three or four doses or 30-60 mg once daily (prolonged release).^270-273^ Administration by sublingual route is contraindicated because it determines an unpredictable hypotensive response, excessive autonomic activation and acute myocardial ischemia;
- The exposure to amlodipine in early pregnancy does not appear to be associated with an increased rate of fetal malformations compared to other antihypertensive agents^290,291^and the antihypertensive effect is slow (± 8 hours);
- **Beta-blockers:** none of the beta-blockers have been associated with teratogenicity^76-79^ IUGR and low placental weight have been associated with the use of atenolol.^79,80,271^ The exposure to any beta-blocker is associated with the risk of bradycardia and neonatal hypoglycemia, which can cause sedation, sleep disorders and depression in pregnant women. In the case of propranolol, there are reports of IUGR, bradycardia and neonatal hypoglycemia, especially with high doses (160 mg/day).^81^ Labetalol is not marketed in Brazil.

Drugs of second line are:

- The following are second-line drugs:
- Clonidine shows an exaggerated increase in arterial pressure (rebound effect) when treatment is discontinued abruptly. It has a greater hypotensive effect than methyldopa;
- Hydralazine is predominantly used intravenously for treatment of severe hypertension in preeclampsia;
- Diuretics: the use of diuretic therapy during pregnancy continues to be controversial, mainly due to theoretical concerns regarding reduced maternal plasma volume. Thiazide diuretics may be continued in pregnant women with chronic SAH, provided that they do not promote volume depletion. Chlorothiazide may increase the risk of congenital anomalies and neonatal complications.^276,286^

The following oral anti-hypertensive are contraindicated during gestation:^290^

- ACEI and ARB, which are associated with fetal acute kidney injury and oligohydramnios and which should be suspended before conception;^291^
- Atenolol (beta-blocker), which leads to IUGR and low placental weight;^292,293^
- Spironolactone, which has an antiandrogenic effect during fetal development;^287^
- Chlorothiazide, which may increase the risk of congenital anomalies and neonatal complications.

### 4.4.4 Anti-hypertensive Drugs for Severe Hypertension in Preeclampsia275,276,278,279,298-300

The maternal and fetal prognosis in severe hypertension is correlated **to i**nitial care provided to these pregnant women.^292^Severe hypertension in preeclampsia is when systolic arterial pressure ≥ 160 or diastolic arterial pressure ≥ 110 mmHg; or both during pregnancy, intrapartum or postpartum period.^277^It is an obstetric emergency, and it requires immediate anti-hypertensive treatment. The goal is not to normalize blood pressure, but to reach levels of 140-150 / 90-100 mmHg^277^ or to reduce 15% to 25% of BP.^275^

Severe preclampsia grave is associated with reversible encephalopathy syndrome (PRES) characterized by headache, visual symptoms, impaired consciousness, epileptic crises, and, occasionally, focal neurological defects.^301^

Pregnant women with severe preeclampsia should be attended or transferred to tertiary healthcare centers. Prior to inter-hospital transfer, blood pressure (BP) must be stabilized and other measures initiated, such as magnesium sulfate for eclampsia prophylaxis.^293^It is recommended that magnesium sulfate should be used for the prevention and treatment of seizures in women with gestational hypertension and pre-eclampsia with severe characteristics or imminence of eclampsia. Maternal stabilization should occur before delivery, even in urgent circumstances.

Admission to the ICU should be considered, in accordance with the following criteria: pregnant women with severe preeclampsia (SAP ≥ 160 mmHg and DAP ≥ 110 mmHg), respiratory insufficiency requiring mechanical ventilatory assistance, eclampsia, HELLP syndrome, oliguria, acute pulmonary edema, and neurological complications, such as stroke or PRES.^294^

Endotracheal intubation is another risk in hypertensive emergencies. Induction of general anesthesia and intubation should never be performed without first taking measures to eliminate or minimize the hypertensive response to intubation. Maternal-fetal monitoring must be strict by the medical and nursing staff during treatment. After initial stabilization, the team should monitor BP closely and institute maintenance therapy as needed.

The American College of Obstetricians and Gynecologists^268,269,280^ makes the following recommendations and conclusions:

- Treatment with first-line agents should be immediate or occur as soon as possible within 30 to 60 minutes after confirmed severe hypertension (blood pressure greater than 160/110 mmHg and persistent for 15 minutes) to reduce the risk of maternal stroke . The patient must be positioned in a sitting or semi-reclining position, with the back supported, they must not be repositioned to be reclined or to stand on their side to obtain low blood pressure, as it will provide a false reading of the pressure measurement;^292^
- Maternal and fetal monitoring by a doctor and nursing staff is recommended during the treatment of severe acute onset hypertension;
- After initial stabilization, the team should monitor blood pressure closely and institute maintenance therapy as needed;
- Intravenous labetalol and hydralazine (IV) are considered first-line drugs for the treatment of severe acute onset hypertension in pregnant women and women in the postpartum period;
- Immediate-release oral nifedipine can also be considered as first-line therapy, especially when IV access is not available;
- The use of labetalol IV, hydralazine IV or oral nifedipine of immediate release for the treatment of severe acute onset hypertension in pregnant or postpartum patients does not require cardiac monitoring;
- In the rare circumstances in which immediate release oral labetalol, hydralazine or nifedipine boluses fail to relieve acute onset, severe hypertension and are administered in appropriate successive doses, emergent consultation with an anesthetist, subspecialist in maternal-fetal medicine or subspecialist in intensive care to discuss second-line intervention is recommended;
- Magnesium sulfate is not recommended as an antihypertensive agent, but magnesium sulfate remains the drug of choice for the prophylaxis of seizures in women with severe acute onset hypertension during pregnancy and the postpartum period. The onset of magnesium should not be delayed in the setting of acute severe hypertension; it is recommended regardless of whether the patient has severe gestational hypertension, pre-eclampsia with severe features or eclampsia.

## 4.5. Practice for Hypertensive Emergency in Preeclampsia (PA ≥ 160/110 mmHg)

In the hypertensive emergency, the most effective drugs are nifedipine, hydralazine and labetalol. There may be subtle differences in your security profiles. The evidence is inadequate for other drugs. Medicines for intravenous use are hydralazine and intravenous labetalol (not available in Brazil). Oral nifedipine is now accepted as first-rate. A recent systematic review by Cochrane found no significant differences between these three drugs in the treatment of hypertensive crisis in terms of efficacy or safety between hydralazine and labetalol or between hydralazine and BCC.^277,295-297^

- **Nifedipine:** initial dose of 10-20 mg orally. The onset time of action of oral nifedipine is 5-10 minutes. The dose should be repeated in 20 minutes, if necessary (if blood pressure is> 155/105 mmHg). Maintaining 10-20 mg every 2-6 hours with the maximum daily dose is 120 mg. Repeat medication if blood pressure is> 155/105 mmHg and administer a maximum of three doses. After 20 min of the third dose and the persistence of arterial hypertension, administer a drug of second choice. It should be noted that the tablets should not be chewed and the formulations should not be used sublingually;
- **Hydralazine:** Initial dose of 5 mg intravenously (maximum dose of 45 mg) in bolus, slowly, over 1 to 2 min, repeat, if necessary, 5 mg every 20 minutes (note: The hydralazine ampoule contains 1 ml, in concentration of 20 mg / ml, dilute an ampoule (1 ml) in 19 ml of distilled water, thus obtaining a concentration of 1 mg / ml). The action starts within 10 to 30 minutes and lasts 2 to 4 hours. Parenteral hydralazine may increase the risk of maternal hypotension (systolic BP, 90 mmHg or less);^271^
- In the rare circumstances in which the bolus of labetalol (not available in Brazil), hydralazine or oral nifedipine (retard) administered in appropriate and successive doses does not control blood pressure levels, it is recommended to discuss intervention with drugs considered to be second line;^267^
- **Nitroglycerin** is considered a medication of choice for preeclampsia associated with acute pulmonary edema (intravenous infusion of 5 mg/min, gradually increasing every 3 to 5 min to a maximum dose of 100 mg/min);
- **Sodium nitroprusside** should be considered as a preferential option for controlling arterial pressure in exceptional situations, such as refractory hypertension of severe hypertension with risk of death. Prolonged treatment with sodium nitroprusside is associated with fetal risk sodium nitroprusside is associated with the fetal risk of intoxication by cyanide, a metabolic product of sodium nitroprusside;for this reason, it should be initiated at 0.25 μg/kg/min up to a maximum of 4 μg/kg/min, for no longer than 4 hours of continuous infusion.^275^

## 4.6. Prophylaxis of Seizure in Preeclampsia - Eclampsia and Magnesium Sulfate Therapy293,275, 299-303

Since the publication of the results of results of The Collaborative Eclâmpsia Trial – Maggie Trial,^302^ o magnesium sulfate (MgSO_4_) is the drug of choice when eclampsia is imminent, and it is the only drug that is effective against seizures in preeclampsia.^299^ Randomized clinical trials have demonstrated that it is superior to hydantoin, diazepam, and placebo for preventing eclampsia and recurrence of seizuresn, in addition its low cost, easy to administer and does not cause sedation.^300-303^ Therefore, the use of magnesium sulfate is highly recommended for cases of imminent eclampsia, HELLP syndrome (15% of these patients develop eclampsia) and pre -eclampsia with clinical and/or laboratory deterioration, including difficult-to-control hypertension.^303^

The initial dose, properly administered, does not pose a risk of intoxication. However, it is recommended to monitor the patellar reflex, respiratory rate and diuresis. If there is no patellar reflex, respiratory depression (respiratory rate < 16 rpm) and diuresis below 25 ml/h, it is recommended to stoped MgSO4 intravenous and measure serum levels.

The therapeutic concentration of the magnesium ion varies from 4 to 7 mEq/L (4.8 to 8.4 mg/dl). The patellar reflex is abolished with 8 to 10 mEq/L, the risk of respiratory arrest starting at 12 mEq/L and cardiac arrest of 25 mEq/L. Calcium gluconate (1 g intravenously – 10 ml at 10% – administered slowly) should be used in cases of signs of magnesium intoxication. In respiratory arrest, in addition to calcium gluconate, endotracheal intubation and mechanical ventilation should be performed. In patients with renal impairment (creatinine ≥ 1.2 mg/dl), the maintenance dose should be half the recommended dose. Magnesium sulfate infusion should be stopped only if diuresis is less than 25 ml. In view of values within normal limits, treatment should be maintained or restarted.^304^

The prevention of convulsive crises in preeclampsia is guided by the following recommendations:

- **Loading dose:** (MgSO4 50% – ampoule with 10ml – contains 5 g de magnésio) – 4 to 6 g of MgSO_4_, intravenous, in a single dose (dilute 8 to 12 ml of 50% solution in 100 ml of 5% glucose solution and administer, with an infusion pump, for 30 minutes);
- **Maintenance dose:** 1 to 2 g per hour, intravenous (dilute 10 ml of MgSO4 50% (1 ampoule) in 490 ml of 0,9% of saline solution. The final concentration will be 1 g/100 ml. Infuse the solution intravenously at a rate of 100 ml per hour in a continuous infusion pump.

It is necessary to maintain the MgSO4 for 24 hours after delivery or the last seizure. In cases of recurrence of the seizure, an additional 2 g of magnesium sulfate is administered intravenously (bolus) and the dose of 2 g/h is used as maintenance. If two of these boluses do not control seizures, the drug of choice will be diphenylhydantoin in its classic regimen for treating seizures. In these cases, the investigation of brain complications, especially intracranial hemorrhages, is recommended.

After the first 24 hours of observation and evaluation, it is necessary to decide on conservative conduct or termination of pregnancy. Childbirth is the only intervention that leads to the resolution of pre-eclampsia and eclampsia. It is recommended that the expectant conduct is only until 37 weeks of gestation. After this gestational date or if the diagnosis of pre-eclampsia is performed at term, the resolution of the pregnancy should be indicated, thus reducing maternal risks, without altering the perinatal results.

### 4.6.1 Key Points

- In women with gestational hypertension, pre-existing hypertension overlapping with gestational hypertension or with damage or symptoms of hypertension and subclinical organs, initiation of drug treatment is recommended when SBP ≥ 140 mmHg or DBP ≥ 90 mmHg;
- A goal treatment for blood pressure in SHG should be 140/80 to 85 mmHg. DBP to ≤ 80 mmHg, antihypertensive drugs should be reduced or discontinued;
- Methyldopa, beta-blockers (except atenolol) and calcium channel blockers are recommended as the drugs of choice for the treatment of hypertension in pregnancy;
- ACE inhibitors, ARBs or direct renin inhibitors are not recommended during pregnancy;
- Diuretic therapy is usually avoided because plasma volume is reduced in women who develop preeclampsia;
- Considers SBP ≥ 170 mmHg or DBP ≥ 110 mmHg to be an emergency in a pregnant woman who should be admitted to hospital immediately for treatment; The consensus is to reduce BP to < 160/105 mmHg to avoid acute hypertensive complications in the mother; fetal heart rate monitoring;
- Magnesium sulphate should be used to prevent and treat seizures in women with gestational hypertension and preeclampsia with severe or imminent eclampsia;
- In a hypertensive emergency, the most effective drugs are nifedipine, hydralazine and labetalol (not available in Brazil);
- In preeclampsia associated with pulmonary edema, nitroglycerin administered as i.v. infusion is recommended;
- The delivery is a single intervention that leads to resolution of preeclampsia and eclampsia.

## 4.7. Prognosis and Prevention of Preeclampsia

Clinical prediction models based on risk factors have low sensitivity, and they generally do not include a large number of pregnant women who might develop preeclampsia during the course of gestation. The following biochemical markers stand out: placental growth factor (PlGF), which is proangiogenic and, when its levels are low between weeks 11 and 13, and soluble FMS-like tyrosine kinase 1 (sFlt-1), which is antiangiogenic and which, when its levels are high, may predict preeclampsia. As neither of them have sufficient sensitivity, the relationship between both factors (sFlt-1/PIGF) is currently being studied, with more promising results. There is, at the moment, however, no predictive laboratory test available in clinical practice.^304^

It is also possible to utilize Doppler ultrasound as an auxiliary tool. By evaluating pulsatility and resistance in uterine arteries, it may classify pregnant women by risk of developing preeclampsia. Doppler ultrasound should be performed between weeks 20 and 22, although there is a good correlation between late preeclampsia (> 34 weeks) and IUGR. In contrast, Doppler ultrasound performed at the end of the first trimester has lower accuracy; nonetheless, in conjunction with clinical history and comorbidities, it may be useful for identifying pregnant women with higher risks and selecting those who will require prophylaxis for preeclampsia.^305^

Diverse substances have been tested to reduce the incidence of preeclampsia. Studies on diet, weight loss, physical activity, vitamins, antioxidants, nitrates, dipyridamole, heparins (LMWH and UFH), and antiplatelet agents have been conducted; of these, only calcium replacement and acetylsalicylic acid (ASA) have shown some benefit.

Calcium replacement (1.5 to 2.0 g daily) reduces the risk of preeclampsia effectively only in the subpopulation with calcium ingestion below 600 mg daily.^306^

Studies have shown the benefits of ASA in low doses (between 75 and 150 mg) for preeclampsia prevention,^307^ and it has recently been included in the recommendations of important international guidelines.^278,308,269^ Study^309^ with 1,776 patients, using 150-mg doses of ASA *versus* placebo, starting between weeks 11 and 14, demonstrated that the total of preeclampsia events was significantly reduced in the ASA group compared to placebo group, reinforcing the protective effect of ASA in pregnant women with high risks.

The precise indication of ASA is for patients classified as high-risk for preeclampsia (Table 27), and it should be initiated between weeks 12 and 16.

**Table 27:** – Recommendations for acetylsalicylic acid use in preeclampsia prophylaxis

Risk level	Risk factor	Recommendation
High	Preeclampsia with adverse fetal outcomes Multifetal gestation Chronic SAH Diabetes mellitus type 1 or 2 Kidney disease Autoimmune disease (lupus/APAS)	A low dose of ASA is recommended if the patient meets one or more of these criteria
Moderate	Nulliparity Obesity (BMI ≥ 30) Family history of preeclampsia (mother or sister) Age ≥ 35 years Poor previous obstetric history (SGA, prematurity, low birth weight, an interval of more than 10 years between gestations)	Consider using a low dose of ASA if the patient has more than one risk factor

*APAS: antiphospholipid antibody syndrome; ASA: acetylsalicylic acid; BMI: body mass index; SAH: systemic arterial hypertension; SGA: small for gestational age.*

### 4.7.1. Key Points

- Predicting preeclampsia in low-risk patients is difficult and depends on joint assessment of clinical history and Doppler US;
- Calcium replacement in patients with low intake reduces the risk of preeclampsia;
- The use of low dose AAS in moderate to high risk pregnant women reduces the risk of preeclampsia and should ideally be started between 12 and 16 weeks.

## 4.8. Arterial Hypertension during the Postpartum Period

Arterial hypertension during the postpartum period has been little studied, because there is still a belief that once the placenta has been removed the problem is solved. To a certain extent, placental delivery marks the moment when the stimulation of the production of inflammatory and vasoconstrictive substances ceases, leading to a gradual return in arterial pressure to pre-gestation levels; nevertheless, some of these inflammatory and vasoconstrictive alterations may remain in the maternal organism for a few days.

### 4.8.1. Recommendations

Hypertension normally improves within the first week (5 to 7 days); however, during this period, there continues to be a risk of related complications, especially in patients with preeclampsia, in addition to the possibility of preeclampsia itself manifesting only during the postpartum period. There is also a risk of eclampsia during this period, and 32% to 44% of convulsions may occur during the postpartum period.

Hypertension during the postpartum period may be aggravated or prolonged by situations such as volume overload (hydration) and use of pain medication, such as non-steroidal anti-inflammatory drugs (vasoconstriction and sodium retention), in addition to cases of stroke with reactive vasoconstriction and in patients with previously undiagnosed chronic hypertension.

In postpartum women with preeclampsia, a new elevation in arterial pressure may occur between 3 and 6 days postpartum, probably due to reabsorption of accumulated edema in the third space, which is a rather common syndrome of preeclampsia.^310^

The treatment objective is to decrease the risk of target organ injury due to hypertensive emergency (acute pulmonary edema, stroke, dissection of the aorta, acute kidney disease). Thus, postpartum women with mild to moderate hypertension (SAP < 160 mmHg and/or DAP < 110 mmHg), who are asymptomatic, may receive follow up without anti-hypertensive medication.

Women may receive any anti-hypertensive medication during the postpartum period. The factor that limits use is breastfeeding; thus, preference should be given to anti-hypertensive medications which pass through breast milk in lower quantities.

In 2013, a review from the Cochrane Library^311^ suggested that the use of furosemide might assist in more effective control and shorten hospitalization time in patients with preeclampsia. The review recommends that each service use its routine medication without giving preference to any class of anti-hypertensive drug. Diuretics should become part of the anti-hypertensive regime after the second day, when the reabsorption of peripheral edema begins. The consultation site https://toxnet.nlm.nih.gov, reviews publications and updates recommendations for use of medication while breastfeeding.^312^

The ACEI captopril and enalapril, which are contraindicated during gestation, are permitted during breastfeeding, as they pass through breast milk in very small quantities. Regarding ARB group II, there is not a sufficient number of studies for liberating the use of this class of medication. The most utilized CCB is nifedipine, which also passes through breast milk in small quantities. Amlodipine and other CCB, such as ARB, lack studies to liberate them without restrictions. Beta-blockers should be individualized on a case-by-base basis. Propranolol and metoprolol are compatible with breastfeeding, whereas atenolol should be avoided.

Diuretic drugs, such as hydrochlorothiazide and furosemide, may deplete intravascular space and decrease milk production; for this reason, they should be used in low doses. Spironolactone may be administered without restriction, and it may be used in patients with resistant hypertension (primary hyperaldosteronism).

Treatment of hypertensive peaks in postpartum women may be done conventionally. A study comparing captopril and clonidine for controlling hypertension (SAP ≥ 180 mmHg and DAP ≥ 110 mmHg) verified that there was no significant difference between the substances, only a tendency for clonidine to be better during the third day of the postpartum period. Both were considered effective and safe for treating postpartum women with hypertensive emergencies.^313^

We recommend hospital discharge after at least 24 hours in cases of SAP < 160 mmHg and DAP < 110 mmHg. After that they should receive close outpatient follow up, with brief reevaluation 1 to 2 weeks after discharge.^314^

### 4.8.2. Key Points

- Hypertension usually improves in the first five to seven days, but after this period there is still a risk of complications, including preeclampsia/eclampsia;
- Priority should be given to medications low-releasing for breastfeeding;
- Outpatient follow-up is important as most of these patients leave the hospital still on medication.

## 4.9. Hypertension During Gestation and Future Cardiovascular Risk

Preeclampsia is an established risk factor for coronary artery disease, chronic hypertension, peripheral vascular disease, and stroke. Possible mechanisms behind the increase in cardiovascular disease include endothelial, vascular, and metabolic dysfunctions found during preeclampsia, which have a common link to other traditional risk factors, such as dyslipidemia, obesity, diabetes mellitus, and kidney disease.

The CHAMPS Study,^315^conducted retrospectively with more than one million women with cardiovascular disease after their first gestation, showed an increase in the risk of myocardial revascularization and hospitalization due to cardiovascular disease, stroke, and peripheral arterial vascular disease; this risk was 2 times higher in patients who had had preeclampsia, gestational hypertension, placental rupture, or infarction.

Another large review^316^including more than 3 million women and nearly 200,000 pregnant women showed increased relative risks of 3.7 for chronic SAH, 2.16 for ischemic heart disease and 1.81 for stroke after 10.4 years, in women whose had preeclampsia.

In this manner, hypertension during gestation should be seen as a sex-related marker of future cardiovascular risk. Furthermore, although it is not one of the main factors used for calculating cardiovascular risk, it is necessary, as part of clinical routine, to include precautions when counseling women after delivery and to intensify control of other modifiable factors with the aim of decreasing their cardiovascular risks.^317^

### 4.9.1. Key Points

- Preeclampsia is a risk factor for coronary artery disease, chronic hypertension, peripheral vascular disease and stroke;
- Patients who have high blood pressure during pregnancy should intensify control of other modifiable factors to reduce future cardiovascular risk.

## 5. Treatment and Prevention of Cardiac Complications

### 5.1. Cardiac Arrhythmias

#### 5.1.1. Epidemiology

Arrhythmias are very frequent complications during pregnancy, whether or not they are associated with structural or electrical heart disease. The first manifestation may occur during gestation, or an aggravation of preexisting arrhythmias may occur.^318^

The occurrence of arrhythmias during gestation requires investigation with special attention to definition or exclusion of structural or electric cardiac injury; this practice is fundamental to determining treatment and prognosis for the patient.^52,318^

A study in hospitalized pregnant women has shown that: 60% of arrhythmias correspond to sinus bradycardia or tachycardia; 19% to supraventricular or ventricular extrasystoles; 14% to supraventricular tachycardia (SVT); 5% to VT or VF; and 2% to other disorders.^319^

AF and paroxysmal supraventricular tachycardia (PSVT) are the most frequently diagnosed sustained SVT during gestation; bradyarrhythmias, conduction disorders, other atrial tachycardias, VT, and VF are relatively rare.^320^

The accepted risks of antiarrhythmic drugs affecting organogenesis and fetal development should be considered during pregnancy, given that most diagnosed arrhythmias do not require specific treatment. Nevertheless, recurring or persistent arrhythmias that cause important symptoms or hemodynamic repercussion should be treated in the same manner they would be for non-pregnant women.^321^

The risks inherent to ionizing radiation used to perform catheter ablation may by minimized with electromechanical mapping and, in some cases of device implantation (pacemaker, ICD, and resynchronizer), with the use of 2-dimensional echo.^322^

##### 5.1.2. Clinical Presentation

Palpitations occur frequently during pregnancy. They may be related to arrhythmias, or they may be consequent to hemodynamic alterations during gestation. Diagnostic evaluation of palpitations in pregnant women does not differ from diagnosis in non-pregnant women, and it has been demonstrated that palpitations are associated with the presence of arrhythmias in only 10% of cases.^323^

Symptomatic sinus bradycardia is rare, and it is generally associated with gestational supine hypotensive syndrome, which is treated by placing pregnant patients in left lateral decubitus. Syncope linked to atrioventricular blocks is, similarly, infrequent, and congenital complete atrioventricular block, especially supra-hisian, with narrow QRS, presents favorable evolution during gestation. Sudden Cardiac Death (SCD), which is rare during gestation, presents a greater risk of occurring in women with VT associated with structural heart disease, and, during gestation and the postpartum period, in women with channelopathies (especially women with long-QT syndrome).^319,320^

##### 5.1.3. Maternal-fetal Risks

Sustained cardiac rhythm disorders may lead to maternal hemodynamic impairment, the risk of thromboembolism, and SCD. They may also compromise fetal development, leading to low birth weight, premature delivery, fetal abnormalities, and other indications for cesarean delivery. For this reason, these disorders should be diagnosed and adequately treated.

The modified WHO classification for maternal risk considers isolated supraventricular and ventricular extrasystoles as class I (in which there is no detectable risk of increased maternal mortality, but there is a mild increase in maternal morbidity); supraventricular arrhythmias are in class II (in which there is a mild increase in maternal mortality and a moderate increase in maternal morbidity), and VT are included in class III (in which there is a significant increase in maternal mortality and morbidity).^324^

Current recommendations suggest that arrhythmias be classified during gestation, in accordance with potential hemodynamic impairment, as the following: low-risk of SCD (PSVT and AF with hemodynamic stability, idiopathic VT, low-risk long QT syndrome, and Wolff-Parkinson-White syndrome); medium-risk of SCD (unstable SVT, VT in patients with structural heart disease, Brugada syndrome, long QT syndrome, and moderate-risk catecholaminergic polymorphic VT); high-risk of SCD (unstable VT in patients with structural heart disease, *torsades de pointes* in patients with long QT syndrome, short QT syndrome, and high-risk catecholaminergic polymorphic VT).^52,320^

For the low-risk group, a cardiologist should participate in delivery planning, and delivery should be indicated by the obstetrician. In the medium-risk group, delivery continues to be indicated by the obstetrician; nevertheless, the multidisciplinary team that accompanies the pregnant patient should include an electrophysiologist, and, during delivery, the team should be prepared to use drugs such as adenosine and beta-blockers, as well as cardioverter-defibrillator (CD). In the high-risk group, there is an indication for cesarean delivery, during which it is necessary to be prepared to use CD and antiarrhythmic drugs, in addition to beta-blockers; patients in this group may require admission to the ICU during the postpartum period.^52^

##### 5.1.4. Treatment

Treatment of arrhythmias in pregnant women is similar to that in non-pregnant women.^325^ According to indication, the following methods may be used: electrical cardioversion, vagal maneuvers, antiarrhythmic drugs, device implantation (pacemaker, ICD, and cardiac resynchronizer), and catheter ablation (Table 28). Treatment of cardiac arrhythmias in the emergency room will be discussed in section 5.7.

**Table 28:** – Practice for acute supraventricular tachycardia

Recommendation
Immediate electrical cardioversion as a first choice for SVT with maternal hemodynamic instability and AF in pregnant women with ventricular pre-excitation syndrome
Vagal maneuvers; in the event that they are inefficient, adenosine for acute reversion of PSVT
Endovenous beta-blockers (metoprolol, propranolol) for acute reversion of PSVT
Endovenous verapamil for acute reversion of PSVT when adenosine and beta-blockers are not effective, or when they are contraindicated
Endovenous procainamide for acute reversion of SVT
Flecainide or ibutilide for acute reversion of flutter and AF in pregnant women with structurally normal hearts
Amiodarone for acute reversion of potentially severe SVT when other therapies are not effective, or when they are contraindicated

*AF: atrial fibrillation; PSVT: paroxysmal supraventricular tachycardia; SVT: supraventricular tachycardia.*

Due to a lack of randomized clinical trials, the indication or contraindication of a given method is based on experimental data from animal studies, registries of side effects of medications used in clinical practice, and case reports or case series. This means that these treatments should only be used when there is maternal and fetal hemodynamic impairment as a result of arrhythmia and/or when there is a risk of maternal SCD during pregnancy and the postpartum period. Whenever possible, all treatments should be postponed to the second or third trimester (thus avoiding the organogenesis period); in the event that medications are used, it is necessary to utilize the lowest dose for the shortest time necessary.

Synchronized electrical cardioversion, which is indicated for reversion of unstable SVT (AF, atrial flutter, atrial tachycardias, PSVT), and unstable or stable VT in the presence of heart disease, is safe during all phases of gestation; it does not compromise fetal blood flow. The pads should be placed in the anterolateral position, with the lateral pad below the mother’s left breast and fetal rhythm monitoring.^326^

During gestation, vagal maneuvers, such as the Valsalva maneuver, carotid sinus massage, immersing the face in 10ºC water, placing a wet towel on the face, may be used safely for acute reversion of PSVT (caused by nodal reentry or by an accessory route, the latter being characteristic of Wolff-Parkinson-White syndrome).^52,325^ The Valsalva maneuver is typically more effective than carotid sinus massage. Eyeball compression is potentially dangerous and should never be used.

When vagal maneuvers fail in the attempt at acute reversion of PSVT, adenosine (6 mg initially; maximum dose of 24 mg) is the drug of first choice for pregnant women, because there is no evidence of negative effects on the fetus, and the maternal effects (chest discomfort and flushing) have short duration.^52,325,327^ Even though they are not first-choice drugs, beta-blockers (metoprolol, propranolol), verapamil, procainamide, and amiodarone may also be used in the attempt at reversion.

In acute management of other sustained supraventricular arrhythmias (AF, flutter, atrial tachycardia), beta-blockers, verapamil and digitalis drugs are indicated for controlling ventricular response, and other drugs, including flecainide, ibutilide, and propafenone, may be used for acute reversion to sinus rhythm.^52,325,327^ For reversion to sinus rhythm in stable idiopathic VT, beta-blockers, sotalol, flecainide, procainamide, lidocaine are indicated. For SVT, overdrive ventricular pacing is an alternative that should be considered (Table 29).

**Table 29:** – Practice for chronic supraventricular tachycardia

Recommendation
Beta-blockers or verapamil to prevent PSVT in pregnant women without pre-excitation on ECG
Beta-blockers for controlling ventricular response in pregnant women with AF or atrial tachycardia
Flecainide or propafenone for preventing PSVT in patients with Wolff-Parkinson-White syndrome
Flecainide, propafenone, or sotalol syndrome for preventing PSVT, atrial tachycardia, and AF when there is no response to beta-blockers
Digoxin or verapamil for controlling heart rate in atrial tachycardia and AF when there is no response to beta-blockers
Catheter ablation with the use of electroanatomical mapping systems for SVT that are not well tolerated or refractory to treatment with antiarrhythmic drugs

*ECG: electrocardiography; AF: atrial fibrillation; PSVT: paroxysmal supraventricular tachycardia; SVT: supraventricular tachycardia.*

Permanent treatment of SVT and VT should be the same as that applied to non-pregnant women, with the exception of restrictions to the use of amiodarone due to fetal implications (hypothyroidism, hyperthyroidism, growth retardation, and prematurity). It should also be considered that bradycardia, fetal hypoglycemia, and low birth weight might be associated with the chronic use of beta-blockers; nevertheless, this fact appears to be dose-dependent. Prescription of beta-blockers should contemplate the benefits, which should exceed the risks; the exception is atenolol, which has recognized teratogenic effects and should, therefore, be avoided during gestation. There are also reports of teratogenicity with the use of diltiazem. Sotalol should not be permanently used in pregnant women with Wolff-Parkinson-White syndrome to prevent episodes of PSVT (Tables 30 and 31).^52,325,327^

**Table 30:** – Practice for acute ventricular tachycardia

Recommendation
Immediate electrical cardioversion as a first choice for pregnant women with sustained VT, with or without hemodynamic instability
Beta-blockers, sotalol, flecainide, procainamide, or overdrive ventricular pacing for reversion of hemodynamically stable, idiopathic, monomorphic sustained VT

*VT: ventricular tachycardia.*

**Table 31:** – Practice for chronic ventricular tachycardia

Recommendation
Beta-blockers in pregnant women with long QT syndrome and catecholaminergic polymorphic VT during gestation and the postpartum period, including those who are breastfeeding
ICD implantation should be performed before gestation; in the event that this is indicated during gestation, it should be performed using minimal radiation (guided by echocardiogram, for example) and, preferably, after the first trimester
Beta-blockers or verapamil for preventing episodes of idiopathic sustained VT
Sotalol or flecainide for preventing episodes of idiopathic sustained VT, if other substances are not effective
Catheter ablation, with the use of electroanatomical mapping systems, for sustained VT that are not well tolerated or refractory to treatment with antiarrhythmic drugs

*ICD: implantable cardioverter-defibrillator; VT: ventricular tachycardia.*

In general, catheter ablation and device implantation, whenever possible, should be performed outside of the gestational period, due to the risks inherent to these procedures, including the risk related to exposure to ionizing radiation. Catheter ablation during gestation has been indicated only for pregnant women who present recurring or persistent severe tachycardias with severe hemodynamic impairment and who do not respond to the usual treatments. There are case reports and small case series of patients with SVT who underwent catheter ablation with the use of mapping strategies that use increasingly smaller amounts of ionizing radiation, thus increasing maternal and fetal safety regarding the future risks of this exposure.^328^ There are no reports of catheter ablation for VT to date.

Women with pacemakers and ICD show positive evolution during gestation; this notwithstanding, the complications inherent to underlying heart disease and devices appear to be present, leading to the need for specialized care.^329^ In the event that they are absolutely indispensable, these devices may be implanted safely during gestation with or without minimal fluoroscopy.^330^

Devices (pacemakers and ICD) should be reprogrammed before cesarean delivery, due to functional interference caused by the electric scalpel. In the event of emergency cesarean delivery, a magnet is placed over the pacemaker generator pocket while the electric scalpel is in use, and the cautery plate is placed far away from the thoracic region. For vaginal delivery, reprogramming is not necessary.

For pregnant women with chronic AF or atrial flutter that are not associated with structural heart disease risk stratification should be performed for thromboembolic phenomena, by means of the CHA_2_DS_2_-VASc risk score,^331^ including indication for anticoagulation when the score is ≥ 2. It is controversial whether the state of hypercoagulability increases the risk score for indication of anticoagulants during gestation. It is necessary to emphasize that new oral anticoagulants (dabigatran, rivaroxaban, apixaban, and edoxaban) should not be used in pregnant women.^332,333^

##### 5.1.5. Key Points

- Initial practice for arrhythmias during pregnancy is to investigate structural cardiac injury;
- “New” arrhythmias, in the absence of structural cardiac injury, should be treated according to maternal symptoms or the complexity of the arrhythmia;
- A 24-hour Holter monitor examination is essential to therapeutic decision making;
- Device implantation (pacemaker, ICD) and radiofrequency ablation with electroanatomical mapping are safe during pregnancy, and they should be indicated when a case is refractory to pharmacological treatment;
- Devices such as pacemakers, ICD, and cardiac resynchronizers should be reprogrammed after cesarean delivery.

## 5.2. Thromboembolism

### 5.2.1. Epidemiology

Venous thromboembolic events are important causes of maternal mortality and they are potentially preventable.^131,334^ They are the main direct cause of maternal death in developed countries and in Brazil; in 2013,^335^ they were the sixth leading cause, behind severe hemorrhage, hypertension during gestation, infection, delivery complications, and abortion. Furthermore, they are a relevant cause of morbidity due to post-thrombotic syndrome. Late diagnosis, delayed or inadequate treatment, and inappropriate prophylaxis are responsible for approximately 3.5% of maternal deaths.^336^

Thromboembolism includes both deep vein thrombosis (DVT) and PTE; 75% to 80% of cases of pregnancy-associated thromboembolism are DVT, and 20% to 25% are PTE. The real incidence of the disease associated with gestation is unknown, but it appears to be between 7 and 25 cases per 10,000 pregnancies, and the clinical impression is that chances are increased 5- to 10-fold during this period. The risk appears to be greater during the third trimester, but it is elevated since the first. During the postpartum period, the risk reaches 20 times that of non-pregnant women, and it decreases gradually until 6 weeks postpartum. Nonetheless, recent studies have shown an increase in the risk of thromboembolism for up to 180 days postpartum in patients with some obstetric risk factors, including cesarean delivery and twin gestation.^131,334,335^

### 5.2.2. Risk Factors

The Table 32 lists preexisting, transitory, and obstetric risk factors associated with thromboembolism during gestation. It has been suggested that the presence of 2 or more of these factors further increases the risk of disease; prior history of thrombosis, however, is the most important individual risk factor. The recurrence of thrombosis during this period increases 3- to 4-fold, accounting for 15% to 25% of all cases of thromboembolism during gestation.^336,337^

**Table 32:** – Risk factors for venous thromboembolism during gestation

Preexisting factors	Transitory factors	Obstetric factors
1. Prior thromboembolism	1. Gestation	**Prenatal:**
2. Thrombophilias	2. Hyperemesis gravidarum	1. Assisted reproduction
3. Family history of thromboembolism	3. Dehydration	2. Multiple pregnancy
4. Comorbidities: SLE, nephrotic syndrome, drepanocytosis, cancer, paraplegia	4. Ovarian hyperstimulation syndrome	3. Preeclampsia
5. Diabetes mellitus	5. Infection	**Delivery:**
6. Inflammatory diseases (especially intestinal)	7. Immobility	1. Prolonged labor
7. Over 35 years of age	8. More than 4 hours of travel	**Surgical:**
8. Obesity		2. Cesarean delivery, Postpartum sterilization
9. Tobacco use		3. Stillbirth
10. Lower limb varicose veins		4. Forceps
11. Parity ≥ 3		**Postpartum:**
12. History of stillbirth		1. Postpartum hemorrhage
13. Pre-term delivery		2. Blood transfusion

*SLE: systemic lupus erythematosus.*

### 5.2.3. Thrombophilia

Thrombophilia comprises a state of congenital or acquired hypercoagulability. This issue, when isolated, even in the context of pregnancy, does not necessarily result in the occurrence of thromboembolism;^338^the rarity of thromboembolism during pregnancy and the high incidence of hereditary thrombophilias do not justify systematic tracking of this disease.

Venous thrombosis is a polygenic disease with incomplete penetration, which makes genetic counseling uncertain. The risk of thromboembolism associated with different thrombophilias and its prevalence in the general population are shown in Table 33.

**Table 33:** – Risk of venous thromboembolism associated with different thrombophilias

Factor	Prevalence in the general population (%)	Risk during pregnancy (%) (with no prior history)	Risk during pregnancy (%) (with prior history)	Percentage of all thromboembolisms
Factor V Leiden heterozygote	1 to 15	0.5 to 3.1	10	40
Factor V Leiden homozygote	< 1	2.2 to 14	17	2
G20210A heterozygote	2 to 5	0.4 to 2.6	> 10	17
G20210A homozygote	< 1	2.0 to 4.0	> 17	0.5
Factor V Leiden/G20210A heterozygote	0.01	4.0 to 8.2	> 20	1 to 3
Antithrombin deficiency	0.02	0.2 to 11.6	40	1
Protein C deficiency	0.2 to 0.4	0.1 to 1.7	4 to 17	14
Protein S deficiency	0.03 to 0.13	0.3 to 6.6	0 to 22	3

*G20210A: mutation of the prothrombin gene.*

There is limited value to tracking thrombophilias in pregnant women with acute thromboembolism, because it does not modify clinical practice. For this reason, investigation of thrombophilia is recommended during gestation in the following situations,^339^ with the following classes of evidence:

- Based on clinical risk (class IB);
- Family history (first-degree relatives) of thromboembolism without a detectable cause or occurring during hormonal exposure, or a minor risk factor, or still under the age of 50 should be investigated (class IIC);
- Thromboembolism with lower transitory risk factor, such as travel time (class IIC).

Investigation of thrombophilia is not recommended in the following situations:

- Prior thromboembolism without an apparent cause (class IB) and thromboembolism related to hormone use or during a previous gestation (class IIC) require indication of tromboprophylaxis;
- Personal history of the disease with a major transitory risk factor (fracture, surgery, prolonged immobility) (class IIB);
- Obstetric history of recurring fetal loss, placenta praevia, IUGR, and preeclampsia.

### 5.2.4. Diagnosis

Final diagnosis may be compromised by signs and symptoms which are inherent to normal pregnancy, such as edema, pain in lower limbs, chest pain, precordial palpitation, and dyspnea. Nevertheless, clinical evaluation is the essential basis for seeking conclusive diagnosis, because there is still not a single screening test that is sufficiently sensitive to define the situation. Furthermore, most studies that evaluate diagnostic imaging examinations for thromboembolism and flowcharts for diagnosis exclude pregnant women due to a concern for maternal-fetal safety.

## 5.2.4.1. Deep Vein Thrombosis

Diagnosis based on clinical picture (anamnesis and clinical examination) is concerning, because it determines whether or not the patients will require permanent anticoagulant therapy during gestation. This situation requires subsidiary examinations in order to conclude diagnosis, which should be expedited, given that sudden death is not uncommon in pregnant women with signs and symptoms compatible with this disease.

Structured risk scores for classifying pregnant women as low-, intermediate-, or high-risk for DVT, such as the Wells’ score, have not been validated for use during gestation. The LEFT rule on the other hand has been proposed for specific prediction of the chance of DVT during pregnancy, and it appears to be promising. If none of its variables are present, the negative predictive value appears to be 100% but this method still needs to be validated by larger prospective studies.^340,341^

- The variables considered by risk scores for DVT are the following:
- Presentation of thrombosis in the left leg;
- Difference of ≥ 2 cm in calf circumference (edema);
- Presentation during the first trimester of pregnancy.

Table 34 lists the complementary examinations used for diagnosing DVT, their sensitivity, specificity, advantages, and disadvantages.

**Table 34:** – Examinations used for diagnosing deep vein thrombosis

Examinations	Accuracy	Advantages	Disadvantages
Physical Examination	Se - 25% to 35% Sp - 30% to 50%	Harmless, may suggest other diagnoses	None
D-dimer dosage	Se - 100% Sp - 60%	Excellent negative predictive value**	Must be associated with ultrasound
Compression ultrasound/duplex scan	Se - 96% for proximal veins Sp - 98%	Low cost Easily repeated	None
MR angiography	Se - 91.5%* Sp - 94.8%*	Pelvic and iliac vein thrombosis	Cost
Venous CT angiography	Se - 95.5%* Sp - 95.2%*	It may be performed in conjunction with pulmonary CT angiography	Cost Use of contrast Radiation

CT: computed tomography; MR: magnetic resonance; Se: sensitivity; Sp: specificity. * Data from meta-analysis of largely heterogeneous studies. ** Not validated for gestation.

***5.2.4.2. D-dimer***

D-dimer dosage is present in the classical algorithm for diagnosing thromboembolism; during pregnancy, however, this marker loses its accuracy for diagnosing PTE, given that it undergoes an increase of approximately 40% during all trimesters, the postpartum period, and complications such as preeclampsia and placenta abruption.^342^ These uncertainties influence the disagreement regarding use of D-dimer in the algorithm for diagnosing thromboembolism during gestation.^336,340,343^

***5.2.4.3. Venous Ultrasound***

A practical approach to suspected DVT begins with the use of compression ultrasound in the affected limb. Analysis of vein compressibility on this examination presents a sensitivity of 96% and a specificity of 98% for diagnosis of DVT above the knee; this is slightly lower for those beneath the knee, although there is a substantial chance of diagnosis in these as well. Knowledge of the fact that DVT frequently presents in proximal veins, but that it may be isolated in iliac veins may limit the ability to exclude DVT with compression ultrasound alone in symptomatic pregnant women. Given that compression maneuvers may not be performed in iliac veins, iliac vein thrombi are diagnosed by direct visualization of intraluminal echogenic mass or absence of spontaneous venous flow on Doppler.

If ultrasound is positive, diagnosis is confirmed, and treatment is initiated immediately. In the event that it is negative and the patient continues to present symptoms, the examination should be repeated every 3 to 7 days, and treatment should be initiated if diagnosis is confirmed. Figure 9 shows 2 flowcharts for diagnosis of DVT during gestation: a venous compression ultrasound starting with the femoral veins and the use of D-dimer to evaluate the need for investigation of the iliac region; and complete venous ultrasound in the leg, including evaluation of the iliac vein.

**Figure 9:** – *Flowchart used for investigating deep vein thrombosis during gestation. DVT: deep vein thrombosis; MR: magnetic resonance; MRA: magnetic resonance angiography; US: ultrasound.*

***5.2.4.4. Iliac Vein Magnetic Resonance***

When the clinical picture of isolated iliac thrombosis arises (whole limb edema, with or without pain in the flanks, buttocks, or lumbar regions), ultrasound does not resolve the situation well, and magnetic resonance should be used. Magnetic resonance may be used to diagnose DVT involving iliac veins during pregnancy, but it depends on the examiner’s expertise.^336,340,341^

***5.2.4.5. Pulmonary Thromboembolism***

Currently, approach to diagnosis of PTE during gestation is uncertain, and further studies are required. Approximately seven guidelines consider diagnosis of PTE during gestation, and the orientations regarding the use of rules for predicting risk, using D-dimer dosage, and choosing imaging methods diverge. Most of the guidelines do not include D-dimer dosage in the diagnostic algorithm for PTE. In relation to ultrasound, some guidelines initially use investigation for diagnosis of DVT; its positivity, however, is only 20% to 40% for PTE, and, if it is negative, diagnosis has to be confirmed by other imaging methods.

Examinations of choice for diagnosing PTE are pulmonary V/Q scintigraphy or CTPA; both tests, however, carry the risk of maternal and fetal exposure to radiation. Pulmonary V/Q scintigraphy exposes the fetus to a greater radiation dose than CTPA; thus, if chest X-ray is normal, only perfusion scintigraphy is considered, therefore reducing the radiation dose. V/Q scintigraphy also exposes the child to a greater risk of neoplasm, and CTPA exposes the mother to a higher radiation dose, leading to a small, yet significant increase in the risk of breast cancer (1 case in 280,000 *versus* less than 1 case in 1,000,000).

The choice between V/Q and CTPA is divergent. Most recommendations indicate V/Q scintigraphy as a first choice, especially perfusion, in the presence of normal chest X-ray. Others, however, recommend using CTPA with low doses for diagnosing PTE, even though they produce a higher proportion of inconclusive results during gestation. Approximately 80% of scintigraphy examinations are diagnostic, i.e., 70% are normal, and 5% to 10% are high probability. Table 35 shows absorbed radiation doses of diagnostic tests for PTE during pregnancy.^131,340^

**Table 35:** – Estimated absorbed radiation of procedures used to diagnose pulmonary thromboembolism

Test	Estimated fetal radiation (mSv)	Estimated maternal breast radiation (mSv)
Chest X-ray	< 0.01	0.01
**Pulmonary perfusion scintigraphy with technetium 99m:**		
Low dose (40 MBq)	0.11 to 0.20	0.28 to 0.50
High dose (200 MBq)	0.20 to 0.60	1.20
Pulmonary ventilation scintigraphy	0.10 to 0.30	< 0.01
Pulmonary angiotomography	0.24 to 0.66	10 to 70

*mSv: millisievert.*

Pregnancy-Adapted YEARS Algorithm^334^ was applied for the diagnosis of PTE in a population of pregnant women and showed that in the absence of factors such as deep venous thrombosis, hemoptysis, PTE as the most likely diagnosis and, D-dimer not exceeding 1000 ng/ml, the diagnosis of PTE it can be ruled out and, consequently, chest angiotomography could be avoided in 32 to 65% of patients.^334^

***5.2.4.6. Differential Diagnosis***

Differential diagnosis of PTE is wide-ranging, given that pulmonary embolism has clinical manifestations similar to those of pneumonia, HF, and AMI. For this reason, it is wise to exclude the presence of coexisting pulmonary embolism with pneumonia manifestations. From the peripheral point of view, DVT in lower limbs should be differentiated from osteomuscular diseases, such as tendinitis, muscular distension, popliteal cyst, popliteal aneurysm, hematoma, cellulitis, lymphangitis, and post-thrombotic syndrome (Figure 10).

**Figure 10:** – *Flowchart for diagnostic investigation of pulmonary thromboembolism during gestation. PTE: pulmonary thromboembolism; US: ultrasound.*

## 5.2.5. Treatment

***5.2.5.1. General Approach***

Faced with strong clinical suspicion of thromboembolism, full permanent anticoagulation should be initiated before confirmation of diagnosis, unless it is contraindicated. Heparin is the preferred anticoagulant, whereas “new” oral anticoagulants, such as dabigatran, rivaroxaban, and apixaban, have not been approved for use during gestation and lactation. In cases of allergy or thrombocytopenia induced by heparin, fondaparinux may be indicated, and it seems to be safe during the second and third trimesters of pregnancy.

***5.2.5.2. Heparin Use***

LMWH and UFH are the options for treating PTE during gestation. LMWH is easy to use, and it seems to be safer and more efficacious than UFH, with data extrapolated from studies that did not include gestation. Intravenous UFH is indicated in patients with increased risk of bleeding or persistent hypotension during PTE. Prolonged heparin use, i.e., for more than 7 weeks, is associated with the risk of osteoporosis, hemorrhage, allergic reactions, skin necrosis, and thrombocytopenia, which are less frequent with the use of LMWH. Suspension is indicated when platelet count drops below 150,000 or the equivalent of 50% of the initial count. In this case, although it is controversial, substitution with fondaparinux may be indicated.

Anticoagulation should be continued throughout the pregnancy and at least during the first 6 weeks postpartum. Platelet count should be performed daily to investigate thrombocytopenia during the first 3 days of treatment and weekly thereafter.

***5.2.5.2.1. Recommended Doses***

- Subcutaneous LMWH: dalteparin 200 units/kg daily or 100 units/kg every 12 hours, or enoxaparin 1 mg/kg every 12 hours. The heparin dose should be controlled by anti-Xa factor in the therapeutic range between 0.6 and 1.0 IU/ml, when it is applied every 12 hours, and in the range of 1 to 2 IU/ml, when it is applied in a daily dose;
- Intravenous UFH: UFH bolus of 80 units/kg followed by an infusion of 18 units/kg/h, adjusted every 6 hours to maintain APTT between 1.5 and 2.5 times baseline. Stabilization of the therapeutic range allows for daily APTT control;
- Subcutaneous UFH: It is reasonable to initiate with 17,500 IU every 12 hours, adjusted every 6 hours to maintain APTT between 1.5 and 2.5 times control. Stabilization of the therapeutic range allows for daily APTT control.

***5.2.5.2.2. Labor and Delivery***

Delivery planning in patients under anticoagulation requires the involvement of a multidisciplinary team, as risks of bleeding and thrombosis should be weighed during the stages of labour, delivery and postpartum period. In cases of spontaneous labor, heparin should be suspended immediately; in planned induced or cesarean delivery, LMWH should be suspended 24 hours in advance; this practice makes neuraxial anesthesia possible. In cases when it is judged risky to suspend heparin for 24 hours, it should be substituted by intravenous UFH, which should be interrupted 4 to 6 hours before delivery. Neuraxial anesthesia may be performed when APTT returns to normal. In the event of planned preterm delivery (triplet gestation, premature rupture of membranes, significant cervical dilatation, preeclampsia, or IUGR), LMWH or subcutaneous UFH should be discontinued at week 36 and substituted with intravenous UFH.

In the occurrence of delivery in patients under full anticoagulation, more bleeding is predicted during the intrapartum and postpartum periods; in addition to this, the risk of spinal hematoma contraindicates neuraxial anesthesia. Accordingly, oxytocin use is suggested during the third stage of labor.^342^

***5.2.5.2.3. The Postpartum Period***

Heparin should be reinitiated 12 hours after cesarean delivery or 6 hours after vaginal delivery, once it has been verified that there is no significant bleeding. Warfarin, when it is indicated, should be initiated on the second day postpartum, in conjunction with heparin, until INR is between 2 and 3 IU. It is indispensable for patients to be on heparin when an oral anticoagulant is initiated, because the oral anticoagulant can stimulate coagulation and may cause vascular purpura during the first days. Oral anticoagulant use does not contraindicate lactation.

***5.2.5.2.4. Duration of Anticoagulation***

Duration of anticoagulant treatment should be individualized. According to studies in the general population, total duration should be from 3 to 6 months in patients with only transitory risk factors. Anticoagulation should be extended for at least 6 weeks postpartum; patients with persistent risk factors, however, may require more prolonged duration of anticoagulation.^131,342^

***5.2.5.3. Inferior Vena Cava Filters***

Temporary removable inferior vena cava filters may be used during gestation with indications similar to non-pregnant patients. This means that they are contraindicated in cases of conventional anticoagulation, such as the following: hemorrhagic stroke, active bleeding and recent surgery; thromboembolism in spite of full anticoagulation; need to interrupt anticoagulation; or when pulmonary circulation is significantly impaired. The use of vena cava filters is limited, because it is associated with risks of insertion and removal, such as filter migration in more than 20% of cases, filter fracture in more than 5%, perforation of the inferior vena cava in 5%, and mortality in 0.12% to 0.3%.^131^

***5.2.5.4. Thrombolysis***

Thrombolysis is reserved for patients with massive PTE and associated hypotension. Maternal mortality is estimated at 1%, fetal loss at 6%, and maternal hemorrhage at 8%. Intravenous UFH should be initiated immediately after thrombolysis, and LMWH should only be initiated once the clinical picture has stabilized.

## 5.2.6. Prophylaxis

Proposed prophylaxis regimes (Table 36) against thromboembolic phenomena during gestation in diverse clinical situations are the following:^131,336,338,342^

**Table 36:** – Proposed prophylaxis regimes

Clinical history	Practice during pregnancy	Practice during postpartum
History of thromboembolism with transitory RF unrelated to estrogen use or the current pregnancy*	Observation	Anticoagulant prophylaxis with a prophylactic or intermediate dose of UFH/LMWH for 6 weeks
History of idiopathic thromboembolism	Prophylactic or intermediate dose of UFH/LMWH	Anticoagulant prophylaxis with a prophylactic or intermediate dose of UFH/LMWH for 6 weeks
Patients with high risk** thrombophilias with history of thromboembolism	Prophylactic or intermediate dose of UFH/LMWH	Prophylactic or intermediate dose of UFH/LMWH for 6 weeks
Patients with lower risk of thrombophilia, without prior thromboembolism or family history of the disease	Observation or prophylactic dose of UFH/LMWH	Prophylactic or intermediate dose of UFH/LMWH for 6 weeks
High-risk patients, without previous thromboembolism and positive family history	Prophylactic or intermediate dose	Prophylactic or intermediate dose of UFH/LMWH for 6 weeks
Pregnant women with previous thromboembolism	Elastic socks	Elastic socks
Pregnant women with ovarian hyperstimulation syndrome	Prophylactic dose of LMWH during the first trimester	

* The British Society for Haematology recommends prenatal prophylaxis in this situation. ** High-risk thrombophilias: antithrombin deficiency, positive antiphospholipid antibody, homozygous for factor V Leiden, or mutation G20210A (prothrombin gene), double heterozygosis (factor V Leiden or mutation G20210A). LMWH: low molecular weight heparin; RF: risk factor; UFH: unfractionated heparin.

- Prophylactic UFH: 5,000 units of subcutaneous UFH, every 12 hours;
- Intermediate dose of UFH: 10,000 units of subcutaneous UFH, every 12 hours;
- Adjusted UFH: subcutaneous UFH, every 12 hours with APTT adjusted to 1.5 to 2.5 times baseline;
- Prophylactic LMWH: dalteparin (5,000 units subcutaneous daily), enoxaparin (40 mg or 0.5 mg/kg subcutaneous), or tinzaparin (4,500 units subcutaneous);
- Intermediate dose of LMWH: dalteparin (5,000 units subcutaneous, every 12 hours) or enoxaparin (40 mg subcutaneous, every 12 hours);
- Adjusted dose of LMWH: dalteparin (200 U/kg or 100 U/kg every 12 hours) or enoxaparin (1 mg/kg every 12 hours) in doses adjusted to 0.6 to 1.2 anti-Xa factor;
- Postpartum: Initiate with intravenous UFH or subcutaneous LMWH + warfarin until INR reaches 2.0. Subsequently, maintain warfarin for 4 to 6 weeks with INR between 2.0 and 3.0.

## 5.2.7. Key Points

- Thromboembolism is an important cause of morbimortality during gestation;
- Gestation and other related factors may increase the risk of the disease;
- Diagnosis of thromboembolism should be confirmed in order to justify treatment of the disease, which is prolonged, requires prophylactics measures, and has future therapeutic implications;
- When thromboembolism is suspected during gestation, venous ultrasound should be the first complementary examination solicited;
- While normal D-dimer dosage appears to have a negative predictive value, it has not been validated during gestation;
- Pulmonary V/Q scintigraphy or CTPA are examinations of choice for diagnosing PTE during gestation;
- Treatment of DVT or low-risk PTE during gestation is based on the use of LMWH or UFH;
- Treatment should be maintained throughout the entire gestation and at least for 6 weeks postpartum;
- Thromboembolic prophylaxis should be used in pregnant women with past history of thromboembolism. It should also be considered in the presence of other risk factors;
- Investigation of thrombophilia should be individualized;
- The absence of factors such as deep vein thrombosis, hemoptysis, PTE as the most likely diagnosis and, D-dimer not exceeding 1000 ng/ml, makes the diagnosis of PTE unlikely.

## 5.3. Therapy and Prevention

### 5.3.1. Heart Failure

HF stands out as the main cause of complications associated with maternal mortality in women with heart disease. It has a prevalence of 0.04% in the general population of pregnant women and 12.5% among women with heart disease. It is important to emphasize that approximately 60% of cases of HF occur during the postpartum period.^344^ Although they are asymptomatic, 0.85% of women in Brazil may eventually present ventricular dysfunction during the postpartum period.^345^ The most frequent situations that should be considered in diagnosis of HF during the pregnancy-postpartum cycle are shown in Table 37.^346^ HF associated with PPCM has been discussed in section 3.3.7.

**Table 37:** – Heart failure during pregnancy

Obstetric causes
Preeclampsia
Peripartum cardiomyopathy
Amniotic fluid embolism
Non-obstetric causes
Cardiomyopathy
Pulmonary embolism + right ventricular dysfunction
Obstructive valve disease (mitral and aortic stenoses)
Valve prostheses (calcification or thrombosis)
Cardiomyopathies due to cardiotoxicity (drug use)

*Adapted from: John Antony and Karen Sliwa.^346^*

Diagnosis of HF during gestation is difficult, because adaptive physiological changes during pregnancy cause signs/symptoms, which should be considered when they are exacerbated. In this manner, interface in interpretation of physiological symptoms of pregnancy *versus* those of HF, as shown in Table 38, requires the application of specific knowledge in order to make the most appropriate decision regarding eventual therapeutic intervention.

**Table 38:** – Signs and symptoms of pregnancy

Signs/symptoms	Normal pregnancy	Complicated pregnancy
Dizziness, palpitation	Common	Exercise syncope
Dyspnea	Common (75%), mild, and non-progressive	Progressive or NYHA FC IV
Orthopnea	Common, especially at the end of gestation	–
Decreased exercise tolerance	Mild and non-progressive	NYHA FC IV
Chest pain	Common, non-progressive, generally skeletal-muscular	Typical angina or important chest pain during gestation or in the postpartum period
Pulse	Increased volume or frequency	Decreased or ascending volume
Peripheral edema	Common, mild	Important or progressive
Apical heart sound	Hyperdynamic, slightly lateralized	Third sound with splitting
Heart rate	Common, sinus tachycardia	AF, persistent SVT, symptomatic ventricular arrhythmias
Neck veins	Slightly distended	Progressively distended with dominant ‘v’ wave

*AF: Atrial Fibrillation; FC: Functional Class; NYHA: New York Heart Association; SVT: Supraventricular Tachycardia.*

From initial evaluation to clinical follow up, the physician should pay attention to personal and family history of heart disease, gestational age at the time when FC progressed from I/II to III/IV, and identification of factors such as cardiac arrhythmias, anemia, and infections (Figure 11).

**Figure 11:** – *Algorithm for diagnosis of heart failure. BNP: natriuretic peptide; HFpEF: heart failure with preserved ejection fraction; HFrEF: heart failure with reduced ejection fraction. Adapted from Rohde et al., 2018.^345^*

Pregnancy is generally poorly tolerated in women with LVEF < 40% and FC III/IV (NYHA), both of which are considered predictive factors of mortality,^347^and pregnancy should be advised against. In cases with LVEF < 20%, pregnancy should be contraindicated, and, during the first trimester, interruption should be considered.

The routine for pregnant women with suspicion of HF should include basic subsidiary examinations, namely, the following: laboratory tests (blood count, serum electrolytes, renal function, fasting blood glucose, glycosylated hemoglobin, lipid profile, thyroid function and liver function); 12-lead ECG to identify arrhythmias, cardiac chamber overload, and conduction disorders; chest X-ray to detect pulmonary congestion; and 2-dimensional transthoracic Doppler echo with Doppler flow analysis, which is the preferred diagnostic imaging test, not only due to its wide availability, but also to the fact that it does not require ionizing radiation. Echo identifies structural cardiac alterations, including myocardial, valve, and pericardial abnormalities, in addition to evaluating hemodynamic aspects.^345^

Studies have confirmed the value of BNP as a marker for HF during gestation as well.^348^ Values above 100 pg/ml contribute to sustaining clinical diagnosis of HF, and they facilitate the implementation of appropriate therapeutic measures. It may be useful to incorporate serum levels of BNP into clinical practice, especially when assessing cardiac events during pregnancy.

Evaluation of prognosis of HF during pregnancy is similar to conventional evaluation; the following invasive examinations, however, should be postponed until after pregnancy: transesophageal echo, CMR, myocardial perfusion spect, PET scan**,** coronary angiotomography, and cardiopulmonary test.

HF prevention during gestation requires multidisciplinary counseling with the obstetrician, and it should observe the following recommendations: (1) weekly or biweekly medical consultation; (2) body weight control; (3) insisting on avoiding activities that require great effort; (4) moderate salt intake restriction; (5) eventual removal from professional activities that require great effort; (6) maintaining non-teratogenic medications and (7) hospitalizing patients who continue in NYHA FC III with optimized medication.^349^

Obstetric evaluation concomitant to cardiologic care is important to establishment of gestational age. In this manner, fetal viability and growth conditions and the placental flow situation are factors that support therapy and reflect the maternal hemodynamic condition.

Pharmacological treatment of HF with reduced ejection fraction (HFrEF) differs from treatment for the general population of women with heart disease regarding the class of drugs used, daily dose, and therapeutic goals,^52^ given that teratogenic drugs should be substituted during preconception.

Beta-blockers, especially beta-1-cardioselective ones (metoprolol, bisoprolol, and carvedilol), are considered first-line drugs, because they are beneficial with respect to mortality due to HF and CSD, and they improve symptoms and reduce rates of re-hospitalization due to HF.^345^ For these reasons, the use of these beta-blockers should be maintained during gestation in cases with HFrEF.

The literature is lacking in data on target doses for reaching therapeutic goals during gestation, which should not be the same as those considered for the general population of women with heart disease. This is because reduced heart rate and decreased arterial pressure resulting from high doses, which are usually factors applied to the population of patients with HF, can impair uteroplacental circulation.

It is generally prudent for doses of drugs used during pregnancy to be fractionated; they should initially be low and gradually increase, with caution, seeking the highest dose tolerated by the mother and the fetus. The following are thus recommended: an initial dose of bisoprolol of 1.25 mg daily, carvedilol of 3.125 mg 2 times daily, and metoprolol succinate of 12.5 mg 2 times daily, in accordance with the recommendations for the population of patients with HF.^345^

Vitality (biophysical profile and cardiotocography) and fetal maturity should be assessed more frequently when compared to the population of healthy pregnant women. During the neonatal period, supervision should last from 24 to 48 hours after birth, considering the most frequent symptoms and signs, such as respiratory depression, bradycardia, hyperbilirubinemia, and hypoglycemia. For this reason, when a patient is close to delivery, a prudent measure is to reduce the beta-blocker progressively, seeking the lowest dose with maternal efficacy.^344^

The occurrence of pulmonary congestion requires the use of loop diuretics, preferably furosemide and thiazide diuretics, in the attempt to optimize preload. In the event that there is no congestion, they should be avoided, due to the risk of causing reduced uteroplacental flow.^346^ Attention should be paid to the deleterious effects of the permanent use of diuretics, such as worsened placental flow, increased uric acid (early marker of preeclampsia), appearance of maternal-fetal electrolytic disorders, and IUGR.

Hydralazine may be used to treat symptoms of HF, with or without nitrates, as an alternative treatment in the event that SAP is > 110 mmHg, especially in cases with associated arterial hypertension, severe left ventricular dysfunction, and/or evidence of congestion.^52,345^Nevertheless, during pregnancy, the association between hydralazine and nitrates has been related to low maternal tolerance due to the usual arterial hypotension.

Digoxin may be used when volume overload persists, notwithstanding therapy with vasodilators and diuretics. When it is necessary in patients with HFrEF, digitalis plays an important role in controlling maternal heart rate, especially in the presence of AF.^345^

Anticoagulation in HF during pregnancy is controversial. LMWH or UFH may be considered in patients in the most common situations, such as dilated cardiomyopathy with LVEF < 35%, prolonged hospitalization and history of thromboembolic events. It is worthwhile to consider that the postpartum period adds a higher risk of thromboembolism; for this reason, anticoagulation is indicated during this phase of the pregnancy-postpartum cycle.

Regarding arrhythmias in HF, AF if the most common, and it may be treated with beta-blockers; if necessary, digoxin is added to control heart rate. Regarding frequent ventricular arrhythmias or sustained ventricular tachyarrhythmia, treatment includes the use of amiodarone and, when risks are higher, ICD are indicated.

When hemodynamic instability and cardiogenic shock occur, the patient should initially be transferred to the ICU, if possible, with MCS.^346^ Urgent cesarean delivery should be considered, with MCS immediately available; in the event of elective delivery, however, it is at the obstetrician’s discretion whether the route of delivery is vaginal or cesarean, considering maternal parity, existing comorbidities, and the severity of cardiac injury.

During the postpartum period, it is necessary to avoid volume overload as a result of infusion of fluids during the intrapartum and postpartum periods. The use of oxytocin in low doses should be considered, in spite of its vasoactive properties, and ergometrine should be avoided due to its peripheral vasoconstrictive effect.

### 5.3.2. Key Points

- The physiological symptoms and signs of pregnancy may delay diagnosis of HF;
- BNP (≤ 100 pg/ml) is a marker of HF that is also valid during pregnancy;
- Serial BNP during gestation assists in HF diagnosis and therapy;
- Beta-blockers are considered first-line drugs, and they should be maintained during gestation in cases of HFrEF;
- During family planning, pregnancy should be advised against in women with chronic HF who present LVEF < 40% and contraindicated in those in FC III/IV with LVEF < 20%.

## 5.4. Therapy and Prevention

### 5.4.1. Infective endocarditis

IE is rare during pregnancy; it occurs in 0.006% of the general population. However in patient with valve disease or congenital heart disease, this percentage reaches 1.2%.^270,350^ Patients with valve prostheses and complex cyanotic heart disease, as well as those who use illicit drugs, constitute a higher-risk group.

IE is a severe disease with maternal mortality close to 33%, consequent HF, and thromboembolic phenomena.^350,351^ During pregnancy, special attention should be paid to fever without an apparent cause and new precordial heart murmur, given that it appearance is very common during normal pregnancy.

The approach to IE requires multidisciplinary care in a tertiary cardiology center, with decisions supported by a heart team that is qualified to offer the resources available for diagnosis, treatment, and follow up, according to conventional recommendations.^350^

Prophylaxis for IE during pregnancy follows the same recommendations that apply non-pregnant patients.^350,351^ Given that the oral cavity is the entryway for the most frequent etiological agents, basic orientations for preventing IE include promotion of oral health, advice on hygiene, and periodic dental consultation for surveillance of gingivitis, which favors periodontal disease.

Antibiotic prophylaxis for dental treatment is controversial; nonetheless, when it is indicated, 2 g of oral amoxacillin or 600 mg of oral clindamycin are used for patients who are allergic to penicillin, 1 hour before dental intervention.

Antibiotic prophylaxis for IE at the moment of vaginal or cesarean delivery is also controversial,^350^ and the lack of evidence regarding disease prevention with antibiotic use at the moment of delivery renders their indication fragile. Nevertheless, it is necessary to consider that the occurrence of IE during the postpartum period is severe, given that, during this period, complications that elevate bacteremia (manual extraction of the placenta, curettage, or placental retention)^352^ are not predictable, and postpartum infection in Brazil is one of the leading obstetric causes of maternal death. For this reason, the decision to use antibiotic prophylaxis for IE at the moment of delivery should be at the discretion of the team caring for the parturient patient, with individualization of each case.

Although it is still controversial, clinical situations at a high risk of IE that may require routine antibiotic prophylaxis are shown in Table 39,^350^ and recommendations regarding means of application are shown in Table 40.

**Table 39:** – High-risk heart diseases for infectious endocarditis350

Prosthetic valves	
Transcatheter valve prostheses	
Prosthetic material used for valvuloplasty, such as rings for annuloplasty and artificial chord	
Prior infectious endocarditis	

**Congenital heart disease**	**Unoperated cyanotic**
	Complex heart disease with residual lesion (shunts, valve regurgitation in the graft location, valve tubes)

**Table 40:** – Antibiotics and doses used one hour before delivery

Antibiotic	Doses
Ampicillin	2.0 g IV or IM
Associated with gentamicin	1.5 mg/kg O, IV, or IM
Patients allergic to penicillin/ampicillin/amoxacillin	
Vancomycin	1.0 g IV for 1 h
Associated with gentamicin	1.5 mg/kg IV or IM

*IM: intramuscular; IV: intravenous; O: oral.*

Clinical diagnosis of IE reviews history of fever; chills; decline in general condition; embolic, peripheral, or central phenomena; vascular or immunological phenomenon; glomerulonephritis; and new murmur. Regarding complementary examinations, transthoracic Doppler echo should be performed whenever clinical suspicion exists; transesophageal echo is indicated when transthoracic echo is negative for IE and in cases of prosthetic valve. Blood cultures should be collected prior to the introduction of antibiotics. A minimum of 3 samples should be taken at 30-minute intervals, by means of sterile peripheral venipuncture, regardless of fever peak. Treatment should be initiated following blood culture collection, and it should be based on epidemiology, clinical history, and blood culture and antibiogram results, in accordance with conventional guidelines.^350,351^

It is worth remembering that the most common etiological agent of IE in Brazil is *Streptococcus viridans* in the oral cavity. The choice of antibiotic, intravenous administration, and duration of antibiotic therapy are the same as in non-pregnant patients, considering the possible toxic effects of antibiotics on the fetus.^52,350,351,353^

There are, accordingly, 3 groups of antibiotics classified regarding risks to gestation: (1) the safest, which include ampicillin, penicillin, amoxacillin, oxacillin, erythromycin, daptomycin, and cephalosporins; (2) those which present intermediate risk and should thus be monitored, such as vancomycin, imipenem, rifampicin, and teicoplanin; and (3) those that are contraindicated, namely, aminoglycosides, quinolones, and tetracycline.^354^

Surgical treatment in cases of IE follows conventional indications, such as failure of etiological treatment, refractory HF, repeat embolic phenomena, periprosthetic complications, abscess, or prosthetic dehiscence. It is recommended that delivery take place before cardiac surgery in cases where the fetus is viable.^351,353^

### 5.4.2. Rheumatic Disease

Rheumatic fever (RF) is an autoimmune disease which occurs following infection of the oropharynx by Lancefield Group A beta-hemolytic *Streptococcus*.^355^ The first rheumatic outbreak affects children in early childhood, and it contributes to an important number of women with valve disease in reproductive age and, therefore, during pregnancy.

Acute RF is rare during pregnancy, but its diagnosis should be considered in pregnant adolescents without previous prophylaxis or those who present a clinical picture of severe HF that does not correspond to the degree of valve involvement.

Diagnosis is guided by the Jones criteria and complementary examinations.^355^Both major (carditis, Sydenham’s chorea, migratory arthritis, erythema marginatum, and subcutaneous nodules) and minor (fever and arthralgia) criteria are valid during gestation; however, acute phase reagents, such as alpha acid-glycoprotein, C-reactive protein, and protein electrophoresis, may be influenced by pregnancy. For this reason, diagnosis is strongly based on the patient’s clinical presentation and history.

Accordingly, it is worth considering that Sydenham’s chorea is a common cause of chorea in patients who have prior history, and there should be differential diagnosis with chorea gravidarum, which may be associated with morbidities other than RF. Both manifestations of chorea are linked to high obstetric risks, such as fetal loss, and they require differential treatment.^355^

The same applies to the distinction between HF consequent to rheumatic carditis and chronic heart valve disease; both increase the risk of maternal death, and they have very different forms of treatment.^356^

Treatment of rheumatic outbreak, which is rare during pregnancy, should be the same as in the general population. Hospitalization is indicated in all cases of suspected carditis, incapacitating arthritis, or severe chorea, and home rest should last for a minimum of 4 weeks and, eventually, until delivery.^357^

Secondary prophylaxis for RF should be maintained during gestation in accordance with the following recommendations: penicillin G benzathine 1,200,000 IU intramuscular every 21 days or phenoxymethylpenicillin 250 mg orally 2 times daily. In patients who are allergic to penicillin, erythromycin 250 mg orally 2 times daily or clindamycin 600 mg daily are recommended.^357^ The use of sulfadiazine is contraindicated during pregnancy.

Duration of prophylaxis does not depend on occurrence during pregnancy, and it is related to the following factors: RF without prior carditis (for 21 years or 5 years after the latest outbreak, applying whichever covers the longer period); RF with prior carditis, mild residual heart valve disease, or resolved valve lesion (for 25 years or 10 years after the latest outbreak, applying whichever covers the longer period); moderate to severe residual valve lesion (for 40 years or lifelong); after valve surgery (for 40 years or lifelong). Patients with risk of repeat pharyngitis, such as those who work in daycare centers or nursing homes, should use secondary prophylaxis for the rest of their lives.^353,358^

### 5.4.3. Key Points

- Antibiotic prophylaxis for IE at the moment of delivery should be performed in patients at a high risk for IE;
- Prophylaxis for RF should be maintained during pregnancy.

## 5.5. Cardiovascular Surgery During Pregnancy

Worldwide experience in cardiac surgery during pregnancy has shown controversial results. Studies are characterized by retrospective nature and heterogeneity of procedures, associated with difficulties to standardization of surgical techniques, which render difficult the judicious analysis of prognostic variables and their reflexes in practice during pregnancy.^359,360^

It is accepted that the risk of maternal death due to cardiac surgery is not greatly modified by pregnancy.^359^ For emergency surgery, however, the risk of maternal mortality increases.^361^ The maternal mortality rate verified of 7.5% to 13.3% is relatively high, in comparison with that of cardiac surgery in the population of young women of fertile age, which encompasses the age range of pregnancy.^359,361,362,363^

Another important aspect for indication of cardiac surgery is gestational age. This is because, the earlier complications appear in patients with severe lesions, the greater the tendency to indicate early surgery, because there is a very high tendency for hemodynamic deterioration to progress during pregnancy, leading to an increase in emergency surgery and maternal death. This logic justifies the notion that the best period to plan cardiac surgery is during the second trimester of gestation, given that the fetus is still not viable, and the physiological and mechanical modifications pregnancy are still not very significant; furthermore, it provides the mother with a reasonable postoperative recovery period. One of the highest risk variables associated with worse maternal-fetal outcomes is emergency.^362,363^

Surgery during pregnancy requires specific precautions; the following stand out: choice of anesthetic drug, continuous maternal-fetal monitoring, and adequate control of anticoagulation. The obstetric team should initiate both maternal and fetal monitoring simultaneously, by means of cardiotocography, in order to control uterine dynamics and fetal heartbeat. Induction of anesthesia should be cautious to avoid periods of hypoxia and hypotension, and drugs without teratogenic effects should be chosen.^52^

Cardiovascular surgery techniques during pregnancy do not differ from those for non-pregnant patients; the surgical team’s experience, however, is fundamental in order to reduce duration of surgery, especially of CPB, in addition to specific precautions which are shown in Table 41.

**Table 41:** – Precautions for cardiac surgery with cardiopulmonary bypass during pregnancy

Control of hemodilution, which should not be below 25% hematocrit level
Use of flow 30% to 40% above usual flow, maintaining mean arterial pressure above 60 mmHg
Use of mild hypothermia or normothermia, in order to avoid fetal arrhythmias in cooling and warming and to decrease uterine contractions
Use of added glucose in the perfusate, in order to avoid fetal bradycardia and improve fetal energy conditions
Adequate control of acid-base balance, avoiding acidosis

Typically, a drop in fetal heart rate occurs during initial installation of CPB, which returns to normal by completion.^359^ This is mainly due to the change to continuous flow, embolic effect of microbubbles, initial hypotension, hemodilution, stacking of red cells, and alterations in peripheral vascular resistance. This “acute dysfunction” of the placenta as a result of impaired uteroplacental flow is the cause of the high incidence of fetal loss, prematurity, neonatal death, and malformations.^361,364^

It has been recommended to indicate delivery before cardiac surgery if the fetus is viable. Nevertheless, it is worth highlighting that corticoid use for fetal pulmonary maturation is very risky for pregnant women with unstable, severe hemodynamic conditions, which are very frequent in this situation. This is because corticoid use in recommended doses (2 doses of betamethasone, 12 mg intramuscular, 12 hours before delivery), associated with delivery, whether cesarean or vaginal, may lead to aggravation of HF, cardiogenic shock, and maternal death.

Prevention of premature labor with the use of natural progesterone suppositories (50 mg, every 12 hours during the intra- and postoperative) is preferable, given that indometacin may lead to closure of the arterial canal, especially after 26th week of gestation.^365^

Cardiac surgery, even though it constitutes a high risk for pregnancy, should be indicated for clinical conditions without other therapeutic options for maternal survival. Surgical procedures in emergency situations are significantly correlated with maternal complications during the postoperative period; for this reason, the moment of surgical indication has direct implications on maternal-fetal results.^361,366^

### 5.5.1. Key Points

- Cardiac surgery during pregnancy should be indicated in clinical conditions without other therapeutic options for maternal survival;
- Emergency surgery is significantly correlated with maternal complications during the postoperative period;
- Cardiac surgery during pregnancy requires differentiated precautions and a hospital protocol.

## 5.6. Percutaneous Cardiac Intervention

### 5.6.1. General Principles

The use of percutaneous interventions during gestation has gradually increased, driven by their greater availability and by the risks imposed during surgery with CPB. In general, these interventions are considered during gestation for severe symptomatic heart diseases whose treatment cannot be postponed because they pose risks to the mother’s life.^52^

The goal of percutaneous intervention during gestation is to save the mother’s life and protect the fetus from the potential risks of radiation. Accordingly, proposing that intervention be performed at the beginning of the second trimester takes the following into consideration: (1) organogenesis is almost complete; (2) fetal thyroid function is not active; (3) uterine volume is moderately increased (greater distance between the fetus and the maternal thorax); 4) facility of using barrier devices for protection.^52^

An alternative method to protect the fetus is by using echo (transthoracic, esophageal, or 3-dimensional) as a substitute to fluoroscopy. This makes it possible to place the catheters and to measure valve orifice diameters and aortic coronary outflow position; it also serves as a guide for balloon catheter valvuloplasty procedures and prosthetic valve insertion, including valve-in-valve procedures, and it assists coronary stent release.

Fluoroscopy should follow the criteria that include; (1) low radiation doses, (2) abdominal shielding, (3) distancing direct radiation from the abdominal region. Procedure duration should be as short as possible, because the risk of radiation to the fetus must always be taken into consideration. Nevertheless, this concern should not impede the use of essential diagnostic procedures, making use of the best available method for the given clinical situation.^52^

### 5.6.2. Percutaneous Valve Interventions

***5.6.2.1. Balloon Catheter Valvuloplasty in Mitral Stenosis***

BCV in mitral stenosis should preferably be performed during the second trimester of gestation, and it should be indicated for women with significant mitral stenosis in NYHA FC III/IV, who do not respond satisfactorily to conventional clinical treatment.^52^ The results of BCV, when its indications are followed, have been shown to be superior to those of conventional surgery, with lower mortality and better clinical condition in approximately 80% of cases.^367^

The criteria for indicating mitral BCV include the following:

- Absence of: (1) severe mitral regurgitation; (2) concomitant valve or coronary lesion requiring correction; (3) left atrial thrombus proven by transesophageal echo;
- Compatible anatomical condition of the mitral valve, namely: (1) certain flexibility; (2) non-excessive calcification; (3) commissural fusion; (4) approachable subvalvular portion;
- Wilkins echocardiographic score equal to or less than 8, allowing for better immediate and long-term result.^368^

Expanding to include patients with Wilkins score up to 10 as a result of pregnancy is controversial, because the potential for complications such as acute mitral insufficiency can be fatal. In very special situations, mitral BCV with an index above 8 requires previous discussion with a heart team and availability of resources in the event that emergency surgery is necessary.^369^

***5.6.2.2. Aortic Stenosis***

Patients who present severe aortic stenosis with manifestations of HF, limiting angina, and syncope during pregnancy are indicated for valve intervention, and balloon aortic valvuloplasty (BAV) may be performed by an experienced operator.^370^ In adolescents, it has good immediate and long-term results; in patients in higher age ranges, however, results are worse. BAV may thus serve as a “bridge”^371^ to temporary improvement in clinical condition, making it possible to reach gestational age for safe delivery in favorable hemodynamic conditions. It is worthwhile to remember that, when the procedure is performed, conventional rescue surgery should be available in the event of emergency. It is, furthermore, essential that, after gestation, these patients receive follow up with clinical examinations and periodic echo to determine the eventual need for definitive heart valve disease correction.

***5.6.2.3. Congenital Pulmonary Valve Stenosis***

Severe, symptomatic pulmonary valve stenosis (PVS) with manifestations of HF, arrhythmias, or syncope is uncommon during pregnancy. In this situation, BCV has been indicated with immediate success.^372^

***5.6.2.4. Percutaneous Implantation of Prosthetic Valve***

In recent years, we have witnessed the development of transcatheter aortic valve implantation (TAVI). It has the great advantage of avoiding cardiac surgery with CPB, but it requires intensive use of ionizing radiation by means of aortic valve tomography for preliminary study of the structures involved (aortic ring, prosthesis diameter, coronary height, and the thoracic and peripheral arterial system), as well as radioscopy during the procedure, to assist in catheter placement and visualization of prosthesis expansion. Conventional TAVI is thus, not approved, during gestation, due to the high fetal radiation burden.

This notwithstanding, arterial ultrasound for evaluation of the arterial system (iliac, aorta, and coronary height), in conjunction with 3-dimensional echo (evaluation of the valve ring) was successful in the first reported case of TAVI during gestation,^373^ which used short periods of radioscopy to place the prosthesis. The fact that pregnant women are younger, with healthy arterial vascular beds, facilitates navigation with catheters; nevertheless, there should exist a degree of valve calcification to allow for prosthesis placement, and this is not always found in this group of patients.

***5.6.2.5. Valve-in-Valve Procedure for Bioprosthetic Valve Dysfunction***

BPV dysfunction is very common in young women, and it sometimes requires valve replacement during gestation. In this scenario, valve-in-valve type procedures are promising in order to avoid surgery with CPB. Prostheses are introduced by means of catheters, using the following routes: femoral artery or other arterial accesses to the aorta, femoral vein followed by transseptal puncture and left atrial access, and left ventricular apical (transapical) incision. A case report^374^ during pregnancy has described transapical implantation of 2 prostheses, mitral and aortic, with the aid of transesophageal echo and restricted use of fluoroscopy, which made it possible to reach vaginal delivery with positive maternal-fetal results.

***5.6.2.6. Coronary Angioplasty***

Primary percutaneous coronary intervention is the treatment of choice for acute coronary syndrome during gestation, while thrombolysis is less utilized. Coronary angioplasty with conventional stents has been considered safe in cases of obstructive arterial disease due to atherosclerotic disease.

While the safety of drug-eluting stents is still not known, the need for dual antiplatelet therapy for a prolonged period of time with this type of stent constitutes a serious restriction to their use during gestation, owing to the hemorrhagic risks. Furthermore, clopidogrel should be interrupted 7 days before delivery, which adds a risk of stent thrombosis.

In spontaneous coronary dissection, the indication for angioplasty should consider the technical difficulties and the vascular fragility peculiar to this situation, which increases the risk of extension of coronary damage, in addition to the fact that its success is considered suboptimal.^375^For this reason, most cases of coronary dissection benefit from conservative treatment.^376,377^ In situations where coronary angioplasty is indicated, the option to use the latest generation of drug-eluting stents, which require dual antiplatelet therapy for a shorter time (3 months) may be a safer option.

The dilemma of this decision is that obstetric risk (maternal hemorrhage) and cardiac risk (stent thrombosis) must be judged on a case-by-case basis by an interdisciplinary team, because, to date, there are no studies on these circumstances that support decision making.

### 5.6.3. Key Points

- Percutaneous intervention during pregnancy should be indicated in cases of complications refractory to conventional clinical treatment or in conditions of imminent risk of maternal life;
- Percutaneous intervention should always be performed after discussion with the Heart Team in Tertiary Cardiology Services.

## 5.7. Cardiovascular Emergencies

### 5.7.1. Acute Heart Failure

Circulatory overload during the pregnancy and postpartum period in patients with structural heart disease, even if it is asymptomatic, may be responsible for acute heart failure (AHF),^378^treating it during gestation can lead to improvement of symptoms and prevention of maternal death. The orientation of attendance follows the recommendations for patients with HF in the emergency room^345^ (Figure 12), but it is necessary to consider the risks of medication use regarding the mother, the fetus, labor, and lactation, as well as necessary adjustments according to gestational age.

**Figure 12:** – *Algorithm for diagnosis when there is clinical suspicion of acute heart failure. ER: emergency room. Adapted from Rohde et al., 2018.^345^*

It is worth mentioning that in addition to the symptoms of CHF, the identification of systemic and/or pulmonary congestion and low output, supported by subsidiary exams, define the determining cause in most cases.^345,378,379^

Laboratory examinations should be part of the investigation of AHF during pregnancy, and they include the following: electrolyte dosage, BNP,^348,380^ renal function, markers of myocardial necrosis, thyroid profile, blood count, and other infectious parameters.

Interaction with the obstetric team is mandatory to determine both gestational age and parameters of fetal vitality and viability. Eventual indication of therapeutic delivery and the route of delivery should be part of the algorithm for attending cases with AHF during pregnancy.

Acute dyspnea during pregnancy should include the following differential diagnoses: AMI, pulmonary congestion in preexisting heart disease, PPCM, PTE, and myocarditis.^222^Orientation for differential diagnosis may be summarized by the following points:

- AMI: dyspnea and angina pain; over 35 years of age; history of tobacco use and use of contraceptives with estrogen components; elevated serum troponin levels; echo with alterations in segmental motility. Definitive diagnosis is made by coronary cineangiography;
- Preexisting heart disease: Dyspnea is more frequent during the second and third trimesters. Serum levels of BNP may be elevated, and echo shows structural cardiac injury. In Brazil, acute pulmonary edema is common as the first manifestation of mitral stenosis, from the second trimester of gestation on;
- PPCM: dyspnea during the last month of gestation or, more frequently, after delivery, with significant elevation in levels of BNP and new systolic dysfunction in the left and right ventricles. CMR is important to determine diagnosis;^222^
- Thromboembolism: Dyspnea is associated with pleuritic chest pain. Levels of troponin and BNP are elevated, and right ventricular dysfunction and PH are signs of greater severity of this event. It is worth emphasizing that sensitivity and negative predictive value of D-dimer are limited when there is suspicion of PVS during pregnancy;^381^
- Myocarditis: Dyspnea is associated with unspecific symptoms related to viral infection. Troponin may be elevated (myocardial inflammatory processes increase cellular release), and echo may demonstrate segmental akinesis or diffuse hypokinesis. CMR with identification of myocardial edema or mesocardial fibrosis reinforce diagnosis.^382,383^

During clinical evaluation, it is fundamental to determine hemodynamic profile. In patients classified as profile B (wet and warm), volume adjustment with diuretics and vasodilators, in the absence of hypotension and shock, should be considered sparingly, keeping the formal contraindication to the use of ACEI and ARB in mind and giving preference to the use of nitrates and hydralazine, in combined therapy, whenever possible.

Loop diuretics are safe. Furosemide is the most commonly used, at an initial dose of 20 to 40 mg, with the possibility of optimization, depending on previous chronic use, diuretic response, and improvement of dyspnea and hypoxemia.^384^Fetal risks are consequent to reduced placental flow due to volume adjustment beyond what is necessary.

In more severe patients or cases of acute pulmonary edema, without hypotension or shock, nitroglycerin or sodium nitroprusside is used in continuous infusion, preferably guided by invasive arterial monitoring. Doses and infusion rates are described in Table 42. Continuous fetal monitoring should also be performed, seeing that the abrupt reduction in maternal arterial pressure may compromise fetal vitality.

**Table 42:** – Recommendations for intravenous vasodilators in acute heart failure

Vasodilator	Posology	Adjustments
Nitroglycerin	Initial: 10 to 20 mcg/min Maximum: 200 mcg/min	Every 15 min Increase: 10 to 20 mcg/min
Sodium nitroprusside	Initial: 0.3 mcg/kg/min Maximum: 5 mcg/kg/min	Every 15 min Increase: 0.3 to 0.5 mcg/kg/min

Non-invasive ventilation (NIV) support with positive pressure is indicated for all patients with peripheral arterial saturation < 90% and respiratory distress or discomfort who do not improve with oxygen therapy.^369^ It is also indicated for patients with acute pulmonary edema, given that, in non-pregnant women, it is known to have benefits for reducing the need for invasive mechanical ventilation support.^348^

In patients with symptomatic hypotension, signs of low cardiac output with organic dysfunction, or cardiogenic shock, there is a need for inotropic agents and, in some cases, association with vasoconstrictors, similarly to non-pregnant patients. Dobutamine is the most widely used inotropic agent, because it promotes a dose-dependent increase in cardiac output, even though its arrhythmogenic effect is limiting, and it presents lower efficacy in cases of chronic beta-blocker use. Milrinone, in addition to increasing cardiac output, is able to reduce peripheral and pulmonary resistance. It is, therefore, indicated in patients with congenital heart disease and PH.^344^ Levosimendan presents a positive inotropic effect, due to its vasodilatory action, however, it should be used with greater caution in pregnant women. Table 43 shows drugs and their recommended doses for treatment of AHF during pregnancy. In patients with AHF due to PPCM, as discussed in section 3.3.7, levosimendan is preferable, keeping the biomolecular effects inherent to catecholamines in mind. A recent study has demonstrated a beneficial effect of levosimendan (at a dose of 0.1 mcg/kg/min) in relation to improvements in ventricular function and systemic congestion in pregnant women with AHF due to PPCM.^385^

**Table 43:** – Posology of inotropic and vasoconstrictor drugs

Inotropic	Posology	Maximum dose
Dobutamine	2.5 mcg/kg/min Evaluate adjustment every 15 min Hemodynamic effect in up to 2 h	10 to 20 mcg/kg/min
Milrinone	Initial: 0.375 mcg/kg/min Adjustment every 4 h	0.75 mcg/kg/min 0.5 mcg/kg/min*
Levosimendan	0.1 mcg/kg/min Adjustment of 0.05 mcg/kg/min every 4 h Infusion for 24 h	0.15 mcg/kg/min
Norepinephrine	Initial: 0.1 to 0.2 mcg/kg/min Adjustment every 15 min	1 mcg/kg/min

* Dose for patients with renal insufficiency.

Norepinephrine is indicated in the occurrence of significant arterial hypotension or cardiogenic shock, because, in addition to its vasoconstrictor effect that modulates vasoplegia and redistributes blood flow, it also has an effect on cardiac output. In refractory patients, who do not respond to pharmacological measures, success has been described with the use of temporary mechanical circulatory assist devices, such as intra-aortic balloon (IAB) and extracorporeal membrane oxygenation (ECMO).^386^

### 5.7.2. Arrhythmia

The main consideration in practice for poorly tolerated arrhythmias with hemodynamic impact is to prioritize the mother’s life. Nonetheless, treatment should also be weighed in relation to the side effects of antiarrhythmic drugs on maternal cardiac output and uteroplacental flow, oxytocic effects, and proarrhythmogenic effects on the fetus.

For these reasons, antiarrhythmic medication, maintenance, discontinuation, or dose optimization should be individualized depending on the type of arrhythmia, gestational period, maternal structural disease, and risk of sudden death.^387^

Nodal reentry tachycardia is the most common SVT, followed by atrioventricular tachycardia. Its occurrence is more frequently observed during pregnancy; its treatment in the emergency room, however, does not present modifications in relation to non-pregnant women. In stable patients, the vagal maneuver is the first choice, followed by the use of adenosine, which does not pass the placental barrier, in a bolus (6 mg, followed by 12 mg if it persists). Regarding CCB, verapamil is a good, safe option. In patients with signs of pre-excitation on resting ECG, there is a formal contraindication to the use of beta-blockers. In patients with hemodynamic instability, synchronized electrical cardioversion is indicated.^388^ There are no contraindications to cardioversion, and, other than choosing the most appropriate form of sedation, there are no additional precautions.^74^ Indication for catheter ablation may be considered during pregnancy, using electromechanical mapping in refractory cases.

AF, atrial flutter, and atrial tachycardia are uncommon during gestation in patients without structural cardiac injury. In situations of accelerated ventricular response, there is a risk of hemodynamic degeneration in both the mother and the fetus. In all patients, it is necessary to rule out association with infection, anemia, and thyrotoxicosis.^389^ In order to control AF frequency in patients with high ventricular response, lanatoside-C, verapamil, or metoprolol are used. Under hemodynamic instability that might be attributable to tachycardia, synchronized electrical cardioversion is indicated. Patients with AF and heart valve disease have a precise indicated for anticoagulation. In cases that are more clinically stable, when opting for rhythm control, electrical cardioversion is preferable to chemical cardioversion, considering the teratogenic effect of amiodarone and the scarcity of evidence in relation to the safety of high doses of propafenone. In cases where time since onset of arrhythmia exceeds 48 hours, it is necessary to perform transesophageal echo.^390^

For patients with flutter, cardioversion is preferable, given its high reversibility rate, observing less than 48 hours of onset or after performance of transesophageal echo to rule out the presence of intracavitary thrombi.

The occurrence of VT during gestation is rare, but it may occur in high-risk patients, especially those with structural disease and ventricular dysfunction. Electrical cardioversion is indicated when the maternal clinical picture is unstable. In patients without hemodynamic instability, lidocaine is safe, and it has the best reversibility rate. The use of amiodarone should be excluded to isolated situations, when cases are refractory and ventricular arrhythmia recurs following electrical cardioversion, and it is necessary to be aware of its dose-dependent effects on the fetus.^391^ICD implantation in indicated patients is approved during pregnancy when it ensures better prognosis during delivery and the postpartum period.^392^

### 5.7.3. Acute Myocardial Infarction

AMI, which is uncommon during pregnancy, is potentially fatal. Over the past decades, its incidence has been found to increase, notwithstanding reduced maternal mortality due to the issue during gestation.^224^

In general, practice for treating AMI during gestation follows the same recommendations as the general population, including revascularization with stent angioplasty or surgical revascularization.^393^Multiprofessional care includes obstetric evaluation and continuous monitoring of the fetus, with evaluation of fetal vitality and cardiotocography.

Clinical treatment of AMI during pregnancy considers the following:^394^

- Oxygen therapy: nasal O_2_ catheter, 2 to 3 L/min;
- Pain control: Morphine sulfate is considered to be safe and effective, but it may lead to respiratory depression in the fetus if administered near delivery;
- Nitrates: Attention should be paid to the risk of maternal hypotension and consequent low uteroplacental flow;
- Beta-blockers: metoprolol, carvedilol, or propranolol. Fetal monitoring with cardiotocography is recommended to control uterine dynamics and fetal heartbeat;
- Aspirin: low doses (< 150 mg);
- Clopidogrel may be used, but it should be suspended 7 days before delivery;
- Heparins: UFH and LMWH are used according to indications. Fondaparinux should only be used when heparins are contraindicated.

Indicated treatment of AMI with ST-segment elevation is coronary reperfusion, as early as possible,^389,395^ by means of either thrombolytic^396^ drugs or, preferably, primary coronary angioplasty with stents. Thrombolytics should be restricted to cases where the hemodynamic room is not available in a timely manner. Restrictions to its use are due to the risk of placental hemorrhage. If percutaneous angioplasty is indicated, there is still controversy regarding the preference of conventional stents to drug-eluting stents.^52^

Risk stratification of patients with acute coronary syndrome without ST segment elevation is indicated, in the same manner as in non-pregnant patients, considering age, vital signs, risk factors, recent or recurrent symptoms, and electrocardiographic and laboratory findings. In low-risk pregnant patients without signs of HF, refractory pain, or electric instability, conservative clinical treatment is indicated. In contrast, in high-risk pregnant patients, invasive stratification during the first 24 to 48 hours following the onset of the acute condition should be prioritized in order to proceed to myocardial revascularization.^396^

Spontaneous coronary artery dissection is a frequent cause of AMI in women, it should, therefore, be the first hypothesis when faced with an acute ischemic event during gestation. Treatment should follow conventional recommended measures.^397^

### 5.7.4. Acute Aortic Syndrome

Most acute aortic syndromes occur in women with diseases predating gestation, but they may also affect patients who were previously healthy. It is estimated that the incidence of dissection of the aorta in the population is from 2.4 to 2.9 out of 100,000 patients yearly, and there appears to exist a strong correlation with pregnancy in women under 40 years of age.^398^

Chest pain in women with aortic disease requires investigation with angiotomography of the aorta, in order to rule out suspicion of acute dissection of the aorta. In pregnant patients with type A dissection, with involvement of the ascending aorta, there is an indication for emergency cardiac surgery, in addition to pressure and heart rate control. The procedure should take place in conjunction with a multiprofessional team in a tertiary cardiology center, and cesarean delivery is indicated when the fetus is viable, followed by correction of the dissection. In situations where the fetus is not viable, cardiovascular surgery is performed, prioritizing the mother’s life (contemplating that fetal mortality is from 20% to 30%).^399^

In women with uncomplicated type B dissection of the aorta, without involvement of the ascending aorta, initial conservative treatment is indicated, maintaining adequate arterial pressure and heart rate control. In the event that there are signs of complication, such as persistent pain, uncontrolled arterial hypertension, progression of dissection, ischemia in a target organ or symptoms of aortic rupture, percutaneous treatment should be considered, even though it has been little described during gestation.^400^Route of delivery should be cesarean once fetal viability has been ensured.

### 5.7.5. Prosthetic Valve Thrombosis

The incidence of thrombosis in mechanical prostheses during pregnancy varies according to the anticoagulation regime utilized. Diagnosis should be considered in previously asymptomatic pregnant women who present dyspnea, chest pain, and symptoms of hypotension. Transesophageal echo is the gold standard examination for definition.^401^

Treatment of valve thrombosis during pregnancy or the postpartum period should be the same as that proposed for non-pregnant patients, taking their clinical condition, thrombus size and localization of the affected prosthesis into consideration.^96^

Thrombolytic use should be considered in critical patients who would present great risks of death if they underwent surgery, in places where a surgical team is not available, and in the event of thrombosis in the tricuspid or pulmonary valve. The following thrombolytic doses are recommended: streptokinase, 1,500,000 IU for 60 minutes without UFH; or alteplase (rT-PA), 10 mg in a bolus + 90 mg for 90 min with UFH.^151,402^ In partially successful cases, i.e., cases that persist with residual thrombi, patients should be referred for surgery 24 hours after thrombolytic infusion has been discontinued.

A protocol with low-dose thrombolytic therapy in slow infusion (rT-PA 25 mg, intravenous infusion for 6 hours, repeated after 24 hours and, if necessary, up to 6 times, reaching a maximum dose of 150 mg, without bolus or concomitant use of heparin) has recently been proposed for pregnant women with prosthesis thrombosis. The results have shown that thrombolysis was efficacious, with no maternal deaths, and fetal mortality rate was around 20%, which is better than the routinely used strategies.^403^ With the enhancement of surgical techniques, however, it is not possible to infer that thrombolysis is superior to surgery during pregnancy.

The issue with surgery is due to high perioperative mortality (between 5% and 18%), which is closely associated with NHYA FC, which is the main predictor variable. Patients in NHYA FC I/III present 4% to 7% mortality, whereas those in FC IV present 17.5% to 31.3%. In contrast, surgery presents a higher rate of success than thrombolysis (81% *versus* 70.9%).^399^ In this scenario, it should be considered in urgent or emergency cases, depending on the patient’s clinical condition. Surgical procedures are associated with maternal and fetal risks, when performed during pregnancy.

In patients with non-obstructive thrombi, who are stable from the hemodynamic point of view and who have no signs of decompensated HF, parenteral anticoagulation at therapeutic doses, with heparin according to APTT and echocardiographic imaging control, is the option. In cases that fail to respond to treatment, thrombolysis or conventional surgery should be indicated.^151,402^

### 5.7.6. Cardiorespiratory Arrest

Cardiorespiratory arrest (CRA) in pregnant women is one of the most dramatic and challenging situations in the emergency room. Although the steps for cardiopulmonary resuscitation (CPR) in pregnant women are very similar to those related to the conventional protocol stipulated by advanced cardiac life support (ACLS), there are different details that require due attention, which are summarized in Figure 13.^404^

**Figure 13:** – Flowchart for guiding intra-hospital care for cardiorespiratory arrest in pregnant women. ACLS: advanced cardiology life support; CRA: cardiorespiratory arrest; IV: intravenous; OTI: orotracheal intubation; PMCS: postmortem cesarean section. * Causes are shown in Table 44.

It is worthwhile to remember that many episodes of CRA are preceded by signs of hemodynamic instability. For this reason, teams providing care should receive training regarding not only prompt recognition and evaluation of these findings, but also complete performance of CPR in a synchronous manner.^405^

The mechanical effects of the pregnant uterus can aggravate desaturation and hypotension in aortocaval compression, favoring cardiorespiratory collapse.In the attempt to reduce aortocaval compression by the gravid uterus, manual left uterine displacement should be performed throughout attendance and during care following CRA.^406^

When indicated, defibrillation should be performed promptly, without delay or questioning. It is known that it does no harm to the fetus; it is completely safe, and the energy doses established by current protocols should be maintained.^407^

In the same manner as the indications for defibrillation regarding energy doses, medications and their doses should be the same as those defined by protocols used in adults in general.^405,407,408^

Attention should be paid to venous access above the diaphragm, thus minimizing the effects of aortocaval compression caused by the gravid uterus, which would make it difficult to recirculate the medication.^409^

For pregnant women, in addition to considering the classic causes of CRA established by the ACLS protocol, which makes use of a mnemonic device with letters *A* to *H*, there are other diverse conditions which may favor cardiorespiratory collapse, and which may be corrected^409^ (Table 44).

**Table 44:** – Main causes of cardiorespiratory arrest in pregnant women and maternal mortality

Letter	Causes	Etiology
A	Accident/trauma Anesthetic complications	High neuraxial block Hypotension Bronchoaspiration Respiratory depression Respiratory airway obstruction Trauma Suicide
B	Bleeding	Coagulopathy Uterine atony Placenta accreta Placenta praevia Uterine rupture Premature placental abruption Transfusion reaction Retained products of conception
C	Cardiovascular causes	Acute infarction Dissection of the aorta Cardiomyopathy Arrhythmias Valve disease Congenital heart disease
D	Drugs	Oxytocin Magnesium Illicit drugs Opioids Insulin
E	Embolic causes	Amniotic fluid embolism Pulmonary embolism Cerebrovascular event
F	Fever	Infection Sepsis
G	General	H’s (hypovolemia, hypoxia, hypoglycemia, hypokalemia, hyperkalemia, hypothermia) T’s (tension pneumothorax, cardiac tamponade, toxicity, infarction, and pulmonary thromboembolism)
H	Hypertension	Preeclampsia Eclampsia HELLP syndrome Intraparenchymal bleeding

*HELLP: hemolysis, elevated liver enzymes, and low platelet count.*

As soon as CRA is identified in a pregnant patient, the performance of perimortem cesarean delivery should promptly be considered if the patient’s uterus is above her umbilicus.^410^This measure is characterized by performing cesarean delivery and birth of the fetus after maternal CRA, in most cases during the period of CPR. A review of the last decade has shown that perimortem cesarean delivery is related to maternal survival in 31.7% of cases, and it has no harmful effects on the mother.^411^

One of the purposes of performing this type of delivery is to facilitate CPR, because it possible to release aortocaval compression by the gravid uterus completely, seeing that lateralizing it to the left is not sufficient. The other purpose is to deliver the child, reducing the risk of anoxia during the period of CRA, thus minimizing definitive neurological sequelae.^412^

The decision to perform urgent cesarean delivery should be made within the first 4 minutes after CRA. Delivery should be in the same place as attendance for CPR, given that patient transfer may lead to delays that increase risks to the fetus and compromise resuscitation maneuvers.^409^It is worth highlighting that the entire CPR protocol should be maintained during performance of the procedure. In situations where the maternal clinical picture is considered irreversible, perimortem cesarean delivery should be performed immediately.

### 5.7.7. Key Points

- In emergency cases, practice should prioritize the mother’s life. It is not justified to omit any treatment that is essential to the mother on account of concerns regarding potentially harmful effects to the fetus;
- Practice for cardiac emergencies during pregnancy should follow conventional protocols, such as ACLS.
- Cesarean section is considered “perimortem” in pregnant women with uterine height above the umbilical scar, in order to improve the maternal-fetal prognosis.

## 6. Family Planning

### 6.1. Pregnancy Counseling and Maternal Risk Stratification

Preconception counseling is essential for women of reproductive age with heart disease, with emphasis on maternal and fetal risks related to gestation and information regarding the safety and efficacy of contraception. The criteria of functional evaluation for approving or contraindicating pregnancy include anamnesis, clinical examination, and subsidiary examinations, such as ECG, chest X-ray, transthoracic or transesophageal echo, CMR, ergospirometry test, and other more specific tests. Invasive intervention for eventual treatment of cardiac lesions, if indicated, should be performed before gestation.

Once a diagnosis of heart disease (anatomical, functional, and syndromic) has been determined, the risk of pregnancy is weighed together with the couple or relatives.^270^ Identification of risk predictors for pregnancy contributes to determining maternal prognosis and decision making, such as approving or advising against conception.

The prospective multicenter study known as CARPREG^190^ considered a study population composed 75% of women with congenital heart disease and 25% of women with acquired heart disease, verifying cardiovascular complications in 13%, including 3 cases of maternal death. The predictors of maternal mortality proposed by this study are shown in Table 45.

**Table 45:** – Predictors of maternal events and risk score from the CARPREG study

1. Previous cardiac event (HF, transitory ischemic attack, pulmonary stroke prior to gestation, or arrhythmia)
2. NYHA FC > II or cyanosis
3. Left heart obstruction (mitral area < 2 cm^2^, aortic valve area < 1.5 cm^2^, or peak left ventricular outflow gradient > 30 mmHg on echo)
4. Reduced systolic ventricular function (< 40%)
**CARPREG risk score (each predictor is worth 1 point)**
• 0 points – 5% risk
• 1 point – 27% risk
• More than 1 point – 75% risk

*FC: functional class; HF: heart failure; NYHA: New York Heart Association.*

Subsequently, the ZAHARA study^413,414^ defined independent predictors of mortality for women with congenital heart disease, generating a very specific risk estimate. The event rate in the 1,300 women studied was 7.6%, and the most frequent complications were arrhythmia (4.7%) and HF (1.6%) (Table 46).

**Table 46:** – Predictors of maternal risk from the ZAHARA study

History of arrhythmia before gestation – **1.5 points**
HF with NYHA FC > II – **0.75 points**
Left heart obstruction (aortic valve stenosis with peak gradient > 50 mmHg or valve area < 1 cm^2^) – **2.5 points**
Mechanical prosthetic valve – **4.25 points**
Moderate to severe systemic atrioventricular valve regurgitation (possibly due to ventricular dysfunction) – **0.75 points**
Moderate to severe subpulmonary atrioventricular valve regurgitation (possibly due to ventricular dysfunction) – **0.75 points**
Cardiovascular medication use before gestation – **1.5 points**
Repaired or unrepaired cyanotic heart disease – **1 point**
**ZAHARA** risk score:
0 to 0.5 – 2.9% risk
0.51 to 1.5 – 7.5% risk
1.51 to 2.5 – 17.5% risk
2.51 to 3.5 – 43.1% risk
> 3.5 – 70% risk

*FC: functional class; HF: heart failure; NYHA: New York Heart Association.*

The classification for the WHO which divides heart diseases by increasing level of severity: (1) risk I includes low-risk heart diseases (accepted as equal to that of the general population); (2) risk II denotes a slight risk of mortality and moderate risk of morbidity; (3) risk III, there is a significant risk of mortality or severe morbidity, (4) risk IV denotes a high risk of mortality that contraindicates pregnancy (Table 47).^415^

**Table 47:** – Modified WHO classification

Risk I
• Pulmonary stenosis, PDA, and mild to moderate uncomplicated mitral valve prolapse
• IAC, IVC, PDA, and uncomplicated, successfully repaired pulmonary vein drainage anomalies
• Isolated atrial or ventricular extrasystoles
**Risk II (uncomplicated):**
• Unoperated uncomplicated IAC and IVC
• Repaired tetralogy of Fallot
• Most arrhythmias
**Risk II-III (individualized evaluation)**
• Mild left ventricular impairment
• Hypertrophic cardiomyopathy
• Native or tissue valvular heart disease (not considered WHO risk I or IV)
• Marfan syndrome without aortic dilatation
• Bicuspid aortic valve with aorta diameter < 45 mm
• Repaired coarctation of the aorta
**Risk III**
• Mechanical prosthetic valve
• Systemic right ventricle
• Fontan circulation
• Cyanotic heart disease (unrepaired)
• Complex congenital heart diseases
• Marfan syndrome with aorta diameters between 40 and 45 mm
• Bicuspid aortic valve with aorta diameters between 45 and 50 mm
**Risk IV (pregnancy contraindicated):**
• Pulmonary arterial hypertension of any etiology
• Severe systemic right ventricular dysfunction (LVEF < 30%, NYHA FC III/IV)
• Peripartum cardiomyopathy with ventricular dysfunction
• Severe mitral stenosis, severe symptomatic aortic stenosis
• Marfan syndrome with dilated aorta > 45 mm
• Aortic dilatation associated with bicuspid valve > 50 mm
• Turner syndrome with aortic index > 25 mm/m^2^
• Tetralogy of Fallot with aorta > 50 mm
• Ehlers-Danlos syndrome
• Fontan procedure with any complication
• Severe coarctation of the aorta

*FC: functional class; IAC: interatrial communication; IVC: interventricular communication, LVEF: left ventricular ejection fraction; NYHA: New York Heart Association; PDA: patent ductus arteriosus.*

Comparison between the 3 studies,^324^ considering the CARPREG, ZARAHA, and WHO scores, revalidated the WHO classification as the most accepted and reliable for predicting risks of heart disease to pregnancy (Tabela 47).

Patients included in the IV-WHO risk should be advised against pregnacy.^324^ The Registry of Pregnancy and Cardiac Disease (ROPAC) validated the modified WHO classification,^416^ which includes an intermediate category (risk II/III-WHO) which means moderate risk of morbidity and mortality. This study also showed differences between developed and emerging countries regarding the characteristics of heart diseases and the complication rates that can lead to distortions in the interpretation of the risk score. The ESC^52^ Guidelines suggest using the modified WHO classification to establishment maternal risk.

This Brazilian Statement understand that WHO classification is the most accepted, and it should be applied to risk stratification of heart diseases for pregnancy. It is worth considering that complicating factors that are expected throughout the natural history of heart diseases, such as complex arrhythmias, prior HF, thromboembolism, or IE, aggravate maternal risk. The resources for care and the availability of a multidisciplinary team should also be considered and individualized during pregnancy counseling.

The ESC Guidelines^52^ added aortic diseases associated with the following to WHO risk IV category: Turner syndrome (aortic size index of 25 mm/m^2^); tetralogy of Fallot (aorta diameter > 50 mm), Ehlers-Danlos vascular syndrome; and Fontan circulation with complications.

#### 6.1.1. Key Points

- Family planning is essential for women with heart disease, regarding both risk stratification for pregnancy and contraceptive choice;
- Risk predictors should be defined before pregnancy;
- The risk classification elaborated by the WHO is currently the most accepted;
- Resources for attendance and availability of a specialized multidisciplinary team should be considered during pregnancy counseling.

## 6.2. Contraception in Patients with Cardiovascular Disease

### 6.2.1. Different Contraceptive Methods

Contraception is the use of methods and techniques with the aim of impeding sexual relations from resulting in pregnancy. It is a family planning resource for constituting desired and consciously planned reproduction. There are currently numerous known contraceptive strategies, which may be grouped into the following categories: behavior-based methods, barrier methods, intrauterine devices (IUD), hormonal methods, and surgical methods.

Hormonal methods include combined (containing estrogen and progestin) and progestin-only methods. The former include combined pills, vaginal rings, patches, and monthly ingestions. Progestin-only methods include progestin-only pills, quarterly injections, etonogestrel subdermal implant, and levonorgestrel-releasing IUD.

Understanding that different means of contraception present different mechanisms of action, adverse event profiles, beneficial non-contraceptive effects, which vary according to any given clinical context, is the basis for selecting the most appropriate contraceptive method; it is also indispensable to evaluate patients’ wishes and expectations, in addition to their beliefs regarding the method, in order to optimize adherence.

In order to choose a contraceptive method, should be considerer 1) safety supported on the into medical eligibility criteria of available methods 2) clinical condition of patiente; 3) effectiveness, determined by the number of failures (i.e. pregnancies) that occur in every 100 women utilizing the method for 12 months, which is known as the Pearl index^417^ (Figure 14).

**Figure 14:** **–** Pearl indexes of the main contraceptive methods. Adapted from Curtis et al.^417^

Patients with severe diseases that contraindicate pregnancy or patients who wish to postpone or avoid pregnancy should receive adequate counseling regarding contraception.^418^ Furthermore, patients with contraindications to gestation have higher surgical risks; for this reason, permanent methods (laparotomic, laparoscopic, or hysteroscopic tubal ligation) are not any more recommended than any other highly efficacious methods.

In recent years, special attention has been given to long-acting reversible contraception (LARC) methods. These methods have greater adherence because they do not depend on the user remembering them; furthermore, they have greater contraceptive efficacy, with a lower number of failures, and they do not contain estrogen. This category includes both types of IUD (copper and levonorgestrel) and etonogestrel subdermal implant.^419,420^

### 6.2.2. Medical Eligibility Criteria

The WHO has analyzed the safety of different contraceptive methods, taking each clinical condition and their relevant characteristics into consideration, including the following: whether the method worsens a preexisting condition or adds additional health risks; and whether the condition renders the contraceptive method less effective.^421^ Safety should always be weighed when comparing the risk of an unplanned pregnancy. It is fundamental to remember that refusing patients access to all contraceptive methods due to concerns related to diseases they have increases the risk of decompensating these diseases should pregnancy occur.

Table 48 shows a summary of the categories of medical eligibility criteria for contraceptive choice.

**Table 48:** – Categories of medical eligibility criteria for contraceptive choice.

Condition for which there is no restriction regarding use of the contraceptive method
Condition where the advantages of using the method generally outweigh theoretical or proven risks
Condition where the theoretical or proven risks outweigh the advantages of using the method
Condition that represents an unacceptable health risk if the contraceptive method were used

*Adapted from the World Health Organization, 2015.^421^*

Accompanying women of fertile with heart disease requires decisions on the application of family planning methods and, therefore, contraception counseling. A pioneering study on the efficacy and safety of contraceptives that included low-dose combined oral contraceptives, quarterly injection of progestin, and IUD in women with heart disease showed good tolerance and safety for patients who followed the eligibility criteria.^422^

### 6.2.3. Contraception in Adverse Conditions

***6.2.3.1. Hypertension***

In patients with hypertension, the use of combined contraceptive methods may worsen blood pressure control. Ethinylestradiol increases the hepatic synthesis of angiotensinogen, which leads to an increase in angiotensin II and aldosterone, with higher systolic volume and greater cardiac output, as well as increased peripheral vascular resistance, thus resulting in greater arterial pressure. In susceptible patients, this increase may be considerable, causing clinical decompensation.^423^ For this reason, patients with hypertension, even if it is controlled, should not use combined methods; there is, however, no contraindication to the use of progestin-only methods in patients with controlled hypertension, and, in patients with uncontrolled hypertension, only quarterly injections should be avoided. Table 49 shows the medical eligibility criteria for different types of contraception in relation to patients with SAH.

**Table 49:** – Medical eligibility criteria for different types of contraception in relation to patients with systemic arterial hypertension

	Combined hormonal contraception				Progestin-only contraception			Intrauterine device	

	Oral	Patch	Vaginal ring	Monthly injection	Oral	Quarterly injection	Subdermal implant	Copper	Levonorgestrel
History of SAH where blood pressure is not known	3	3	3	3	2	2	2	1	2
Controlled SAH	3	3	3	3	1	2	1	1	1
SAH with elevated blood pressure – SAP 140 to 159 mmHg and/or DAP 90 to 99 mmHg – SAP ≥ 160 mmHg and/or DAP ≥ 100 mmHg	3	3	3	3	1	2	1	1	1
	4	4	4	4	2	3	2	1	2
Target organ disease	4	4	4	4	2	3	2	1	2

*DAP: diastolic arterial pressure; SAH: systemic arterial hypertension; SAP: systemic arterial pressure. Adapted from the World Health Organization, 2015.^421^*

***6.2.3.2. Diabetes Melittus***

Patients with diabetes are at a greater risk of cardiovascular events than healthy women, and they are more exposed to unfavorable outcomes during pregnancy.^424^ For this reason, contraception in patients with diabetes should be guided by the best available evidence.^425^Table 50 summarizes the eligibility criteria for different contraceptive methods in patients with diabetes.

**Table 50:** – Medical eligibility criteria for different types of contraception in relation to patients with diabetes

	Combined hormonal contraception			Progestin-only contraception			Intrauterine device	
	
Oral	Patch	Vaginal ring	Monthly injection	Oral	Quarterly injection	Subdermal implant	Copper	Levonorgestrel
Without vascular lesion	2	2	2	2	2	2	1	2
2								
Nephropathy, neuropathy, or retinopathy	3 / 4	3 / 4	3 / 4	2	3	2	1	2
3/4								
Other vascular disease	3 / 4	3 / 4	3 / 4	2	3	2	1	2
3/4								
or > 20 years’ disease duration								

*Adapted from the World Health Organization. Medical Eligibility Criteria for Contraceptive Use. 5th ed. Geneva: World Health Organization; 2015.^421^*

There is a theoretical concern that, due to its glucocorticoid effect, quarterly depot injections of medroxyprogesterone acetate may worsen glycemic control, and, in patients with vasculopathy, they may increase the risk of thromboembolic and cardiovascular events; for this reason, it is classified as category 3.

***6.2.3.3. Heart Valve Disease***

Complicated heart valve diseases are included in the WHO list of conditions that expose women to greater health risks due to undesired pregnancy.^415,426^ Nevertheless, several studies have shown expressively low rates of use of contraceptive methods in women with heart disease.^422,427^ To comprehend the criteria summarized in Table 51, heart valve diseases are divided into complicated and uncomplicated. Those that are accompanied by PH, risk of AF, and history of subacute bacterial endocarditis are considered complicated. Table 51 shows the medical eligibility criteria for different types of contraception in relation to patients with heart valve disease.

**Table 51:** – Medical eligibility criteria for different types of contraception in relation to patients with heart valve disease

	Combined hormonal contraception				Progestin-only contraception			Intrauterine device	

	Oral	Patch	Vaginal ring	Monthly injection	Oral	Quarterly injection	Subdermal implant	Copper	Levonorgestrel
Uncomplicated	2	2	2	2	1	1	1	1	1
Complicated	4	4	4	4	1	1	1	2	2

*Adapted from the World Health Organization. Medical Eligibility Criteria for Contraceptive Use. 5th ed. Geneva: World Health Organization, 2015.^421^*

Currently, the indication for antibiotic prophylaxis during IUD insertion is controversial, and the available evidence does not seem to justify making it mandatory. Deciding whether or not to use it is at the attending physician’s discretion, considering associated risks and benefits. It is, however, indispensable to remember that the best way to avoid pelvic infection is by performing adequate antisepsis.

***6.2.3.4. Previous Cardiovascular Events***

Women with ischemic coronary disease or stroke may safely initiate progestin-only contraceptive methods, with the exception of the quarterly injection. However, if events occurred after hormonal contraceptive use, it should be changed to a non-hormonal method. In this clinical context, combined methods, also should be avoided.^428,429^Table 52 shows the medical eligibility criteria of different contraceptive methods in relation to patients with previous cardiovascular events.

**Table 52:** – Medical eligibility criteria for different types of contraception in relation to patients with previous cardiovascular events

	Combined hormonal contraception				Progestin-only contraception			Intrauterine device	

	Oral	Patch	Vaginal ring	Monthly injection	Oral	Quarterly injection	Subdermal implant	Copper	Levonorgestrel
Ischemic heart disease	4	4	4	4	I: 2, C: 3	3	I: 2, C: 3	1	I: 2, C: 3
Stroke	4	4	4	4	I: 2, C: 3	3	I: 2, C: 3	1	2

*I: initiation; C: continuation. Adapted from the World Health Organization, 2015.^421^*

***6.2.3.5. Obesity***

In the absence of other clinical conditions, obese patients do not have contraindications to the use of any method. Furthermore, even if it is necessary to investigate metabolic syndrome and screen for other cardiovascular conditions due to obesity, the results of complementary examinations should not delay the introduction of contraceptive methods.^430^

With respect to quarterly injections (150-mg doses of intramuscular depot medroxyprogesterone acetate), there is a Brazilian study showing significantly higher weight gain in women using quarterly injections, in comparison with copper IUD.^431^ For this reason, quarterly injections are not typically the first choice; there is, however, no formal contraindication, and the method may be used.

Specifically in obese women, there is a theoretical concern that methods may be less efficacious. Even if this is the case, their efficacy continues to be high; for this reason, they should not be contraindicated.

***6.2.3.6. Congenital Heart Disease***

Contraception counseling in patients with congenital heart disease begins at menarche, with advice regarding the risks of gestation and choice of contraception method. Congenital heart diseases are not explicitly listed in the WHO’s eligibility criteria, and they should be understood within the physiopathology of each group of heart disease and the risk of unplanned pregnancy (Table 53). Complex congenital heart diseases present diverse structural lesions which complicate risk stratification of contraceptive use.^415,427^ In any event, cyanotic heart diseases, diseases with PAH, Eisenmenger syndrome, and diseases with an elevated risk of thromboembolism have an absolute contraindication to the use of combined methods. For these groups of patients in WHO classes III and IV, the use of progestin-only methods is recommended, and monthly injections are recommended in cases with low risks of tromboembolism.^432-434^Due to pain issues, when patients have more delicate heart conditions that occur with the risk of arrhythmias, the IUD should be inserted in a hospital environment, with the possibility of prompt relief provided by an anesthesiologist with experience in women with heart disease, due to the risk of vagal reaction following IUD insertion.

**Table 53:** – Recommendations for contraceptive use in patients with congenital heart disease

	OC	Progestin-only pill	Implant	Depo-Provera	IUD	Barrier
1. Surgically corrected defects:						
Without residual lesions: IAC/IVC/PDA	1	1	1	1	1	
Shunt and/or residual obstruction	3	1	1	1	3	1
Prosthetic valve, tubes, patches	2	1	1	1	2	1
Pulmonary and/or systemic hypertension	4	2	2	2	3	1
2. Uncorrected, residual, or postoperative defects:						
Small IVC	2	1	1	1	4	1
Mild to moderate shunt (IAC, IVC, PDA)	4	1	1	1	4	2
Residual pulmonary or systemic hypertension (CoA)	2	1	1	1	4	3
Complex cyanotic defects	4	1	1	1	4	1
3. Complicated defects due to:						
Cyanosis	4	1	1	1	-	1
Ventricular dysfunction	3	1	1	1	-	1
Atrial fibrillation/flutter	4	2	2	2-	4	2
Eisenmenger syndrome	4	2	2	2	4	4

*CoA: Coarctation of the aorta; IAC: interatrial communication; IUD: intrauterine devices; IVC: interventricular communication; OC: oral contraceptives; PDA: patent ductus arteriosus.*

***6.2.3.7. Pulmonary Hypertension***

As the literature is very limited, eligibility criteria for patients with PAH are not included. For this reason, contraception should be effective, tolerable, and non-harmful, because all patients with PAH should be advised against becoming pregnant. For this reason, barrier methods or “fertility-awareness” based methods are not recommended, because they have very elevated failure rates. Among reversible hormonal contraceptives, estrogen-containing compounds are not recommended due to the risk of PTE, leaving progestin-only methods, which may be injectable, oral, or via subcutaneous implantation, which is the most indicated.^419,420^ Copper-T IUD pose a risk of metrorrhagia, while long-acting reversible contraceptive (LARC) methods with levonorgestrel may be recommended when the patient does not present structural cardiac injury.

Unplanned pregnancy is very frequent in women with heart disease, especially due inadequate contraception counseling. In fact, myths about the eventual risks and lack of knowledge about the efficacy and application of eligibility criteria are factors which favor maternal mortality. Faced with this reality, contraception counseling regarding preferences, contraindications, and efficacy of methods should be initiated during the immediate postpartum period, even before hospital discharge.^435^

### 6.2.4. Contraception and Adolescence

Age alone does not represent a contraindication to different methods of contraception; nevertheless, during adolescence, doubts may arise regarding strategies for presenting and prescribing contraceptives. Indication of methods should be based on eligibility criteria and, when attending adolescents, it is necessary to consider ethical and legal aspects, which are not always known.

Article 226 of the Brazilian Constitution guarantees the right to family planning free of coercion, and the Child and Adolescent Statute (Law Number 8069, July 13, 1990) clearly addresses important issues in providing care to adolescents who require contraceptive methods, based on privacy and confidentiality rights.

Adolescent patients have the right to privacy, i.e., to be attended alone, in a private consultation space. Confidentiality is defined as an agreement between physicians and patients, meaning that information discussed during and after consultation may not be disclosed to adolescents’ parents or guardians without their express consent.^420^

Confidentiality is supported by rules of medical bioethics, through moral principles of autonomy (article 103 of the Code of Medical Ethics). In this manner, adolescents have the right to sexual education, access to information about contraception, confidentiality and secrecy regarding their sexual activity, and the prescription of contraceptive methods; there are no ethical infractions when medical professionals proceed in this manner.

Contraceptive counseling involving short-lasting methods such as pills is generally applied without problems following these precepts. On the other hand, in relation to long-lasting methods (intrauterine methods and implants), as they require medical procedure for insertion, doubts may arise. The Brazilian Federation of Gynecological and Obstetric Associations (FEBRASGO, acronym in Portuguese) suggests that, for these methods, the consent of adolescents and their legal guardians should be considered, reinforcing contraceptive counseling.^436^

With respect to adolescents with heart disease, contraception should be safe and effective; there is, however, a great barrier to the knowledge of different options and their access, often due to the high initial costs. During contraception counseling for adolescents with WHO risks III/IV for pregnancy, it is necessary to present all available methods with low Pearl indexes, good tolerance, and acceptance for continuity of the method, such as IUD and implants. Nevertheless, the most popular forms of contraception in adolescents continue to be condoms and withdrawal, which represent a high rate of unplanned pregnancy and high risk.

Lack of knowledge, inadequate counseling, social-cultural taboos, legal restrictions, and moralistic attitudes regarding sexuality during adolescence are common, even in patients who choose or wish to use a contraceptive method. Although long-acting methods (IUD and implant) are prioritized by medical entities,^419^ difficulties in access and acceptance by adolescents demonstrate that traditional methods, such as combined oral contraceptives and condoms, should receive focus during counseling, with the aim of improving rates of continuity and, in final analysis, reducing the possibility of high-risk pregnancies and maternal mortality due to heart disease.

***6.2.4.1. Key Points***

- There are numerous contraceptive methods (behavior-based, barrier, IUD, hormonal, and surgical) that may be prescribed to women with heart disease;
- Choice of contraceptive methods should consider patients individually, including their wishes and tolerance, as well as the eligibility criteria proposed by the WHO;
- Ethical and legal aspects should be considered regarding contraception in adolescents.

## 6.3. Ethical Considerations

The advances in medicine transformed Michel Peter’s proverb, *“Women with heart disease, don’t get married, and, if you do, don’t get pregnant,”* outdated. That was the case two centuries ago for preserving the lives of young women with heart disease. We are currently living in a new era, in which the risk of pregnancy is generally lower, with resources to cope with most complications that may eventually occur.

Heart disease and pregnancy should be a comprehensive topic based on medical ethics, integrating several moments during which the multidisciplinary interface is stablished in the care of pregnant women and her child. Physicians should apply scientific rigor, based on validated clinical recommendations, clarify the benefits and possible risks and respect the patient's right to participate freely and actively in the decision-making process, obtaining consent informed for all decisions.

From moments before conception there have been situations related to maternal and fetal safety. Heart evaluation may reveal different degrees of risk due to pathological, clinical and therapeutic situations. Decision-making results in possible conflicts in the doctor-patient relationship, which require the application of bioethics fundamentals. Prudence must prevail. Therapeutic measures should consider the informed consent of the patient, which is based on their right to answer yes or no.

Furthermore, qualification of multidisciplinary teams is fundamental to family planning in young women with heart disease, based on maternal risk stratification, regarding the article 226 of the Brazilian Constitution, which states the following: “Based on the principles of human dignity and responsible parenthood, family planning is a free choice of the couple, it being within the competence of the State to provide educational and scientific resources **for the exercise of this right, any coercion by official or private agencies being forbidden” (our emphasis added). This norm refers to other items: a) dignity of the human person (Article 1, III) and b) right to liberty (Article 5, Heading).”

During pregnancy, the doctor-patient relationship requires total reception by the doctor and adherence by the patient, obviously with adequate availability of institutional resources and the health system.

Interdisciplinary team is desirable at all times of pregnancy and postpartum period; however, it expands its value in the approximation of childbirth, when it is essential the professional competence of the care team. The decision of moment and type of delivery, the search for technological and infrastructure support in general are well assisted by the application on bioethics fundaments.

The puerperium has specific peculiarities and the mother with heart disease demands a higher level of care than usual, while the newborn already has a life of her own, with her particular demands. Thus, there are conflicts, such as non-consent for a medical instruction, it is up to the doctor – or the Service – to make a critical reassessment, based on bioethics at the Bedside for the specific case. The agreement made with the patient must be strictly adhered to by the doctor.
